# Essential and toxic metal concentrations in biological samples of multiple sclerosis patients: A systematic review and meta-analysis

**DOI:** 10.1371/journal.pone.0313851

**Published:** 2024-12-06

**Authors:** Alireza Kooshki, Reyhane Farmani, Alireza Amirabadizadeh, Omid Mehrpour, Mohammad Javad Sanjari, Samaneh Nakhaee

**Affiliations:** 1 Student Research Committee, Birjand University of Medical Sciences, Birjand, Iran; 2 Research Institute for Endocrine Sciences, Student Research Committee, Endocrine Research Center, Shahid Beheshti University of Medical Sciences, Tehran, Iran; 3 Medical Toxicology and Drug Abuse Research Center (MTDRC), Birjand University of Medical Sciences, Birjand, Iran; 4 Poison & Drug Information Center, Wayne State University, School of Medicine, Detroit, MI, United States of America; Kermanshah University of Medical Sciences, ISLAMIC REPUBLIC OF IRAN

## Abstract

The role of trace elements and toxic metals on human health has been extensively discussed concerning disease pathogenesis and risk factors of diseases. In this systematic review and meta-analysis, we aimed to investigate arsenic (As), cadmium (Cd), lead (Pb), iron (Fe), zinc (Zn), and magnesium (Mg) levels in individuals with multiple sclerosis (MS) and healthy controls. We searched different databases/search engines for this systematic review and meta-analysis, including Web of Science, PubMed, Scopus, and Google Scholar, until June 27, 2024. Out of 5466 studies identified, 65 met our eligibility criteria and were included in the systematic review. For the meta-analysis, 58 studies with 10420 participants (5316 multiple sclerosis patients and 5104 healthy controls) had adequate data for inclusion. Results from the pooled data, analyzed using a random-effects model, revealed higher levels of As (Hedge’s g = 4.00 μg/l, 95% CI = 2.03 to 5.98, P <0.001; I^2^ = 97.69%, P<0.001) and Cd (Hedge’s g = 1.20 μg/l, 95% CI = 0.13 to 2.27, P = 0.028; I^2^ = 97.99%, P<0.001) in multiple sclerosis patients compared to healthy ones. However, no significant differences were observed in the concentrations of Zn, Fe, Mg, and Pb between the two groups. This study identified elevated As and Cd levels in MS patients, indicating the need for targeted interventions and public health guidelines for toxic metal exposure. Limiting exposure to contaminated environments and maintaining essential element levels through natural resources or supplements is essential, as there may be a possible relationship between multiple sclerosis and the concentrations of these elements in humans.

## 1. Introduction

Considered the most common non-traumatic disabling disease and involving young adults aged between 20 and 40, MS is a chronic inflammatory demyelinating disease of the central nervous system (CNS) [1,2]. Due to remarkable disability caused by sensation, motor, and autonomic deficiencies, MS is known to be an autoimmune disease mediated by T-cells [2]. MS is classified into four clinical subtypes: progressive relapsing MS (PRMS), relapsing-remitting MS (RRMS), primary progressive MS (PPMS), and secondary progressive MS (SPMS) [3]. An increasing incidence of uncertain etiology has been reported in developing and developed countries [4]. At least 350,000 individuals in the United States and 93,500 Canadians have been affected by this progressive disease, with an increase of 2.5 million cases globally every year [2,5,6]. Interactions have been observed between environmental, dietary, and immunological factors and MS [7,8].

Trace elements and some toxic metals are involved in the pathogenesis of MS [9,10]. Toxic metals can exert their toxic effects through multiple mechanisms, such as the formation of free radicals, enzyme inhibition, and cell membrane disturbance [11]. Some studies have proposed metal-dependent autoimmunity and the role of exposure and genetics in determining individual sensitivity and the ability to detoxify these metals [11,12]. Some toxic metals, including As, Pb, and Cd, were investigated in this study because of their roles in MS pathogenicity. These metals are preventable due to their enormous industrial usage [13]. They have no biological function and are toxic even at low dosages [14].

It has been reported that Cd can damage the brain via reactive radical formation and interfere with antioxidant enzyme activity [15]. It is a potentially toxic metal that can cause some serious cardiovascular, hepatic, and renal conditions [16]. It can also interfere with MS pathogenesis through oxidative stress or increased lipid peroxidation in the plasma and cerebral cortex [16].

Pb is a well-known toxic metal that threatens public health and is widely involved in the pathogenesis of MS. Accumulation of Pb in cells has been associated with the development of autoantibodies against different cellular structures such as neurofilaments, neuronal cytoskeletal proteins, and myelin basic protein [17,18]. Pb can also interfere with the absorption of trace elements [6]. In return, deficiency in trace elements such as Zn, iron (Fe), and Cu can make humans vulnerable to toxic metals and increase their toxicity [19].

Trace elements are also involved in different pathophysiological mechanisms of MS [9]. Magnesium (Mg) inhibits nerve cells and decreases the stimulation effect of the autonomic ganglia by preventing excessive stimulation due to its competitive nature with calcium [20]. In contrast, Fe is one of the chief trace elements that accomplish vital rules in the brain [21]. Fe is critical for the mitochondrial metabolism and myelination of neural cells [20]. However, abnormalities in Fe metabolism can lead to Fe deposition in the brain. This accumulation of Fe can interfere with MS pathogenesis through multiple pathways, such as lipid peroxidation and cell death [21]. Zn, a cofactor for more than 300 enzymes, is critical for all organisms [22]. Since homeostasis of this essential element is important for the human brain, altering this crucial element can affect nervous or immune system functionality, as lower levels of Zn can induce neural cell apoptosis. In return, high levels of Zn can cause neurotoxicity [22].

Since 1970, many studies have investigated the association between certain elements and MS pathogenesis; however, this issue has remained unresolved for five decades, with the available data being inconclusive. Our systematic review and meta-analysis aim to make significant advances over prior studies by addressing key gaps identified in the literature. Unlike previous review studies, such as those by Stojsavljević et al. (2024) [23] and Sarihi et al. (2021) [24], which focused on different toxic metals and essential elements, our study has a broader scope and includes more biological samples and a longer search period. In contrast, our review aims to examine a wider range of elements, including three toxic metals (Cd, Pb, As) and three essential elements (Fe, Zn, Mg), across various body fluids (serum, whole blood, plasma, and cerebrospinal fluid [CSF]). Furthermore, our meta-analysis takes a novel approach by conducting subgroup analyses based on the economic status of the countries where each study was conducted, comparing developing and developed countries to gain insights into potential economic differences in study quality. By explaining these previous studies’ limitations and employing novel methodological techniques, our study tends to provide a more thorough understanding of the potential impact of essential elements and toxic metals in MS.

## 2. Methods

### 2.1. Literature search

This systematic review and meta-analysis were performed according to Preferred Reporting Items for Systematic Reviews and Meta-Analyses (PRISMA) criteria. A comprehensive search of the databases was carried out by two independent individuals until June 27, 2024, using both keywords and Medical Subject Headings (MeSH) terms. The following MeSH terms were employed in our search query: multiple sclerosis, iron, zinc, cadmium, arsenic, magnesium, lead, heavy metals, and trace elements. The MeSH terms and keywords were searched in the title or abstract of the studies using four databases and search engines: Web of Science, PubMed, Scopus, and Google Scholar. The search strategies used in different databases are listed in Table 1. The search was performed using EndNote software X8. All literature search results were de-duplicated and imported into EndNote for subsequent screening. Hand searching was the primary method used to identify the studies. The review’s protocol was registered in PROSPERO (The International Prospective Register of Systematic Reviews) under the registration number 586199.

**Table 1:** Search strategies in different databases for retrieving the relevant documents.

Database/ search engine	Search strategy	Results
**Pub Med**	(((multiple sclerosis[MeSH Terms]) OR ("multiple sclerosis"[Title/Abstract])) AND (((((((((((((((((((((((iron[MeSH Terms]) OR (zinc[MeSH Terms])) OR (cadmium[MeSH Terms])) OR (arsenic[MeSH Terms])) OR (Magnesium[MeSH Terms])) OR (lead[MeSH Terms])) OR (elements, trace[MeSH Terms])) OR (heavy metals[MeSH Terms])) OR (iron[Title/Abstract])) OR (zinc[Title/Abstract])) OR (magnesium[Title/Abstract])) OR (arsenic[Title/Abstract])) OR (cadmium[Title/Abstract])) OR (trace element[Title/Abstract])) OR (trace elements[Title/Abstract])) OR (heavy metal[Title/Abstract])) OR (heavy metals[Title/Abstract])) OR (zn[Title/Abstract])) OR (fe[Title/Abstract])) OR (mg[Title/Abstract])) OR (pb[Title/Abstract])) OR (cd[Title/Abstract])) OR (trace elements[Pharmacological Action])))	1869
	("lead"[Title/Abstract] AND ("multiple sclerosis"[MeSH Terms] OR "multiple sclerosis"[Title/Abstract])) AND (2021:2022[pdat])	438
**Scopus**	(TITLE-ABS ("multiple sclerosis")) AND ((CHEMNAME (lead)) OR (TITLE-ABS ("heavy metal*" OR "trace element*" OR "chemical element*" OR cadmium OR arsenic OR zinc OR iron OR magnesium)))	1078
**Web of Science**	ti = (("multiple sclerosis") AND ("heavy metal*" OR "trace element*" OR "chemical element*" OR cadmium OR arsenic OR lead OR zinc OR iron OR magnesium))	459
	ab = ("multiple sclerosis") AND (ti = (lead) OR ab = ("heavy metal*" OR "trace element*" OR "chemical element*" OR cadmium OR arsenic OR pb OR zinc OR iron OR magnesium))	1060
**Google Scholar**	allintitle:(multiple sclerosis)("trace element" OR "trace elements" OR "heavy metal" OR "heavy metals" OR iron OR zinc OR cadmium OR arsenic OR magnesium OR lead)	562

#### 2.1.1 Inclusion criteria for study selection

The present systematic review and meta-analysis include human studies that have measured at least one of the six elements (Pb, As, Cd, Fe, Zn, Mg) in the serum, plasma, whole blood, or CSF and have both MS and healthy control groups for comparison. The results from the databases were consolidated, and duplicates were eliminated. The remaining studies were screened based on their titles and abstracts, and those not meeting the inclusion criteria were excluded. The remaining studies were subsequently examined based on their full text or sufficient abstracts. The included studies were case-controls, cross-sectionals, and cohorts. As studies were predominantly conducted on adults due to the nature of the disease, we did not specify an age limit for this review. No restrictions were imposed on the language or time frame of the studies, and all types of MS (PRMS, RRMS, PPMS, and SPMS) were included. The references of relevant studies were also assessed for additional related studies. Studies that provided the mean and standard deviation or median and interquartile range (IQR) for the concentration of the elements in both the control and MS groups were included in our meta-analysis. Some articles did not provide the essential data for meta-analysis and were only included in our systematic review.

#### 2.1.2 Exclusion criteria for study selection

Studies that only investigated the concentrations of selected elements (Zn, Fe, Mg, Cd, As, and Pb) in patient groups without a comparison or control group were excluded. Review studies, letters to the editor, books, animal studies, *in vitro* studies, randomized control trials, and conference papers were excluded, as were articles that did not provide sufficient data.

### 2.2. Data extraction

The data from studies were extracted and shown in Table 2. An electronic data abstraction form was used to record study characteristics, including the first author, year of publication, country, study type, diagnostic criteria for MS, sample size, mean age, gender, type of MS, assessment method for different elements, type of body fluid, and the main results.

**Table 2:** Characteristics included human studies for assessments of toxic and essential element concentrations in biological samples of multiple sclerosis and healthy individuals (data extracted by AK, SN and RF on June and July of 2024).

First author (year)	Country	Type of study	Diagnostic criteria of MS	Sample size (control, case)	Mean age (control, case)	Gender F/M (control, case)	Type of MS	Assessment method	Body fluid	Main result	Eligibility
Alimonti A (2007) [25]	Italy	Case-control	McDonald’s criteria	124,60	44.8±12.7 38.5±10.4	43/81, 38/20	RR-MS & SP-MS	ICP-AES ICP-MS	Serum	Fe– Zn– Mg = Pb = Cd +	Included in SR and MA
Aliomrani M (2017) [26]	Iran	Case-control	McDonald’s criteria	74,69	31.8±10.3 35.2±10.9	36/38, 58/11	RR-MS	GFAAS	Whole Blood	Pb = Cd + As +	Included in SR and MA
Bahrampour Juybari K (2018) [8]	Iran	Case-control	McDonald’s criteria	50,50	45.3±15.3 49.6±13.5	37/13 35/15	RR-MS	AAS	Serum	As +	Included in SR and MA
Dehghanifiroozabadi M (2019) [27]	Iran	Case-control	Method proposed by Poser [28]	29,29	38.61±6.50 36.27±8.77	22/7, 22/7	RR-MS	GFAAS	Whole Blood	Pb +	Included in SR and MA
Nashmi A (2020) [29]	Iraq	Case-control	NM	25,25	41.12±10.11 42.46±9.3	14/11, 15/10	RR-MS & SP-MS & PP-MS	FAAS GFAAS Semi-automated enzymatic measurement	Serum	Pb + Cd + Zn -	Included in SR and MA
Forte G (2005) [7]	Italy	Case-control	McDonald’s criteria	69,60	38.4±9.7 38.7±9.9	NA 40/20 (matched)	MS	ICP-AES ICP-MS	Plasma	Mg = Cd = Pb– Zn– Fe –	Included in SR and MA
Ghoreishi A (2015) [30]	Iran	Case-control	NM	50,50	32±2.65 32±3.35	NM	MS	Polarography	Serum	Pb = Zn + Cd +	Included in SR and MA
Giacoppo S (2014) [31]	Italy	Case-control	NM	33,41	35.7±7.31 40.63±9.66	16/17, 31/10	MS	ICP-MS	Plasma	Zn = Pb =	Included in SR and MA
Janghorbani M (2017) [32]	Iran	Case-control	McDonald’s criteria	90,55	45.1±15.7 31.6±14.83	67/28, 47/8	RR-MS & SP-MS & PP-MS	ICP-AES	Plasma	Fe– Zn– Mg + Pb = Cd -	Included in SR and MA
Madeddu R (2011) [33]	Italy	Case-control	McDonald’s criteria	22,29	59±18 50±12	11/11, 21/8	RR-MS & SP-MS & PP-MS	SF-ICP-MS	CSF	Cd = Fe = Zn = Pb =	Included in SR and MA
Paknejad B (2019) [34]	Iran	Case-control	McDonald’s criteria	74,62	33.83±10.36 35.33±11.0	36/38, 52/10	RR-MS	GFAAS	Serum	Cd + Pb = As +	Included in SR and MA
Visconti A (2005) [35]	Italy	Case-control	Clinical evidence (based on MRI findings)	12, 12	28.3±8.8 28.2±7.9	7/5,8/4	MS	SF-ICP-MS ICP-AES	Serum	Cd + Fe– Mg + Pb = Zn =	Included in SR and MA
Yousefi B (2014) [16]	Iran	Case-control	Clinical evidence	38, 38	NM 31±7.5	38/0, 38/0	MS	GFAAS	Serum	As +	Included in SR and MA
Oliveira M (2020) [36]	Brazil	Case-control	McDonald’s criteria	30, 30	22 to 57 25 to 63	15/15, 23/7	MS	ICP-MS	Whole Blood	Zn = Pb + Mg– Fe –	Included in SR and MA
Abo-Krysha N (2008) [37]	Egypt	Case-control	Clinical evidence (based on MRI findings, clinical and examination), method proposed by Poser (107)	10, 20	NM 29.94±8.84	10/0, 20/0	RR-MS & PP-MS & SP-MS	bathophenanthroline	Serum	Fe =	Included in SR and MA
Akbay GD (2020) [38]	Turkey	Case-control	NM	56, 56	NM	42/14, 42/14	MS	spectrophotometry	Serum	Mg–	Included in SR and MA
Al-Radaideh A (2021) [39]	Jordan	Case-control	McDonald’s criteria	34, 65	34±7.1 32±6.3	18/16, 41/24	RR-MS	spectrophotometry	Serum	Fe–	Included in SR and MA
Algül S (2021) [40]	Turkey	Case-control	NM	20, 20	NM	NM	MS	FAAS	Plasma	Fe– Zn–	Included in SR and MA
Alizadeh A (2016) [41]	Iran	Case-control	NM	30, 26	31.33 28.13	16/14, 22/4	RR-MS	GFAAS	Serum	Zn = Pb = Mg -	Included in SR
Armon-Omer A (2019) [42]	Israel	Case-control	Clinical evidence (based on clinical, laboratory, MRI findings)	83, 63	40.6 ± 11.9 44.7 ± 14.0	49/34, 42/21	MS	NM	Serum	Fe– Mg =	Included in SR and MA
Bergsland N (2017) [43]	Italy	Case-control	NM	24, 22	50.1±11.3 46.3±10.0	17/7, 13/9	MS	colorimetric end-point kit	Serum	Fe =	Included in SR and MA
Dore-Duffy P (1983) [44]	USA	Case-control	Clinical evidence	60, 68	NM	30/30, 50/18	MS	AAS	Plasma	Zn =	Included in SR and MA
										Zn =	Included in SR and MA
									Serum		Included in SR and MA
Elberry RA (2020) [45]	Egypt	Case-control	Clinical evidence	50, 50	NM	NM	RR-MS & PP-MS & SP-MS & PR-MS	NM	Whole Blood	Pb +	Included in SR and MA
Gellein K (2008) [46]	Norway	Case-control	NM	13, 9	49.4±3.6 43.9±2.8	8/5, 8/1	MS	HR-ICP-MS	Whole Blood	Cd = Fe = Zn = Mg = Pb =	Included in SR and MA
									Plasma	Cd = Fe = Zn = Pb = Mg =	Included in SR and MA
									CSF	Cd = Fe = Zn = Pb = Mg =	Included in SR and MA
									Serum	Fe =	Included in SR and MA
Ghazavi A (2012) [47]	Iran	Case-control	McDonald’s criteria	60, 60	36.4 ±18 34.02 ±17	38/22, 43/17	RR-MS & SP-MS	colorimetric method	Serum	Zn–	Included in SR and MA
Moradi A (2016) [48]	Iran	Case-control	NM	25, 25	NM	NM	MS	ICP	Serum	Cd + Pb + Fe– Zn –	Included in SR
Nasrabadi M N (2011) [49]	Iran	Case-control	NM	20, 40	NM	16/4, 31/9	RR-MS	neutron activation analysis	Whole Blood	Zn– Fe =	Included in SR
Bsteh G (2019) [50]	Austria	Cross-sectional	McDonald’s criteria	16, 71	31.8±11.6 46.19±7.83	8/8, 52/19	aRR-MS iRR-MS aPMS iPMS	NM	Serum	Fe =	Included in SR and MA
Kurup RK (2002) [51]	India	Case-control	Method proposed by Poser [28]	15,15	25 to 30 NA	NM (matched)	RMS	AAS	Plasma	Mg -	Included in SR and MA
Mezzaroba L (2020) [52]	Brazil	Case-control	McDonald’s criteria	182, 174	39.5±9.8 41.9±13.2	128/54, 121/53	CIS RR-MS PPMS SPMS	FAAS	Serum	Zn–	Included in SR and MA
Taheri M (2015) [53]	Iran	Case-control	NM	43, 33	NM	NM 25/8 (matched)	MS	AAS	Serum	Zn–	Included in SR
Thamer AA (2014) [54]	Iraq	Case-control	McDonald’s criteria	50, 112	33.54±8.4 33.48±8.09	33/17 77/35	MS	AAS	Serum	Zn– Mg –	Included in SR and MA
Harbige L S (2011) [55]	England	Case-control	NM	9, 21	25 to 45 22 to 68	NM	RR-MS	ICP-MS	Plasma	Zn =	Included in SR and MA
Linke E (1977) [56]	Germany	Case-control	NM	24, 40	NM	NM	MS	AAS	CSF	Zn + Mg =	Included in SR and MA
The Birmingham research unit (1976) [57]	England	Case-control	Clinical evidence	21, 21	NM	15/6 15/6	MS	AAS	Whole Blood	Pb =	Included in SR and MA
Palm R (1982) [58]	Sweden	Case-control	McDonald’s criteria	50, 50	NM 36.9 (matched)	29/21 29/21	MS	FAAS	Serum	Zn-	Included in SR and MA
Pawlitzki M (2018) [59]	Germany	Case-control	McDonald’s criteria	50,151	43 ± 14 55 ± 9 (PMS) 42 ± 11(RMS)	38/12 18/0 (PMS) 95/38 (RMS)	PMS RMS	AAS	Serum	Zn-	Included in SR and MA
Rieder H.P (1983) [60]	Switzerland	Case-control	Clinical evidence	35, 119	NM	21/14 66/53	MS	AAS	Whole Blood	Fe = Zn+	Included in SR and MA
Ristori G (2011) [61]	Italy	Cohort	Clinical evidence	49, 49	33.2±6.1 36.1±6.9	26/23 29/20	CIS	mass and optical spectrometry	Serum	Cd+ Mg+ Zn+ Fe- Pb =	Included in SR and MA
Sedighi B (2013) [62]	Iran	Case-control	McDonald’s criteria	39, 58	NM	30/9 48/10	RR-MS	AAS	Serum	Zn =	Included in SR and MA
Sfagos C (2005) [63]	Greece	Case-control	Clinical evidence (based on history, neurological examination, and EDDS)	40, 27	NM 37.5 ± 5.5 (matched)	NM 10/17 (RR-MS) 30/10 (CP-MS) (matched)	RR-MS CP-MS	NM	Serum	Fe =	Included in SR and MA
Shaheen H (2006) [64]	Egypt	Case-control	Clinical evidence, the method proposed by Poser [28]	15,40	NM 30.53±8.14	NM 30/10 (matched)	SP-MS RR-MS PP-MS	AAS	Serum	Zn =	Included in SR and MA
Siotto M (2019) [65]	Italy	Case-control	Clinical evidences, Method proposed by Polman [66]	40, 60	40.3 ± 10.86 37.2 ± 9.06	22/18 45/15	RR-MS	photometric test	Serum	Fe =	Included in SR and MA
Smith D.K (1989) [67]	USA	Case-control	Clinical evidence (based on the laboratory), method proposed by Poser [28]	33, 27	NM	11/22	MS	AAS	Plasma	Zn =	Included in SR and MA
Socha K (2017) [68]	Poland	Case-control	McDonald’s criteria, Clinical evidences	68, 101	40.1 ± 13 40.9 ± 10.2	47/21 64/37	RR-MS	AAS	Serum	Zn-	Included in SR and MA
Stelmasiak Z (1995) [69]	Poland	Case-control	Method proposed by Poser [28]	20, 24	21 to 45 (mean 33) 29 to 60 (mean 37)	10/10 17/7	MS	colorimetric	Plasma	Mg-	Included in SR and MA
Moghtaderi A (2015) [70]	Iran	Case-control	McDonald’s criteria, method proposed by Polman [66]	61, 59	31.54 ± 13.51 (included both case and control)	31/30 41/18	MS RR-MS	AAS	CSF	Fe =	Included in SR and MA
Iranmanesh F (2012) [71]	Iran	Case-control	McDonald’s criteria	30, 30	NM 35.2 (Matched)	NM 26/4 (Matched)	RR-MS SP-MS PP-MS CP-MS	colorimetric	Serum	Fe =	Included in SR and MA
Iranmanesh M (2012) [72]	Iran	Case-control	McDonald’s criteria, Clinical evidences (based on MRI findings)	25, 25	(men with MS) 28±3.44 (women with MS)24±2.55 (men in control)28 ±4.85 (women in control) 24 ±2.31	16/9 16/9	RR-MS	colorimetric	Serum	Fe+ Zn-	Included in SR and MA
Karpińska E (2017) [73]	Poland	Case-control	McDonald’s criteria	41, 101	40.09 ± 14.1 40.86 ± 10.2	29/12 64/37	RR-MS	FAAS	Serum	Mg =	Included in SR and MA
Ellidag H Yaser (2014) [74]	Turkey	Case-control	McDonald’s criteria	35,35	38 ± 10 38 ± 11	22/13 20/15	RR-MS	assay kits (Abbott)	Serum	Fe =	Included in SR and MA
Markovska O (2017) [75]	Poland	Case-control	McDonald’s criteria	32, 110	NM 38.1 (matched)	NM 67/43 (matched)	PP-MS RP-MS RMS	colorimetric	Whole Blood	Mg + Fe -	Included in SR and MA
Masoud S A (2007) [76]	Iran	Case-control	Clinical evidence (based on MRI findings)	35, 35	NM 32.3 ± 6.4 (matched)	NM 28/7 (matched)	RR-MS SP-MS	autoanalyzer	Serum	Mg- Zn-	Included in SR and MA
Matar A (2020) [77]	Lebanon	Case-control	McDonald’s criteria	42, 27	42.8 ± 12.9 38.3 ± 16.0	14/13 29/13	RR-MS	colorimetric	Serum	Zn = Fe =	Included in SR and MA
Oraby M I (2019) [78]	Egypt	Case-control	McDonald’s criteria	25, 50	28.28±7.08 30.88 ± 9.01(Relapsing MS) 32.28 ±8.07 (Remission MS)	14/11 21/4 (Relapsing MS) 16/9 (Remission MS)	Relapsing MS Remission MS	colorimetric	Serum	Zn-	Included in SR and MA
Kapaki E (1989) [79]	Greece	Case-control	NM	28, 15	46 34	10/18 3/12	MS	AAS	Serum	Zn = Mg =	Included in SR and MA
									CSF	Zn = Mg =	Included in SR and MA
Heipertz R (1979) [80]	Germany	Case-control	NM	112, 69	17 to 79 NM	NM	MS	AAS	CSF	Mg =	Included in SR and MA
MELØ T M (2002) [81]	Norway	Case-control	Method proposed by Poser [28], clinical evidence (based on MRI findings)	19, 18	NM (matched)	NM (matched)	RR-MS PC-MS	HR-ICP-MS	CSF	Zn =	Included in SR and MA
van Rensburg S J (2006) [82]	South Africa	Case-control	McDonald’s criteria	22, 26	NM (matched)	NM (matched)	MS	Vitros 250 Chemical analyzer	Serum	Fe -	Included in SR and MA
Akcin s 2024 [83]	Turkey	Case-control	McDonald’s criteria	30, 30	32.30±2.42 35.93±1.92	22/8, 24/6	PR-MS	Zn: photometric Fe, Mg: routine Abbote measurement kit	Serum	Zn = Fe = Mg =	Included in SR
Mahmoud HR 2023 [84]	Egypt	Case-control	NM	30, 60	NM	NM	MS(30 SPMS and 30 RRMS)	NM	Serum	Zn =	Included in SR
Mohamed SR 2022 [85]	Egypt	Cross-sectional	McDonald’s criteria	86, 86	31.71 ± 6.32 33.63 ± 7.4	57/29, 53/33	PPMS RRMS SPMS	ICP-MS	Whole blood	Pb + Cd + Zn -	Included in SR
Pomary PC 2023 [86]	Spain	Case-control	McDonald’s criteria	13, 18	33.38 ± 10.3 49.33 ± 9.6	10/3, 11/7	PPMS	ICP-MS	CSF	Fe = Zn = Mg - Pb +	Included in SR and MA
				13, 40	33.38 ± 10.3 46.14 ± 9.66	10/3, 13/9	SPMS		CSF	Fe = Zn = Mg = Pb =	Included in SR and MA
Jabbar AA 2023 [87]	Iraq	Case-control	NM	25, 25	37.36 ± 8.29 39.26 ± 19.59	9/16, 13/12	NPMS PMS	Spectrophotometry	Serum	Zn -	Included in SR
Stojsavljevi’c A 2024 [88]	Serbia	Case-control	McDonald’s criteria	100, 215	42.18 ± 9.43 46.79 ± 8.22	66/34, 120/95	MS(150 RRMS, 52 SPMS, and 13 PPMS)	ICP-MS	Serum	Zn -	Included in SR and MA

F/M (Female/Male), MS (Multiple sclerosis), NM (Not Mentioned), RRMS (remitting relapsing MS), PC-MS (Primary chronic), SP-MS (secondery progressive MS), PMS (progressive MS), CP-MS (chronic progressive MS), PP-MS (primary progressive MS), CP-MS (Chronic progressive MS), MRI (Magnetic resonance imaging), AAS (Atomic absorption spectrometry), CSF (cerebrospinal fluid), ICP (individually coupled plasma), iPMS (inactive PMS), aPMS (active PMS), iRR-MS (inactive RR-MS), aRR-MS (active RR-MS), GFAAS (graphite furnace AAS), FAAS (flame AAS), EAAS (electrothermal AAS), -,+, = (respectively indicate, significantly lower, higher and same level of that element in samples of MS patients compared to controls), McDonald’s criteria (Guideline for establishing MS diagnosis that include physical examination, symptoms, and laboratory results such as MRI data and lumbar puncture), EDDS (is measured by neurologists based on muscle function, eye sight, tremor, balance and … during physical examination), systematic review (SR), Meta-analysis (MA).

### 2.3. Quality assessment

A predefined checklist was used to check the quality of the included studies. Two reviewers (AK and SN) evaluated each study individually using the Joanna Briggs Institute (JBI) critical assessment tool. The discrepancy was corrected by a third researcher (OM). The JBI tool contains components requiring two "yes/no" answers. A "yes" answer indicates one score, and a "no" / unclear answer indicates a zero point [89]. No identified studies that met our research criteria were excluded after the quality evaluation (see S1 File).

### 2.4. Statistical analysis

Meta-analysis was performed by STATA version 17.0. The heterogeneity of included studies was assessed using the I-squared (I^2^) and Chi-square-based Q-test. If a considerable heterogeneity (I^2^ statistic more than 70% and *p*-value of Q-test < 0.1) was observed, we analyzed the pooled estimates using a random-effects model. We also reported the possibility of publication bias using the Egger and Begg test.

## 3. Results

### 3.1. Selection of studies

**Fig 1:**
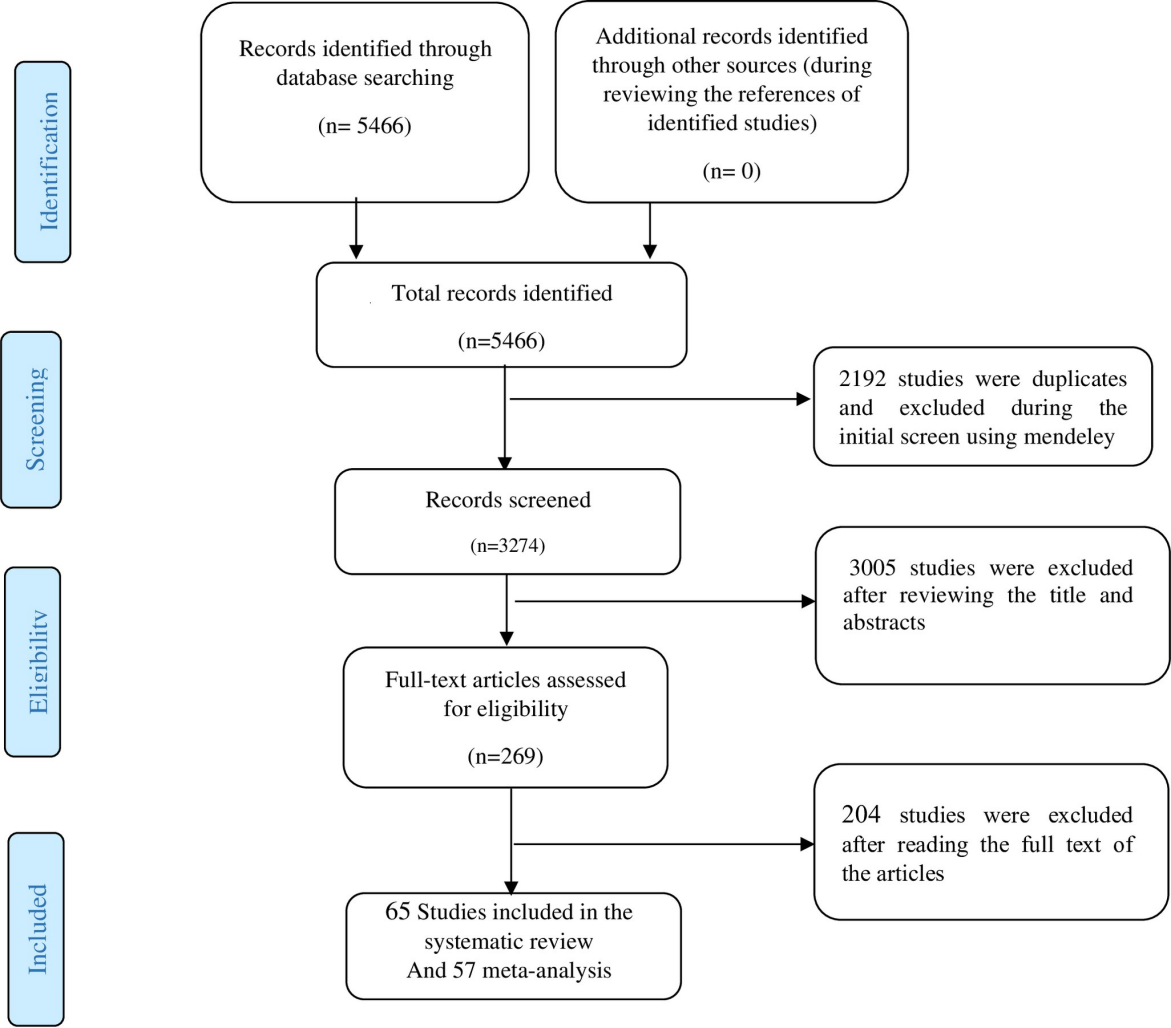
PRISMA flowchart of the literature search and strategy for selecting relevant documents.

A total of 5466 studies were obtained from various databases (see S2 File), and 2192 studies were removed as duplicates. Next, we scanned the titles and abstracts, and 3005 studies were excluded because some were reviews, letters, books, duplicates, or did not meet our inclusion criteria. After checking the full text of related studies, 204 were removed. Finally, 65 studies met our inclusion criteria and were included in the quality assessment and systematic review (Fig 1).

### 3.2 Study characteristics

Sixty-five studies in the systematic review measured at least one of the six elements (Pb, As, Cd, Fe, Zn, and Mg) in the serum, plasma, whole blood, or CSF of patients with MS and healthy individuals. Among these studies, 57 had sufficient data for the analysis and were included in the meta-analysis [7,8,16,25–27,29–40,42–47,50–52,54–61,63–65,67–83,86,88]. These studies were published between 1976 and 2024, and among sixty-five included studies, two of them are cross-sectional studies, one is a cohort study, and the rest are case-control studies. Concentrations of different elements were assessed using different methods such as inductively coupled plasma (ICP) [7,25,31–33,35,36,46,48,55,81,85,86,88] or atomic absorption spectroscopy (AAS) [8,16,26,27,29,34,40,41,44,51–54,56–60,62,64,67,68,70,79,80,90]. Most studies have used McDonald’s criteria [91] to diagnose MS.

### 3.3. Systematic review

#### 3.3.1. Cadmium (Cd)

Cd concentration was measured in fifteen studies for patients with MS and healthy individuals (2 studies used CSF [33,46], and 13 studies used blood [whole blood = 3 [26,46,85], plasma = 3 [7,32,46], and serum = 7 [25,29,30,34,35,48,61]]).

Thirteen studies measured Cd levels in the blood. In nine studies (7 case-control studies, one cross-sectional study, and 1 cohort study), Cd levels were higher in the MS group (2 in whole blood [26,85], and 7 in serum [25,29,30,34,35,48,61]). In contrast, three case-control studies did not find any significant differences between the MS group and the healthy group (1 in whole blood [46] and 2 in plasma [7,46]). However, only one case-control study among these twelve reported a drop in plasma Cd levels in MS patients [32].

Both case-control studies that measured Cd levels in CSF reported no significant differences between the control and MS groups [33,46].

#### 3.3.2. Arsenic (As)

Four studies quantified As in the blood of MS patients and healthy controls (four studies used whole blood [whole blood = 1 [26], and serum = 3 [8,16,34]]). All studies were case-control and reported higher levels of As in the MS group compared to the control group (n = 4) [8,16,26,34].

#### 3.3.3. Lead (Pb)

Twenty studies measured Pb levels in various body fluids of patients with MS and healthy controls (17 used blood [whole blood = 7, serum = 8, plasma = 3], and three used CSF) [7,25–27,29–36,41,45,46,48,57,61,85,86]. It is important to note that some studies measured element concentrations in two or more body fluids.

Seventeen of these studies examined blood Pb levels. There were six studies (5 case-control studies and one cross-sectional study) that showed significantly higher levels of Pb in patients with MS (4 in whole blood [27,36,45] and 2 in serum [29,48]). In contrast, twelve studies (11 case-control studies and 1 cohort study) found the same levels of Pb in both MS patients and healthy controls (3 in whole blood [26,46,57], 6 in serum [25,30,34,35,41,61], and 3 in plasma [31,32,46]), and one case-control study found lower levels of Pb plasma in MS patients [7].

Three case-control studies measured Pb levels in CSF, and two did not report any significant difference between the MS and control group [33,46], while the other reported increased Pb levels in PPMS patients and non-significant Pb levels in SPMS patients [86].

#### 3.3.4. Zinc (Zn)

Forty-one studies assessed Zn levels in the body fluids of patients with MS and healthy controls (36 used blood [whole blood = 5, serum = 26, and plasma = 7], and five used CSF) [7,25,29–33,35,36,40,41,44,46–49,52,54–56,58–62,64,67,68,72,76–79,81,83–88,92,93]. Findings of three studies (2 case-control studies and 1 cohort study) showed that blood Zn levels of patients with MS were higher than those of healthy individuals (2 in serum [30,61], 1 in whole blood [60]). In contrast, sixteen case-control studies did not show any significant differences between these two groups (9 in serum [35,41,44,62,64,77,79,83,84], 5 in plasma [31,44,46,55,67], and 2 in whole blood [36,46]). There were nineteen studies (15 case-control studies and one cross-sectional study) that reported blood Zn levels of patients with MS were lower compared to healthy controls (15 in serum [25,29,47,48,52,54,58,59,68,72,76,78,87,88,92], 2 in plasma [32,40], and 2 in whole blood [49,93]). Five case-control studies measured Zn levels in CSF [33,46,56,79,86]. Three of them reported the same levels of Zn in the CSF of the MS and control groups [33,46,79,86], and one found significantly lower Zn levels in MS patients [56].

#### 3.3.5. Iron (Fe)

Thirty-one studies assessed Fe levels in MS patients and a healthy control group (27 used blood [whole blood = 5, serum = 17, and plasma = 5], and four used CSF). Fourteen studies (13 case-control studies and one cross-sectional study) observed the equivalent levels of Fe in both groups (10 in serum [37,46,50,63,65,71,77,83,94,95], 1 in plasma [46], 3 in whole blood [46,49,60]), whereas twelve (11 case-control studies, and 1 cohort study) reported lower Fe levels in patients with MS (6 in serum [25,35,39,42,61,82], 4 in plasma [7,32,40], 2 in whole blood [36,75]). In comparison, only one study reported higher Fe levels in patients with MS [72].

In all four case-control studies that measured Fe levels in CSF, there was no significant difference between the levels of Fe in patients with MS and the control group [33,46,70,86].

#### 3.3.6. Magnesium (Mg)

Twenty-three studies measured Mg levels in MS patients and healthy controls (19 used blood[whole blood = 3, serum = 11, and plasma = 5], and four used CSF). Seven case-control studies reported equivalent levels of this essential element in both groups (4 in serum [25,42,79,83,90], 2 in plasma, and 1 in whole blood [46]). In comparison, eight case-control studies observed lower Mg levels in patients with MS (5 in serum [38,41,54,76], 2 in plasma [7,46], and 1 in whole blood [36]). In contrast, four studies (3 case-control studies and 1 cohort study) reported higher levels of Mg in patients with MS compared to healthy individuals (2 in serum [35,61], 1 in plasma [32], and 1 in whole blood [75]).

Four case-control studies that measured CSF Mg levels reported no significant differences between the MS and control groups [46,56,79,80]. One study reported lower Mg levels in PPMS patients and no significant difference between SPMS patients and the health control group [86].

### 3.4 Meta-analysis

#### 3.4.1. Cadmium (Cd)

**Fig 2:**
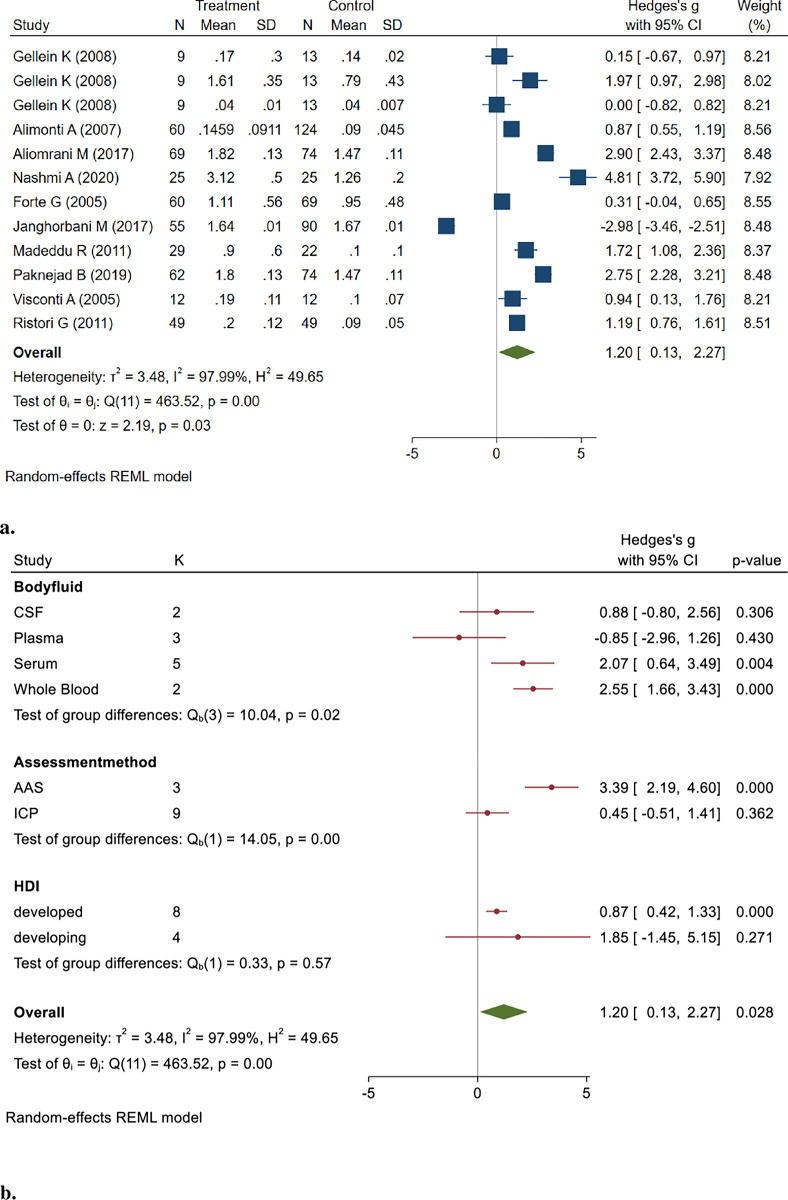
**a&b.** Point and Pooled estimates of Hedge’s *g* effect size with 95% confidence intervals of Cd concentration in patients with Multiple sclerosis compared to healthy controls in the subgroups of different body fluids (CSF, plasma, serum, whole blood) and assessment methods (AAS, ICP) using random model. Heterogeneity indices, as well as the *p*-value for Cochran’s Q-test of heterogeneity, are also shown. (Cd = Cadmium, ICP = Individually Coupled Plasma, AAS = Atomic Absorption Spectrometry, CSF = Cerebrospinal Fluid, NM = Not Mentioned, HDI = Human Development Index).

Cd concentrations in 448 MS patient samples compared to 578 healthy controls using the random effect model were pooled, and the results showed that the Cd levels in MS patients were significantly higher than those in controls. (Hedges’ g: 1.20, 95% CI: 0.13, 2.27, *P* = 0.028) (Fig 2).

The pooled studies were heterogeneous (I^2^ = 97.99%, Q = 463.52, *p* < 0.001). The results of the Egger test (z = 1.16, *P* = 0.2473) indicated no publication bias.

The sensitivity analysis demonstrated that excluding certain studies altered the statistical significance of the overall effect. Notably, when the study by Nashmi A (2020) was excluded, the previously significant pooled effect estimate became non-significant (Hedges’ g: 0.89, 95% CI: -0.08, 1.86, p = 0.07) [29]. These findings indicate that this study may heavily influence the overall significance of the meta-analysis results, suggesting a potential source of bias or heterogeneity.

The results of subgroup analysis based on different samples showed that the pooled concentrations of Cd in serum (Hedges’ g: 2.07, 95% CI: 0.64, 3.49, *p* = 0.004) and whole blood (Hedges’ g: 2.55, 95% CI: 1.66, 3.43, *p* < 0.001) were significantly higher in patients with MS. In addition, subgroup analysis based on the assessment method showed that Cd concentration significantly differed in the AAS method (Hedges’ g: 3.39, 95% CI: 2.19, 4.60, *p* < 0.001) but not in the ICP method. The sub-group analysis of the human development index (HDI) showed significantly higher Cd levels in MS patients in developed countries than in controls (Hedges’ g: 0.87, 95% CI: 0.42, 1.33, *p* < 0.001).

#### 3.4.2. Arsenic (As)

**Fig 3:**
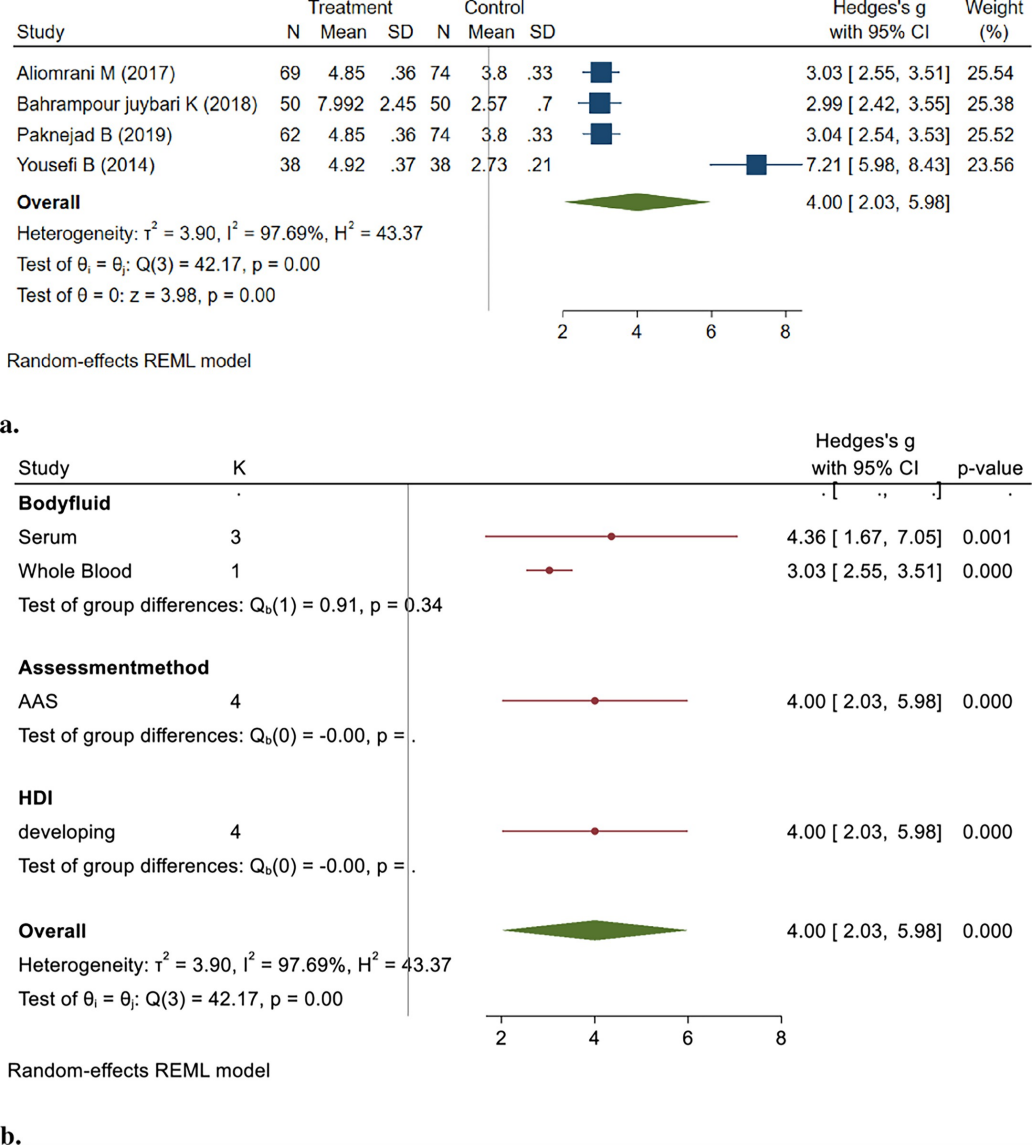
**A&b**. Point and Pooled estimates of Hedge’s *g* effect size with 95% confidence intervals of As concentrations in patients with Multiple sclerosis compared to healthy controls in the subgroups of different body fluids (CSF, plasma, serum, whole blood) using a random model. Heterogeneity indices, as well as the *p*-value for Cochran’s Q-test of heterogeneity, are also shown. (As = Arsenic, CSF = Cerebrospinal Fluid, HDI = Human Development Index).

Four studies measured As levels in the blood of 219 patients with MS and 236 controls showed a significantly higher As level in the blood of patients with MS (Hedges’ g: 4.00, 95% CI: 2.03, 5.98, *P*<0.001) (Fig 3).

Pooled studies showed heterogeneity (I^2^ = 97.69%, Q = 42.17, *p* < 0.001). The results of the Begg test (z = 1.02, *P* = 0.3082) indicated no publication bias.

To assess the robustness of the pooled results, we conducted a sensitivity analysis by systematically removing each study from the meta-analysis. The findings remained consistent, indicating that no single study disproportionately influenced the pooled effect estimate (Hedges’ g: 4.37, 95% CI: 1.70, 7.05, *P* = 0.001).

#### 3.4.3. Lead (Pb)

**Fig 4:**
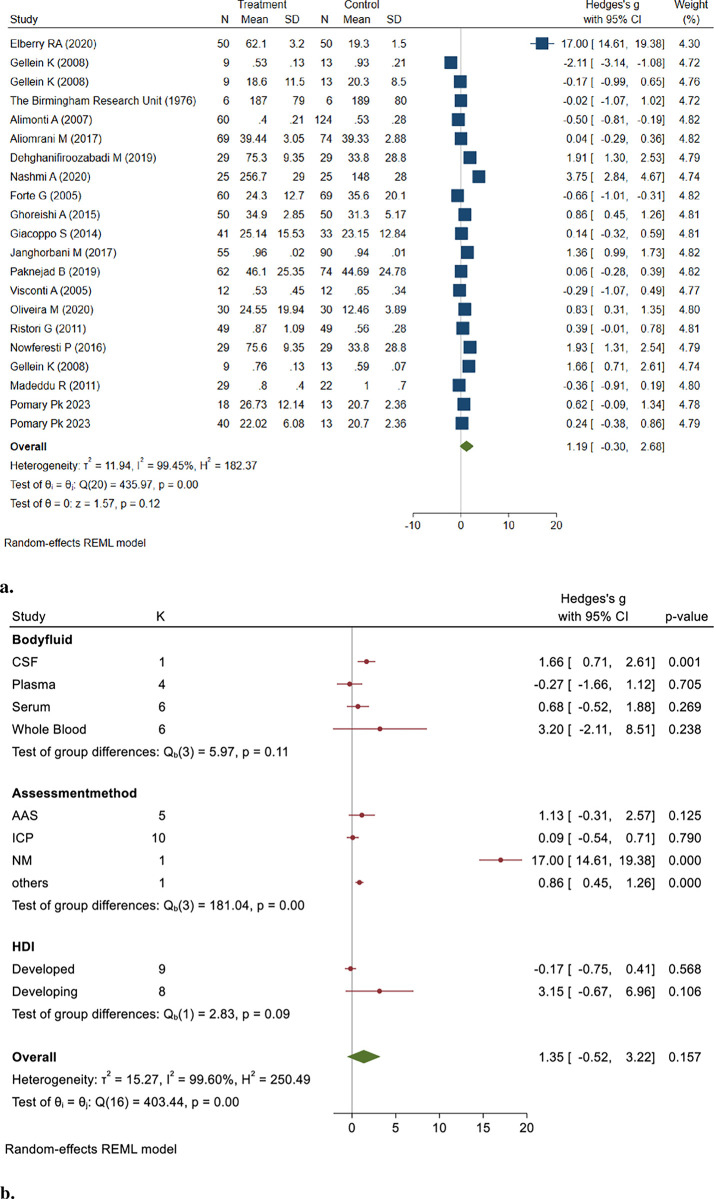
**a&b.** Point and Pooled estimates of Hedge’s *g* effect size with 95% confidence intervals of Pb concentrations in patients with Multiple sclerosis compared to healthy controls in the subgroups of different body fluids (CSF, plasma, serum, whole blood) and assessment methods (AAS, ICP) using random model. Heterogeneity indices and the *p*-value for Cochran’s Q-test of heterogeneity are also shown. (Pb = lead, ICP = Individually Coupled Plasma, AAS = Atomic Absorption Spectrometry, CSF = Cerebrospinal Fluid, NM = Not Mentioned, HDI = Human Development Index).

This section included 712 MS patient samples and 789 control samples. The results of the random effect model showed no significant difference in Pb concentrations between MS patients and healthy controls (Hedges’ g: 1.19, 95% CI: -0.30, 2.68, *P*>0.05) (Fig 4).

The pooled studies were heterogeneous (I^2^ = 99.45, Q = 435.97, *p* = 0.00). Based on the results of the Egger test (z = 5.40, *P*<0.001), there was a publication bias.

To assess the robustness of the pooled results, we conducted a sensitivity analysis by systematically removing each study from the meta-analysis. The findings remained consistent, indicating that no single study disproportionately influenced the pooled effect (Hedges’ g: 1.36, 95% CI: -0.17, 2.84, *P* = 0.15).

Subgroup analysis indicated a higher level of Pb in the CSF of patients with MS than in healthy controls (Hedges’ g: 1.66, 95% CI: 0.71, 2.61, *P* = 0.001). The results of the Egger test (z = 5.73, *P* = 0.000) indicated publication bias.

#### 3.4.4. Zinc (Zn)

**Fig 5:**
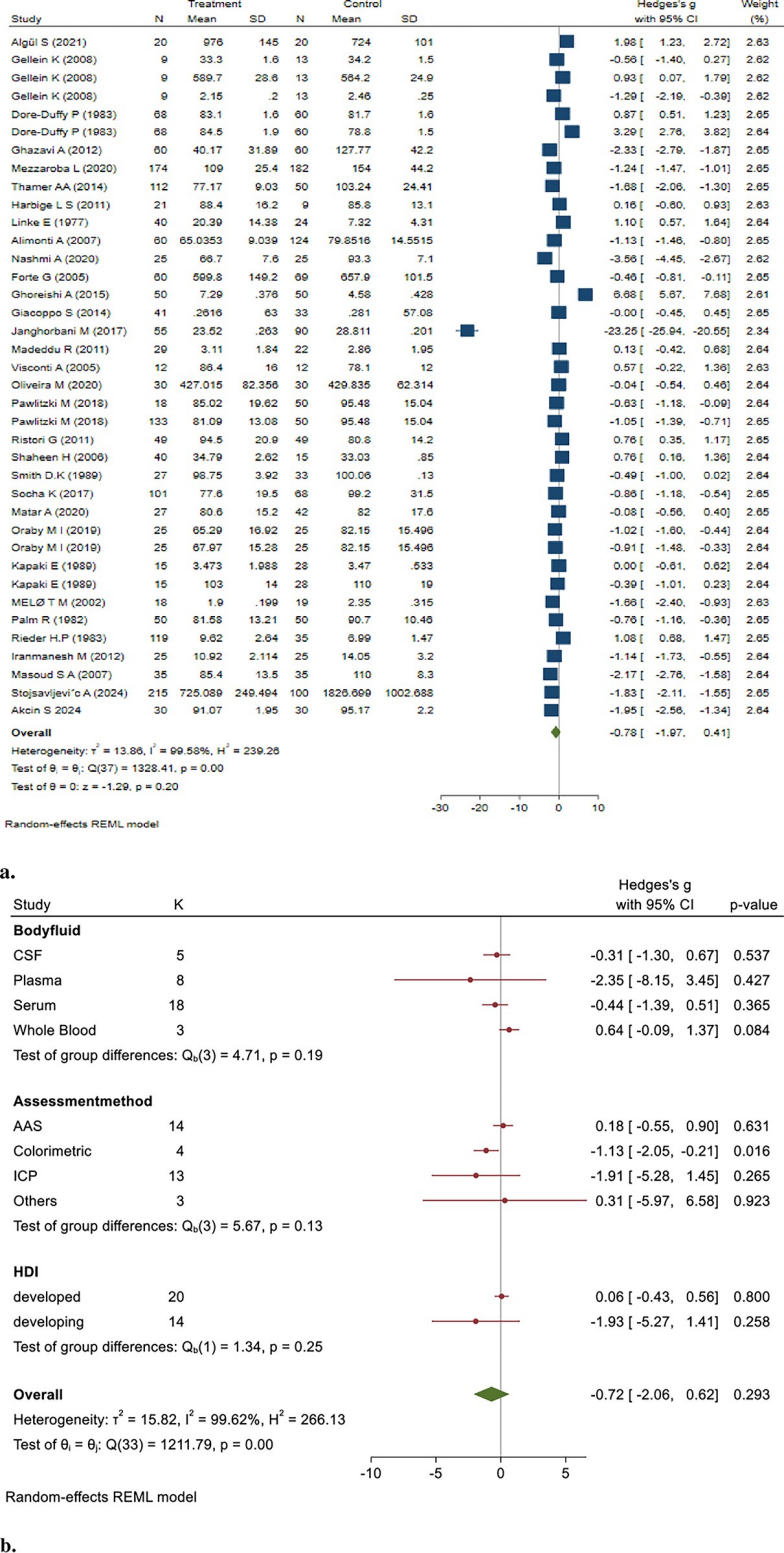
**a&b.** Point and Pooled estimates of Hedge’s *g* effect size with 95% confidence intervals of Zn concentrations in patients with Multiple sclerosis compared to healthy controls in the subgroups of different body fluids (CSF, plasma, serum, whole blood) and assessment methods (AAS, ICP)using random model. Heterogeneity indices, as well as the *p*-value for Cochran’s Q-test of heterogeneity, are also shown. (Zn = Zinc, ICP = Individually Coupled Plasma, AAS = Atomic Absorption Spectrometry, CSF = Cerebrospinal Fluid, NM = Not Mentioned, HDI = Human Development Index).

Among the 1919 MS patient samples and 1591 control samples, there was no significant difference between the two groups (Hedges’ g: -0.78, 95% CI: -1.97, 0.41, *P* = 0.198) (Fig 5).

The pooled studies were heterogeneous (I^2^ = 99.58, Q = 1328.41, *p* = 0.00). Publication bias was found using the Egger test (z = -5.93, *P* = 0.000).

To assess the robustness of the pooled results, we conducted a sensitivity analysis by systematically removing each study from the meta-analysis. The findings remained consistent, indicating that no single study disproportionately influenced the pooled effect (Hedges’ g: -0.97, 95% CI: -2.12, 0.48, *P* = 0.22).

The results of the subgroup analysis indicated that studies that used the colorimetric method for measuring Zn levels found lower Zn levels in MS patient samples than in healthy controls (Hedges’ g: -1.10, 95% CI: -1.82, -0.37, *P* = 0.003).

#### 3.4.5. Iron (Fe)

**Fig 6:**
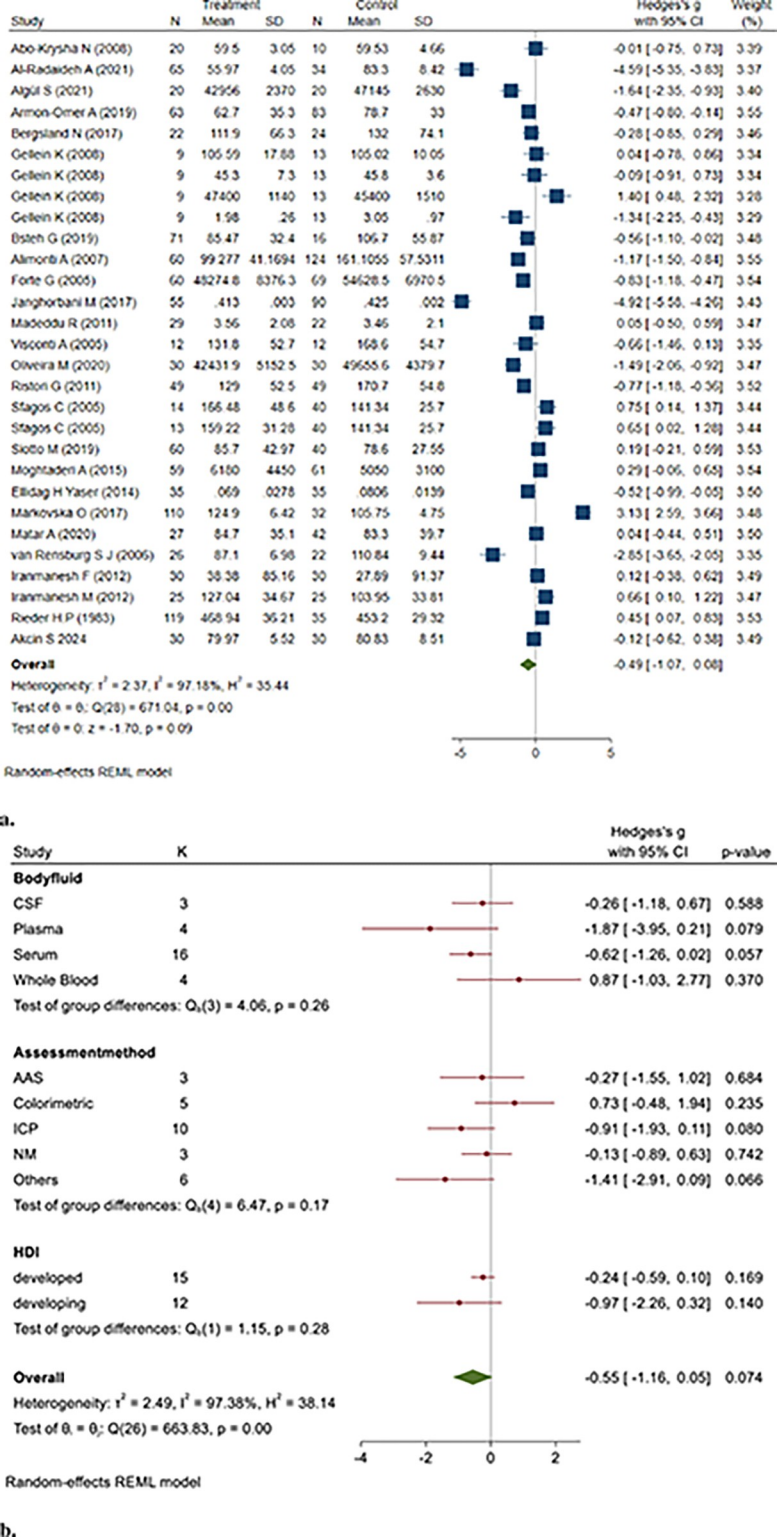
**a&b.** Point and Pooled estimates of Hedge’s *g* effect size with 95% confidence intervals of Fe concentrations in patients with Multiple sclerosis compared to healthy controls in the subgroups of different body fluids (CSF, plasma, serum, whole blood) and assessment methods (AAS, ICP) using random model. Heterogeneity indices and the *p*-value for Cochran’s Q-test of heterogeneity are also shown. (Fe = Iron, ICP = Individually Coupled Plasma, AAS = Atomic Absorption Spectrometry, CSF = Cerebrospinal Fluid, HDI = Human Development Index).

We analyzed 1140 MS patient samples and 1027 controls. The results of the random effect model indicated no significant difference in Fe levels between MS patients and healthy controls. (Hedges’ g: -0.49, 95% CI: -1.07, 0.08, *P* = 0.090) (Fig 6).

The pooled studies were heterogeneous (I^2^ = 97.18, Q = 671.04, *p* = 0.00). We also found no publication bias using the Egger test (z = -1.05, *P* = 0.2923). Subgroup analysis based on different variables showed no significant differences.

The sensitivity analysis revealed that the exclusion of certain studies impacted the statistical significance of the overall effect. Specifically, when the study by Markovska O (2017) was removed, the pooled effect estimate became statistically significant (Hedges’ g: -0.62, 95% CI: -1.15, -0.10, *P* = 0.02) [75].

These results suggest that the inclusion of this study contributed to the non-significant overall result, indicating potential heterogeneity.

#### 3.4.6. Magnesium (Mg)

**Fig 7:**
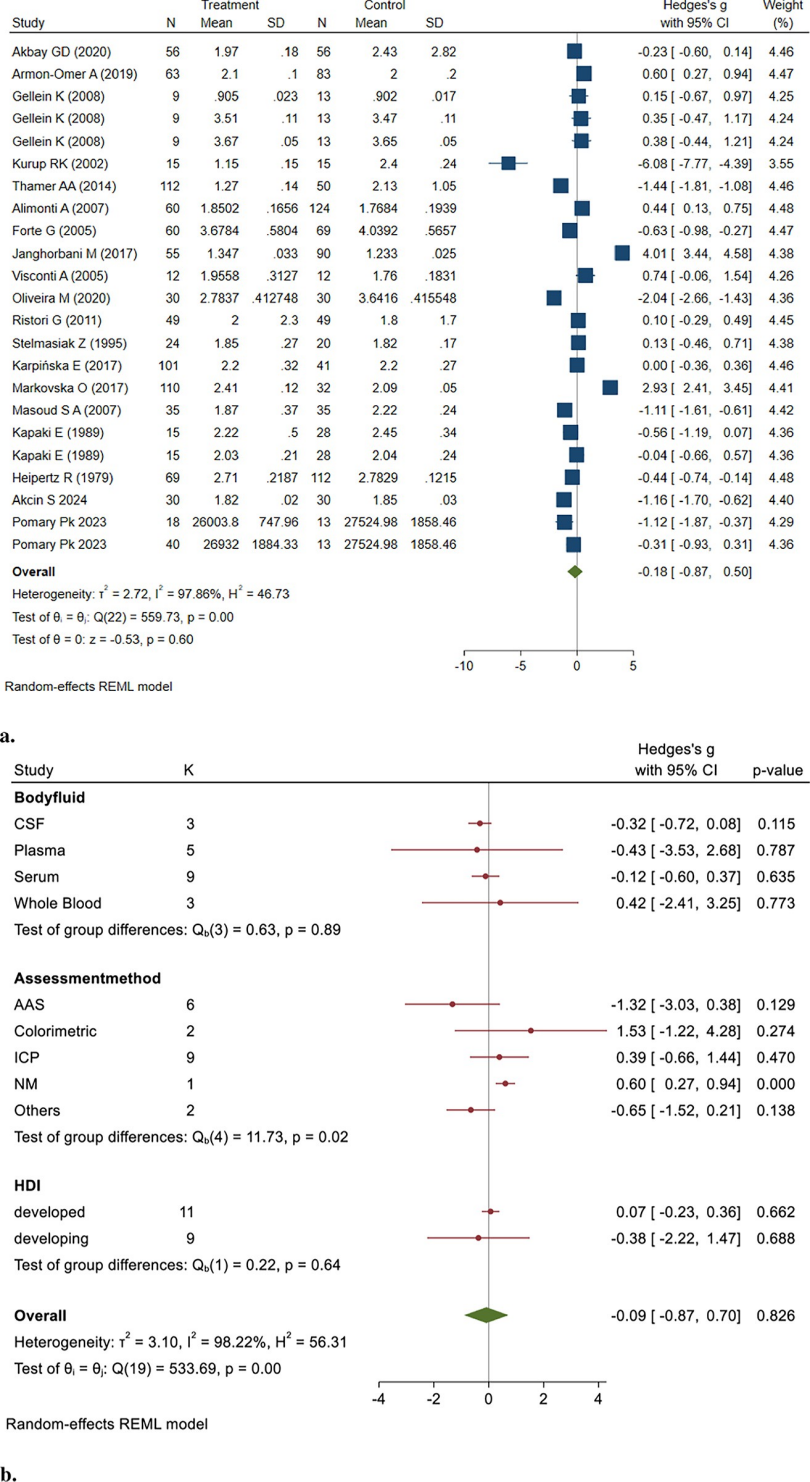
**a&b.** Point and Pooled estimates of Hedge’s *g* effect size with 95% confidence intervals of Mg concentrations in patients with Multiple sclerosis compared to healthy controls in the subgroups of different body fluids (CSF, plasma, serum, whole blood) and assessment methods (AAS, ICP) using random model. Heterogeneity indices and the *p*-value for Cochran’s Q-test of heterogeneity are also shown. (Mg = Magnesium, ICP = Individually Coupled Plasma, AAS = Atomic Absorption Spectrometry, CSF = Cerebrospinal Fluid, NM = Not Mentioned, HDI = Human Development Index).

This section compared 996 MS patient samples and 956 healthy control samples. The results of the random effect model showed no significant difference in body fluid Mg concentrations of MS patients compared to healthy controls (Hedges’ g: -0.18, 95% CI: -0.87, 0.50, *P* = 0.598) (Fig 7).

The study pooled was heterogeneous (I^2^ = 97.86, Q = 559.73, *p* = 0.00). No publication bias was observed Based on the Egger test results (z = -2.49, *P* = 0.0129).

To assess the robustness of the pooled results, we conducted a sensitivity analysis by systematically removing each study from the meta-analysis. The findings remained consistent, indicating that no single study disproportionately influenced the pooled effect (Hedges’ g: -0.36, 95% CI: -0.94, 0.59, *P* = 0.33).

## 4. Discussion

MS is recognized as the most prevalent chronic inflammatory demyelinating disease affecting the CNS. The primary pathological features associated with MS are axonal loss and inflammatory plaque; however, the precise etiology of this disease remains unknown [6].

Environmental factors, including changes in essential elements and toxic metals, are thought to play a role in MS pathogenesis [25,30,61]. This systematic review and meta-analysis aimed to explore the relationship between three essential elements (Fe, Zn, and Mg) and three toxic metals (Cd, As, and Pb) concerning MS pathogenicity across various body samples, including whole blood, serum, plasma, and CSF. Our study differs from previous ones by incorporating a broader range of biological specimens, allowing for a more comprehensive assessment of these elements’ concentrations and potential correlations with this disease. In contrast to prior studies, we did not impose temporal restrictions on the inclusion criteria, thus encompassing all relevant studies up to June 27, 2024. This inclusive approach and the expanded sample scope contribute to a more current and exhaustive systematic review and meta-analysis. This meta-analysis revealed statistically equivalent essential elements in blood and CSF levels between healthy populations and MS patients.

Conversely, contrary to our results, another meta-analysis on these essential elements reported elevated levels of Fe and Zn in the blood of patients with MS [20]. This discrepancy may be attributed to the more comprehensive nature of our study and the inclusion of more studies, which led to differing analytical results. Trace elements are essential for human health; even subtle concentration changes can significantly affect health.

Fe, widely recognized as the most important of these elements, is vital for human life. Fe is a cofactor for numerous enzymes, and abnormalities in Fe concentration can lead to neurodegeneration [20]. Multiple studies have demonstrated elevated Fe levels in patients with MS using magnetic resonance imaging (MRI) and other imaging methods, potentially resulting in decreased Fe concentrations in the blood [96–99]. Our findings in this meta-analysis indicated that Fe concentration in CSF and blood was the same in MS patients as in controls. Furthermore, after subgroup assessment, the cause of heterogeneity remained unidentified. It is noteworthy that this essential element appears to be neither affected by nor affecting MS pathogenicity, based on the results of this analysis.

Zn is also recognized as a principal element in human blood. Zn is a cofactor for more than 300 enzymes and a component of myelin basic protein [20]. Our findings indicated that Zn levels were not significantly different between MS patients and controls. In contrast, a meta-analysis conducted by Stojsavljevi’c et al. (2024) [23] demonstrated lower Zn levels in the serum or plasma of MS patients compared to controls. This outcome discrepancy can be attributed to our review’s greater number of studies. However, after performing subgroup analysis using the colorimetric method, the results showed a reduction in Zn levels in biological samples (CSF and blood) of MS patients. These findings highlight the importance of the measurement methods in the research.

Lastly, Mg plays an important role in the nervous system because of its ability to reduce nerve cell excitability [100]. This essential element also activates approximately 320 enzymes. Mg is also known to interact with other elements, such as calcium, zinc, and aluminum, and affect the immune system by increasing the synthesis of tumor necrosis factor and interleukin 6, major inflammation mediators [101]. Our analysis found no significant difference in circulating (CSF and blood) Mg levels between MS patients and controls. In the subgroup analyses, we also did not find the cause of heterogeneity for this important element in the human body.

Regarding toxic metals, we found significantly higher levels of AS and Cd in the blood and CSF of MS patients than in controls. Our findings align with the previous meta-analysis conducted on the As and Cd levels in MS patients and their control group by Sarihi et al. (2021) [24]. However, our results showed that Pb levels were not significantly different, which supports Sarihi’s (2021) findings [24]. As is known to lead to adverse renal, hepatic, and cardiovascular conditions [102]. The data on the nervous system is limited, but some studies have reported that AS exposure can lead to brain injury and neuropathy [16]. Yousefi et al. [16] also found an association between As levels and increased brain oxidative stress, indicating the role of As in MS pathogenesis. Additionally, Yen et al. [103] showed that exposure to inorganic As causes an increase in lipid peroxidation in the cerebral cortex.

Cd, with its extreme health hazards, is also known to increase lipid peroxidation pathways [104]. Despite the unknown mechanism of Cd pathogenicity, it has been reported that this toxic metal can displace Fe and Cu from various proteins and increase Fe and Cu-free levels [26]. Our analysis also reported higher Cd levels in CSF and blood of MS patients. After subgroup analysis, we found that the Cd level assessment method can affect the results. A significant difference (with P<0.000) was found in the AAS method while studying the measured Cd level by the ICP method and found no difference in Cd between MS patients and controls. Cd levels in CSF and plasma were not significantly different in contrast to the studies that reported Cd levels in whole blood and serum of MS patients were higher than in controls. Further studies are suggested to determine the cause of the difference between the two methods of element assessment in body fluids and the difference in plasma and serum concentrations.

Pb and other toxic metals are a major risk to human health [6]. It is reported that Pb can act as a hapten by binding to myelin proteins and is responsible for autoantibody production against myelin proteins [105]. Regarding Pb, no difference was observed in the analysis between MS patients and controls. The AAS method showed a significant difference, indicating higher Pb in MS samples than controls, unlike the ICP method. For body fluid subgroups, all outcomes were neutral.

Another important topic worth noting is that after applying the HDI subgroup to these elements, the results showed that pooled developing countries reported larger confidence intervals for all six elements than developed countries’ narrow confidence intervals. This difference might have been due to the more precise methods, stricter protocols, and more advanced equipment in developed countries than in developing ones, which should be considered.

## 5. Limitations

This systematic review and meta-analysis concluded that several limitations should be considered. The results should be interpreted cautiously, considering the different results with different methods and high heterogeneity. More studies must be performed on this subject on a bigger scale, measuring these elements with ICP and AAS methods to get more reliable results.

## 6. Conclusion

Our study revealed a significant finding regarding the toxic metal concentrations in patients with MS compared to the healthy control group. Specifically, our findings demonstrated elevated levels of As and Cd in MS patients, indicating a potential association between these toxic metals and MS pathogenesis. In contrast, no significant differences were observed in the concentrations of all included essential elements (Zn, Fe, Mg) between the MS and control groups.

These results highlight the need for further research to elucidate the mechanisms through which As and Cd may contribute to MS development. The absence of significant differences in essential elements (Zn, Fe, Mg) and Pb indicates that their role in MS pathology may not be as pronounced or that other factors might influence their levels. Our findings emphasize the importance of considering toxic metal exposure in the context of MS and suggest potential avenues for targeted interventions, identifying susceptible individuals, and developing preventive measures. Moreover, establishing new public health guidelines for toxic metals, particularly As and Cd, is paramount. Considering the prevalence of MS globally and the potential link between this disease and the concentration of elements in the human body, limiting exposure to contaminated environments and maintaining essential element levels through natural resources or supplementation is crucial.

## Supporting information

S1 Checklist PRISMA 2020 checklist. (PDF)

S1 File Risk of bias assessment for each study. (DOCX)

S2 File A list of obtained studies from various databases (n = 5466). (RAR)

S3 File (RTF)

S4 File (XLSX)
