# Supplementary material for: Essential and toxic metal concentrations in biological samples of multiple sclerosis patients: A systematic review and meta-analysis
Source: PLoS One. 2024 Dec 6;19(12):e0313851. doi: 10.1371/journal.pone.0313851 (PMC11623488; doi:10.1371/journal.pone.0313851)
Supplement: S1 File — (DOCX) [file pone.0313851.s002.docx]

**S1 Table**: Risk of bias assessment for each study

| **Study** | **Design** | **Q1** | **Q2** | **Q3** | **Q4** | **Q5** | **Q6** | **Q7** | **Q8** | **Q9** | **Q10** | **Q11** | **Risk level** | **Overall quality** |
| --- | --- | --- | --- | --- | --- | --- | --- | --- | --- | --- | --- | --- | --- | --- |
| Alimonti A (2007)  (29) | Case-control | Y | Y | Y | Y | Y | Y | Y | Y | Y | Y | - | Low | High |
| Aliomrani M (2017)  (30) | Case-control | Y | Y | Y | Y | Y | Y | Y | Y | Y | Y | - | Low | High |
| Bahrampour Juybari K (2018)  (8) | Case-control | Y | Y | Y | Y | Y | Y | Y | Y | Y | Y | - | Low | High |
| Dehghanifiroozabadi M (2019)  (33) | Case-control | Y | Y | Y | Y | Y | Y | Y | Y | Y | Y | - | Low | High |
| Nashmi A (2020)  (55) | Case-control | Y | Y | Y | Y | Y | Y | Y | Y | Y | Y | - | Low | High |
| Forte G (2005)  (7) | Case-control | Y | Y | Y | Y | Y | N | U | Y | Y | Y | - | Low | High |
| Ghoreishi A (2015)  (39) | Case-control | Y | Y | Y | Y | Y | Y | Y | Y | Y | Y | - | Low | High |
| Giacoppo S (2014)  (40) | Case-control | Y | Y | Y | Y | Y | U | U | Y | Y | Y | - | Low | High |
| Janghorbani M (2017)  (44) | Case-control | Y | Y | Y | Y | Y | U | Y | Y | Y | Y | - | Low | High |
| Madeddu R (2011)  (49) | Case-control | Y | Y | Y | Y | Y | Y | Y | Y | Y | Y | - | Low | High |
| Paknejad B (2019)  (58) | Case-control | Y | Y | Y | Y | Y | Y | Y | Y | Y | Y | - | Low | High |
| Visconti A (2005)  (71) | Case-control | Y | Y | Y | Y | Y | Y | Y | Y | Y | Y | - | Low | High |
| Yousefi B (2014)  (16) | Case-control | Y | Y | Y | Y | Y | N | U | Y | Y | Y | - | Low | High |
| Oliveira M (2020)  (56) | Case-control | Y | Y | Y | Y | Y | U | U | Y | Y | Y | - | Low | High |
| Abo-Krysha N (2008)  (79) | Case-control | Y | Y | Y | Y | Y | N | U | Y | Y | Y | - | Low | High |
| Akbay GD (2020)  (26) | Case-control | Y | Y | Y | Y | Y | N | U | Y | Y | Y | - | Low | High |
| Al-Radaideh A (2021)  (27) | Case-control | Y | Y | Y | Y | Y | U | U | Y | Y | Y | - | Low | High |
| Algül S (2021)  (28) | Case-control | Y | Y | Y | Y | Y | U | U | Y | Y | Y | - | Low | High |
| Alizadeh A (2016)  (82) | Case-control | Y | Y | Y | Y | Y | Y | Y | Y | Y | Y | - | Low | High |
| Armon-Omer A (2019)  (31) | Case-control | Y | Y | Y | Y | Y | Y | U | Y | Y | Y | - | Low | High |
| Bergsland N (2017)  (77) | Case-control | Y | Y | Y | Y | Y | U | U | Y | Y | Y | - | Low | High |
| Dore-Duffy P (1983)  (34) | Case-control | Y | Y | Y | Y | Y | N | N | Y | Y | Y | - | Low | High |
| Elberry RA (2020)  (37) | Case-control | Y | Y | Y | Y | Y | N | N | Y | Y | Y | - | Low | High |
| Gellein K (2008)  (39) | Case-control | Y | Y | Y | Y | Y | Y | Y | Y | Y | Y | - | Low | High |
| Elberry RA (2020)  (35) | Case-control | Y | Y | Y | Y | Y | Y | Y | Y | Y | Y | - | Low | High |
| Gellein K (2008)  (37) | Case-control | Y | Y | Y | Y | Y | Y | Y | Y | Y | Y | - | Low | High |
| Nasrabadi M N (2011)  (88) | Case-control | Y | Y | Y | Y | Y | Y | Y | Y | Y | Y | - | Low | High |
| Bsteh G (2019)  (34) | Cross-sectional | Y | Y | Y | Y | Y | Y | Y | Y | - | - | - | Low | High |
| Kurup RK (2002)  (50) | Case-control | Y | Y | Y | Y | Y | Y | Y | Y | Y | Y | - | Low | High |
| Ghazavi A (2012)  (38) | Case-control | Y | Y | Y | Y | Y | Y | Y | Y | Y | Y | - | Low | High |
| Moradi A (2016)  (80) | Case-control | Y | Y | Y | Y | Y | Y | Y | Y | Y | Y | - | Low | High |
| Nasrabadi M N (2011)  (87) | Case-control | Y | Y | Y | Y | Y | N | U | Y | Y | Y | - | Low | High |
| Bsteh G (2019)  (32) | Case-control | Y | Y | Y | Y | Y | U | U | Y | Y | Y | - | Low | High |
| Kurup RK (2002)  (47) | Case-control | Y | Y | Y | Y | Y | Y | Y | Y | Y | Y | - | Low | High |
| Mezzaroba L (2020)  (53) | Case-control | Y | Y | Y | Y | Y | U | U | Y | Y | Y | - | Low | High |
| Taheri M (2015)  (83) | Case-control | Y | Y | Y | Y | Y | Y | Y | Y | Y | Y | - | Low | High |
| Thamer AA (2014)  (68) | Case-control | Y | Y | Y | Y | Y | Y | Y | Y | Y | Y | - | Low | High |
| Harbige L S (2011)  (41) | Case-control | Y | Y | Y | Y | Y | Y | Y | Y | Y | Y | - | Low | High |
| Linke E (1977)  (48) | Cohort | Y | Y | Y | Y | Y | Y | Y | Y | Y | Y | Y | Low | High |
| The Birmingham research unit (1976)  (69) | Case-control | Y | Y | Y | Y | Y | Y | Y | Y | Y | Y | - | Low | High |
| Palm R (1982)  (59) | Case-control | Y | Y | Y | Y | Y | Y | Y | Y | Y | Y | - | Low | High |
| Pawlitzki M (2018)  (60) | Case-control | Y | Y | Y | Y | Y | Y | Y | Y | Y | Y | - | Low | High |
| Rieder H.P (1983)  (61) | Case-control | Y | Y | Y | Y | Y | Y | Y | Y | Y | Y | - | Low | High |
| Ristori G (2011)  (62) | Case-control | Y | Y | Y | Y | Y | Y | Y | Y | Y | Y | - | Low | High |
| Sedighi B (2013)  (84) | Case-control | Y | Y | Y | Y | Y | Y | Y | Y | Y | Y | - | Low | High |
| Sfagos C (2005)  (63) | Case-control | Y | Y | Y | Y | Y | Y | Y | Y | Y | Y | - | Low | High |
| Shaheen H (2006)  (64) | Case-control | Y | Y | Y | Y | Y | Y | Y | Y | Y | Y | - | Low | High |
| Siotto M (2019)  (75) | Case-control | Y | Y | Y | Y | Y | Y | Y | Y | Y | Y | - | Low | High |
| Smith D.K (1989)  (65) | Case-control | Y | Y | Y | Y | Y | Y | Y | Y | Y | Y | - | Low | High |
| Socha K (2017)  (66) | Case-control | Y | Y | Y | Y | Y | Y | Y | Y | Y | Y | - | Low | High |
| Stelmasiak Z (1995)  (67) | Case-control | Y | Y | Y | Y | Y | Y | Y | Y | Y | Y | - | Low | High |
| Moghtaderi A (2015)  (54) | Case-control | Y | Y | Y | Y | Y | Y | Y | Y | Y | Y | - | Low | High |
| Iranmanesh F (2012)  (76) | Case-control | Y | Y | Y | Y | Y | Y | Y | Y | Y | Y | - | Low | High |
| Iranmanesh M (2012)  (43) | Case-control | Y | Y | Y | Y | Y | Y | Y | Y | Y | Y | - | Low | High |
| Karpińska E (2017)  (46) | Case-control | Y | Y | Y | Y | Y | Y | Y | Y | Y | Y | - | Low | High |
| Ellidag H Yaser (2014)  (36) | Case-control | Y | Y | Y | Y | Y | Y | Y | Y | Y | Y | - | Low | High |
| Markovska O (2017)  (50) | Case-control | Y | Y | Y | Y | Y | Y | Y | Y | Y | Y | - | Low | High |
| Masoud S A (2007)  (78) | Case-control | Y | Y | Y | Y | Y | Y | Y | Y | Y | Y | - | Low | High |
| Matar A (2020)  (51) | Case-control | Y | Y | Y | Y | Y | Y | Y | Y | Y | Y | - | Low | High |
| Oraby M I (2019)  (57) | Case-control | Y | Y | Y | Y | Y | Y | Y | Y | Y | Y | - | Low | High |
| Kapaki E (1989)  (45) | Case-control | Y | Y | Y | Y | Y | Y | Y | Y | Y | Y | - | Low | High |
| Mohamed SR  2022  (81) | Cross-sectional | Y | Y | Y | Y | Y | Y | Y | Y | - | - | - | Low | High |
| Heipertz R (1979)  (42) | Case-control | Y | Y | Y | Y | Y | Y | Y | Y | Y | Y | - | Low | High |
| MELØ T M (2002)  (52) | Case-control | Y | Y | Y | Y | Y | Y | Y | Y | Y | Y | - | Low | High |
| van Rensburg S J (2006)  (70) | Case-control | Y | Y | Y | Y | Y | Y | Y | Y | Y | Y | - | Low | High |

**Y** = Yes, **N** = No, **U** = Unclear, **NA** = Not Applicable

**JBI checklist for case-control studies**

Q1. Were the groups comparable other than the presence of disease in cases or the absence of disease in controls?

Q2. Were cases and controls matched appropriately?

Q3. Were the same criteria used for identification of cases and controls?

Q4. Was exposure measured in a standard, valid and reliable way?

Q5. Was exposure measured in the same way for cases and controls?

Q6. Were confounding factors identified?

Q7. Were strategies to deal with confounding factors stated?

Q8. Were outcomes assessed in a standard, valid and reliable way for cases and controls?

Q9. Was the exposure period of interest long enough to meaningful?

Q10.Was appropriate statistical analysis used?

**JBI checklist for cohort study**

Q1. Were the two groups similar and recruited from the same population?

Q2. Were the exposures measured similarly to assign people to both exposed and unexposed groups?

Q3. Was the exposure measured in a valid and reliable way?

Q4. Were confounding factors identified?

Q5. Were strategies to deal with confounding factors stated?

Q6. Were the groups/participants free of the outcome at the start of the study (or at the moment of exposure)?

Q7. Were the outcomes measured in a valid and reliable way?

Q8. Was the follow up time reported and sufficient to be long enough for outcomes to occur?

Q9. Was follow up complete, and if not, were the reasons to loss to follow up described and explored?

Q10.Were strategies to address incomplete follow up utilized?

Q11.Was appropriate statistical analysis used?

**JBI checklist for cross-sectional studies**

Q1. Were the criteria for inclusion in the sample clearly defined?

Q2. Were the study subjects and the setting described in detail?

Q3. Was the exposure measured in a valid and reliable way?

Q4. Were objective, standard criteria used for measurement of the condition?

Q5. Were confounding factors identified?

Q6. Were strategies to deal with confounding factors stated?

Q7. Were the outcomes measured in a valid and reliable way?

Q8. Was appropriate statistical analysis used?
