# Supplementary material for: Essential and toxic metal concentrations in biological samples of multiple sclerosis patients: A systematic review and meta-analysis
Source: PLoS One. 2024 Dec 6;19(12):e0313851. doi: 10.1371/journal.pone.0313851 (PMC11623488; doi:10.1371/journal.pone.0313851)
Supplement: S3 File — (RTF) [file pone.0313851.s004.rtf]

1.	Lead and multiple sclerosis. Journal of the Royal College of General Practitioners. 1976.
2.	Transactions of the American Clinical and Climatological Association. The ninetieth annual meeting. 1978.
3.	[Diagnosis of magnesium deficiency should be encouraged (proceedings)]. MMW Munch Med Wochenschr. 1979;121(12):408.
4.	When your patients ask about mercury in amalgam. J Am Dent Assoc. 1990;120(4):395-8.
5.	Heteroaryl butyric acids and their derivatives as inhibitors of matrix metalloproteinases - Warner-Lambert Company: W09918079. EXPERT OPINION ON THERAPEUTIC PATENTS. 2000;10(1):111-5.
6.	Efficacy of peripheral nerve evaluation versus first stage tined lead in multiple sclerosis patients. EUROPEAN UROLOGY SUPPLEMENTS. 2005;4(3):162-.
7.	IL12A, MPHOSPH9/CDK2AP1 and RGS1 are novel multiple sclerosis susceptibility loci. Genes Immun. 2010;11(5):397-405.
8.	Study could lead to potential new treatment methods for multiple sclerosis. EXPERT REVIEW OF NEUROTHERAPEUTICS. 2011;11(7):921-.
9.	Correction: Multiple sclerosis in the Mount Etna region: Possible role of volcanogenic trace elements (PLoS ONE). PLoS ONE. 2014.
10.	Aarli JA. Role of cytokines in neurological disorders. Curr Med Chem. 2003;10(19):1931-7.
11.	Abad C, Tan YV, Lopez R, Nobuta H, Dong HM, Phan P, et al. Vasoactive intestinal peptide loss leads to impaired CNS parenchymal T-cell infiltration and resistance to experimental autoimmune encephalomyelitis. PROCEEDINGS OF THE NATIONAL ACADEMY OF SCIENCES OF THE UNITED STATES OF AMERICA. 2010;107(45):19555-60.
12.	Abakumova TO, Nukolova NV, Gusev EI, Chekhonin VP. Contrast agents in MRI-diagnosis of multiple sclerosis. Zhurnal Nevrologii i Psihiatrii imeni SS Korsakova. 2015.
13.	Abarca-Zabalía J, García MI, Lozano Ros A, Marín-Jiménez I, Martínez-Ginés ML, López-Cauce B, et al. Differential Expression of SMAD Genes and S1PR1 on Circulating CD4+ T Cells in Multiple Sclerosis and Crohn's Disease. Int J Mol Sci. 2020;21(2).
14.	Abasıyanık Z, Kahraman T, Ertekin Ö, Baba C, Özakbaş S. Prevalence and determinants of falls in persons with multiple sclerosis without a clinical disability. Mult Scler Relat Disord. 2021;49:102771.
15.	Abbaszadeh S, Tabary M, Aryannejad A, Abolhasani R, Araghi F, Khaheshi I, et al. Air pollution and multiple sclerosis: a comprehensive review. NEUROLOGICAL SCIENCES. 2021;42(10):4063-72.
16.	Abdelhak A, Weber MS, Tumani H. Primary progressive multiple sclerosis: Putting together the Puzzle. Frontiers in Neurology. 2017.
17.	Abdollahi F, Sajedi SA, Majdinasab N, Saghatoleslami M, Rad HS, Ghorbani A, et al. Is there an association between brain iron accumulations with blood iron metabolism markers in multiple sclerosis patients? Multiple Sclerosis Journal. 2017;23(1):12-.
18.	Abdollahpour I, Nedjat S, Salimi Y, Mansournia MA, Vahid F, Weinstock-Guttman B. The role of dietary antioxidant index and index of nutritional quality in MS onset: finding from an Iranian population-based incident case-control study. Nutr Neurosci. 2022;25(2):379-86.
19.	Abdolmaleki A, Kondori BJ, Raei M, Ghaleh HEG. Cell therapy procedure using anti-inflammatory macrophage M2 can potentially reduce Clinical Score in animals with Experimental Autoimmune Encephalomyelitis: A preclinical systematic review and meta-analysis study. Fundam Clin Pharmacol. 2023;37(2):215-25.
20.	Abhari AP, Etemadifar M, Yazdanpanah N, Rezaei N. N-Methyl-D-Aspartate (NMDA)-Type Glutamate Receptors and Demyelinating Disorders: A Neuroimmune Perspective. Mini Rev Med Chem. 2022;22(20):2624-40.
21.	Abo-Krysha N, Rashed L. The role of iron dysregulation in the pathogenesis of multiple sclerosis: An Egyptian study. Multiple Sclerosis. 2008.
22.	Abo-Krysha N, Rashed L. The role of iron dysregulation in the pathogenesis of multiple sclerosis: an Egyptian study. MULTIPLE SCLEROSIS JOURNAL. 2008;14(5):602-8.
23.	Abo-Krysha N, Rashed L. The role of iron dysregulation in the pathogenesis of multiple sclerosis: an Egyptian study. MULTIPLE SCLEROSIS JOURNAL. 2008;14(5):602-8.
24.	Abokrysha N. Magnesium sulphate as a treatment of acute attack of multiple sclerosis. Journal of Medical Hypotheses and Ideas. 2010;4:5.
25.	Abramson E, Hardman C, Shimizu AJ, Hwang S, Hester LD, Snyder SH, et al. Designed PKC-targeting bryostatin analogs modulate innate immunity and neuroinflammation. Cell Chem Biol. 2021;28(4):537-45.e4.
26.	Absinta M, Sati P, Schindler M, Leibovitch EC, Ohayon J, Wu TX, et al. Persistent 7-tesla phase rim predicts poor outcome in new multiple sclerosis patient lesions. JOURNAL OF CLINICAL INVESTIGATION. 2016;126(7):2597-609.
27.	Acevedo AR, Nava C, Arriada N, Violante A, Corona T. Cardiovascular dysfunction in multiple sclerosis. Acta Neurol Scand. 2000;101(2):85-8.
28.	Achiron A, Gurevich M, Snir Y, Segal E, Mandel M. Zinc-ion binding and cytokine activity regulation pathways predicts outcome in relapsing–remitting multiple sclerosis. Clinical & Experimental Immunology. 2007;149(2):235-42.
29.	Acosta-Ampudia Y, Monsalve DM, Ramírez-Santana C. Identifying the culprits in neurological autoimmune diseases. J Transl Autoimmun. 2019;2:100015.
30.	Actis GC, Rosina F. Inflammatory bowel disease: An archetype disorder of outer environment sensor systems. World J Gastrointest Pharmacol Ther. 2013;4(3):41-6.
31.	Adam O. Nutrition and immun system: Rheumatism. Aktuelle Ernahrungsmedizin. 2002.
32.	Adam P, Sobek O, Scott CS, Dolezil D, Kasik J, Hajdukova L, et al. Immunophenotypic analysis of cerebrospinal fluid cell populations with the Cell-Dyn Sapphire haematology analyser: method feasibility and preliminary observations. Int J Lab Hematol. 2010;32(1 Pt 2):22-32.
33.	Adam P, Sobeka O, Scott CS. Analysis of cerebrospinal fluid cell populations with monoclonal antibodies. Folia Microbiol (Praha). 2007;52(5):529-34.
34.	Adamczyk B, Adamczyk-Sowa M. New Insights into the Role of Oxidative Stress Mechanisms in the Pathophysiology and Treatment of Multiple Sclerosis. OXIDATIVE MEDICINE AND CELLULAR LONGEVITY. 2016;2016.
35.	Adamczyk B, Adamczyk-Sowa M. New Insights into the Role of Oxidative Stress Mechanisms in the Pathophysiology and Treatment of Multiple Sclerosis. Oxidative Medicine and Cellular Longevity. 2016.
36.	Adamczyk B, Adamczyk-Sowa M. New Insights into the Role of Oxidative Stress Mechanisms in the Pathophysiology and Treatment of Multiple Sclerosis. Oxid Med Cell Longev. 2016;2016:1973834.
37.	Adamczyk B, Niedziela N, Adamczyk-Sowa M. Novel Approaches of Oxidative Stress Mechanisms in the Multiple Sclerosis Pathophysiology and Therapy2017. 155-71 p.
38.	Adamczyk B, Niedziela N, Adamczyk-Sowa M. Novel Approaches of Oxidative Stress Mechanisms in the Multiple Sclerosis Pathophysiology and Therapy. In: Zagon IS, McLaughlin PJ, editors. Multiple Sclerosis: Perspectives in Treatment and Pathogenesis. Brisbane (AU): Codon Publications
Copyright: The Authors.; 2017.
39.	Adamczyk-Sowa M, Sowa P, Mucha S, Zostawa J, Mazur B, Owczarek M, et al. Changes in Serum Ceruloplasmin Levels Based on Immunomodulatory Treatments and Melatonin Supplementation in Multiple Sclerosis Patients. MEDICAL SCIENCE MONITOR. 2016;22:2484-91.
40.	Adamczyk-Sowa M, Sowa P, Pierzchala K, Polaniak R, Labuz-Roszak B. Antioxidative enzymes activity and malondialdehyde concentration during mitoxantrone therapy in multiple sclerosis patients. J Physiol Pharmacol. 2012;63(6):683-90.
41.	Adams CW. Perivascular iron deposition and other vascular damage in multiple sclerosis. J Neurol Neurosurg Psychiatry. 1988;51(2):260-5.
42.	Adams CW. Perivascular iron deposition and other vascular damage in multiple sclerosis. Journal of Neurology, Neurosurgery & Psychiatry. 1988;51(2):260-5.
43.	Adams CWM. PERIVASCULAR IRON DEPOSITION AND OTHER VASCULAR DAMAGE IN MULTIPLE-SCLEROSIS. JOURNAL OF NEUROLOGY NEUROSURGERY AND PSYCHIATRY. 1988;51(2):260-5.
44.	Adebiyi OE, Bynoe MS. Roles of Adenosine Receptor (subtypes A(1) and A(2A)) in Cuprizone-Induced Hippocampal Demyelination. Mol Neurobiol. 2023;60(10):5878-90.
45.	Adlam JP. Multiple sclerosis: A diathesis? Gazzetta Sanitaria. 1973.
46.	Afsharzadeh N, Lavi Arab F, Sankian M, Samiei L, Tabasi NS, Afsharzadeh D, et al. Comparative assessment of proliferation and immunomodulatory potential of Hypericum perforatum plant and callus extracts on mesenchymal stem cells derived adipose tissue from multiple sclerosis patients. Inflammopharmacology. 2021;29(5):1399-412.
47.	Afsheen N, Rafique S, Rafeeq H, Irshad K, Hussain A, Huma Z, et al. Neurotoxic effects of environmental contaminants-measurements, mechanistic insight, and environmental relevance. Environ Sci Pollut Res Int. 2022;29(47):70808-21.
48.	Aghaie T, Jazayeri MH, Avan A, Anissian A, Salari AA. Gold nanoparticles and polyethylene glycol alleviate clinical symptoms and alter cytokine secretion in a mouse model of experimental autoimmune encephalomyelitis. IUBMB Life. 2019;71(9):1313-21.
49.	Aharoni R, Eilam R, Arnon R. Astrocytes in Multiple Sclerosis-Essential Constituents with Diverse Multifaceted Functions. Int J Mol Sci. 2021;22(11).
50.	Ahmad SH, Agrawal CS, Sud S, Mahajan M. Iron quantification in deep subcortical nuclei and its correlation with extracranial venous system in multiple sclerosis and controls. International Journal of Current Research and Review. 2020.
51.	Ahmad SI. Reactive oxygen species in biology and human health2017.
52.	Aiello I, Pastorino M, Sotgiu S, Pirastru MI, Sau GF, Sanna G, et al. Epidemiology of myasthenia gravis in northwestern Sardinia. Neuroepidemiology. 1997;16(4):199-206.
53.	Airas L, Sucksdorff M, Tuisku J, Rissanen E. Natalizumab Treatment of Multiple Sclerosis Leads to Diminished Microglial Activation in the Normal Appearing White Matter. NEUROLOGY. 2018;90.
54.	Aita JF, Snyder DH, Reichl W. Myasthenia gravis and multiple sclerosis: an unusual combination of diseases. Neurology. 1974;24(1):72-5.
55.	Akbay G, Karakullukçu E, Mutlu A. A Controlled Descriptive Study to Determine the Nutritional Status and Biochemical Parameters of Multiple Sclerosis Patients. Neurology India. 2020.
56.	Akbay GD, Karakullukcu E, Mutlu AA. A Controlled Descriptive Study to Determine the Nutritional Status and Biochemical Parameters of Multiple Sclerosis Patients. NEUROLOGY INDIA. 2020;68(4):867-74.
57.	Akbulut G, Orhan G, Gurkas E, Sanlier N, Gezmen-Karadag M, Karadag YS, et al. Determination of nutritional status via Food Frequency Consumption (FFQ) and serum proteins and anemia parameters in Multiple Sclerosis (MS) patients. Gazi Medical Journal. 2014.
58.	Akçin Ş, Gürsoy AE, Selek Ş, ÇİMen YA, Köktaşoğlu F, Ustunova S, et al. Oxidative Stress, Serum Mineral and Trace Element Levels in Patients with Multiple Sclerosis with or without Restless Legs Syndrome. Eastern Journal Of Medicine. 2024;29(1):129-37.
59.	Al-Araji A, Mohammed AI. Multiple sclerosis in Iraq: does it have the same features encountered in Western countries? J Neurol Sci. 2005;234(1-2):67-71.
60.	Al-Ebraheem A, Miller L, Geraki K, Desmond KL, Stanisz GJ, Bock NA, et al. Transition Metal Distribution in the Brain and Spinal Cord of a Dysmyelinated Rodent Model.  ICXOM23: INTERNATIONAL CONFERENCE ON X-RAY OPTICS AND MICROANALYSIS2016.
61.	Al-Ebraheem A, Miller L, Geraki K, Desmond KL, Stanisz GJ, Bock NA, et al. Transition Metal Distribution in the Brain and Spinal Cord of a Dysmyelinated Rodent Model.  ICXOM23: INTERNATIONAL CONFERENCE ON X-RAY OPTICS AND MICROANALYSIS2016.
62.	Al-Hussain F, Alfallaj MM, Alahmari AN, Almazyad AN, Alsaeed TK, Abdurrahman AA, et al. Relationship between Neutrophil-to-Lymphocyte Ratio and Stress in Multiple Sclerosis Patients. J Clin Diagn Res. 2017;11(5):Cc01-cc4.
63.	Al-Hussain F, Alfallaj MM, Alahmari AN, Almazyad AN, Alsaeed TK, Abdurrahman AA, et al. Relationship between Neutrophilto-Lymphocyte Ratio and Stress in Multiple Sclerosis Patients. JOURNAL OF CLINICAL AND DIAGNOSTIC RESEARCH. 2017;11(5).
64.	Al-Kafaji G, Bakheit HF, AlAli F, Fattah M, Alhajeri S, Alharbi MA, et al. Next-generation sequencing of the whole mitochondrial genome identifies functionally deleterious mutations in patients with multiple sclerosis. PLoS One. 2022;17(2):e0263606.
65.	Al-Namaeh M. Systematic review and meta-analysis of the development of multiple sclerosis in clinically isolated syndrome. Eur J Ophthalmol. 2021;31(4):1643-55.
66.	Al-Radaideh A, Athamneh I, Alabadi H, Hbahbih M. Cortical and Subcortical Morphometric and Iron Changes in Relapsing-Remitting Multiple Sclerosis and Their Association with White Matter T2Lesion Load: A3-Tesla Magnetic Resonance Imaging Study. CLINICAL NEURORADIOLOGY. 2019;29(1):51-64.
67.	Al-Radaideh A, Athamneh I, Alabadi H, Hbahbih M. Cortical and subcortical morphometric and iron changes in relapsing-remitting multiple sclerosis and their association with white matter T2 lesion load. Clinical Neuroradiology. 2019;29(1):51-64.
68.	Al-Radaideh A, Athamneh I, Alabadi H, Hbahbih M. Deep gray matter changes in relapsing-remitting multiple sclerosis detected by multi-parametric, high-resolution magnetic resonance imaging (MRI). Eur Radiol. 2021;31(2):706-15.
69.	Al-Radaideh A, Athamneh I, Alabadi H, Hbahbih M. Deep gray matter changes in relapsing-remitting multiple sclerosis detected by multi-parametric, high-resolution magnetic resonance imaging (MRI). EUROPEAN RADIOLOGY. 2021;31(2):706-15.
70.	Al-Radaideh A, El-Haj N, Hijjawi N. Iron deposition and atrophy in cerebral grey matter and their possible association with serum iron in relapsing-remitting multiple sclerosis. CLINICAL IMAGING. 2021;69:238-42.
71.	Al-Radaideh A, El-Haj N, Hijjawi N. Iron deposition and atrophy in cerebral grey matter and their possible association with serum iron in relapsing-remitting multiple sclerosis. Clinical Imaging. 2021;69:238-42.
72.	Al-Radaideh AM, Wharton SJ, Lim SY, Tench CR, Morgan PS, Bowtell RW, et al. Increased iron accumulation occurs in the earliest stages of demyelinating disease: an ultra-high field susceptibility mapping study in Clinically Isolated Syndrome. MULTIPLE SCLEROSIS JOURNAL. 2013;19(7):896-903.
73.	Al-Zubaidi MA. The Effect of Interferon Beta-1b and Methylprednisolone Treatment on the Serum Trace Elements in Iraqi Patients with Multiple Sclerosis. Journal of Clinical & Diagnostic Research. 2012;6(6).
74.	Alanazy MH, Alomar MA, Aljafen BN, Muayqil TA. Multiple sclerosis and myasthenia gravis following severe weight loss. Neurosciences (Riyadh). 2018;23(2):158-61.
75.	Alanazy MH, Asiri A, Edrees MF, Abuzinadah AR. Impact of neurological diseases on family planning: A single-center experience. Medicine (Baltimore). 2020;99(44):e22978.
76.	Albouz-Abo S, Wilson JC, Bernard CC, von Itzstein M. A conformational study of the human and rat encephalitogenic myelin oligodendrocyte glycoprotein peptides 35-55. Eur J Biochem. 1997;246(1):59-70.
77.	Albrecht DS, Granziera C, Hooker JM, Loggia ML. In Vivo Imaging of Human Neuroinflammation. ACS Chem Neurosci. 2016;7(4):470-83.
78.	Alcover-Sanchez B, Garcia-Martin G, Escudero-Ramirez J, Gonzalez-Riano C, Lorenzo P, Gimenez-Cassina A, et al. Absence of R-Ras1 and R-Ras2 causes mitochondrial alterations that trigger axonal degeneration in a hypomyelinating disease model. Glia. 2021;69(3):619-37.
79.	Alexander CS, editor ANTECEDENTS IN THE WORKPLACE WHICH LEAD TO THE EXACERBATORY FACTORS OF CHRONIC FATIGUE, UNCERTAINTY ABOUT THE FUTURE AND DEPRESSION IN INDIVIDUALS WITH MULTIPLE SCLEROSIS2004: Jordan Whitney Enterprises, Inc.
80.	Alexander JS, Chervenak R, Weinstock-Guainan B, Tsunoda I, Ramanathan M, Martinez N, et al. Blood circulating microparticle species in relapsing-remitting and secondary progressive multiple sclerosis. A case-control, cross sectional study with conventional MRI and advanced iron content imaging outcomes. JOURNAL OF THE NEUROLOGICAL SCIENCES. 2015;355(1-2):84-9.
81.	Alexander JS, Chervenak R, Weinstock-Guttman B, Tsunoda I, Ramanathan M, Martinez N, et al. Blood circulating microparticle species in relapsing-remitting and secondary progressive multiple sclerosis. A case-control, cross sectional study with conventional MRI and advanced iron content imaging outcomes. Journal of the Neurological Sciences. 2015.
82.	Alexander JS, Chervenak R, Weinstock-Guttman B, Tsunoda I, Ramanathan M, Martinez N, et al. Blood circulating microparticle species in relapsing-remitting and secondary progressive multiple sclerosis. A case-control, cross sectional study with conventional MRI and advanced iron content imaging outcomes. J Neurol Sci. 2015;355(1-2):84-9.
83.	Alghamdi SS, Mustafa SM, Moore Ii BM. Synthesis and biological evaluation of a ring analogs of the selective CB2 inverse agonist SMM-189. Bioorg Med Chem. 2021;33:116035.
84.	Algül S, Kapan O, Bengü AŞ. Investigation of some trace element levels in multiple sclerosis. Kastamonu Medical Journal. 2021;1(1):1-4.
85.	Alimonti A, Ristori G, Giubilei F, Stazi MA, Pino A, Visconti A, et al. Serum chemical elements and oxidative status in Alzheimer's disease, Parkinson disease and multiple sclerosis. Neurotoxicology. 2007;28(3):450-6.
86.	Aliomrani M, Sahraian MA, Shirkhanloo H, Sharifzadeh M, Khoshayand MR, Ghahremani MH. Blood concentrations of cadmium and lead in multiple sclerosis patients from Iran. Iranian journal of pharmaceutical research: IJPR. 2016;15(4):825.
87.	Aliomrani M, Sahraian MA, Shirkhanloo H, Sharifzadeh M, Khoshayand MR, Ghahremani MH. Correlation between heavy metal exposure and GSTM1 polymorphism in Iranian multiple sclerosis patients. NEUROLOGICAL SCIENCES. 2017;38(7):1271-8.
88.	Aliomrani M, Sahraian MA, Shirkhanloo H, Sharifzadeh M, Khoshayand MR, Ghahremani MH. Correlation between heavy metal exposure and GSTM1 polymorphism in Iranian multiple sclerosis patients. Neurological Sciences. 2017;38(7):1271-8.
89.	Alizadeh A, Mehrpour O, Nikkhah K, Bayat G, Espandani M, Golzari A, et al. Comparison of serum Concentration of Se, Pb, Mg, Cu, Zn, between MS patients and healthy controls. Electron Physician. 2016;8(8):2759-64.
90.	Alizadeh-Ghodsi M, Zavvari A, Ebrahimi-Kalan A, Shiri-Shahsavar MR, Yousefi B. The hypothetical roles of arsenic in multiple sclerosis by induction of inflammation and aggregation of tau protein: A commentary. Nutritional Neuroscience. 2018;21(2):92-6.
91.	Alnahdi MA, Aljarba SI, Al Malik YM. Alemtuzumab-induced simultaneous onset of autoimmune haemolytic anaemia, alveolar haemorrhage, nephropathy, and stroke: A case report. Mult Scler Relat Disord. 2020;41:102141.
92.	Aloisi F, Cross AH. MINI-review of Epstein-Barr virus involvement in multiple sclerosis etiology and pathogenesis. J Neuroimmunol. 2022;371:577935.
93.	Alonso-Moreno M, Ladrón-Guevara M, Ciudad-Gutiérrez P. Systematic review of gender bias in clinical trials of monoclonal antibodies for the treatment of multiple sclerosis. Neurologia (Engl Ed). 2021.
94.	Alqahtani S, Promtong P, Oliver AW, He XT, Walker TD, Povey A, et al. Silver nanoparticles exhibit size-dependent differential toxicity and induce expression of syncytin-1 in FA-AML1 and MOLT-4 leukaemia cell lines. Mutagenesis. 2016;31(6):695-702.
95.	Alshehri A, Al-Iedani O, Koussis N, Khormi I, Lea R, Lechner-Scott J, et al. Stability of longitudinal DTI metrics in MS with treatment of injectables, fingolimod and dimethyl fumarate. Neuroradiol J. 2023;36(4):388-96.
96.	Altinoz MA, Ozcan EM, Ince B, Guloksuz S. Hemoglobins as new players in multiple sclerosis: metabolic and immune aspects. Metab Brain Dis. 2016;31(5):983-92.
97.	Altokhis AI, Alotaibi AM, Felmban GA, Constantinescu CS, Evangelou N. Iron rims as an imaging biomarker in ms: A systematic mapping review. Diagnostics. 2020.
98.	AlTokhis AI, AlOtaibi AM, Felmban GA, Constantinescu CS, Evangelou N. Iron Rims as an Imaging Biomarker in MS: A Systematic Mapping Review. Diagnostics (Basel). 2020;10(11).
99.	Altokhis AI, Hibbert AM, Allen CM, Mougin O, Alotaibi A, Lim SY, et al. Longitudinal clinical study of patients with iron rim lesions in multiple sclerosis. Mult Scler. 2022;28(14):2202-11.
100.	Altura BT, Bertschat F, Jeremias A, Ising H, Altura BM. Comparative findings on serum IMg2+ of normal and diseased human subjects with the NOVA and KONE ISE's for Mg2+. Scand J Clin Lab Invest Suppl. 1994;217:77-81.
101.	Alvarez-Sanchez N, Dunn SE. Potential biological contributers to the sex difference in multiple sclerosis progression. Front Immunol. 2023;14:1175874.
102.	Amato MP, Zipoli V, Portaccio E. Cognitive changes in multiple sclerosis. Expert Rev Neurother. 2008;8(10):1585-96.
103.	Amatullah H, Fraschilla I, Digumarthi S, Huang J, Adiliaghdam F, Bonilla G, et al. Epigenetic reader SP140 loss of function drives Crohn's disease due to uncontrolled macrophage topoisomerases. Cell. 2022;185(17):3232-47.e18.
104.	Ambler J. Isoelectric focusing on cellulose acetate membranes: an up-date on materials and methods. Electrophoresis. 1989;10(7):520-3.
105.	Amer KA, Aldosari AA, Somaily MY, Shawkhan RA, Almuhsini RA, Al Mater MA, et al. The Assessment of the Prevalence and Disability Severity of Musculoskeletal Pain in Patients With Multiple Sclerosis in Saudi Arabia. Cureus. 2022;14(12):e32413.
106.	Amin B, Maurer A, Voelter W, Melms A, Kalbacher H. New potential serum biomarkers in multiple sclerosis identified by proteomic strategies. Current Medicinal Chemistry. 2014.
107.	Aminzadeh KK, Etminan M. Dental amalgam and multiple sclerosis: a systematic review and meta-analysis. J Public Health Dent. 2007;67(1):64-6.
108.	Amirifard H, Sanei Sistani S, Nezamdoust Z, Haratirad E, Banaie S, Khosravi A. Evaluation of the relationship between retinal nerve layer thickness and corpus callosum atrophy in MRI with memory impairment in patients with multiple sclerosis. J Family Med Prim Care. 2022;11(6):3174-7.
109.	Ammatuna E, Montefusco E, Pacilli M, Divona M, Ardiri D, Centonze D, et al. Use of arsenic trioxide in secondary acute promyelocytic leukemia developing after treatment of multiple sclerosis with mitoxantrone. Leukemia & lymphoma. 2009;50(7):1217-8.
110.	Ammatuna E, Montesinos P, Hasan SK, Ramadan SM, Esteve J, Hubmann M, et al. Presenting features and treatment outcome of acute promyelocytic leukemia arising after multiple sclerosis. HAEMATOLOGICA-THE HEMATOLOGY JOURNAL. 2011;96(4):621-5.
111.	Amoriello R, Chernigovskaya M, Greiff V, Carnasciali A, Massacesi L, Barilaro A, et al. TCR repertoire diversity in Multiple Sclerosis: High-dimensional bioinformatics analysis of sequences from brain, cerebrospinal fluid and peripheral blood. EBIOMEDICINE. 2021;68.
112.	An K, Xue MJ, Zhong JY, Yu SN, Lan TS, Qi ZQ, et al. Arsenic trioxide ameliorates experimental autoimmune encephalomyelitis in C57BL/6 mice by inducing CD4(+) T cell apoptosis. J Neuroinflammation. 2020;17(1):147.
113.	An K, Xue MJ, Zhong JY, Yu SN, Lan TS, Qi ZQ, et al. Arsenic trioxide ameliorates experimental autoimmune encephalomyelitis in C57BL/6 mice by inducing CD4(+) T cell apoptosis. JOURNAL OF NEUROINFLAMMATION. 2020;17(1).
114.	Anderson SA, Shukaliak-Quandt J, Jordan EK, Arbab AS, Martin R, McFarland H, et al. Magnetic resonance imaging of labeled T-Cells in a mouse model of multiple sclerosis. ANNALS OF NEUROLOGY. 2004;55(5):654-9.
115.	Andreu-Caravaca L, Ramos-Campo DJ, Chung LH, Manonelles P, Abellán-Aynés O, Rubio-Arias J. Impact of Lockdown during COVID-19 Pandemic on Central Activation, Muscle Activity, Contractile Function, and Spasticity in People with Multiple Sclerosis. Biomed Res Int. 2021;2021:2624860.
116.	Andrusishina IN. DIAGNOSTIC VALUES OF CALCIUM AND MAGNESIUM FORMS DETERMINED IN HUMAN SERUM AND SALIVA. JOURNAL OF ELEMENTOLOGY. 2010;15(3):425-33.
117.	Andrusishina IN. Diagnostic values of calcium and magnesium forms determined in human serum and saliva. Journal of Elementology. 2010.
118.	Andrusishina IN. DIAGNOSTIC VALUES OF CALCIUM AND MAGNESIUM FORMS DETERMINED IN HUMAN SERUM AND SALIVA. JOURNAL OF ELEMENTOLOGY. 2010;15(3):425-33.
119.	Angelov DN, Waibel S, Guntinas-Lichius O, Lenzen M, Neiss WF, Tomov TL, et al. Therapeutic vaccine for acute and chronic motor neuron diseases: implications for amyotrophic lateral sclerosis. Proc Natl Acad Sci U S A. 2003;100(8):4790-5.
120.	Anglen J, Gruninger SE, Chou HN, Weuve J, Turyk ME, Freels S, et al. Occupational mercury exposure in association with prevalence of multiple sclerosis and tremor among US dentists. J Am Dent Assoc. 2015;146(9):659-68.e1.
121.	Annaházi A, Schemann M. Contribution of the Enteric Nervous System to Autoimmune Diseases and Irritable Bowel Syndrome. Adv Exp Med Biol. 2022;1383:1-8.
122.	Anthony DC, Sibson NR, McAteer MA, Davis B, Choudhury RP. Detection of brain pathology by magnetic resonance imaging of iron oxide micro-particles. Methods in molecular biology (Clifton, NJ). 2011.
123.	Anthony DC, Sibson NR, McAteer MA, Davis B, Choudhury RP. Detection of brain pathology by magnetic resonance imaging of iron oxide micro-particles. Methods Mol Biol. 2011;686:213-27.
124.	Antonen J, Syrjälä P, Oikarinen R, Frey H, Krohn K. Acute multiple sclerosis exacerbations are characterized by low cerebrospinal fluid suppressor/cytotoxic T-cells. Acta Neurol Scand. 1987;75(2):156-60.
125.	Antunes JL, Schlesinger EB, Michelsen WJ. The abnormal brain scan in demyelinating diseases. Arch Neurol. 1974;30(3):269-71.
126.	Anyachor CP, Dooka DB, Orish CN, Amadi CN, Bocca B, Ruggieri F, et al. Mechanistic considerations and biomarkers level in nickel-induced neurodegenerative diseases: An updated systematic review. IBRO Neurosci Rep. 2022;13:136-46.
127.	Aoun R, Gratch D, Kaminetzky D, Kister I. Immune Checkpoint Inhibitors in Patients with Pre-existing Neurologic Autoimmune Disorders. Curr Neurol Neurosci Rep. 2023;23(11):735-50.
128.	Aparicio SR, Marsden P. Application of standard micro-anatomical staining methods to epoxy resin-embedded sections. J Clin Pathol. 1969;22(5):589-92.
129.	Apseloff G. Therapeutic uses of gallium nitrate: past, present, and future. Am J Ther. 1999;6(6):327-39.
130.	Araki S, Uchino M, Kumamoto T. Prevalence studies of multiple sclerosis, myasthenia gravis, and myopathies in Kumamoto district, Japan. Neuroepidemiology. 1987;6(3):120-9.
131.	Aranapakam V, Grosu GT, Davis JM, Hu B, Ellingboe J, Baker JL, et al. Synthesis and structure - Activity relationship of α-sulfonylhydroxamic acids as novel, orally active matrix metalloproteinase inhibitors for the treatment of osteoarthritis. Journal of Medicinal Chemistry. 2003.
132.	Aranapakam V, Grosu GT, Davis JM, Hu B, Ellingboe J, Baker JL, et al. Synthesis and structure-activity relationship of alpha-sulfonylhydroxamic acids as novel, orally active matrix metalloproteinase inhibitors for the treatment of osteoarthritis. J Med Chem. 2003;46(12):2361-75.
133.	Arasa F. [Etiopathogenesis of multiple sclerosis]. Folia Clin Int (Barc). 1968;18(12):668-85.
134.	Arbuzova EE, Selyanina NV, Krivtsov AV. Influence of single nucleotide polymorphisms of “zinc fingers” proteins genes on multiple sclerosis. Russian Neurological Journal. 2019.
135.	Armon-Omer A, Mansor T, Edelstein M, Bukovetzky E, Groisman L, Rorman E, et al. Association between multiple sclerosis and urinary levels of toxic metals and organophosphates: A cross-sectional study in Israel. Mult Scler Relat Disord. 2024;83:105445.
136.	Armon-Omer A, Waldman C, Simaan N, Neuman H, Tamir S, Shahien R. New Insights on the Nutrition Status and Antioxidant Capacity in Multiple Sclerosis Patients. NUTRIENTS. 2019;11(2).
137.	Armon-Omer A, Waldman C, Simaan N, Neuman H, Tamir S, Shahien R. New insights on the nutrition status and antioxidant capacity in multiple sclerosis patients. Nutrients. 2019.
138.	Armon-Omer A, Waldman C, Simaan N, Neuman H, Tamir S, Shahien R. New Insights on the Nutrition Status and Antioxidant Capacity in Multiple Sclerosis Patients. Nutrients. 2019;11(2).
139.	Armon-Omer A, Waldman C, Simaan N, Neuman H, Tamir S, Shahien R. New Insights on the Nutrition Status and Antioxidant Capacity in Multiple Sclerosis Patients. NUTRIENTS. 2019;11(2).
140.	Armstrong MA, Shah S, Hawkins SA, Bell AL, Roberts SD. Reduction of monocyte 5'nucleotidase activity by gamma-interferon in multiple sclerosis and autoimmune diseases. Ann Neurol. 1988;24(1):12-6.
141.	Arruda LC, Clave E, Moins-Teisserenc H, Douay C, Farge D, Toubert A. Resetting the immune response after autologous hematopoietic stem cell transplantation for autoimmune diseases. Curr Res Transl Med. 2016;64(2):107-13.
142.	Asche CV, Singer ME, Jhaveri M, Chung H, Miller A. All-cause health care utilization and costs associated with newly diagnosed multiple sclerosis in the United States. Journal of Managed Care Pharmacy. 2010.
143.	Asgari R, Yarani R, Mohammadi P, Emami Aleagha MS. HIF-1α in the Crosstalk Between Reactive Oxygen Species and Autophagy Process: A Review in Multiple Sclerosis. Cell Mol Neurobiol. 2022;42(7):2121-9.
144.	Ashkarran AA, Gharibi H, Zeki DA, Radu I, Khalighinejad F, Keyhanian K, et al. Multi-omics analysis of magnetically levitated plasma biomolecules. Biosens Bioelectron. 2023;220:114862.
145.	Ashtari F, Esmaeil N, Mansourian M, Poursafa P, Mirmosayyeb O, Barzegar M, et al. An 8-year study of people with multiple sclerosis in Isfahan, Iran: Association between environmental air pollutants and severity of disease. Journal of Neuroimmunology. 2018.
146.	Askey-Jones S, Silber E, David A, Chalder T, Shaw P, Gray R. From efficacy to effectiveness: does a nurse-led cognitive behavior therapy intervention work in patients with multiple sclerosis in the real-life clinical setting? MULTIPLE SCLEROSIS JOURNAL. 2008;14:S137-S.
147.	Askey-Jones S, Silber E, Shaw P, Gray R, David AS. A nurse-led mental health service for people with multiple sclerosis. JOURNAL OF PSYCHOSOMATIC RESEARCH. 2012;72(6):463-5.
148.	Askey-Jones S, Silber E, Shaw P, Gray R, David AS. A nurse-led mental health service for people with multiple sclerosis. JOURNAL OF PSYCHOSOMATIC RESEARCH. 2012;72(6):463-5.
149.	Askey-Jones S, Silber E, Shaw P, Gray R, David AS. A nurse-led mental health service for people with multiple sclerosis. JOURNAL OF PSYCHOSOMATIC RESEARCH. 2012;72(6):463-5.
150.	Aslam N, Khan IU, Bashamakh A, Alghool FA, Aboulnour M, Alsuwayan NM, et al. Multiple Sclerosis Diagnosis Using Machine Learning and Deep Learning: Challenges and Opportunities. Sensors (Basel). 2022;22(20).
151.	Aslani M, Mortazavi-Jahromi SS, Mirshafiey A. Efficient roles of miR-146a in cellular and molecular mechanisms of neuroinflammatory disorders: An effectual review in neuroimmunology. Immunol Lett. 2021;238:1-20.
152.	Asmarian N, Sharafi Z, Mousavi A, Jacques R, Tamayo I, Bind MA, et al. Multiple sclerosis incidence rate in southern Iran: a Bayesian epidemiological study. BMC Neurol. 2021;21(1):309.
153.	Aspli KT, Flaten TP, Roos PM, Holmoy T, Skogholt JH, Aaseth J. Iron and copper in progressive demyelination - New lessons from Skogholt's disease. JOURNAL OF TRACE ELEMENTS IN MEDICINE AND BIOLOGY. 2015;31:183-7.
154.	Aspli KT, Flaten TP, Roos PM, Holmøy T, Skogholt JH, Aaseth J. Iron and copper in progressive demyelination - New lessons from Skogholt's disease. Journal of Trace Elements in Medicine and Biology. 2015.
155.	Aspli KT, Flaten TP, Roos PM, Holmøy T, Skogholt JH, Aaseth J. Iron and copper in progressive demyelination--New lessons from Skogholt's disease. J Trace Elem Med Biol. 2015;31:183-7.
156.	Aspli KT, Flaten TP, Roos PM, Holmoy T, Skogholt JH, Aaseth J. Iron and copper in progressive demyelination - New lessons from Skogholt's disease. JOURNAL OF TRACE ELEMENTS IN MEDICINE AND BIOLOGY. 2015;31:183-7.
157.	Åström ME, Roos PM. Geochemistry of multiple sclerosis in Finland. Sci Total Environ. 2022;841:156672.
158.	Atabati H, Yazdanpanah E, Mortazavi H, Bajestani SG, Raoofi A, Esmaeili SA, et al. Immunoregulatory Effects of Tolerogenic Probiotics in Multiple Sclerosis. Adv Exp Med Biol. 2021;1286:87-105.
159.	Attar AM, Kharkhaneh A, Etemadifar M, Keyhanian K, Davoudi V, Saadatnia M. Serum Mercury Level and Multiple Sclerosis. BIOLOGICAL TRACE ELEMENT RESEARCH. 2012;146(2):150-3.
160.	Attar AM, Kharkhaneh A, Etemadifar M, Keyhanian K, Davoudi V, Saadatnia M. Serum mercury level and multiple sclerosis. Biological Trace Element Research. 2012.
161.	Attar AM, Kharkhaneh A, Etemadifar M, Keyhanian K, Davoudi V, Saadatnia M. Serum mercury level and multiple sclerosis. Biol Trace Elem Res. 2012;146(2):150-3.
162.	Attar AM, Kharkhaneh A, Etemadifar M, Keyhanian K, Davoudi V, Saadatnia M. Serum Mercury Level and Multiple Sclerosis. BIOLOGICAL TRACE ELEMENT RESEARCH. 2012;146(2):150-3.
163.	Au M, Mitrev N, Leong RW, Kariyawasam V. Dual biologic therapy with ocrelizumab for multiple sclerosis and vedolizumab for Crohn's disease: A case report and review of literature. World J Clin Cases. 2022;10(8):2569-76.
164.	Aubert-Broche B, Fonov V, Narayanan S, Arnold DL, Araujo D, Fetco D, et al. Onset of multiple sclerosis before adulthood leads to failure of age-expected brain growth. NEUROLOGY. 2014;83(23):2140-6.
165.	Aubert-Broche B, Fonov V, Narayanan S, Arnold DL, Araujo D, Fetco D, et al. Onset of multiple sclerosis before adulthood leads to failure of age-expected brain growth. NEUROLOGY. 2014;83(23):2140-6.
166.	Aubert-Broche B, Fonov V, Narayanan S, Arnold DL, Araujo D, Fetco D, et al. Onset of multiple sclerosis before adulthood leads to failure of age-expected brain growth. NEUROLOGY. 2014;83(23):2140-6.
167.	Aulova KS, Toporkova LB, Lopatnikova JA, Alshevskaya AA, Sedykh SE, Buneva VN, et al. Changes in cell differentiation and proliferation lead to production of abzymes in EAE mice treated with DNA-Histone complexes. JOURNAL OF CELLULAR AND MOLECULAR MEDICINE. 2018;22(12):5816-32.
168.	Aulova KS, Toporkova LB, Lopatnikova JA, Alshevskaya AA, Sedykh SE, Buneva VN, et al. Changes in cell differentiation and proliferation lead to production of abzymes in EAE mice treated with DNA-Histone complexes. JOURNAL OF CELLULAR AND MOLECULAR MEDICINE. 2018;22(12):5816-32.
169.	Aun MV, Freua F, Marussi VHR, Giavina-Bianchi P. Case Report: Rapid Desensitization to Ocrelizumab for Multiple Sclerosis Is Effective and Safe. Front Immunol. 2022;13:840238.
170.	Aureli L, Gioia M, Cerbara I, Monaco S, Fasciglione GF, Marini S, et al. Structural Bases for Substrate and Inhibitor Recognition by Matrix Metalloproteinases. CURRENT MEDICINAL CHEMISTRY. 2008;15(22):2192-222.
171.	Aureli L, Gioia M, Cerbara I, Monaco S, Fasciglione GF, Marini S, et al. Structural bases for substrate and inhibitor recognition by matrix metaloproteinases. Current Medicinal Chemistry. 2008.
172.	Aureli L, Gioia M, Cerbara I, Monaco S, Fasciglione GF, Marini S, et al. Structural bases for substrate and inhibitor recognition by matrix metalloproteinases. Curr Med Chem. 2008;15(22):2192-222.
173.	Avasarala J. Anti-CD20 Cell Therapies in Multiple Sclerosis-A Fixed Dosing Schedule for Ocrelizumab is Overkill. Drug Target Insights. 2017;11:1177392817737515.
174.	Avasarala J, Guduru Z, McLouth CJ, Wilburn A, Talbert J, Sutton P, et al. Use of anti-TNF-α therapy in Crohn's disease is associated with increased incidence of multiple sclerosis. Mult Scler Relat Disord. 2021;51:102942.
175.	Averseng-Peaureaux D, Mizzi M, Colineaux H, Mahieu L, Pera MC, Brassat D, et al. Paediatric optic neuritis: factors leading to unfavourable outcome and relapses. BRITISH JOURNAL OF OPHTHALMOLOGY. 2018;102(6):808-13.
176.	Averseng-Peaureaux D, Mizzi M, Colineaux H, Mahieu L, Pera MC, Brassat D, et al. Paediatric optic neuritis: factors leading to unfavourable outcome and relapses. BRITISH JOURNAL OF OPHTHALMOLOGY. 2018;102(6):808-13.
177.	Ay A, Alkanli N, Atli E, Gurkan H, Gulyasar T, Guler S, et al. Investigation of Relationship Between Small Noncoding RNA (sncRNA) Expression Levels and Serum Iron, Copper, and Zinc Levels in Clinical Diagnosed Multiple Sclerosis Patients. Mol Neurobiol. 2023;60(2):875-83.
178.	Aydin O, Ellidag HY, Eren E, Kurtulus F, Yaman A, Yilmaz N. Ischemia modified albumin is an indicator of oxidative stress in multiple sclerosis. BIOCHEMIA MEDICA. 2014;24(3):383-9.
179.	Aydin O, Ellidag HY, Eren E, Kurtulus F, Yaman A, Yilmaz N. Ischemia modified albumin is an indicator of oxidative stress in multiple sclerosis. Biochemia Medica. 2014.
180.	Aydin O, Ellidag HY, Eren E, Kurtulus F, Yaman A, Yilmaz N. Ischemia modified albumin is an indicator of oxidative stress in multiple sclerosis. BIOCHEMIA MEDICA. 2014;24(3):383-9.
181.	Aydin O, Ellidag HY, Eren E, Kurtulus F, Yaman A, Yılmaz N. Ischemia modified albumin is an indicator of oxidative stress in multiple sclerosis. Biochem Med (Zagreb). 2014;24(3):383-9.
182.	Aykaç S, Eliaçık S. What are the trends in the treatment of multiple sclerosis in recent studies? - A bibliometric analysis with global productivity during 1980-2021. Mult Scler Relat Disord. 2022;68:104185.
183.	Aynacıoğlu A, Bilir A, Tuna MY. Involvement of midkine in autoimmune and autoinflammatory diseases. Mod Rheumatol. 2019;29(4):567-71.
184.	Azari H, Karimi E, Shekari M, Tahmasebi A, Nikpoor AR, Negahi AA, et al. Construction of a lncRNA-miRNA-mRNA network to determine the key regulators of the Th1/Th2 imbalance in multiple sclerosis. Epigenomics. 2021;13(22):1797-815.
185.	Azizi G, Haidari MR, Khorramizadeh M, Naddafi F, Sadria R, Javanbakht MH, et al. Effects of imatinib mesylate in mouse models of multiple sclerosis and in vitro determinants. Iran J Allergy Asthma Immunol. 2014;13(3):198-206.
186.	Azizi Z, Daryani N, Salim MR, Anbardan S. A case of concurrent multiple sclerosis and celiac disease. Govaresh. 2014.
187.	Azzam S, Broadwater L, Li S, Freeman EJ, McDonough J, Gregory RB. A SELDI mass spectrometry study of experimental autoimmune encephalomyelitis: sample preparation, reproducibility, and differential protein expression patterns. Proteome Sci. 2013;11(1):19.
188.	B T, W Z, H C, S C, X L, Dm H. Sex-specific differences in rim appearance of multiple sclerosis lesions on quantitative susceptibility mapping. Multiple Sclerosis and Related Disorders. 2020.
189.	B T, W Z, H C, S C, X L, Dm H. Sex-specific differences in rim appearance of multiple sclerosis lesions on quantitative susceptibility mapping. Mult Scler Relat Disord. 2020;45:102317.
190.	Baasch E. [Theoretical considerations on the etiology of multiple sclerosis. Is multiple sclerosis a mercury allergy?]. Schweiz Arch Neurol Neurochir Psychiatr. 1966;98(1):1-19.
191.	Bachrata B, Bollmann S, Jin J, Tourell M, Dal-Bianco A, Trattnig S, et al. Super-resolution QSM in little or no additional time for imaging (NATIve) using 2D EPI imaging in 3 orthogonal planes. Neuroimage. 2023;283:120419.
192.	Badam TV, Hellberg S, Mehta RB, Lechner-Scott J, Lea RA, Tost J, et al. CD4(+) T-cell DNA methylation changes during pregnancy significantly correlate with disease-associated methylation changes in autoimmune diseases. Epigenetics. 2022;17(9):1040-55.
193.	Badam TVS, de Weerd HA, Martínez-Enguita D, Olsson T, Alfredsson L, Kockum I, et al. A validated generally applicable approach using the systematic assessment of disease modules by GWAS reveals a multi-omic module strongly associated with risk factors in multiple sclerosis. BMC Genomics. 2021;22(1):631.
194.	Badaracco ME, Siri MVR, Toscano M, Rabinovich G, Pasquini JM. Low Iron Availability Avoids Demyelination Induced by Cuprizone.  9TH EUROPEAN MEETING ON GLIAL CELLS IN HEALTH AND DISEASE2009. p. 135-9.
195.	Badaracco ME, Siri MVR, Toscano M, Rabinovich G, Pasquini JM. Low Iron Availability Avoids Demyelination Induced by Cuprizone.  9TH EUROPEAN MEETING ON GLIAL CELLS IN HEALTH AND DISEASE2009. p. 135-9.
196.	Baderna V, Schultz J, Kearns LS, Fahey M, Thompson BA, Ruddle JB, et al. A novel AFG3L2 mutation close to AAA domain leads to aberrant OMA1 and OPA1 processing in a family with optic atrophy. ACTA NEUROPATHOLOGICA COMMUNICATIONS. 2020;8(1).
197.	Baderna V, Schultz J, Kearns LS, Fahey M, Thompson BA, Ruddle JB, et al. A novel AFG3L2 mutation close to AAA domain leads to aberrant OMA1 and OPA1 processing in a family with optic atrophy. ACTA NEUROPATHOLOGICA COMMUNICATIONS. 2020;8(1).
198.	Baeten K, Adriaensens P, Hendriks J, Theunissen E, Gelan J, Hellings N, et al. Tracking of myelin-reactive T cells in Experimental Autoimmune Encephalomyelitis (EAE) animals using small particles of iron oxide and MRI. NMR in Biomedicine. 2010.
199.	Baeten K, Adriaensens P, Hendriks J, Theunissen E, Gelan J, Hellings N, et al. Tracking of myelin-reactive T cells in experimental autoimmune encephalomyelitis (EAE) animals using small particles of iron oxide and MRI. NMR Biomed. 2010;23(6):601-9.
200.	Baeten K, Hendriks JJ, Hellings N, Theunissen E, Vanderlocht J, Ryck LD, et al. Visualisation of the kinetics of macrophage infiltration during experimental autoimmune encephalomyelitis by magnetic resonance imaging. Journal of Neuroimmunology. 2008.
201.	Baeten K, Hendriks JJ, Hellings N, Theunissen E, Vanderlocht J, Ryck LD, et al. Visualisation of the kinetics of macrophage infiltration during experimental autoimmune encephalomyelitis by magnetic resonance imaging. J Neuroimmunol. 2008;195(1-2):1-6.
202.	Baeten K, Hendriks JJA, Hellings N, Theunissen E, Vanderlocht J, De Ryck L, et al. Visualisation of the kinetics of macrophage infiltration during experimental autoimmune encephalomyelitis by magnetic resonance imaging. JOURNAL OF NEUROIMMUNOLOGY. 2008;195(1-2):1-6.
203.	Baeten K, Hendriks JJA, Hellings N, Theunissen E, Vanderlocht J, De Ryck L, et al. Visualisation of the kinetics of macrophage infiltration during experimental autoimmune encephalomyelitis by magnetic resonance imaging. JOURNAL OF NEUROIMMUNOLOGY. 2008;195(1-2):1-6.
204.	Báez Martín MM, Cruz Menor M, Cabrera Abreu I, Cabrera Gómez J, Galvizu Sánchez R. Utility of auditory middle latency response in patients with Multiple Sclerosis relapsing-remitting type. Revista Ecuatoriana de Neurologia. 2007.
205.	Bagchi B, Al-Sabi A, Kaza S, Scholz D, O'Leary VB, Dolly JO, et al. Disruption of Myelin Leads to Ectopic Expression of K(V)1.1 Channels with Abnormal Conductivity of Optic Nerve Axons in a Cuprizone-Induced Model of Demyelination. PLOS ONE. 2014;9(2).
206.	Bagchi B, Al-Sabi A, Kaza S, Scholz D, O'Leary VB, Dolly JO, et al. Disruption of Myelin Leads to Ectopic Expression of K(V)1.1 Channels with Abnormal Conductivity of Optic Nerve Axons in a Cuprizone-Induced Model of Demyelination. PLOS ONE. 2014;9(2).
207.	Bagnato F, Durastanti V, Finamore L, Volante G, Millefiorini E. Beta-2 microglobulin and neopterin as markers of disease activity in multiple sclerosis. Neurol Sci. 2003;24 Suppl 5:S301-4.
208.	Bagnato F, Hametner S, Boyd E, Endmayr V, Shi Y, Ikonomidou V, et al. Untangling the R2* contrast in multiple sclerosis: A combined MRI-histology study at 7.0 Tesla. PLoS ONE. 2018.
209.	Bagnato F, Hametner S, Boyd E, Endmayr V, Shi Y, Ikonomidou V, et al. Untangling the R2* contrast in multiple sclerosis: A combined MRI-histology study at 7.0 Tesla. PLoS One. 2018;13(3):e0193839.
210.	Bagnato F, Hametner S, Boyd E, Endmayr V, Shi YP, Ikonomidou V, et al. Untangling the R2*contrast in multiple sclerosis: A combined MRI-histology study at 7.0 Tesla. PLOS ONE. 2018;13(3).
211.	Bagnato F, Hametner S, Boyd E, Endmayr V, Shi YP, Ikonomidou V, et al. Untangling the R2*contrast in multiple sclerosis: A combined MRI-histology study at 7.0 Tesla. PLOS ONE. 2018;13(3).
212.	Bagnato F, Hametner S, Welch EB. Visualizing iron in multiple sclerosis. MAGNETIC RESONANCE IMAGING. 2013;31(3):376-84.
213.	Bagnato F, Hametner S, Welch EB. Visualizing iron in multiple sclerosis. Magnetic Resonance Imaging. 2013.
214.	Bagnato F, Hametner S, Welch EB. Visualizing iron in multiple sclerosis. MAGNETIC RESONANCE IMAGING. 2013;31(3):376-84.
215.	Bagnato F, Hametner S, Welch EB. Visualizing iron in multiple sclerosis. Magn Reson Imaging. 2013;31(3):376-84.
216.	Bagnato F, Hametner S, Welch EB. Visualizing iron in multiple sclerosis. Magnetic resonance imaging. 2013;31(3):376-84.
217.	Bagnato F, Hametner S, Yao B, van Gelderen P, Merkle H, Cantor FK, et al. Tracking iron in multiple sclerosis: a combined imaging and histopathological study at 7 Tesla. BRAIN. 2011;134:3599-612.
218.	Bagnato F, Hametner S, Yao B, Van Gelderen P, Merkle H, Cantor FK, et al. Tracking iron in multiple sclerosis: A combined imaging and histopathological study at 7 Tesla. Brain. 2011.
219.	Bagnato F, Hametner S, Yao B, van Gelderen P, Merkle H, Cantor FK, et al. Tracking iron in multiple sclerosis: a combined imaging and histopathological study at 7 Tesla. BRAIN. 2011;134:3599-612.
220.	Bagnato F, Hametner S, Yao B, van Gelderen P, Merkle H, Cantor FK, et al. Tracking iron in multiple sclerosis: a combined imaging and histopathological study at 7 Tesla. Brain. 2011;134(Pt 12):3602-15.
221.	Bagnato F, Hametner S, Yao B, van Gelderen P, Merkle H, Cantor FK, et al. Tracking iron in multiple sclerosis: a combined imaging and histopathological study at 7 Tesla. BRAIN. 2011;134:3599-612.
222.	Bagnato F, Hametner S, Yao B, van Gelderen P, Merkle H, Cantor FK, et al. Tracking iron in multiple sclerosis: a combined imaging and histopathological study at 7 Tesla. Brain. 2011;134(12):3602-15.
223.	Bagnato F, Hametner S, Yao B, van Gelderen P, Merkle H, Cantor FK, et al. Tracking iron in multiple sclerosis: a combined imaging and histopathological study at 7 tesla. Brain 134 (Pt 12): 3602–3615. 2011.
224.	Bagnato F, Pozzilli C, Scagnolari C, Bellomi F, Pasqualetti P, Gasperini C, et al. A one-year study on the pharmacodynamic profile of interferon-beta1a in MS. Neurology. 2002;58(9):1409-11.
225.	Bagnato F, Sati P, Hemond CC, Elliott C, Gauthier SA, Harrison DM, et al. Imaging chronic active lesions in multiple sclerosis: a consensus statement. Brain. 2024;147(9):2913-33.
226.	Bagnato F, Zivadinov R, Cecchinelli D, Tancredi A, Grop A, Pierallini A, et al. beta2-microglobulin serum level is not a marker of disease activity in multiple sclerosis. Eur J Neurol. 2004;11(7):455-60.
227.	Bailey CV. Lead Content of the Spinal Fluid with Special Reference to Multiple Sclerosis.(Proc. Soc. Exptl. Biol. Med., vol. xxxv, pp. 210–3, 1936.) Garvey, PH, and Rockwell, FV. Journal of Mental Science. 1938;84(348):239-.
228.	Bakhshai J, Bleu-Lainé R, Jung M, Lim J, Reyes C, Sun L, et al. The cost effectiveness and budget impact of natalizumab for formulary inclusion. J Med Econ. 2010;13(1):63-9.
229.	Bakshi R, Benedict RH, Bermel RA, Caruthers SD, Puli SR, Tjoa CW, et al. T2 hypointensity in the deep gray matter of patients with multiple sclerosis: a quantitative magnetic resonance imaging study. Arch Neurol. 2002;59(1):62-8.
230.	Bakshi R, Benedict RHB, Bermel RA, Caruthers SD, Puli SR, Tjoa CW, et al. T2 hypointensity in the deep gray matter of patients with multiple sclerosis - A quantitative magnetic resonance imaging study. ARCHIVES OF NEUROLOGY. 2002;59(1):62-8.
231.	Bakshi R, Benedict RHB, Bermel RA, Caruthers SD, Puli SR, Tjoa CW, et al. T2 hypointensity in the deep gray matter of patients with multiple sclerosis: A quantitative magnetic resonance imaging study. Archives of Neurology. 2002.
232.	Bakshi R, Benedict RHB, Bermel RA, Caruthers SD, Puli SR, Tjoa CW, et al. T2 hypointensity in the deep gray matter of patients with multiple sclerosis - A quantitative magnetic resonance imaging study. ARCHIVES OF NEUROLOGY. 2002;59(1):62-8.
233.	Bakshi R, Dmochowski J, Shaikh ZA, Jacobs L. Gray matter T2 hypointensity is related to plaques and atrophy in the brains of multiple sclerosis patients. Journal of the Neurological Sciences. 2001.
234.	Bakshi R, Dmochowski J, Shaikh ZA, Jacobs L. Gray matter T2 hypointensity is related to plaques and atrophy in the brains of multiple sclerosis patients. J Neurol Sci. 2001;185(1):19-26.
235.	Bakshi R, Shaikh ZA, Janardhan V. MRI T2 shortening ('black T2') in multiple sclerosis: frequency, location, and clinical correlation. NEUROREPORT. 2000;11(1):15-21.
236.	Bakshi R, Shaikh ZA, Janardhan V. MRI T2 shortening ('black T2') in multiple sclerosis: Frequency, location, and clinical correlation. NeuroReport. 2000.
237.	Bakshi R, Shaikh ZA, Janardhan V. MRI T2 shortening ('black T2') in multiple sclerosis: frequency, location, and clinical correlation. Neuroreport. 2000;11(1):15-21.
238.	Bakshi R, Shaikh ZA, Janardhan V. MRI T2 shortening ('black T2') in multiple sclerosis: frequency, location, and clinical correlation. NEUROREPORT. 2000;11(1):15-21.
239.	Balaratnam MS, Stevenson VL. Intrathecal baclofen pumps: what the neurologist needs to know. Pract Neurol. 2022;22(3):241-6.
240.	Baldacchino K, Peveler WJ, Lemgruber L, Smith RS, Scharler C, Hayden L, et al. Myelinated axons are the primary target of hemin-mediated oxidative damage in a model of the central nervous system. Exp Neurol. 2022;354:114113.
241.	Ballerini C. Experimental Autoimmune Encephalomyelitis. Methods Mol Biol. 2021;2285:375-84.
242.	Bamm VV, Harauz G. Hemoglobin as a source of iron overload in multiple sclerosis: does multiple sclerosis share risk factors with vascular disorders? CELLULAR AND MOLECULAR LIFE SCIENCES. 2014;71(10):1789-98.
243.	Bamm VV, Harauz G. Hemoglobin as a source of iron overload in multiple sclerosis: Does multiple sclerosis share risk factors with vascular disorders? Cellular and Molecular Life Sciences. 2014.
244.	Bamm VV, Harauz G. Hemoglobin as a source of iron overload in multiple sclerosis: does multiple sclerosis share risk factors with vascular disorders? CELLULAR AND MOLECULAR LIFE SCIENCES. 2014;71(10):1789-98.
245.	Bamm VV, Harauz G. Hemoglobin as a source of iron overload in multiple sclerosis: does multiple sclerosis share risk factors with vascular disorders? Cell Mol Life Sci. 2014;71(10):1789-98.
246.	Bamm VV, Harauz G. Hemoglobin as a source of iron overload in multiple sclerosis: does multiple sclerosis share risk factors with vascular disorders? CELLULAR AND MOLECULAR LIFE SCIENCES. 2014;71(10):1789-98.
247.	Bamm VV, Harauz G. Hemoglobin as a source of iron overload in multiple sclerosis: does multiple sclerosis share risk factors with vascular disorders? Cellular and molecular life sciences. 2014;71(10):1789-98.
248.	Bamm VV, Henein MEL, Sproul SLJ, Lanthier DK, Harauz G. Potential role of ferric hemoglobin in MS pathogenesis: Effects of oxidative stress and extracellular methemoglobin or its degradation products on myelin components. FREE RADICAL BIOLOGY AND MEDICINE. 2017;112:494-503.
249.	Bamm VV, Henein MEL, Sproul SLJ, Lanthier DK, Harauz G. Potential role of ferric hemoglobin in MS pathogenesis: Effects of oxidative stress and extracellular methemoglobin or its degradation products on myelin components. Free Radical Biology and Medicine. 2017.
250.	Bamm VV, Henein MEL, Sproul SLJ, Lanthier DK, Harauz G. Potential role of ferric hemoglobin in MS pathogenesis: Effects of oxidative stress and extracellular methemoglobin or its degradation products on myelin components. Free Radic Biol Med. 2017;112:494-503.
251.	Bamm VV, Henein MEL, Sproul SLJ, Lanthier DK, Harauz G. Potential role of ferric hemoglobin in MS pathogenesis: Effects of oxidative stress and extracellular methemoglobin or its degradation products on myelin components. FREE RADICAL BIOLOGY AND MEDICINE. 2017;112:494-503.
252.	Bamm VV, Lanthier DK, Stephenson EL, Smith GS, Harauz G. In vitro study of the direct effect of extracellular hemoglobin on myelin components. Biochim Biophys Acta. 2015;1852(1):92-103.
253.	Bamm VV, Lanthier DK, Stephenson EL, Smith GST, Harauz G. In vitro study of the direct effect of extracellular hemoglobin on myelin components. BIOCHIMICA ET BIOPHYSICA ACTA-MOLECULAR BASIS OF DISEASE. 2015;1852(1):92-103.
254.	Bamm VV, Lanthier DK, Stephenson EL, Smith GST, Harauz G. In vitro study of the direct effect of extracellular hemoglobin on myelin components. Biochimica et Biophysica Acta - Molecular Basis of Disease. 2015.
255.	Bamm VV, Lanthier DK, Stephenson EL, Smith GST, Harauz G. In vitro study of the direct effect of extracellular hemoglobin on myelin components. BIOCHIMICA ET BIOPHYSICA ACTA-MOLECULAR BASIS OF DISEASE. 2015;1852(1):92-103.
256.	Bammer H. [Ceruloplasmin and copper metabolism in multiple sclerosis]. Dtsch Z Nervenheilkd. 1966;189(4):312-29.
257.	Bankoti J, Apeltsin L, Hauser SL, Allen S, Albertolle ME, Witkowska HE, et al. In multiple sclerosis, oligoclonal bands connect to peripheral B-cell responses. ANNALS OF NEUROLOGY. 2014;75(2):266-76.
258.	Bankoti J, Apeltsin L, Hauser SL, Allen S, Albertolle ME, Witkowska HE, et al. In multiple sclerosis, oligoclonal bands connect to peripheral B-cell responses. Ann Neurol. 2014;75(2):266-76.
259.	Bankoti J, Apeltsin L, Hauser SL, Allen S, Albertolle ME, Witkowska HE, et al. In multiple sclerosis, oligoclonal bands connect to peripheral B-cell responses. ANNALS OF NEUROLOGY. 2014;75(2):266-76.
260.	Bar-Or A, Rieckmann P, Traboulsee A, Yong VW. Targeting Progressive Neuroaxonal Injury Lessons from Multiple Sclerosis. CNS DRUGS. 2011;25(9):783-99.
261.	Bar-Or A, Rieckmann P, Traboulsee A, Yong VW. Targeting progressive neuroaxonal injury: Lessons from multiple sclerosis. CNS Drugs. 2011.
262.	Bar-Or A, Rieckmann P, Traboulsee A, Yong VW. Targeting progressive neuroaxonal injury: lessons from multiple sclerosis. CNS Drugs. 2011;25(9):783-99.
263.	Barabino AV, Gandullia P, Calvi A, Vignola S, Arrigo S, Marco RD. Sudden blindness in a child with Crohn's disease. World J Gastroenterol. 2011;17(38):4344-6.
264.	Baracchini C, Atzori M, Gallo P. CCSVI and MS: no meaning, no fact. NEUROLOGICAL SCIENCES. 2013;34(3):269-79.
265.	Baracchini C, Atzori M, Gallo P. CCSVI and MS: No meaning, no fact. Neurological Sciences. 2013.
266.	Baracchini C, Atzori M, Gallo P. CCSVI and MS: no meaning, no fact. Neurol Sci. 2013;34(3):269-79.
267.	Baracchini C, Gallo P. Fact or fiction: Chronic cerebro-spinal insufficiency. Perspectives in Medicine. 2012.
268.	Baranger K, Rivera S, Liechti FD, Grandgirard D, Bigas J, Seco J, et al. Endogenous and synthetic MMP inhibitors in CNS physiopathology. Prog Brain Res. 2014;214:313-51.
269.	Baranovicova E, Kantorova E, Kalenska D, Lichardusova L, Bittsan-Sky M, Dobrota D. Thalamic paramagnetic iron by T2* relaxometry correlates with severity of multiple sclerosis. J Biomed Res. 2017;31(4):301-5.
270.	Baranovicova E, Kantorova E, Kalenska D, Lichardusova L, Bittsan-Sky M, Dobrota D. Thalamic paramagnetic iron by T2* relaxometry correlates with severity of multiple sclerosis. Journal of Biomedical Research. 2017;31(4):301.
271.	Baranovicova E, Kantorova E, Kalenska D, Lichardusova L, Bittsansky M, Dobrota D. Thalamic paramagnetic iron by T2* relaxometry correlates with severity of multiple sclerosis. JOURNAL OF BIOMEDICAL RESEARCH. 2017;31(4):301-5.
272.	Baranovicova E, Kantorova E, Kalenska D, Lichardusova L, Bittsansky M, Dobrota D. Thalamic paramagnetic iron by T2* relaxometry correlates with severity of multiple sclerosis. Journal of Biomedical Research. 2017.
273.	Baranovicova E, Kantorova E, Kalenska D, Lichardusova L, Bittsansky M, Dobrota D. Thalamic paramagnetic iron by T2* relaxometry correlates with severity of multiple sclerosis. JOURNAL OF BIOMEDICAL RESEARCH. 2017;31(4):301-5.
274.	Baranovicova E, Kantorova E, Kalenska D, Lichardusova L, Bittsansky M, Dobrota D. Thalamic paramagnetic iron by T2* relaxometry correlates with severity of multiple sclerosis. JOURNAL OF BIOMEDICAL RESEARCH. 2017;31(4):301-5.
275.	Baranzini SE. The genetics of autoimmune diseases: a networked perspective. Curr Opin Immunol. 2009;21(6):596-605.
276.	Baranzini SE. Symposium 2-1  The autoimmunome: Similarities and differences among genetic susceptibility to common immune-related diseases. Nihon Rinsho Meneki Gakkai Kaishi. 2014;37(4):261.
277.	Barbato L, Scalise F, Grasso MA, Spagnolo S. Iron man: extreme endovascular treatment for chronic cerebrospinal venous insufficiency. Eur J Cardiothorac Surg. 2015;47(5):934-5.
278.	Barkhane Z, Elmadi J, Satish Kumar L, Pugalenthi LS, Ahmad M, Reddy S. Multiple Sclerosis and Autoimmunity: A Veiled Relationship. Cureus. 2022;14(4):e24294.
279.	Barkhof F, Thomas DL. Mapping deep gray matter iron in multiple sclerosis by using quantitative magnetic susceptibility. Radiology. 2018.
280.	Barkhof F, Thomas DL. Mapping Deep Gray Matter Iron in Multiple Sclerosis by Using Quantitative Magnetic Susceptibility. RADIOLOGY. 2018;289(2):497-8.
281.	Barkhof F, Thomas DL. Mapping Deep Gray Matter Iron in Multiple Sclerosis by Using Quantitative Magnetic Susceptibility. Radiology. 2018;289(2):497-8.
282.	Barkhof F, Thomas DL. Mapping deep gray matter iron in multiple sclerosis by using quantitative magnetic susceptibility. Radiological Society of North America; 2018. p. 497-8.
283.	Barlow J, Edwards R, Turner A. The experience of attending a lay-led, chronic disease self-management programme from the perspective of participants with multiple sclerosis. PSYCHOLOGY & HEALTH. 2009;24(10):1167-80.
284.	Barlow J, Edwards R, Turner A. The experience of attending a lay-led, chronic disease self-management programme from the perspective of participants with multiple sclerosis. PSYCHOLOGY & HEALTH. 2009;24(10):1167-80.
285.	Barlow J, Edwards R, Turner A. The experience of attending a lay-led, chronic disease self-management programme from the perspective of participants with multiple sclerosis. PSYCHOLOGY & HEALTH. 2009;24(10):1167-80.
286.	Barlow J, Turner A, Edwards R, Gilchrist M. A randomised controlled trial of lay-led self-management for people with multiple sclerosis. PATIENT EDUCATION AND COUNSELING. 2009;77(1):81-9.
287.	Barlow J, Turner A, Edwards R, Gilchrist M. A randomised controlled trial of lay-led self-management for people with multiple sclerosis. PATIENT EDUCATION AND COUNSELING. 2009;77(1):81-9.
288.	Barlow J, Turner A, Edwards R, Gilchrist M. A randomised controlled trial of lay-led self-management for people with multiple sclerosis. PATIENT EDUCATION AND COUNSELING. 2009;77(1):81-9.
289.	Barnish M, Sheikh M, Scholey A. Nutrient Therapy for the Improvement of Fatigue Symptoms. Nutrients. 2023;15(9).
290.	Barnum SR, Jones JL, Benveniste EN. Interferon-gamma regulation of C3 gene expression in human astroglioma cells. J Neuroimmunol. 1992;38(3):275-82.
291.	Barnum SR, Jones JL, Benveniste EN. Interleukin-1 and tumor necrosis factor-mediated regulation of C3 gene expression in human astroglioma cells. Glia. 1993;7(3):225-36.
292.	Barron KD. The microglial cell. A historical review. JOURNAL OF THE NEUROLOGICAL SCIENCES. 1995;134:57-68.
293.	Barron KD. The microglial cell. A historical review. Journal of the Neurological Sciences. 1995.
294.	Barron KD. The microglial cell. A historical review. J Neurol Sci. 1995;134 Suppl:57-68.
295.	Barron KD. The microglial cell. A historical review. JOURNAL OF THE NEUROLOGICAL SCIENCES. 1995;134:57-68.
296.	Bartak H, Fareh T, Ben Othman N, Viard D, Cohen M, Rocher F, et al. Dental Adverse Effects of Anti-CD20 Therapies. Neurol Ther. 2024;13(3):917-30.
297.	Bartolini S, Inzitari D, Castagnoli A, Amaducci L. Correlation of isotopic cisternographic patterns in multiple sclerosis with CSF IgG values. Ann Neurol. 1982;12(5):486-9.
298.	Bartos A, Fialová L, Soukupová J, Kukal J, Malbohan I, Pit'ha J. Elevated intrathecal antibodies against the medium neurofilament subunit in multiple sclerosis. J Neurol. 2007;254(1):20-5.
299.	Bartos A, Fialová L, Soukupová J, Kukal J, Malbohan I, Pitha J. Antibodies against light neurofilaments in multiple sclerosis patients. Acta Neurol Scand. 2007;116(2):100-7.
300.	Basak J, Majsterek I. miRNA-Dependent CD4(+) T Cell Differentiation in the Pathogenesis of Multiple Sclerosis. Mult Scler Int. 2021;2021:8825588.
301.	Basiri K, Etemadifar M, Maghzi AH, Zarghami N. Frequency of myasthenia gravis in multiple sclerosis: Report of five cases from Isfahan, Iran. Neurol India. 2009;57(5):638-40.
302.	Bassil F, Monvoisin A, Canron MH, Vital A, Meissner WG, Tison F, et al. Region-Specific Alterations of Matrix Metalloproteinase Activity in Multiple System Atrophy. MOVEMENT DISORDERS. 2015;30(13):1802-12.
303.	Bassil F, Monvoisin A, Canron MH, Vital A, Meissner WG, Tison F, et al. Region-Specific Alterations of Matrix Metalloproteinase Activity in Multiple System Atrophy. Movement Disorders. 2015.
304.	Bassil F, Monvoisin A, Canron MH, Vital A, Meissner WG, Tison F, et al. Region-Specific Alterations of Matrix Metalloproteinase Activity in Multiple System Atrophy. Mov Disord. 2015;30(13):1802-12.
305.	Bassil F, Monvoisin A, Canron MH, Vital A, Meissner WG, Tison F, et al. Region-Specific Alterations of Matrix Metalloproteinase Activity in Multiple System Atrophy. MOVEMENT DISORDERS. 2015;30(13):1802-12.
306.	Bates MN. Mercury amalgam dental fillings: an epidemiologic assessment. Int J Hyg Environ Health. 2006;209(4):309-16.
307.	Batur-Caglayan HZ, Irkec C, Yildirim-Capraz I, Atalay-Akyurek N, Dumlu S. A case of multiple sclerosis and celiac disease. Case Rep Neurol Med. 2013;2013:576921.
308.	Bauthman MS. Effectiveness of Anti-Cluster of Differentiation 20 as a Disease-Modifying Therapy in Multiple Sclerosis Across Its Different Phenotypes at the University Hospital of Caen. Cureus. 2022;14(2):e22120.
309.	Bawand R, Ghiasian M, Fathoallahi N, Moradi A. Effects of disease-modifying treatments discontinuation in patients with relapsing-remitting multiple sclerosis: A 5 year prospective cohort study. Mult Scler Relat Disord. 2022;63:103857.
310.	Bayliss OB, Adams CW, Hallpike JF. The PASDORO method for simultaneously demonstrating DNA and lipids in the brain. Histochem J. 1970;2(1):87-9.
311.	Bazzi S, Caslin B, Raskin M, Karmakar A, Mohler K, Maguire C, et al. Binge alcohol consumption in murine models of multiple sclerosis leads to sex-specific disease development. MULTIPLE SCLEROSIS JOURNAL. 2020;26(3_SUPPL):645-.
312.	Beard JL, Connor JR, Jones BC. Iron in the brain. Nutr Rev. 1993;51(6):157-70.
313.	Beck CW, Bender MJ. Aragonite, CaCO3, as urinary calculi. J Urol. 1969;101(2):208-11.
314.	Beckett RP, Davidson AH, Drummond AH, Huxley P, Whittaker M. Recent advances in matrix metalloproteinase inhibitor research. DRUG DISCOVERY TODAY. 1996;1(1):16-26.
315.	Beckett RP, Davidson AH, Drummond AH, Huxley P, Whittaker M. Recent advances in matrix metalloproteinase inhibitor research. Drug Discovery Today. 1996.
316.	Beckmann N, Cannet C, Babin AL, Ble FX, Zurbruegg S, Kneuer R, et al. In vivo visualization of macrophage infiltration and activity in inflammation using magnetic resonance imaging. WILEY INTERDISCIPLINARY REVIEWS-NANOMEDICINE AND NANOBIOTECHNOLOGY. 2009;1(3):272-98.
317.	Beckmann N, Cannet C, Babin AL, Blé FX, Zurbruegg S, Kneuer R, et al. In vivo visualization of macrophage infiltration and activity in inflammation using magnetic resonance imaging. Wiley Interdisciplinary Reviews: Nanomedicine and Nanobiotechnology. 2009.
318.	Beckmann N, Cannet C, Babin AL, Blé FX, Zurbruegg S, Kneuer R, et al. In vivo visualization of macrophage infiltration and activity in inflammation using magnetic resonance imaging. Wiley Interdiscip Rev Nanomed Nanobiotechnol. 2009;1(3):272-98.
319.	Becucci L, Benci S, Nuti F, Real-Fernandez F, Vaezi Z, Stella L, et al. Interaction Study of Phospholipid Membranes with an N-Glucosylated beta-Turn Peptide Structure Detecting Autoantibodies Biomarkers of Multiple Sclerosis. MEMBRANES. 2015;5(4):576-96.
320.	Becucci L, Benci S, Nuti F, Real-Fernandez F, Vaezi Z, Stella L, et al. Interaction study of phospholipid membranes with an n-glucosylated β-turn peptide structure detecting autoantibodies biomarkers of multiple sclerosis. Membranes. 2015.
321.	Becucci L, Benci S, Nuti F, Real-Fernandez F, Vaezi Z, Stella L, et al. Interaction Study of Phospholipid Membranes with an N-Glucosylated β-Turn Peptide Structure Detecting Autoantibodies Biomarkers of Multiple Sclerosis. Membranes (Basel). 2015;5(4):576-96.
322.	Becucci L, Benci S, Nuti F, Real-Fernandez F, Vaezi Z, Stella L, et al. Interaction Study of Phospholipid Membranes with an N-Glucosylated beta-Turn Peptide Structure Detecting Autoantibodies Biomarkers of Multiple Sclerosis. MEMBRANES. 2015;5(4):576-96.
323.	Behl T, Kaur G, Sehgal A, Bhardwaj S, Singh S, Buhas C, et al. Multifaceted Role of Matrix Metalloproteinases in Neurodegenerative Diseases: Pathophysiological and Therapeutic Perspectives. INTERNATIONAL JOURNAL OF MOLECULAR SCIENCES. 2021;22(3).
324.	Behl T, Kaur G, Sehgal A, Bhardwaj S, Singh S, Buhas C, et al. Multifaceted role of matrix metalloproteinases in neurodegenerative diseases: Pathophysiological and therapeutic perspectives. International Journal of Molecular Sciences. 2021.
325.	Behl T, Kaur G, Sehgal A, Bhardwaj S, Singh S, Buhas C, et al. Multifaceted Role of Matrix Metalloproteinases in Neurodegenerative Diseases: Pathophysiological and Therapeutic Perspectives. Int J Mol Sci. 2021;22(3).
326.	Behzadi AH, Gupta A, Prince MR. Potential role of lipoic acid as a chelator in prevention and treatment of gadolinium brain retention. Med Hypotheses. 2018;114:29.
327.	Belgrade MJ. Following the clues to neuropathic pain - Distribution and other leads reveal the cause and the treatment approach. POSTGRADUATE MEDICINE. 1999;106(6):127-+.
328.	Belgrade MJ. Following the clues to neuropathic pain - Distribution and other leads reveal the cause and the treatment approach. POSTGRADUATE MEDICINE. 1999;106(6):127-+.
329.	Bellizzi A, Anzivino E, Rodio DM, Cioccolo S, Scrivo R, Morreale M, et al. Human Polyomavirus JC monitoring and noncoding control region analysis in dynamic cohorts of individuals affected by immune-mediated diseases under treatment with biologics: an observational study. Virol J. 2013;10:298.
330.	Bellizzi A, Barucca V, Fioriti D, Colosimo MT, Mischitelli M, Anzivino E, et al. Early years of biological agents therapy in Crohn's disease and risk of the human polyomavirus JC reactivation. J Cell Physiol. 2010;224(2):316-26.
331.	Belousov MI, Sholomov, II. Eliseev Yu. Yu. Antoropogennoe zagryaznenie gorodskoy sredy kadmiem i nikelem i ego vozmozhnoe vliyanie na razvitie rasseyannogo skleroza [Anthropogenic impurity of the urban environment by cadmium and nickel and its possible influence on the development of multiple sclerosis]. Izv Samar nauch tsentra RAN. 2014:764-8.
332.	Belova AN, Solovieva VS, Boyko AN. Anemia and dysregulation of iron metabolism in multiple sclerosis. Zhurnal Nevrologii i Psihiatrii imeni SS Korsakova. 2018.
333.	Belova AN, Solovieva VS, Boyko AN. [Anemia and dysregulation of iron metabolism in multiple sclerosis]. Zh Nevrol Psikhiatr Im S S Korsakova. 2018;118(8. Vyp. 2):10-7.
334.	Belova AN, Solovieva VS, Boyko AN. Anemia and dysregulation of iron metabolism in multiple sclerosis. Zhurnal Nevrologii i Psikhiatrii Imeni SS Korsakova. 2018;118(8. Vyp. 2):10-7.
335.	Ben-Ami E, Miller A, Berrih-Aknin S. T cells from autoimmune patients display reduced sensitivity to immunoregulation by mesenchymal stem cells: role of IL-2. Autoimmun Rev. 2014;13(2):187-96.
336.	Ben-Zacharia AB, Bethoux FA, Volandes A. Self-Perceived Knowledge and Comfort Discussing Palliative Care and End-of-Life Issues among Professionals Managing Neuroinflammatory Diseases. J Palliat Med. 2021;24(5):725-35.
337.	Benarroch EE. Nrf2, cellular redox regulation, and neurologic implications. Neurology. 2017.
338.	Benjamins JA, Nedelkoska L, Bealmear B, Lisak RP. ACTH protects mature oligodendroglia from excitotoxic and inflammation-related damage in vitro. Glia. 2013;61(8):1206-17.
339.	Benjamins JA, Nedelkoska L, Lisak RP. Adrenocorticotropin hormone 1-39 promotes proliferation and differentiation of oligodendroglial progenitor cells and protects from excitotoxic and inflammation-related damage. J Neurosci Res. 2014;92(10):1243-51.
340.	Bentley S, Morgan L, Exall E, Arbuckle R, Rossom RC, Roche N, et al. Qualitative Interviews to Support Development and Cognitive Debriefing of the Adelphi Adherence Questionnaire (ADAQ©): A Patient-Reported Measure of Medication Adherence Developed for Use in a Range of Diseases, Treatment Modalities, and Countries. Patient Prefer Adherence. 2022;16:2579-92.
341.	Bepari AK, Takebayashi H, Namme JN, Rahman GMS, Reza HM. A computational study to target necroptosis via RIPK1 inhibition. J Biomol Struct Dyn. 2023;41(14):6502-17.
342.	Berard JA, Freedman MS, Marrie RA, Marriott JJ, Atkins HL, Szwajcer D, et al. Mesenchymal stem cell therapy and cognition in MS: Preliminary findings from a phase II clinical trial. Mult Scler Relat Disord. 2022;61:103779.
343.	Berard JL, Zarruk JG, Arbour N, Prat A, Yong VW, Jacques FH, et al. Lipocalin 2 is a novel immune mediator of experimental autoimmune encephalomyelitis pathogenesis and is modulated in multiple sclerosis. GLIA. 2012;60(7):1145-59.
344.	Berard JL, Zarruk JG, Arbour N, Prat A, Yong VW, Jacques FH, et al. Lipocalin 2 is a novel immune mediator of experimental autoimmune encephalomyelitis pathogenesis and is modulated in multiple sclerosis. GLIA. 2012.
345.	Berard JL, Zarruk JG, Arbour N, Prat A, Yong VW, Jacques FH, et al. Lipocalin 2 is a novel immune mediator of experimental autoimmune encephalomyelitis pathogenesis and is modulated in multiple sclerosis. Glia. 2012;60(7):1145-59.
346.	Berard JL, Zarruk JG, Arbour N, Prat A, Yong VW, Jacques FH, et al. Lipocalin 2 is a novel immune mediator of experimental autoimmune encephalomyelitis pathogenesis and is modulated in multiple sclerosis. GLIA. 2012;60(7):1145-59.
347.	Berg D. Hyperechogenicity of the substantia nigra: pitfalls in assessment and specificity for Parkinson's disease. JOURNAL OF NEURAL TRANSMISSION. 2011;118(3):453-61.
348.	Berg D. Hyperechogenicity of the substantia nigra: pitfalls in assessment and specificity for Parkinson's disease. Journal of neural transmission (Vienna, Austria : 1996). 2011.
349.	Berg D. Hyperechogenicity of the substantia nigra: pitfalls in assessment and specificity for Parkinson's disease. J Neural Transm (Vienna). 2011;118(3):453-61.
350.	Bergaglio T, Luchicchi A, Schenk GJ. Engine Failure in Axo-Myelinic Signaling: A Potential Key Player in the Pathogenesis of Multiple Sclerosis. Front Cell Neurosci. 2021;15:610295.
351.	Bergien SO, Petersen CM, Lynning M, Kristiansen M, Skovgaard L. Use of natural medicine and dietary supplements concomitant with conventional medicine among people with Multiple Sclerosis. MULTIPLE SCLEROSIS AND RELATED DISORDERS. 2020;44.
352.	Bergien SO, Petersen CM, Lynning M, Kristiansen M, Skovgaard L. Use of natural medicine and dietary supplements concomitant with conventional medicine among people with Multiple Sclerosis. Multiple Sclerosis and Related Disorders. 2020.
353.	Bergien SO, Petersen CM, Lynning M, Kristiansen M, Skovgaard L. Use of natural medicine and dietary supplements concomitant with conventional medicine among people with Multiple Sclerosis. Mult Scler Relat Disord. 2020;44:102197.
354.	Bergien SO, Petersen CM, Lynning M, Kristiansen M, Skovgaard L. Use of natural medicine and dietary supplements concomitant with conventional medicine among people with Multiple Sclerosis. MULTIPLE SCLEROSIS AND RELATED DISORDERS. 2020;44.
355.	Bergsland N, Agostini S, Lagana MM, Mancuso R, Mendozzi L, Tavazzi E, et al. Serum iron concentration is associated with subcortical deep gray matter iron levels in multiple sclerosis patients. NEUROREPORT. 2017;28(11):645-8.
356.	Bergsland N, Agostini S, Laganà MM, Mancuso R, Mendozzi L, Tavazzi E, et al. Serum iron concentration is associated with subcortical deep gray matter iron levels in multiple sclerosis patients. NeuroReport. 2017.
357.	Bergsland N, Agostini S, Lagana MM, Mancuso R, Mendozzi L, Tavazzi E, et al. Serum iron concentration is associated with subcortical deep gray matter iron levels in multiple sclerosis patients. NEUROREPORT. 2017;28(11):645-8.
358.	Bergsland N, Agostini S, Laganà MM, Mancuso R, Mendozzi L, Tavazzi E, et al. Serum iron concentration is associated with subcortical deep gray matter iron levels in multiple sclerosis patients. Neuroreport. 2017;28(11):645-8.
359.	Bergsland N, Agostini S, Lagana MM, Mancuso R, Mendozzi L, Tavazzi E, et al. Serum iron concentration is associated with subcortical deep gray matter iron levels in multiple sclerosis patients. NEUROREPORT. 2017;28(11):645-8.
360.	Bergsland N, Agostini S, Laganà MM, Mancuso R, Mendozzi L, Tavazzi E, et al. Serum iron concentration is associated with subcortical deep gray matter iron levels in multiple sclerosis patients. Neuroreport. 2017;28(11):645-8.
361.	Bergsland N, Tavazzi E, Lagana MM, Baglio F, Cecconi P, Viotti S, et al. White Matter Tract Injury is Associated with Deep Gray Matter Iron Deposition in Multiple Sclerosis. JOURNAL OF NEUROIMAGING. 2017;27(1):107-13.
362.	Bergsland N, Tavazzi E, Laganà MM, Baglio F, Cecconi P, Viotti S, et al. White Matter Tract Injury is Associated with Deep Gray Matter Iron Deposition in Multiple Sclerosis. Journal of Neuroimaging. 2017.
363.	Bergsland N, Tavazzi E, Lagana MM, Baglio F, Cecconi P, Viotti S, et al. White Matter Tract Injury is Associated with Deep Gray Matter Iron Deposition in Multiple Sclerosis. JOURNAL OF NEUROIMAGING. 2017;27(1):107-13.
364.	Bergsland N, Tavazzi E, Laganà MM, Baglio F, Cecconi P, Viotti S, et al. White Matter Tract Injury is Associated with Deep Gray Matter Iron Deposition in Multiple Sclerosis. J Neuroimaging. 2017;27(1):107-13.
365.	Bergsland N, Tavazzi E, Lagana MM, Baglio F, Cecconi P, Viotti S, et al. White Matter Tract Injury is Associated with Deep Gray Matter Iron Deposition in Multiple Sclerosis. JOURNAL OF NEUROIMAGING. 2017;27(1):107-13.
366.	Bergsland N, Tavazzi E, Laganà MM, Baglio F, Cecconi P, Viotti S, et al. White matter tract injury is associated with deep gray matter iron deposition in multiple sclerosis. Journal of Neuroimaging. 2017;27(1):107-13.
367.	Bergsland N, Tavazzi E, Schweser F, Jakimovski D, Hagemeier J, Dwyer MG, et al. Targeting Iron Dyshomeostasis for Treatment of Neurodegenerative Disorders. CNS DRUGS. 2019;33(11):1073-86.
368.	Bergsland N, Tavazzi E, Schweser F, Jakimovski D, Hagemeier J, Dwyer MG, et al. Targeting Iron Dyshomeostasis for Treatment of Neurodegenerative Disorders. CNS Drugs. 2019.
369.	Bergsland N, Tavazzi E, Schweser F, Jakimovski D, Hagemeier J, Dwyer MG, et al. Targeting Iron Dyshomeostasis for Treatment of Neurodegenerative Disorders. CNS Drugs. 2019;33(11):1073-86.
370.	Bergsland N, Tavazzi E, Schweser F, Jakimovski D, Hagemeier J, Dwyer MG, et al. Targeting Iron Dyshomeostasis for Treatment of Neurodegenerative Disorders. CNS DRUGS. 2019;33(11):1073-86.
371.	Berlow TA, Sammi MK, Pollaro J, Selzer AH, Bourdette D, Rooney WD. A NOVEL NON-INVASIVE MEASUREMENT OF IRON LEVELS IN HUMAN BRAIN USING MAGNETIC RESONANCE DATA ACQUISITION AND ITS VALIDATION AT 3T: A POTENTIAL CLINICAL BIOMARKER FOR MULTIPLE SCLEROSIS. MULTIPLE SCLEROSIS JOURNAL. 2012;18(9):S19-S.
372.	Berman SM, Walczak P, Bulte JW. MRI of transplanted neural stem cells. Methods Mol Biol. 2011;711:435-49.
373.	Berman SMC, Walczak P, Bulte JWM. MRI of transplanted neural stem cells. Methods in Molecular Biology2010.
374.	Berman SMC, Walczak P, Bulte JWM. MRI of Transplanted Neural Stem Cells. In: Modo M, Bulte JWM, editors. MAGNETIC RESONANCE NEUROIMAGING: METHODS AND PROTOCOLS. 7112011. p. 435-49.
375.	Bermel RA, Puli SR, Rudick RA, Weinstock-Guttman B, Fisher E, Munschauer FE, et al. Prediction of longitudinal brain atrophy in multiple sclerosis by gray matter magnetic resonance imaging T2 hypointensity. ARCHIVES OF NEUROLOGY. 2005;62(9):1371-6.
376.	Bermel RA, Puli SR, Rudick RA, Weinstock-Guttman B, Fisher E, Munschauer FE, 3rd, et al. Prediction of longitudinal brain atrophy in multiple sclerosis by gray matter magnetic resonance imaging T2 hypointensity. Arch Neurol. 2005;62(9):1371-6.
377.	Bermel RA, Puli SR, Rudick RA, Weinstock-Guttman B, Fisher E, Munschauer FE, et al. Prediction of longitudinal brain atrophy in multiple sclerosis by gray matter magnetic resonance imaging T2 hypointensity. ARCHIVES OF NEUROLOGY. 2005;62(9):1371-6.
378.	Bermel RA, Puli SR, Rudick RA, Weinstock-Guttman B, Fisher E, Munschauer Iii FE, et al. Prediction of longitudinal brain atrophy in multiple sclerosis by gray matter magnetic resonance imaging T2 hypointensity. Archives of Neurology. 2005.
379.	Bernabéu-Sanz Á, Morales S, Naranjo V, Sempere Á P. Contribution of Gray Matter Atrophy and White Matter Damage to Cognitive Impairment in Mildly Disabled Relapsing-Remitting Multiple Sclerosis Patients. Diagnostics (Basel). 2021;11(3).
380.	Bernd H, De Kerviler E, Gaillard S, Bonnemain B. Safety and Tolerability of Ultrasmall Superparamagnetic Iron Oxide Contrast Agent Comprehensive Analysis of a Clinical Development Program. INVESTIGATIVE RADIOLOGY. 2009;44(6):336-42.
381.	Bernd H, De Kerviler E, Gaillard S, Bonnemain B. Safety and tolerability of ultrasmall superparamagnetic iron oxide contrast agent: Comprehensive analysis of a clinical development program. Investigative Radiology. 2009.
382.	Bernd H, De Kerviler E, Gaillard S, Bonnemain B. Safety and tolerability of ultrasmall superparamagnetic iron oxide contrast agent: comprehensive analysis of a clinical development program. Invest Radiol. 2009;44(6):336-42.
383.	Bernd H, De Kerviler E, Gaillard S, Bonnemain B. Safety and Tolerability of Ultrasmall Superparamagnetic Iron Oxide Contrast Agent Comprehensive Analysis of a Clinical Development Program. INVESTIGATIVE RADIOLOGY. 2009;44(6):336-42.
384.	Bernheimer JH. Restless Legs Syndrome Presenting as an Acute Exacerbation of Multiple Sclerosis. MULTIPLE SCLEROSIS INTERNATIONAL. 2011;2011.
385.	Bernheimer JH. Restless legs syndrome presenting as an acute exacerbation of multiple sclerosis. Mult Scler Int. 2011;2011:872948.
386.	Bernheimer JH. Restless Legs Syndrome Presenting as an Acute Exacerbation of Multiple Sclerosis. MULTIPLE SCLEROSIS INTERNATIONAL. 2011;2011.
387.	Bernier R, Gavoille A, Chirpaz N, Jamilloux Y, Kodjikian L, Mathis T, et al. Diagnostic value of lumbar puncture for the etiological assessment of uveitis: a retrospective cohort of 188 patients. Graefes Arch Clin Exp Ophthalmol. 2022;260(5):1651-62.
388.	Berrih-Aknin S. Myasthenia Gravis: paradox versus paradigm in autoimmunity. J Autoimmun. 2014;52:1-28.
389.	Bertoni R, Mestanza Mattos FG, Porta M, Arippa F, Cocco E, Pau M, et al. Effects of immersive virtual reality on upper limb function in subjects with multiple sclerosis: A cross-over study. Mult Scler Relat Disord. 2022;65:104004.
390.	Bessa J, Meyer CA, Mudry M, Schlicht S, Smith SH, Iglesias A, et al. Altered subcellular localization of IL-33 leads to non-resolving lethal inflammation. JOURNAL OF AUTOIMMUNITY. 2014;55:33-41.
391.	Bettencourt A, Carvalho C, Leal B, Brás S, Lopes D, Martins da Silva A, et al. The Protective Role of HLA-DRB1(∗)13 in Autoimmune Diseases. J Immunol Res. 2015;2015:948723.
392.	Bettencourt A, Silva AM, Santos E, Gomes S, Mendonca D, Costa PP, et al. HFE gene polymorphisms and severity in Portuguese patients with multiple sclerosis. EUROPEAN JOURNAL OF NEUROLOGY. 2011;18(4):663-6.
393.	Bettencourt A, Silva AM, Santos E, Gomes S, Mendonça D, Costa PP, et al. HFE gene polymorphisms and severity in Portuguese patients with multiple sclerosis. European Journal of Neurology. 2011.
394.	Bettencourt A, Silva AM, Santos E, Gomes S, Mendonça D, Costa PP, et al. HFE gene polymorphisms and severity in Portuguese patients with multiple sclerosis. Eur J Neurol. 2011;18(4):663-6.
395.	Bettencourt A, Silva AM, Santos E, Gomes S, Mendonca D, Costa PP, et al. HFE gene polymorphisms and severity in Portuguese patients with multiple sclerosis. EUROPEAN JOURNAL OF NEUROLOGY. 2011;18(4):663-6.
396.	Bever CT, Jr., Jacobson S, Mingioli ES, McFarland HF, McFarlin DE, Levy HB. Changes in leukocyte recirculation, NK cell activity, and HLA-DR expression in peripheral blood mononuclear cells of MS patients treated with Poly ICLC. Int J Immunopharmacol. 1991;13(5):613-8.
397.	Bever CT, Jr., Panitch HS, Johnson KP. Increased cathepsin B activity in peripheral blood mononuclear cells of multiple sclerosis patients. Neurology. 1994;44(4):745-8.
398.	Bezuglova AM, Konenkova LP, Doronin BM, Buneva VN, Nevinsky GA. Affinity and catalytic heterogeneity and metal-dependence of polyclonal myelin basic protein-hydrolyzing IgGs from sera of patients with systemic lupus erythematosus. J Mol Recognit. 2011;24(6):960-74.
399.	Bhargava P, Mische L, Smith M, Nourbakhsh B, Reyes A, Martin K, et al. Bile Acid Metabolism is Altered in Multiple Sclerosis and Supplementation Leads to Amelioration of Neuroinflammation. NEUROLOGY. 2019;92(15).
400.	Bhat R, Mahapatra S, Axtell RC, Steinman L. Amelioration of ongoing experimental autoimmune encephalomyelitis with fluoxetine. J Neuroimmunol. 2017;313:77-81.
401.	Bian W, Harter K, Hammond-Rosenbluth KE, Lupo JM, Xu D, Kelley DA, et al. A serial in vivo 7T magnetic resonance phase imaging study of white matter lesions in multiple sclerosis. Mult Scler. 2013;19(1):69-75.
402.	Bian W, Harter K, Hammond-Rosenbluth KE, Lupo JM, Xu D, Kelley DAC, et al. A serial in vivo 7T magnetic resonance phase imaging study of white matter lesions in multiple sclerosis. MULTIPLE SCLEROSIS JOURNAL. 2013;19(1):69-75.
403.	Bian W, Harter K, Hammond-Rosenbluth KE, Lupo JM, Xu D, Kelley DAC, et al. A serial in vivo 7T magnetic resonance phase imaging study of white matter lesions in multiple sclerosis. Multiple Sclerosis Journal. 2013.
404.	Bian W, Harter K, Hammond-Rosenbluth KE, Lupo JM, Xu D, Kelley DAC, et al. A serial in vivo 7T magnetic resonance phase imaging study of white matter lesions in multiple sclerosis. MULTIPLE SCLEROSIS JOURNAL. 2013;19(1):69-75.
405.	Bian W, Tranvinh E, Tourdias T, Han M, Liu T, Wang Y, et al. In Vivo 7T MR quantitative susceptibility mapping reveals opposite susceptibility contrast between cortical and white matter lesions in multiple sclerosis. American Journal of Neuroradiology. 2016.
406.	Bian W, Tranvinh E, Tourdias T, Han M, Liu T, Wang Y, et al. In Vivo 7T MR Quantitative Susceptibility Mapping Reveals Opposite Susceptibility Contrast between Cortical and White Matter Lesions in Multiple Sclerosis. AJNR Am J Neuroradiol. 2016;37(10):1808-15.
407.	Bieger WP. Immunotoxicology of metals. Laboratory diagnosis of sensitization induced by mercury and dental metals. Klinisches Labor. 1996.
408.	Bigliardi G, Mandrioli J, Valzania F, Nichelli P, Casula N, Simone AM, et al. Primary progressive multiple sclerosis and generalized myasthenia gravis: an uncommon association. Neurol Sci. 2010;31(6):833-6.
409.	Bingöl EN, Taştekil I, Yay C, Keskin N, Ozbek P. How Epstein-Barr virus envelope glycoprotein gp350 tricks the CR2? A molecular dynamics study. J Mol Graph Model. 2022;114:108196.
410.	Binzer S, Hillert J, Manouchehrinia A. Concomitant autoimmunity and risk of multiple sclerosis disability worsening. Mult Scler Relat Disord. 2024;87:105637.
411.	Birkl C. Post-mortem Assessment of Iron in Multiple Sclerosis Brain using Magnetic Resonance Imaging. 2015.
412.	Birkl C, Carassiti D, Hussain F, Langkammer C, Enzinger C, Fazekas F, et al. Assessment of ferritin content in multiple sclerosis brains using temperature-induced R*(2) changes. MAGNETIC RESONANCE IN MEDICINE. 2018;79(3):1609-15.
413.	Birkl C, Carassiti D, Hussain F, Langkammer C, Enzinger C, Fazekas F, et al. Assessment of ferritin content in multiple sclerosis brains using temperature-induced R*2 changes. Magnetic Resonance in Medicine. 2018.
414.	Birkl C, Carassiti D, Hussain F, Langkammer C, Enzinger C, Fazekas F, et al. Assessment of ferritin content in multiple sclerosis brains using temperature-induced R*(2) changes. Magn Reson Med. 2018;79(3):1609-15.
415.	Birkl C, Carassiti D, Hussain F, Langkammer C, Enzinger C, Fazekas F, et al. Assessment of ferritin content in multiple sclerosis brains using temperature-induced R*(2) changes. MAGNETIC RESONANCE IN MEDICINE. 2018;79(3):1609-15.
416.	Birmingham Research Unit of the Royal College of General P. Lead and multiple sclerosis. The Journal of the Royal College of General Practitioners. 1976;26(169):622-6.
417.	Bisgård C. [The value of CT in disseminated sclerosis]. Ugeskr Laeger. 1987;149(44):2978-9.
418.	Bishnoi A, Holtzer R, Hernandez ME. Brain Activation Changes While Walking in Adults with and without Neurological Disease: Systematic Review and Meta-Analysis of Functional Near-Infrared Spectroscopy Studies. Brain Sci. 2021;11(3).
419.	Bitarafan S, Harirchian MH, Nafissi S, Sahraian MA, Togha M, Siassi F, et al. Dietary intake of nutrients and its correlation with fatigue in multiple sclerosis patients. IRANIAN JOURNAL OF NEUROLOGY. 2014;13(1):28-32.
420.	Bitarafan S, Harirchian MH, Nafissi S, Sahraian MA, Togha M, Siassi F, et al. Dietary intake of nutrients and its correlation with fatigue in multiple sclerosis patients. Iran J Neurol. 2014;13(1):28-32.
421.	Bitarafan S, Harirchian MH, Nafissi S, Sahraian MA, Togha M, Siassi F, et al. Dietary intake of nutrients and its correlation with fatigue in multiple sclerosis patients. IRANIAN JOURNAL OF NEUROLOGY. 2014;13(1):28-32.
422.	Bivins A, Hou K, Ayesu N, Ellsworth B, Jr., Montenegro S, Tu X, et al. Clinical evaluation of natalizumab for formulary consideration. Expert Opin Biol Ther. 2010;10(8):1279-87.
423.	Bizzozero OA, Ziegler JL, De Jesus G, Bolognani F. Acute depletion of reduced glutathione causes extensive carbonylation of rat brain proteins. JOURNAL OF NEUROSCIENCE RESEARCH. 2006;83(4):656-67.
424.	Bjørklund G, Hilt B, Dadar M, Lindh U, Aaseth J. Neurotoxic effects of mercury exposure in dental personnel. Basic Clin Pharmacol Toxicol. 2019;124(5):568-74.
425.	Bjørklund G, Peana M, Dadar M, Chirumbolo S, Aaseth J, Martins N. Mercury-induced autoimmunity: Drifting from micro to macro concerns on autoimmune disorders. Clin Immunol. 2020;213:108352.
426.	Bjørklund G, Zou L, Peana M, Chasapis CT, Hangan T, Lu J, et al. The Role of the Thioredoxin System in Brain Diseases. Antioxidants (Basel). 2022;11(11).
427.	Blanco Y, Escudero D, Lleixà C, Llufriu S, Egri N, García RR, et al. mRNA COVID-19 Vaccination Does Not Exacerbate Symptoms or Trigger Neural Antibody Responses in Multiple Sclerosis. Neurol Neuroimmunol Neuroinflamm. 2023;10(6).
428.	Blanco-Kelly F, Matesanz F, Alcina A, Teruel M, Díaz-Gallo LM, Gómez-García M, et al. CD40: novel association with Crohn's disease and replication in multiple sclerosis susceptibility. PLoS One. 2010;5(7):e11520.
429.	Blazejewska AI, Al-Radaideh AM, Wharton S, Lim SY, Bowtell RW, Constantinescu CS, et al. Increase in the iron content of the substantia nigra and red nucleus in multiple sclerosis and clinically isolated syndrome: A 7 Tesla MRI study. Journal of Magnetic Resonance Imaging. 2015.
430.	Blazejewska AI, Al-Radaideh AM, Wharton S, Lim SY, Bowtell RW, Constantinescu CS, et al. Increase in the Iron Content of the Substantia Nigra and Red Nucleus in Multiple Sclerosis and Clinically Isolated Syndrome: A 7 Tesla MRI Study. JOURNAL OF MAGNETIC RESONANCE IMAGING. 2015;41(4):1065-70.
431.	Blazejewska AI, Al-Radaideh AM, Wharton S, Lim SY, Bowtell RW, Constantinescu CS, et al. Increase in the iron content of the substantia nigra and red nucleus in multiple sclerosis and clinically isolated syndrome: a 7 Tesla MRI study. J Magn Reson Imaging. 2015;41(4):1065-70.
432.	Blazejewska AI, Al‐Radaideh AM, Wharton S, Lim SY, Bowtell RW, Constantinescu CS, et al. Increase in the iron content of the substantia nigra and red nucleus in multiple sclerosis and clinically isolated syndrome: a 7 Tesla MRI study. Journal of Magnetic Resonance Imaging. 2015;41(4):1065-70.
433.	Blezer EL, Deddens LH, Kooij G, Drexhage J, van der Pol SM, Reijerkerk A, et al. In vivo MR imaging of intercellular adhesion molecule-1 expression in an animal model of multiple sclerosis. Contrast Media Mol Imaging. 2015;10(2):111-21.
434.	Blezer ELA, Deddens LH, Kooij G, Drexhage J, van der Pol SMA, Reijerkerk A, et al. In vivo MR imaging of intercellular adhesion molecule-1 expression in an animal model of multiple sclerosis. CONTRAST MEDIA & MOLECULAR IMAGING. 2015;10(2):111-21.
435.	Blezer ELA, Deddens LH, Kooij G, Drexhage J, van der Pol SMA, Reijerkerk A, et al. In vivo MR imaging of intercellular adhesion molecule-1 expression in an animal model of multiple sclerosis. Contrast Media and Molecular Imaging. 2015.
436.	Blezer ELA, Deddens LH, Kooij G, Drexhage J, van der Pol SMA, Reijerkerk A, et al. In vivo MR imaging of intercellular adhesion molecule-1 expression in an animal model of multiple sclerosis. CONTRAST MEDIA & MOLECULAR IMAGING. 2015;10(2):111-21.
437.	Blinkenberg M, Akeson P, Sillesen H, Lövgaard S, Sellebjerg F, Paulson OB, et al. Chronic cerebrospinal venous insufficiency and venous stenoses in multiple sclerosis. Acta Neurol Scand. 2012;126(6):421-7.
438.	Bloem L. Iron and multiple sclerosis [PhD Thesis]. Stellenbosch, South Africa: University of Stellenbosch. 2007.
439.	Bo L, Vedeler CA, Nyland H, Trapp BD, Mork SJ. Intracortical multiple sclerosis lesions are not associated with increased lymphocyte infiltration. MULTIPLE SCLEROSIS. 2003;9(4):323-31.
440.	Bo L, Vedeler CA, Nyland H, Trapp BD, Mork SJ. Intracortical multiple sclerosis lesions are not associated with increased lymphocyte infiltration. MULTIPLE SCLEROSIS. 2003;9(4):323-31.
441.	Boaventura M, Sastre-Garriga J, Garcia-Vidal A, Vidal-Jordana A, Quartana D, Carvajal R, et al. T1/T2-weighted ratio in multiple sclerosis: A longitudinal study with clinical associations. NeuroImage: Clinical. 2022.
442.	Boaventura M, Sastre-Garriga J, Garcia-Vidal A, Vidal-Jordana A, Quartana D, Carvajal R, et al. T1/T2-weighted ratio in multiple sclerosis: A longitudinal study with clinical associations. Neuroimage Clin. 2022;34:102967.
443.	Bodil Roth E, Theander E, Londos E, Sandberg-Wollheim M, Larsson A, Sjöberg K, et al. Pathogenesis of autoimmune diseases: antibodies against transglutaminase, peptidylarginine deiminase and protein-bound citrulline in primary Sjögren's syndrome, multiple sclerosis and Alzheimer's disease. Scand J Immunol. 2008;67(6):626-31.
444.	Bodke S, Joshi N, Alavala RR, Suares D. In silico exploration of CB2 receptor agonist in the management of neuroinflammatory conditions by pharmacophore modeling. Comput Biol Chem. 2024;110:108049.
445.	Bollmann S, Kristensen MH, Larsen MS, Olsen MV, Pedersen MJ, Ostergaard LR, et al. SHARQnet - Sophisticated harmonic artifact reduction in quantitative susceptibility mapping using a deep convolutional neural network. ZEITSCHRIFT FUR MEDIZINISCHE PHYSIK. 2019;29(2):139-49.
446.	Bollmann S, Kristensen MH, Larsen MS, Olsen MV, Pedersen MJ, Ostergaard LR, et al. SHARQnet - Sophisticated harmonic artifact reduction in quantitative susceptibility mapping using a deep convolutional neural network. ZEITSCHRIFT FUR MEDIZINISCHE PHYSIK. 2019;29(2):139-49.
447.	Bollmann S, Kristensen MH, Larsen MS, Olsen MV, Pedersen MJ, Østergaard LR, et al. SHARQnet – Sophisticated harmonic artifact reduction in quantitative susceptibility mapping using a deep convolutional neural network. Zeitschrift fur Medizinische Physik. 2019.
448.	Bollmann S, Kristensen MH, Larsen MS, Olsen MV, Pedersen MJ, Østergaard LR, et al. SHARQnet - Sophisticated harmonic artifact reduction in quantitative susceptibility mapping using a deep convolutional neural network. Z Med Phys. 2019;29(2):139-49.
449.	Bollmann S, Rasmussen KGB, Kristensen M, Blendal RG, Ostergaard LR, Plocharski M, et al. DeepQSM - using deep learning to solve the dipole inversion for quantitative susceptibility mapping. NEUROIMAGE. 2019;195:373-83.
450.	Bollmann S, Rasmussen KGB, Kristensen M, Blendal RG, Østergaard LR, Plocharski M, et al. DeepQSM - using deep learning to solve the dipole inversion for quantitative susceptibility mapping. NeuroImage. 2019.
451.	Bollmann S, Rasmussen KGB, Kristensen M, Blendal RG, Østergaard LR, Plocharski M, et al. DeepQSM - using deep learning to solve the dipole inversion for quantitative susceptibility mapping. Neuroimage. 2019;195:373-83.
452.	Bollmann S, Rasmussen KGB, Kristensen M, Blendal RG, Ostergaard LR, Plocharski M, et al. DeepQSM - using deep learning to solve the dipole inversion for quantitative susceptibility mapping. NEUROIMAGE. 2019;195:373-83.
453.	Bolviken B, Celius EG, Nilsen R, Strand T. Radon: A possible risk factor in multiple sclerosis. NEUROEPIDEMIOLOGY. 2003;22(1):87-94.
454.	Bølviken B, Celius EG, Nilsen R, Strand T. Radon: A possible risk factor in multiple sclerosis. Neuroepidemiology. 2003.
455.	Bølviken B, Celius EG, Nilsen R, Strand T. Radon: a possible risk factor in multiple sclerosis. Neuroepidemiology. 2003;22(1):87-94.
456.	Bolviken B, Celius EG, Nilsen R, Strand T. Radon: A possible risk factor in multiple sclerosis. NEUROEPIDEMIOLOGY. 2003;22(1):87-94.
457.	Bongioanni MR, Durelli L, Ferrero B, Imperiale D, Oggero A, Verdun E, et al. Systemic high-dose recombinant-alpha-2a-interferon therapy modulates lymphokine production in multiple sclerosis. JOURNAL OF THE NEUROLOGICAL SCIENCES. 1996;143(1-2):91-9.
458.	Bongioanni MR, Durelli L, Ferrero B, Imperiale D, Oggero A, Verdun E, et al. Systemic high-dose recombinant-alpha-2a-interferon therapy modulates lymphokine production in multiple sclerosis. J Neurol Sci. 1996;143(1-2):91-9.
459.	Bongioanni MR, Durelli L, Ferrero B, Imperiale D, Oggero A, Verdun E, et al. Systemic high-dose recombinant-alpha-2a-interferon therapy modulates lymphokine production in multiple sclerosis. JOURNAL OF THE NEUROLOGICAL SCIENCES. 1996;143(1-2):91-9.
460.	Bonnechère B. Integrating Rehabilomics into the Multi-Omics Approach in the Management of Multiple Sclerosis: The Way for Precision Medicine? Genes (Basel). 2022;14(1).
461.	Bonnemain B. Nanoparticles: The industrial viewpoint. Applications in diagnostic imaging. Annales Pharmaceutiques Francaises. 2008.
462.	Bonnemain B. [Nanoparticles: the industrial viewpoint. Applications in diagnostic imaging]. Ann Pharm Fr. 2008;66(5-6):263-7.
463.	Bonnier G, Fischi-Gomez E, Roche A, Hilbert T, Kober T, Krueger G, et al. Personalized pathology maps to quantify diffuse and focal brain damage. NEUROIMAGE-CLINICAL. 2019;21.
464.	Bonnier G, Fischi-Gomez E, Roche A, Hilbert T, Kober T, Krueger G, et al. Personalized pathology maps to quantify diffuse and focal brain damage. NeuroImage: Clinical. 2019.
465.	Bonnier G, Fischi-Gomez E, Roche A, Hilbert T, Kober T, Krueger G, et al. Personalized pathology maps to quantify diffuse and focal brain damage. Neuroimage Clin. 2019;21:101607.
466.	Bonnier G, Fischi-Gomez E, Roche A, Hilbert T, Kober T, Krueger G, et al. Personalized pathology maps to quantify diffuse and focal brain damage. NEUROIMAGE-CLINICAL. 2019;21.
467.	Bonnier G, Maréchal B, Fartaria MJ, Falkowskiy P, Marques JP, Simioni S, et al. The Combined Quantification and Interpretation of Multiple Quantitative Magnetic Resonance Imaging Metrics Enlightens Longitudinal Changes Compatible with Brain Repair in Relapsing-Remitting Multiple Sclerosis Patients. Front Neurol. 2017;8:506.
468.	Bonnier G, Maréchal B, Fartaria MJ, Falkowskiy P, Marques JP, Simioni S, et al. The combined quantification and interpretation of multiple quantitative magnetic resonance imaging metrics enlightens longitudinal changes compatible with brain repair in relapsing-remitting multiple sclerosis patients. Frontiers in Neurology. 2017.
469.	Booth DR, Arthur AT, Teutsch SM, Bye C, Rubio J, Armati PJ, et al. Gene expression and genotyping studies implicate the interleukin 7 receptor in the pathogenesis of primary progressive multiple sclerosis. J Mol Med (Berl). 2005;83(10):822-30.
470.	Borkakoti N. Matrix metalloproteases: variations on a theme. PROGRESS IN BIOPHYSICS & MOLECULAR BIOLOGY. 1998;70(1):73-94.
471.	Borkakoti N. Matrix metalloproteases: Variations on a theme. Progress in Biophysics and Molecular Biology. 1998.
472.	Borkakoti N. Matrix metalloproteases: variations on a theme. Prog Biophys Mol Biol. 1998;70(1):73-94.
473.	Borkakoti N. Matrix metalloprotease inhibitors: design from structure. BIOCHEMICAL SOCIETY TRANSACTIONS. 2004;32:17-20.
474.	Borkakoti N. Matrix metalloprotease inhibitors: Design from structure. Biochemical Society Transactions. 2004.
475.	Borkakoti N. Matrix metalloprotease inhibitors: design from structure. Biochem Soc Trans. 2004;32(Pt 1):17-20.
476.	Borkakoti N. Matrix metalloprotease inhibitors: design from structure. BIOCHEMICAL SOCIETY TRANSACTIONS. 2004;32:17-20.
477.	Borreani C, Giordano A, Falautano M, Lugaresi A, Martinelli V, Granella F, et al. Experience of an information aid for newly diagnosed multiple sclerosis patients: a qualitative study on the SIMS-Trial. Health Expect. 2014;17(1):36-48.
478.	Börü Ü T, Bilgiç AB, Köseoğlu Toksoy C, Yılmaz AY, Tasdemir M, Sensöz NP, et al. Prevalence of Multiple Sclerosis in a Turkish City Bordering an Iron and Steel Factory. J Clin Neurol. 2018;14(2):234-41.
479.	Boru UT, Bilgic AB, Toksoy CK, Yilmaz AY, Tasdemir M, Sensoz NP, et al. Prevalence of Multiple Sclerosis in a Turkish City Bordering an Iron and Steel Factory. JOURNAL OF CLINICAL NEUROLOGY. 2018;14(2):234-41.
480.	Boru UT, Bilgic AB, Toksoy CK, Yilmaz AY, Tasdemir M, Sensoz NP, et al. Prevalence of Multiple Sclerosis in a Turkish City Bordering an Iron and Steel Factory. JOURNAL OF CLINICAL NEUROLOGY. 2018;14(2):234-41.
481.	Boru UT, Bilgic AB, Toksoy CK, Yilmaz AY, Tasdemir M, Sensoz NP, et al. Prevalence of Multiple Sclerosis in a Turkish City Bordering an Iron and Steel Factory. JOURNAL OF CLINICAL NEUROLOGY. 2018;14(2):234-41.
482.	Börü ÜT, Bilgiç AB, Toksoy CK, Yılmaz AY, Tasdemir M, Sensöz NP, et al. Prevalence of multiple sclerosis in a Turkish city bordering an iron and steel factory. Journal of Clinical Neurology. 2018;14(2):234-41.
483.	Börü ÜT, Bilgiç AB, Toksoy CK, Yılmaz AY, Tasdemir M, Sensöz NP, et al. Prevalence of multiple sclerosis in a Turkish city bordering an iron and steel factory. Journal of Clinical Neurology (Korea). 2018.
484.	Boru UT, Boluk C, Tasdemir M, Gezer T, Serim VA. Air pollution, a possible risk factor for multiple sclerosis. ACTA NEUROLOGICA SCANDINAVICA. 2020;141(5):431-7.
485.	Boshes B. Possible Relation of Lead Intoxication to Multiple Sclerosis. The Journal of Nervous and Mental Disease. 1936;83(5):602.
486.	Boski N, Gulati V, Raj R, Gulati P. Multiple Sclerosis-Minimizing Errors in Radiological Diagnosis. Neurol India. 2021;69(6):1539-46.
487.	Bostrom I, Landtblom AM, Lauer K. An ecological study of industry in a high-risk region of multiple sclerosis. JOURNAL OF THE NEUROLOGICAL SCIENCES. 2011;311(1-2):50-7.
488.	Boström I, Landtblom AM, Lauer K. An ecological study of industry in a high-risk region of multiple sclerosis. Journal of the Neurological Sciences. 2011.
489.	Boström I, Landtblom AM, Lauer K. An ecological study of industry in a high-risk region of multiple sclerosis. J Neurol Sci. 2011;311(1-2):50-7.
490.	Bostrom I, Landtblom AM, Lauer K. An ecological study of industry in a high-risk region of multiple sclerosis. JOURNAL OF THE NEUROLOGICAL SCIENCES. 2011;311(1-2):50-7.
491.	Botha J, Potocnik FC, Matsha T, Erasmus RT. Iron and the folate-vitamin B12-methylation pathway in multiple sclerosis. Metab Brain Dis. 2006;21:121137van.
492.	Boullerne AI, Nedelkoska L, Benjamins JA. Synergism of nitric oxide and iron in killing the transformed murine oligodendrocyte cell line N20.1. JOURNAL OF NEUROCHEMISTRY. 1999;72(3):1050-60.
493.	Boullerne AI, Nedelkoska L, Benjamins JA. Synergism of nitric oxide and iron in killing the transformed murine oligodendrocyte cell line N20.1. Journal of Neurochemistry. 1999.
494.	Boullerne AI, Nedelkoska L, Benjamins JA. Synergism of nitric oxide and iron in killing the transformed murine oligodendrocyte cell line N20.1. J Neurochem. 1999;72(3):1050-60.
495.	Boullerne AI, Nedelkoska L, Benjamins JA. Synergism of nitric oxide and iron in killing the transformed murine oligodendrocyte cell line N20.1. JOURNAL OF NEUROCHEMISTRY. 1999;72(3):1050-60.
496.	Boullerne AI, Nedelkoska L, Benjamins JA. Role of calcium in nitric oxide-induced cytotoxicity: EGTA protects mouse oligodendrocytes. JOURNAL OF NEUROSCIENCE RESEARCH. 2001;63(2):124-35.
497.	Boullerne AI, Nedelkoska L, Benjamins JA. Role of calcium in nitric oxide-induced cytotoxicity: EGTA protects mouse oligodendrocytes. Journal of Neuroscience Research. 2001.
498.	Boullerne AI, Nedelkoska L, Benjamins JA. Role of calcium in nitric oxide-induced cytotoxicity: EGTA protects mouse oligodendrocytes. J Neurosci Res. 2001;63(2):124-35.
499.	Boullerne AI, Nedelkoska L, Benjamins JA. Role of calcium in nitric oxide-induced cytotoxicity: EGTA protects mouse oligodendrocytes. JOURNAL OF NEUROSCIENCE RESEARCH. 2001;63(2):124-35.
500.	Bouton C, Bhagat N, Chandrasekaran S, Herrero J, Markowitz N, Espinal E, et al. Decoding Neural Activity in Sulcal and White Matter Areas of the Brain to Accurately Predict Individual Finger Movement and Tactile Stimuli of the Human Hand. Front Neurosci. 2021;15:699631.
501.	Bowern N, Ramshaw IA, Clark IA, Doherty PC. Inhibition of autoimmune neuropathological process by treatment with an iron-chelating agent. J Exp Med. 1984;160(5):1532-43.
502.	Boyko OB, Alston SR, Fuller GN, Hulette CM, Johnson GA, Burger PC. Utility of postmortem magnetic resonance imaging in clinical neuropathology. Arch Pathol Lab Med. 1994;118(3):219-25.
503.	Boyle AJ, Lindberg A, Tong J, Zhai D, Liu F, Vasdev N. Preliminary PET imaging of [(11)C]evobrutinib in mouse models of colorectal cancer, SARS-CoV-2, and lung damage: Radiosynthesis via base-aided palladium-NiXantphos-mediated (11)C-carbonylation. J Labelled Comp Radiopharm. 2024;67(6):235-44.
504.	Boziki M, Grigoriadis N. An Update on the Role of Matrix Metalloproteinases in the Pathogenesis of Multiple Sclerosis. MEDICINAL CHEMISTRY. 2018;14(2):155-69.
505.	Boziki M, Grigoriadis N. An update on the role of matrix metalloproteinases in the pathogenesis of multiple sclerosis. Medicinal Chemistry. 2018.
506.	Boziki M, Grigoriadis N. An Update on the Role of Matrix Metalloproteinases in the Pathogenesis of Multiple Sclerosis. Med Chem. 2018;14(2):155-69.
507.	Braakhuis HEM, Berger MAM, van der Stok GA, van Meeteren J, de Groot V, Beckerman H, et al. Three distinct physical behavior types in fatigued patients with multiple sclerosis. J Neuroeng Rehabil. 2019;16(1):105.
508.	Braakhuis HEM, Berger MAM, van der Stok GA, van Meeteren J, de Groot V, Beckerman H, et al. Three distinct physical behavior types in fatigued patients with multiple sclerosis. JOURNAL OF NEUROENGINEERING AND REHABILITATION. 2019;16(1).
509.	Braakhuis HEM, Berger MAM, van der Stok GA, van Meeteren J, de Groot V, Beckerman H, et al. Three distinct physical behavior types in fatigued patients with multiple sclerosis. JOURNAL OF NEUROENGINEERING AND REHABILITATION. 2019;16(1).
510.	Braenne I, Zeng LY, Willenborg C, Tragante V, Kessler T, Willer CJ, et al. Genomic correlates of glatiramer acetate adverse cardiovascular effects lead to a novel locus mediating coronary risk. PLOS ONE. 2017;12(8).
511.	Braenne I, Zeng LY, Willenborg C, Tragante V, Kessler T, Willer CJ, et al. Genomic correlates of glatiramer acetate adverse cardiovascular effects lead to a novel locus mediating coronary risk. PLOS ONE. 2017;12(8).
512.	Brahic M, Bureau JF, Michiels T. The genetics of the persistent infection and demyelinating disease caused by Theiler's virus.  ANNUAL REVIEW OF MICROBIOLOGY. 592005. p. 279-98.
513.	Brahic M, Bureau JF, Michiels T. The genetics of the persistent infection and demyelinating disease caused by Theiler's virus. Annual Review of Microbiology2005.
514.	Brahic M, Bureau JF, Michiels T. The genetics of the persistent infection and demyelinating disease caused by Theiler's virus. Annu Rev Microbiol. 2005;59:279-98.
515.	Braid BS. Multiple Sclerosis and heavy metals:(a case history). Townsend Letter: The Examiner of Alternative Medicine. 2007(287):125-7.
516.	Branca JJV, Morucci G, Pacini A. Cadmium-induced neurotoxicity: still much ado. NEURAL REGENERATION RESEARCH. 2018;13(11):1879-82.
517.	Branca JJV, Morucci G, Pacini A. Cadmium-induced neurotoxicity: Still much ado. Neural Regeneration Research. 2018.
518.	Branca JJV, Morucci G, Pacini A. Cadmium-induced neurotoxicity: still much ado. Neural Regen Res. 2018;13(11):1879-82.
519.	Brass SD, Benedict RH, Weinstock-Guttman B, Munschauer F, Bakshi R. Cognitive impairment is associated with subcortical magnetic resonance imaging grey matter T2 hypointensity in multiple sclerosis. Mult Scler. 2006;12(4):437-44.
520.	Brass SD, Benedict RHB, Weinstock-Guttman B, Munschauer F, Bakshi R. Cognitive impairment is associated with subcortical magnetic resonance imaging grey matter T2 hypointensity in multiple sclerosis. MULTIPLE SCLEROSIS JOURNAL. 2006;12(4):437-44.
521.	Brass SD, Benedict RHB, Weinstock-Guttman B, Munschauer F, Bakshi R. Cognitive impairment is associated with subcortical magnetic resonance imaging grey matter T2 hypointensity in multiple sclerosis. Multiple Sclerosis. 2006.
522.	Brass SD, Benedict RHB, Weinstock-Guttman B, Munschauer F, Bakshi R. Cognitive impairment is associated with subcortical magnetic resonance imaging grey matter T2 hypointensity in multiple sclerosis. MULTIPLE SCLEROSIS JOURNAL. 2006;12(4):437-44.
523.	Brass SD, Chen NK, Mulkern RV, Bakshi R. Magnetic resonance imaging of iron deposition in neurological disorders. Topics in Magnetic Resonance Imaging. 2006.
524.	Brass SD, Chen NK, Mulkern RV, Bakshi R. Magnetic resonance imaging of iron deposition in neurological disorders. Top Magn Reson Imaging. 2006;17(1):31-40.
525.	Bredholt M, Frederiksen JL. Zinc in Multiple Sclerosis: A Systematic Review and Meta-Analysis. ASN NEURO. 2016;8(3).
526.	Bredholt M, Frederiksen JL. Zinc in multiple sclerosis: A systematic review and meta-analysis. ASN Neuro. 2016.
527.	Bredholt M, Frederiksen JL. Zinc in Multiple Sclerosis: A Systematic Review and Meta-Analysis. ASN NEURO. 2016;8(3).
528.	Bredholt M, Frederiksen JL. Zinc in multiple sclerosis - a systematic review and meta-analysis. EUROPEAN JOURNAL OF NEUROLOGY. 2016;23:311-.
529.	Bredholt M, Frederiksen JL. Zinc in Multiple Sclerosis: A Systematic Review and Meta-Analysis. ASN Neuro. 2016;8(3).
530.	Bredholt M, Frederiksen JL. Zinc in multiple sclerosis: A systematic review and meta-analysis. ASN neuro. 2016;8(3):1759091416651511.
531.	Breijyeh Z, Jubeh B, Bufo SA, Karaman R, Scrano L. Cannabis: A Toxin-Producing Plant with Potential Therapeutic Uses. Toxins (Basel). 2021;13(2).
532.	Bremm RP, Berthold C, Krüger R, Koch KP, Gonçalves J, Hertel F. Therapeutic maps for a sensor-based evaluation of deep brain stimulation programming. Biomed Tech (Berl). 2021;66(6):603-11.
533.	Brenner T, Nizri E, Irony-Tur-Sinai M, Hamra-Amitay Y, Wirguin I. Acetylcholinesterase inhibitors and cholinergic modulation in Myasthenia Gravis and neuroinflammation. J Neuroimmunol. 2008;201-202:121-7.
534.	Brenu EW, Broadley S, Nguyen T, Johnston S, Ramos S, Staines D, et al. A Preliminary Comparative Assessment of the Role of CD8+ T Cells in Chronic Fatigue Syndrome/Myalgic Encephalomyelitis and Multiple Sclerosis. J Immunol Res. 2016;2016:9064529.
535.	Breyer U, Kanig K. Cerebrospinal fluid electrolyte disturbances in neurological disorders: with special reference to inorganic phosphate. Neurology. 1970;20(3):247-53.
536.	Brinkman CJ, Nillesen WM, Hommes OR. T-cell subpopulations in blood and cerebrospinal fluid of multiple sclerosis patients: effect of cyclophosphamide. Clin Immunol Immunopathol. 1983;29(3):341-8.
537.	Brkic M, Balusu S, Libert C, Vandenbroucke RE. Friends or Foes: Matrix Metalloproteinases and Their Multifaceted Roles in Neurodegenerative Diseases. MEDIATORS OF INFLAMMATION. 2015;2015.
538.	Brkic M, Balusu S, Libert C, Vandenbroucke RE. Friends or Foes: Matrix Metalloproteinases and Their Multifaceted Roles in Neurodegenerative Diseases. Mediators of Inflammation. 2015.
539.	Brkic M, Balusu S, Libert C, Vandenbroucke RE. Friends or Foes: Matrix Metalloproteinases and Their Multifaceted Roles in Neurodegenerative Diseases. Mediators Inflamm. 2015;2015:620581.
540.	Broche-Pérez Y, Jiménez-Morales RM, Monasterio-Ramos LO, Bauer J. Validity and reliability of the 10-item Connor-Davidson Resilience Scale (CD-RISC-10) in a sample of Spanish-speaking patients with Multiple Sclerosis. Mult Scler Relat Disord. 2022;63:103914.
541.	Brochet B, Deloire MS, Touil T, Anne O, Caillé JM, Dousset V, et al. Early macrophage MRI of inflammatory lesions predicts lesion severity and disease development in relapsing EAE. Neuroimage. 2006;32(1):266-74.
542.	Brochet B, Deloire MSA, Touil T, Anne O, Caille JM, Dousset V, et al. Early macrophage MRI of inflammatory lesions predicts lesion severity and disease development in relapsing EAE. NEUROIMAGE. 2006;32(1):266-74.
543.	Brochet B, Deloire MSA, Touil T, Anne O, Caillé JM, Dousset V, et al. Early macrophage MRI of inflammatory lesions predicts lesion severity and disease development in relapsing EAE. NeuroImage. 2006.
544.	Brochet B, Deloire MSA, Touil T, Anne O, Caille JM, Dousset V, et al. Early macrophage MRI of inflammatory lesions predicts lesion severity and disease development in relapsing EAE. NEUROIMAGE. 2006;32(1):266-74.
545.	Brod SA. A proposal: How to study pro-myelinating proteins in MS. Autoimmun Rev. 2022;21(1):102924.
546.	Brod SA. A proposal: How to study pro-myelinating proteins in MS. Autoimmun Rev. 2022;21(1):102924.
547.	Bromley L, Horvath PJ, Bennett SE, Weinstock-Guttman B, Ray AD. Impact of nutritional intake on function in people with mild-to-moderate multiple sclerosis. International Journal of MS Care. 2019.
548.	Bromley L, Horvath PJ, Bennett SE, Weinstock-Guttman B, Ray AD. Impact of Nutritional Intake on Function in People with Mild-to-Moderate Multiple Sclerosis. Int J MS Care. 2019;21(1):1-9.
549.	Brooks DJ, Beaney RP, Lammertsma AA, Leenders KL, Horlock PL, Kensett MJ, et al. Quantitative measurement of blood-brain barrier permeability using rubidium-82 and positron emission tomography. J Cereb Blood Flow Metab. 1984;4(4):535-45.
550.	Brown D, Moezzi D, Dong Y, Koch M, Yong VW. Combination of Hydroxychloroquine and Indapamide Attenuates Neurodegeneration in Models Relevant to Multiple Sclerosis. Neurotherapeutics. 2021.
551.	Brown D, Moezzi D, Dong Y, Koch M, Yong VW. Combination of Hydroxychloroquine and Indapamide Attenuates Neurodegeneration in Models Relevant to Multiple Sclerosis. Neurotherapeutics. 2021;18(1):387-400.
552.	Brown D, Moezzi D, Dong YF, Koch M, Yong VW. Combination of Hydroxychloroquine and Indapamide Attenuates Neurodegeneration in Models Relevant to Multiple Sclerosis. NEUROTHERAPEUTICS. 2021;18(1):387-400.
553.	Brown D, Moezzi D, Dong YF, Koch M, Yong VW. Combination of Hydroxychloroquine and Indapamide Attenuates Neurodegeneration in Models Relevant to Multiple Sclerosis. NEUROTHERAPEUTICS. 2021;18(1):387-400.
554.	Brown JS, Jr. Correlation of mollicutes and their viruses with multiple sclerosis and other demyelinating diseases. Med Hypotheses. 2003;60(2):298-303.
555.	Brown RB. Sodium Toxicity in the Nutritional Epidemiology and Nutritional Immunology of COVID-19. Medicina (Kaunas). 2021;57(8).
556.	Brown RB. Non-Specific Low Back Pain, Dietary Salt Intake, and Posterior Lumbar Subcutaneous Edema. Int J Environ Res Public Health. 2022;19(15).
557.	Broza YY, Har-Shai L, Jeries R, Cancilla JC, Glass-Marmor L, Lejbkowicz I, et al. Exhaled Breath Markers for Nonimaging and Noninvasive Measures for Detection of Multiple Sclerosis. ACS Chem Neurosci. 2017;8(11):2402-13.
558.	Brüggemann F, Gross S, Süße M, Hok P, Strauss S, Ziemssen T, et al. Polypharmacy in patients with multiple sclerosis and the impact on levels of care and therapy units. Front Neurol. 2023;14:1330066.
559.	Bruijstens AL, Molenaar S, Wong YYM, Kraaij R, Neuteboom RF. Gut microbiota analysis in pediatric-onset multiple sclerosis compared to pediatric monophasic demyelinating syndromes and pediatric controls. Eur J Neurol. 2023;30(11):3507-15.
560.	Brunner E, Vandemeulebroecke M, Mütze T. Win odds: An adaptation of the win ratio to include ties. Stat Med. 2021;40(14):3367-84.
561.	Brunner W. PRURITUS - ALSO A CHALLENGE IN INTERNAL-MEDICINE. SCHWEIZERISCHE MEDIZINISCHE WOCHENSCHRIFT. 1995;125(46):2244-50.
562.	Brunner W. Pruritus - Also a challenge in internal medicine. Schweizerische Medizinische Wochenschrift. 1995.
563.	Brunner W. [Pruritus--also a challenge in internal medicine]. Schweiz Med Wochenschr. 1995;125(46):2244-50.
564.	Brunner W. PRURITUS - ALSO A CHALLENGE IN INTERNAL-MEDICINE. SCHWEIZERISCHE MEDIZINISCHE WOCHENSCHRIFT. 1995;125(46):2244-50.
565.	Bsteh G, Haschka D, Tymoszuk P, Berek K, Petzer V, Hegen H, et al. Serum hepcidin levels in multiple sclerosis. MULTIPLE SCLEROSIS JOURNAL-EXPERIMENTAL TRANSLATIONAL AND CLINICAL. 2019;5(4).
566.	Bsteh G, Haschka D, Tymoszuk P, Berek K, Petzer V, Hegen H, et al. Serum hepcidin levels in multiple sclerosis. MULTIPLE SCLEROSIS JOURNAL-EXPERIMENTAL TRANSLATIONAL AND CLINICAL. 2019;5(4).
567.	Bsteh G, Haschka D, Tymoszuk P, Berek K, Petzer V, Hegen H, et al. Serum hepcidin levels in multiple sclerosis. Multiple Sclerosis Journal - Experimental, Translational and Clinical. 2019.
568.	Bsteh G, Haschka D, Tymoszuk P, Berek K, Petzer V, Hegen H, et al. Serum hepcidin levels in multiple sclerosis. Mult Scler J Exp Transl Clin. 2019;5(4):2055217319885984.
569.	Buch S, Subramanian K, Jella PK, Chen Y, Wu Z, Shah K, et al. Revealing vascular abnormalities and measuring small vessel density in multiple sclerosis lesions using USPIO. NeuroImage: Clinical. 2021.
570.	Buch S, Subramanian K, Jella PK, Chen Y, Wu Z, Shah K, et al. Revealing vascular abnormalities and measuring small vessel density in multiple sclerosis lesions using USPIO. Neuroimage Clin. 2021;29:102525.
571.	Buch S, Subramanian K, Jella PK, Chen YS, Wu Z, Shah K, et al. Revealing vascular abnormalities and measuring small vessel density in multiple sclerosis lesions using USPIO. NEUROIMAGE-CLINICAL. 2021;29.
572.	Buch S, Subramanian K, Jella PK, Chen YS, Wu Z, Shah K, et al. Revealing vascular abnormalities and measuring small vessel density in multiple sclerosis lesions using USPIO. NEUROIMAGE-CLINICAL. 2021;29.
573.	Bui B, Byun J, Jacobs J, Liu AK. Multiple Sclerosis in a Patient With Prior West Nile Encephalitis. Cureus. 2022;14(9):e28935.
574.	Bulk M, van Harten T, Kenkhuis B, Inglese F, Hegeman I, van Duinen S, et al. Quantitative susceptibility mapping in the thalamus and basal ganglia of systemic lupus erythematosus patients with neuropsychiatric complaints. NEUROIMAGE-CLINICAL. 2021;30.
575.	Bulk M, van Harten T, Kenkhuis B, Inglese F, Hegeman I, van Duinen S, et al. Quantitative susceptibility mapping in the thalamus and basal ganglia of systemic lupus erythematosus patients with neuropsychiatric complaints. NeuroImage: Clinical. 2021.
576.	Bulk M, van Harten T, Kenkhuis B, Inglese F, Hegeman I, van Duinen S, et al. Quantitative susceptibility mapping in the thalamus and basal ganglia of systemic lupus erythematosus patients with neuropsychiatric complaints. Neuroimage Clin. 2021;30:102637.
577.	Bulk M, van Harten T, Kenkhuis B, Inglese F, Hegeman I, van Duinen S, et al. Quantitative susceptibility mapping in the thalamus and basal ganglia of systemic lupus erythematosus patients with neuropsychiatric complaints. NEUROIMAGE-CLINICAL. 2021;30.
578.	Bumb A, Brechbiel MW, Choyke P, Fugger L, Dobson PJ, editors. Nanomedicine: Engineering of a tri-imageable nanoparticle2007.
579.	Buonomo AR, Zappulo E, Viceconte G, Scotto R, Borgia G, Gentile I. Risk of opportunistic infections in patients treated with alemtuzumab for multiple sclerosis. Expert Opin Drug Saf. 2018;17(7):709-17.
580.	Burchiel KJ. Ectopic impulse generation in demyelinated axons: effects of PaCO2, pH, and disodium edetate. Ann Neurol. 1981;9(4):378-83.
581.	Burg N, Salmon JE, Hla T. Sphingosine 1-phosphate receptor-targeted therapeutics in rheumatic diseases. Nat Rev Rheumatol. 2022;18(6):335-51.
582.	Burgetova A, Dusek P, Pudlac A, Nytrova P, Vaneckova M, Horakova D, et al., editors. Deep grey matter iron content in neuromyelitis optica and multiple sclerosis2019: SAGE PUBLICATIONS LTD 1 OLIVERS YARD, 55 CITY ROAD, LONDON EC1Y 1SP, ENGLAND.
583.	Burgetova A, Dusek P, Pudlac A, Nytrova P, Vaneckova M, Horakova D, et al. Deep grey matter iron content in neuromyelitis optica and multiple sclerosis. MULTIPLE SCLEROSIS JOURNAL. 2019;25:705-6.
584.	Burgetova A, Dusek P, Uher T, Vaneckova M, Vejrazka M, Burgetova R, et al. CSF Markers of Oxidative Stress Are Associated with Brain Atrophy and Iron Accumulation in a 2-Year Longitudinal Cohort of Early MS. Int J Mol Sci. 2023;24(12).
585.	Burgetova A, Dusek P, Uher T, Vaneckova M, Vejrazka M, Burgetova R, et al. Oxidative Stress Markers in Cerebrospinal Fluid of Newly Diagnosed Multiple Sclerosis Patients and Their Link to Iron Deposition and Atrophy. Diagnostics (Basel). 2022;12(6).
586.	Burgetova A, Dusek P, Vaneckova M, Horakova D, Langkammer C, Krasensky J, et al. Thalamic Iron Differentiates Primary-Progressive and Relapsing-Remitting Multiple Sclerosis. AMERICAN JOURNAL OF NEURORADIOLOGY. 2017;38(6):1079-86.
587.	Burgetova A, Dusek P, Vaneckova M, Horakova D, Langkammer C, Krasensky J, et al. Thalamic iron differentiates primary-progressive and relapsing-remitting multiple sclerosis. American Journal of Neuroradiology. 2017.
588.	Burgetova A, Dusek P, Vaneckova M, Horakova D, Langkammer C, Krasensky J, et al. Thalamic Iron Differentiates Primary-Progressive and Relapsing-Remitting Multiple Sclerosis. AMERICAN JOURNAL OF NEURORADIOLOGY. 2017;38(6):1079-86.
589.	Burgetova A, Dusek P, Vaneckova M, Horakova D, Langkammer C, Krasensky J, et al. Thalamic Iron Differentiates Primary-Progressive and Relapsing-Remitting Multiple Sclerosis. AJNR Am J Neuroradiol. 2017;38(6):1079-86.
590.	Burgetova A, Dusek P, Vaneckova M, Horakova D, Langkammer C, Krasensky J, et al. Thalamic Iron Differentiates Primary-Progressive and Relapsing-Remitting Multiple Sclerosis. AMERICAN JOURNAL OF NEURORADIOLOGY. 2017;38(6):1079-86.
591.	Burgetova A, Dusek P, Vaneckova M, Horakova D, Langkammer C, Krasensky J, et al. Thalamic iron differentiates primary-progressive and relapsing-remitting multiple sclerosis. American Journal of Neuroradiology. 2017;38(6):1079-86.
592.	Burgetova A, Seidl Z, Krasensky J, Horakova D, Vaneckova M. Multiple Sclerosis and the Accumulation of Iron in the Basal Ganglia: Quantitative Assessment of Brain Iron Using MRI T-2 Relaxometry. EUROPEAN NEUROLOGY. 2010;63(3):136-43.
593.	Burgetova A, Seidl Z, Krasensky J, Horakova D, Vaneckova M. Multiple sclerosis and the accumulation of iron in the basal ganglia: Quantitative assessment of brain iron using MRI T2 relaxometry. European Neurology. 2010.
594.	Burgetova A, Seidl Z, Krasensky J, Horakova D, Vaneckova M. Multiple Sclerosis and the Accumulation of Iron in the Basal Ganglia: Quantitative Assessment of Brain Iron Using MRI T-2 Relaxometry. EUROPEAN NEUROLOGY. 2010;63(3):136-43.
595.	Burgetova A, Seidl Z, Krasensky J, Horakova D, Vaneckova M. Multiple sclerosis and the accumulation of iron in the Basal Ganglia: quantitative assessment of brain iron using MRI t(2) relaxometry. Eur Neurol. 2010;63(3):136-43.
596.	Burgetova A, Seidl Z, Krasensky J, Horakova D, Vaneckova M. Multiple Sclerosis and the Accumulation of Iron in the Basal Ganglia: Quantitative Assessment of Brain Iron Using MRI T-2 Relaxometry. EUROPEAN NEUROLOGY. 2010;63(3):136-43.
597.	Burgetova A, Seidl Z, Krasensky J, Horakova D, Vaneckova M. Multiple sclerosis and the accumulation of iron in the basal ganglia: quantitative assessment of brain iron using MRI T2 relaxometry. European neurology. 2010;63(3):136-43.
598.	Burgetova A, Seidl Z, Vaneckova M, Krasensky J, Horakova D. Magnetic Resonance Relaxometry in Multiple Sclerosis-T2 Relaxation Time Measurement in Central Gray Matter. CESKA A SLOVENSKA NEUROLOGIE A NEUROCHIRURGIE. 2010;73(1):26-31.
599.	Burgetová A, Seidl Z, Vaněčková M, Krásenský J, Horáková D. Magnetic resonance relaxometry in multiple sclerosis - T2 relaxation time measurement in central gray matter. Ceska a Slovenska Neurologie a Neurochirurgie. 2010.
600.	Burgetova A, Seidl Z, Vaneckova M, Krasensky J, Horakova D. Magnetic Resonance Relaxometry in Multiple Sclerosis-T2 Relaxation Time Measurement in Central Gray Matter. CESKA A SLOVENSKA NEUROLOGIE A NEUROCHIRURGIE. 2010;73(1):26-31.
601.	Burgos RA, Alarcon P, Quiroga J, Manosalva C, Hancke J. Andrographolide, an Anti-Inflammatory Multitarget Drug: All Roads Lead to Cellular Metabolism. MOLECULES. 2021;26(1).
602.	Burisch J, Jess T, Egeberg A. Incidence of Immune-Mediated Inflammatory Diseases Among Patients With Inflammatory Bowel Diseases in Denmark. Clin Gastroenterol Hepatol. 2019;17(13):2704-12.e3.
603.	Burster T, Beck A, Tolosa E, Schnorrer P, Weissert R, Reich M, et al. Differential processing of autoantigens in lysosomes from human monocyte-derived and peripheral blood dendritic cells. JOURNAL OF IMMUNOLOGY. 2005;175(9):5940-9.
604.	Burster T, Beck A, Tolosa E, Schnorrer P, Weissert R, Reich M, et al. Differential processing of autoantigens in lysosomes from human monocyte-derived and peripheral blood dendritic cells. J Immunol. 2005;175(9):5940-9.
605.	Burster T, Beck A, Tolosa E, Schnorrer P, Weissert R, Reich M, et al. Differential processing of autoantigens in lysosomes from human monocyte-derived and peripheral blood dendritic cells. JOURNAL OF IMMUNOLOGY. 2005;175(9):5940-9.
606.	Buscarinu MC, Romano S, Mechelli R, Pizzolato Umeton R, Ferraldeschi M, Fornasiero A, et al. Intestinal Permeability in Relapsing-Remitting Multiple Sclerosis. Neurotherapeutics. 2018;15(1):68-74.
607.	Busch AK, Fringer A. Psychosocial Impact of Multiple Sclerosis on Couples: Relationship Between Anxiety, Depression, and Stress Communication of Both Partners. J Prim Care Community Health. 2022;13:21501319221119142.
608.	Bustamante-Barrientos FA, Luque-Campos N, Araya MJ, Lara-Barba E, de Solminihac J, Pradenas C, et al. Mitochondrial dysfunction in neurodegenerative disorders: Potential therapeutic application of mitochondrial transfer to central nervous system-residing cells. J Transl Med. 2023;21(1):613.
609.	Butler EJ. Chronic neurological disease as a possible form of lead poisoning. J Neurol Neurosurg Psychiatry. 1952;15(2):119-28.
610.	Butler R, Bradford D, Rodgers KE. Analysis of shared underlying mechanism in neurodegenerative disease. Front Aging Neurosci. 2022;14:1006089.
611.	Butovsky O, Landa G, Kunis G, Ziv Y, Avidan H, Greenberg N, et al. Induction and blockage of oligodendrogenesis by differently activated microglia in an animal model of multiple sclerosis. J Clin Invest. 2006;116(4):905-15.
612.	Cabaraux P, Agrawal SK, Cai H, Calabro RS, Casali C, Damm L, et al. Consensus Paper: Ataxic Gait. Cerebellum. 2023;22(3):394-430.
613.	Cacciaguerra L, Sechi E, Rocca MA, Filippi M, Pittock SJ, Flanagan EP. Neuroimaging features in inflammatory myelopathies: A review. Front Neurol. 2022;13:993645.
614.	Cadegiani FA. Remission of Severe Myasthenia Gravis After Massive-Dose Vitamin D Treatment. Am J Case Rep. 2016;17:51-4.
615.	Cajamarca-Baron J, Guavita-Navarro D, Buitrago-Bohorquez J, Gallego-Cardona L, Navas A, Cubides H, et al. [SARS-CoV-2 (COVID-19) in Patients with some Degree of Immunosuppression]. Reumatol Clin. 2021;17(7):408-19.
616.	Cajamarca-Baron J, Guavita-Navarro D, Buitrago-Bohorquez J, Gallego-Cardona L, Navas A, Cubides H, et al. SARS-CoV-2 (COVID-19) in patients with some degree of immunosuppression. Reumatol Clin (Engl Ed). 2021;17(7):408-19.
617.	Calabrese V, Lodi R, Tonon C, D'Agata V, Sapienza M, Scapagnini G, et al. Oxidative stress, mitochondrial dysfunction and cellular stress response in Friedreich's ataxia. J Neurol Sci. 2005;233(1-2):145-62.
618.	Calabrese V, Lodi R, Tonon C, D'Agata V, Sapienza M, Scapagnini G, et al. Oxidative stress, mitochondrial dysfunction and cellular stress response in Friedreich's ataxia. JOURNAL OF THE NEUROLOGICAL SCIENCES. 2005;233(1-2):145-62.
619.	Calabrese V, Lodi R, Tonon C, D'Agata V, Sapienza M, Scapagnini G, et al. Oxidative stress, mitochondrial dysfunction and cellular stress response in Friedreich's ataxia. Journal of the Neurological Sciences. 2005.
620.	Calabrese V, Lodi R, Tonon C, D'Agata V, Sapienza M, Scapagnini G, et al. Oxidative stress, mitochondrial dysfunction and cellular stress response in Friedreich's ataxia. JOURNAL OF THE NEUROLOGICAL SCIENCES. 2005;233(1-2):145-62.
621.	Calafiore D, Invernizzi M, Ammendolia A, Marotta N, Fortunato F, Paolucci T, et al. Efficacy of Virtual Reality and Exergaming in Improving Balance in Patients With Multiple Sclerosis: A Systematic Review and Meta-Analysis. Front Neurol. 2021;12:773459.
622.	Cali F, Tse A, Wong J, Lin J, Sadiq S. Intrathecal Injections of Primary Progressive Multiple Sclerosis Derived Antibodies Result in Motor Deficits and CNS Pathology in Mice Suggesting Leading Role of Antibodies in CSF Effects. NEUROLOGY. 2019;92(15).
623.	Çalıkuşu İ, Uzunhisarcıklı E, Fidan U, Çetinkaya MB. Analysing the effect of robotic gait on lower extremity muscles and classification by using deep learning. Comput Methods Biomech Biomed Engin. 2022;25(12):1350-69.
624.	Callander M, Haghighi S, Landtblom AM, Ahlgren CE, Nilsson SI, Rydberg L, et al. Multiple sclerosis immunopathic trait and HLA-DR(2)15 as independent risk factors in multiple sclerosis. Mult Scler. 2007;13(4):441-5.
625.	Callegari I, Oechtering J, Schneider M, Perriot S, Mathias A, Voortman MM, et al. Cell-binding IgM in CSF is distinctive of multiple sclerosis and targets the iron transporter SCARA5. Brain. 2024;147(3):839-48.
626.	Caltabiano R, De Pasquale R, Piombino E, Campo G, Nicoletti F, Cavalli E, et al. Macrophage Migration Inhibitory Factor (MIF) and Its Homologue d-Dopachrome Tautomerase (DDT) Inversely Correlate with Inflammation in Discoid Lupus Erythematosus. Molecules. 2021;26(1).
627.	Calvi A, Haider L, Prados F, Tur C, Chard D, Barkhof F. In vivo imaging of chronic active lesions in multiple sclerosis. MULTIPLE SCLEROSIS JOURNAL.
628.	Calvi A, Haider L, Prados F, Tur C, Chard D, Barkhof F. In vivo imaging of chronic active lesions in multiple sclerosis. Multiple Sclerosis Journal. 2020.
629.	Calvi A, Haider L, Prados F, Tur C, Chard D, Barkhof F. In vivo imaging of chronic active lesions in multiple sclerosis. Mult Scler. 2022;28(5):683-90.
630.	Campbell AM, Herdan G, Tatlow WF, Whittle EG. Lead in relation to disseminated sclerosis. Brain. 1950;73(1):52-71.
631.	Campbell NK, Fitzgerald HK, Dunne A. Regulation of inflammation by the antioxidant haem oxygenase 1. Nat Rev Immunol. 2021;21(7):411-25.
632.	Campos-Escamilla C. The role of transferrins and iron-related proteins in brain iron transport: applications to neurological diseases. In: Donev R, editor. ADVANCES IN PROTEIN CHEMISTRY AND STRUCTURAL BIOLOGY, VOL 123: TRANSPORT PROTEINS. 1232021. p. 133-62.
633.	Campos-Escamilla C. The role of transferrins and iron-related proteins in brain iron transport: applications to neurological diseases. Advances in Protein Chemistry and Structural Biology2021.
634.	Campos-Escamilla C. The role of transferrins and iron-related proteins in brain iron transport: applications to neurological diseases. Adv Protein Chem Struct Biol. 2021;123:133-62.
635.	Cannas D, Loi E, Serra M, Firinu D, Valera P, Zavattari P. Relevance of Essential Trace Elements in Nutrition and Drinking Water for Human Health and Autoimmune Disease Risk. NUTRIENTS. 2020;12(7).
636.	Cannas D, Loi E, Serra M, Firinu D, Valera P, Zavattari P. Relevance of essential trace elements in nutrition and drinking water for human health and autoimmune disease risk. Nutrients. 2020.
637.	Cannas D, Loi E, Serra M, Firinu D, Valera P, Zavattari P. Relevance of Essential Trace Elements in Nutrition and Drinking Water for Human Health and Autoimmune Disease Risk. Nutrients. 2020;12(7).
638.	Cannon JR, Greenamyre JT. The role of environmental exposures in neurodegeneration and neurodegenerative diseases. Toxicological Sciences. 2011.
639.	Cano A, Fonseca E, Ettcheto M, Sánchez-López E, de Rojas I, Alonso-Lana S, et al. Epilepsy in Neurodegenerative Diseases: Related Drugs and Molecular Pathways. Pharmaceuticals (Basel). 2021;14(10).
640.	Cantor F. Central and peripheral fatigue: exemplified by multiple sclerosis and myasthenia gravis. Pm r. 2010;2(5):399-405.
641.	Cao L. [Preliminary studies of T lymphocyte subsets in patients with neurologic diseases]. Zhonghua Shen Jing Jing Shen Ke Za Zhi. 1990;23(3):159-61, 90.
642.	Cao L, Li M, Yao L, Yan P, Wang X, Yang Z, et al. Siponimod for multiple sclerosis. Cochrane Database Syst Rev. 2021;11(11):Cd013647.
643.	Cao RR, He P, Lei SF. Novel microbiota-related gene set enrichment analysis identified osteoporosis associated gut microbiota from autoimmune diseases. J Bone Miner Metab. 2021;39(6):984-96.
644.	Caprariello AV, Adams DJ. The landscape of targets and lead molecules for remyelination. Nat Chem Biol. 2022;18(9):925-33.
645.	Cariccio VL, Sama A, Bramanti P, Mazzon E. Mercury Involvement in Neuronal Damage and in Neurodegenerative Diseases. BIOLOGICAL TRACE ELEMENT RESEARCH. 2019;187(2):341-56.
646.	Cariccio VL, Samà A, Bramanti P, Mazzon E. Mercury Involvement in Neuronal Damage and in Neurodegenerative Diseases. Biological Trace Element Research. 2019.
647.	Cariccio VL, Samà A, Bramanti P, Mazzon E. Mercury Involvement in Neuronal Damage and in Neurodegenerative Diseases. Biol Trace Elem Res. 2019;187(2):341-56.
648.	Carl J, Hartung V, Tallner A, Pfeifer K. The Relevance of Competences for a Healthy, Physically Active Lifestyle in Persons with Multiple Sclerosis: a Path Analytical Approach. Behav Med. 2022;48(4):331-41.
649.	Carlens C, Hergens MP, Grunewald J, Ekbom A, Eklund A, Höglund CO, et al. Smoking, use of moist snuff, and risk of chronic inflammatory diseases. Am J Respir Crit Care Med. 2010;181(11):1217-22.
650.	Carlson AK, Amin M, Cohen JA. Drugs Targeting CD20 in Multiple Sclerosis: Pharmacology, Efficacy, Safety, and Tolerability. Drugs. 2024;84(3):285-304.
651.	Carmona A, Carboni E, Gomes LC, Roudeau S, Maass F, Lenz C, et al. Metal dyshomeostasis in the substantia nigra of patients with Parkinson's disease or multiple sclerosis. J Neurochem. 2024;168(2):128-41.
652.	Carol Stein E, Schiffer RB, Jackson Hall W, Young N. Multiple sclerosis and the workplace: Report of an industry-based cluster. Neurology. 1987.
653.	Carta MG, Conti A, Lecca F, Sancassiani F, Cossu G, Carruxi R, et al. The Burden of Depressive and Bipolar Disorders in Celiac Disease. Clin Pract Epidemiol Ment Health. 2015;11:180-5.
654.	Carta S, Ferraro D, Ferrari S, Briani C, Mariotto S. Oligoclonal bands: clinical utility and interpretation cues. Crit Rev Clin Lab Sci. 2022;59(6):391-404.
655.	Casella G, Bordo BM, Schalling R, Villanacci V, Salemme M, Di Bella C, et al. Neurological disorders and celiac disease. Minerva Gastroenterol Dietol. 2016;62(2):197-206.
656.	Caslin B, Karmakar A, Maguire C, Helmsdoerfer K, Mohler K, Kirwin S, et al. Alcohol consumption leads to sex-specific amelioration of disability in a mouse model of multiple sclerosis. JOURNAL OF IMMUNOLOGY. 2019;202(1).
657.	Caslin B, Maguire C, Karmakar A, Helmsdoerfer K, Mohler K, Ward J, et al. Moderate alcohol diet leads to sex-specific disease amelioration in a mouse model of multiple sclerosis via effects on the Gut-CNS Axis. MULTIPLE SCLEROSIS JOURNAL. 2019;25:669-.
658.	Castaigne P, Lhermitte F, Schuller E, Rouques C. [Cerebrospinal fluid proteins in the course of amyotrophic lateral sclerosis]. Rev Neurol (Paris). 1971;125(5):393-400.
659.	Castellaro M, Magliozzi R, Palombit A, Pitteri M, Silvestri E, Camera V, et al. Heterogeneity of Cortical Lesion Susceptibility Mapping in Multiple Sclerosis. AMERICAN JOURNAL OF NEURORADIOLOGY. 2017;38(6):1087-95.
660.	Castellaro M, Magliozzi R, Palombit A, Pitteri M, Silvestri E, Camera V, et al. Heterogeneity of cortical lesion susceptibility mapping in multiple sclerosis. American Journal of Neuroradiology. 2017.
661.	Castellaro M, Magliozzi R, Palombit A, Pitteri M, Silvestri E, Camera V, et al. Heterogeneity of Cortical Lesion Susceptibility Mapping in Multiple Sclerosis. AJNR Am J Neuroradiol. 2017;38(6):1087-95.
662.	Castellaro M, Magliozzi R, Palombit A, Pitteri M, Silvestri E, Camera V, et al. Heterogeneity of Cortical Lesion Susceptibility Mapping in Multiple Sclerosis. AMERICAN JOURNAL OF NEURORADIOLOGY. 2017;38(6):1087-95.
663.	Castellaro M, Magliozzi R, Rossi S, Pitteri M, Montemezzi S, Pizzini FB, et al., editors. Iron loss of thalamic nuclei evaluated with quantitative susceptibility mapping is related to intrathecal macrophages activity and cortical pathology of multiple sclerosis2017: SAGE PUBLICATIONS LTD 1 OLIVERS YARD, 55 CITY ROAD, LONDON EC1Y 1SP, ENGLAND.
664.	Castellaro M, Magliozzi R, Rossi S, Pitteri M, Montemezzi S, Pizzini FB, et al. Iron loss of thalamic nuclei evaluated with quantitative susceptibility mapping is related to intrathecal macrophages activity and cortical pathology of multiple sclerosis. MULTIPLE SCLEROSIS JOURNAL. 2017;23:534-.
665.	Castro Á DS, Albuquerque LDS, Melo MLP, D'Almeida JAC, Braga RAM, Assis RC, et al. Relationship between zinc-related nutritional status and the progression of multiple sclerosis. Mult Scler Relat Disord. 2022;66:104063.
666.	Catalanotto F, Doreduffy P, Donaldson J, Ostrom KM. TASTE SENSITIVITY AND ZINC-METABOLISM IN MULTIPLE-SCLEROSIS. JOURNAL OF DENTAL RESEARCH. 1981;60:559-.
667.	Cavalli E, Mazzon E, Basile MS, Mammana S, Pennisi M, Fagone P, et al. In Silico and In Vivo Analysis of IL37 in Multiple Sclerosis Reveals Its Probable Homeostatic Role on the Clinical Activity, Disability, and Treatment with Fingolimod. Molecules. 2019;25(1).
668.	Cebrián-Pérez EM, Prieto JM, Lema M, Amigo-Jorrín MC, Iglesias-Gómez S, Noya-García M. [Time evolution of TNF-alpha, VCAM-1, IL-4, IL-10, neopterin and CD-30 in patients treated with interferon]. Rev Neurol. 2004;39(3):213-7.
669.	Ceccarelli A, Filippi M, Neema M, Arora A, Valsasina P, Rocca MA, et al. T2 hypointensity in the deep gray matter of patients with benign multiple sclerosis. MULTIPLE SCLEROSIS JOURNAL. 2009;15(6):678-86.
670.	Ceccarelli A, Filippi M, Neema M, Arora A, Valsasina P, Rocca MA, et al. T2 hypointensity in the deep gray matter of patients with benign multiple sclerosis. Multiple Sclerosis. 2009.
671.	Ceccarelli A, Filippi M, Neema M, Arora A, Valsasina P, Rocca MA, et al. T2 hypointensity in the deep gray matter of patients with benign multiple sclerosis. Mult Scler. 2009;15(6):678-86.
672.	Ceccarelli A, Filippi M, Neema M, Arora A, Valsasina P, Rocca MA, et al. T2 hypointensity in the deep gray matter of patients with benign multiple sclerosis. MULTIPLE SCLEROSIS JOURNAL. 2009;15(6):678-86.
673.	Ceccarelli A, Rocca MA, Neema M, Martinelli V, Arora A, Tauhid S, et al. Deep gray matter T2 hypointensity is present in patients with clinically isolated syndromes suggestive of multiple sclerosis. MULTIPLE SCLEROSIS JOURNAL. 2010;16(1):39-44.
674.	Ceccarelli A, Rocca MA, Neema M, Martinelli V, Arora A, Tauhid S, et al. Deep gray matter T2 hypointensity is present in patients with clinically isolated syndromes suggestive of multiple sclerosis. Multiple Sclerosis. 2010.
675.	Ceccarelli A, Rocca MA, Neema M, Martinelli V, Arora A, Tauhid S, et al. Deep gray matter T2 hypointensity is present in patients with clinically isolated syndromes suggestive of multiple sclerosis. Mult Scler. 2010;16(1):39-44.
676.	Ceccarelli A, Rocca MA, Neema M, Martinelli V, Arora A, Tauhid S, et al. Deep gray matter T2 hypointensity is present in patients with clinically isolated syndromes suggestive of multiple sclerosis. MULTIPLE SCLEROSIS JOURNAL. 2010;16(1):39-44.
677.	Ceccarelli A, Rocca MA, Perego E, Moiola L, Ghezzi A, Martinelli V, et al. Deep grey matter T2 hypo-intensity in patients with paediatric multiple sclerosis. MULTIPLE SCLEROSIS JOURNAL. 2011;17(6):702-7.
678.	Ceccarelli A, Rocca MA, Perego E, Moiola L, Ghezzi A, Martinelli V, et al. Deep grey matter T2 hypo-intensity in patients with paediatric multiple sclerosis. Multiple Sclerosis Journal. 2011.
679.	Ceccarelli A, Rocca MA, Perego E, Moiola L, Ghezzi A, Martinelli V, et al. Deep grey matter T2 hypo-intensity in patients with paediatric multiple sclerosis. Mult Scler. 2011;17(6):702-7.
680.	Ceccarelli A, Rocca MA, Perego E, Moiola L, Ghezzi A, Martinelli V, et al. Deep grey matter T2 hypo-intensity in patients with paediatric multiple sclerosis. MULTIPLE SCLEROSIS JOURNAL. 2011;17(6):702-7.
681.	Cendrowski W, Szajbel W. The oxidase activity of serum ceruloplasmin in multiple sclerosis. Acta Neurol Scand. 1966;42(3):379-84.
682.	Cénit MC, Alcina A, Márquez A, Mendoza JL, Díaz-Rubio M, de las Heras V, et al. STAT3 locus in inflammatory bowel disease and multiple sclerosis susceptibility. Genes Immun. 2010;11(3):264-8.
683.	Cepok S, Rosche B, Grummel V, Vogel F, Zhou D, Sayn J, et al. Short-lived plasma blasts are the main B cell effector subset during the course of multiple sclerosis. BRAIN. 2005;128:1667-76.
684.	Cepok S, Rosche B, Grummel V, Vogel F, Zhou D, Sayn J, et al. Short-lived plasma blasts are the main B cell effector subset during the course of multiple sclerosis. Brain. 2005;128(Pt 7):1667-76.
685.	Cepok S, Rosche B, Grummel V, Vogel F, Zhou D, Sayn J, et al. Short-lived plasma blasts are the main B cell effector subset during the course of multiple sclerosis. BRAIN. 2005;128:1667-76.
686.	Cerasuolo M, Di Meo I, Auriemma MC, Trojsi F, Maiorino MI, Cirillo M, et al. Iron and Ferroptosis More than a Suspect: Beyond the Most Common Mechanisms of Neurodegeneration for New Therapeutic Approaches to Cognitive Decline and Dementia. Int J Mol Sci. 2023;24(11).
687.	Cervellati C, Romani A, Fainardi E, Trentini A, Squerzanti M, Baldi E, et al. Serum ferroxidase activity in patients with multiple sclerosis: a pilot study. In Vivo. 2014;28(6):1197-200.
688.	Cesbron E, Monfort JB, Giannesini C, Duriez P, Moguelet P, Senet P, et al. [Primary cutaneous CD30+ T-cell lymphoproliferation during treatment with fingolimod: Case report and literature review]. Ann Dermatol Venereol. 2018;145(6-7):433-8.
689.	Ceylan U, Haupeltshofer S, Kamper L, Dann J, Ambrosius B, Gold R, et al. Clozapine Regulates Microglia and Is Effective in Chronic Experimental Autoimmune Encephalomyelitis. FRONTIERS IN IMMUNOLOGY. 2021;12.
690.	Ceylan U, Haupeltshofer S, Kämper L, Dann J, Ambrosius B, Gold R, et al. Clozapine Regulates Microglia and Is Effective in Chronic Experimental Autoimmune Encephalomyelitis. Frontiers in Immunology. 2021.
691.	Ceylan U, Haupeltshofer S, Kämper L, Dann J, Ambrosius B, Gold R, et al. Clozapine Regulates Microglia and Is Effective in Chronic Experimental Autoimmune Encephalomyelitis. Front Immunol. 2021;12:656941.
692.	Ceylan U, Haupeltshofer S, Kamper L, Dann J, Ambrosius B, Gold R, et al. Clozapine Regulates Microglia and Is Effective in Chronic Experimental Autoimmune Encephalomyelitis. FRONTIERS IN IMMUNOLOGY. 2021;12.
693.	Chakrabarti B, Bairagya HR, Mishra DK, Chatterjee PK, Mukhopadhyay BP. Insight towards the conserved water mediated recognition of catalytic and structural Zn+2 ions in human Matrix Metalloproteinase-8 enzyme: A study by MD-simulation methods. BIOINFORMATION. 2013;9(3):126-33.
694.	Chakrabarti B, Bairagya HR, Mishra DK, Chatterjee PK, Mukhopadhyay BP. Insight towards the conserved water mediated recognition of catalytic and structural Zn(+2) ions in human Matrix Metalloproteinase-8 enzyme: A study by MD-simulation methods. Bioinformation. 2013;9(3):126-33.
695.	Chakrabarti B, Bairagya HR, Mishra DK, Chatterjee PK, Mukhopadhyay BP. Insight towards the conserved water mediated recognition of catalytic and structural Zn+2 ions in human Matrix Metalloproteinase-8 enzyme: A study by MD-simulation methods. BIOINFORMATION. 2013;9(3):126-33.
696.	Chakrabarty A, Emerson MR, LeVine SM. Heme oxygenase-1 in SJL mice with experimental allergic encephalomyelitis. MULTIPLE SCLEROSIS. 2003;9(4):372-81.
697.	Chakrabarty A, Emerson MR, LeVine SM. Heme oxygenase-I in SJL mice with experimental allergic encephalomyelitis. Multiple Sclerosis. 2003.
698.	Chakrabarty A, Emerson MR, LeVine SM. Heme oxygenase-1 in SJL mice with experimental allergic encephalomyelitis. Mult Scler. 2003;9(4):372-81.
699.	Chakrabarty A, Emerson MR, LeVine SM. Heme oxygenase-1 in SJL mice with experimental allergic encephalomyelitis. MULTIPLE SCLEROSIS. 2003;9(4):372-81.
700.	Chakraborty S, Dutta S, Saha R, Moi SC, Sukul D, Panja SS. Efficacy of a photo-catalyst towards the degradation of a pharmaceutical compound, 4-aminopyridine by application of response surface methodology. DESALINATION AND WATER TREATMENT. 2017;76:389-97.
701.	Chakraborty S, Dutta S, Saha R, Moi SC, Sukul D, Panja SS. Efficacy of a photo-catalyst towards the degradation of a pharmaceutical compound, 4-aminopyridine by application of response surface methodology. Desalination and Water Treatment. 2017.
702.	Chakraborty S, Dutta S, Saha R, Moi SC, Sukul D, Panja SS. Efficacy of a photo-catalyst towards the degradation of a pharmaceutical compound, 4-aminopyridine by application of response surface methodology. DESALINATION AND WATER TREATMENT. 2017;76:389-97.
703.	Chakravarty A. Friedreich's ataxia - yesterday, today and tomorrow. NEUROLOGY INDIA. 2003;51(2):176-82.
704.	Chakravarty A. Friedreich's ataxia - Yesterday, today and tomorrow. Neurology India. 2003.
705.	Chakravarty A. Friedreich's ataxia--yesterday, today and tomorrow. Neurol India. 2003;51(2):176-82.
706.	Chalkou K, Vickers AJ, Pellegrini F, Manca A, Salanti G. Decision Curve Analysis for Personalized Treatment Choice between Multiple Options. Med Decis Making. 2023;43(3):337-49.
707.	Chalmer TA, Kalincik T, Laursen B, Sorensen PS, Magyari M, Sellebjerg F, et al. Treatment escalation leads to fewer relapses compared with switching to another moderately effective therapy. JOURNAL OF NEUROLOGY. 2019;266(2):306-15.
708.	Chalmer TA, Kalincik T, Laursen B, Sorensen PS, Magyari M, Sellebjerg F, et al. Treatment escalation leads to fewer relapses compared with switching to another moderately effective therapy. JOURNAL OF NEUROLOGY. 2019;266(2):306-15.
709.	Chan A, Weilbach FX, Toyka KV, Gold R. Mitoxantrone induces cell death in peripheral blood leucocytes of multiple sclerosis patients. Clin Exp Immunol. 2005;139(1):152-8.
710.	Chandler S, Lury J. Macrophage metalloelastase (MMP-12) degrades matrix and myelin proteins. FASEB Journal. 1996.
711.	Chandler S, Miller KM, Clements JM, Lury J, Corkill D, Anthony DC, et al. Matrix metalloproteinases, tumor necrosis factor and multiple sclerosis: an overview. J Neuroimmunol. 1997;72(2):155-61.
712.	Chandler S, Miller KM, Clements JM, Lury J, Corkill D, Anthony DCC, et al. Matrix metalloproteinases, tumor necrosis factor and multiple sclerosis: An overview. JOURNAL OF NEUROIMMUNOLOGY. 1997;72(2):155-61.
713.	Chandler S, Miller KM, Clements JM, Lury J, Corkill D, Anthony DCC, et al. Matrix metalloproteinases, tumor necrosis factor and multiple sclerosis: An overview. Journal of Neuroimmunology. 1997.
714.	Chandler S, Miller KM, Clements JM, Lury J, Corkill D, Anthony DCC, et al. Matrix metalloproteinases, tumor necrosis factor and multiple sclerosis: An overview. JOURNAL OF NEUROIMMUNOLOGY. 1997;72(2):155-61.
715.	Chang MC, Lee BJ, Yang D, Kim CR, Park D, Kim S. The association between cognition and gait disturbance in central nervous system demyelinating disorder with mild disability. BMC Neurol. 2023;23(1):177.
716.	Chard DT, Dalton CM, Swanton J, Fisniku LK, Miszkiel KA, Thompson AJ, et al. MRI only conversion to multiple sclerosis following a clinically isolated syndrome. J Neurol Neurosurg Psychiatry. 2011;82(2):176-9.
717.	Chataway J. Biotin in progressive multiple sclerosis: A new lead? MULTIPLE SCLEROSIS JOURNAL. 2016;22(13):1640-1.
718.	Chataway J. Biotin in progressive multiple sclerosis: A new lead? : SAGE Publications Sage UK: London, England; 2016. p. 1640-1.
719.	Chatterjee A, Chakravarty A. Neurological Complications Following COVID-19 Vaccination. Curr Neurol Neurosci Rep. 2023;23(1):1-14.
720.	Chatzikonstantinou S, Poulidou V, Arnaoutoglou M, Kazis D, Heliopoulos I, Grigoriadis N, et al. Signaling through the S1P-S1PR Axis in the Gut, the Immune and the Central Nervous System in Multiple Sclerosis: Implication for Pathogenesis and Treatment. Cells. 2021;10(11).
721.	Chawla S, Kister I, Herbert J, Brisset JC, Dusek P, Wuerfel JT, et al., editors. Assessment of iron deposition pattern in multiple sclerosis and neuromyelitis optica lesions with ultra-high field quantitative susceptibility mapping2014: SAGE PUBLICATIONS LTD 1 OLIVERS YARD, 55 CITY ROAD, LONDON EC1Y 1SP, ENGLAND.
722.	Chawla S, Kister I, Herbert J, Brisset JC, Dusek P, Wuerfel JT, et al. Assessment of iron deposition pattern in multiple sclerosis and neuromyelitis optica lesions with ultra-high field quantitative susceptibility mapping. MULTIPLE SCLEROSIS JOURNAL. 2014;20:24-.
723.	Chawla S, Kister I, Sinnecker T, Brisset J, Dusek P, Herbert J, et al. Differentiating Iron and Non-iron Related Pathological Features of Multiple Sclerosis Lesions using Ultra High Field MRI (P6. 119). AAN Enterprises; 2015.
724.	Chawla S, Kister I, Sinnecker T, Wuerfel J, Brisset JC, Paul F, et al. Longitudinal study of multiple sclerosis lesions using ultra-high field (7T) multiparametric MR imaging. PLoS ONE. 2018.
725.	Chawla S, Kister I, Sinnecker T, Wuerfel J, Brisset JC, Paul F, et al. Longitudinal study of multiple sclerosis lesions using ultra-high field (7T) multiparametric MR imaging. PLoS One. 2018;13(9):e0202918.
726.	Chawla S, Kister I, Sinnecker T, Wuerfel J, Brisset JC, Paul F, et al. Longitudinal study of multiple sclerosis lesions using ultra-high field (7T) multiparametric MR imaging. PLOS ONE. 2018;13(9).
727.	Chawla S, Kister I, Sinnecker T, Wuerfel J, Brisset JC, Paul F, et al. Longitudinal study of multiple sclerosis lesions using ultra-high field (7T) multiparametric MR imaging. PLOS ONE. 2018;13(9).
728.	Chawla S, Kister I, Wuerfel J, Brisset JC, Liu S, Sinnecker T, et al. Iron and Non-Iron-Related Characteristics of Multiple Sclerosis and Neuromyelitis Optica Lesions at 7T MRI. AMERICAN JOURNAL OF NEURORADIOLOGY. 2016;37(7):1223-30.
729.	Chawla S, Kister I, Wuerfel J, Brisset JC, Liu S, Sinnecker T, et al. Iron and non-iron-related characteristics of multiple sclerosis and neuromyelitis optica lesions at 7T MRI. American Journal of Neuroradiology. 2016.
730.	Chawla S, Kister I, Wuerfel J, Brisset JC, Liu S, Sinnecker T, et al. Iron and Non-Iron-Related Characteristics of Multiple Sclerosis and Neuromyelitis Optica Lesions at 7T MRI. AMERICAN JOURNAL OF NEURORADIOLOGY. 2016;37(7):1223-30.
731.	Chawla S, Kister I, Wuerfel J, Brisset JC, Liu S, Sinnecker T, et al. Iron and Non-Iron-Related Characteristics of Multiple Sclerosis and Neuromyelitis Optica Lesions at 7T MRI. AJNR Am J Neuroradiol. 2016;37(7):1223-30.
732.	Chawla S, Kister I, Wuerfel J, Brisset JC, Liu S, Sinnecker T, et al. Iron and Non-Iron-Related Characteristics of Multiple Sclerosis and Neuromyelitis Optica Lesions at 7T MRI. AMERICAN JOURNAL OF NEURORADIOLOGY. 2016;37(7):1223-30.
733.	Chawla S, Kister I, Wuerfel J, Brisset JC, Liu S, Sinnecker T, et al. Iron and non-iron-related characteristics of multiple sclerosis and neuromyelitis optica lesions at 7T MRI. American Journal of Neuroradiology. 2016;37(7):1223-30.
734.	Cheli VT, Correale J, Paez PM, Pasquini JM. Iron Metabolism in Oligodendrocytes and Astrocytes, Implications for Myelination and Remyelination. ASN NEURO. 2020;12.
735.	Cheli VT, Correale J, Paez PM, Pasquini JM. Iron Metabolism in Oligodendrocytes and Astrocytes, Implications for Myelination and Remyelination. ASN Neuro. 2020.
736.	Cheli VT, Correale J, Paez PM, Pasquini JM. Iron Metabolism in Oligodendrocytes and Astrocytes, Implications for Myelination and Remyelination. ASN Neuro. 2020;12:1759091420962681.
737.	Chen C, He Y. Causal associations between autoimmune diseases and sarcopenia-related traits: a bi-directional Mendelian randomization study. Front Genet. 2024;15:1325058.
738.	Chen CC, Zechariah A, Hsu YH, Chen HW, Yang LC, Chang C. Neuroaxonal ion dyshomeostasis of the normal-appearing corpus callosum in experimental autoimmune encephalomyelitis. Exp Neurol. 2008;210(2):322-30.
739.	Chen CD, Rudy MA, Zeldich E, Abraham CR. A method to specifically activate the Klotho promoter by using zinc finger proteins constructed from modular building blocks and from naturally engineered Egr1 transcription factor backbone. FASEB JOURNAL. 2020;34(6):7234-46.
740.	Chen CD, Rudy MA, Zeldich E, Abraham CR. A method to specifically activate the Klotho promoter by using zinc finger proteins constructed from modular building blocks and from naturally engineered Egr1 transcription factor backbone. FASEB Journal. 2020.
741.	Chen CD, Rudy MA, Zeldich E, Abraham CR. A method to specifically activate the Klotho promoter by using zinc finger proteins constructed from modular building blocks and from naturally engineered Egr1 transcription factor backbone. Faseb j. 2020;34(6):7234-46.
742.	Chen CD, Rudy MA, Zeldich E, Abraham CR. A method to specifically activate the Klotho promoter by using zinc finger proteins constructed from modular building blocks and from naturally engineered Egr1 transcription factor backbone. FASEB JOURNAL. 2020;34(6):7234-46.
743.	Chen MH, Goverover Y. Self-awareness in multiple sclerosis: Relationships with executive functions and affect. Eur J Neurol. 2021;28(5):1627-35.
744.	Chen P, Miah MR, Aschner M. Metals and Neurodegeneration. F1000Research. 2016.
745.	Chen W, Zhang Y, Mu K, Gauthier SA, Wang Y, Zhu W. Iron is a biomarker for differentiating multiple sclerosis lesions from ischemic demyelinating lesions.
746.	Chen W, Zhang Y, Mu K, Pan C, Gauthier SA, Zhu W, et al. Quantifying the susceptibility variation of normal-appearing white matter in multiple sclerosis by quantitative susceptibility mapping. American Journal of Roentgenology. 2017.
747.	Chen W, Zhang Y, Mu K, Pan C, Gauthier SA, Zhu W, et al. Quantifying the Susceptibility Variation of Normal-Appearing White Matter in Multiple Sclerosis by Quantitative Susceptibility Mapping. AJR Am J Roentgenol. 2017;209(4):889-94.
748.	Chen WW, Zhang Y, Mu KT, Pan C, Gauthier SA, Zhu WZ, et al. Quantifying the Susceptibility Variation of Normal-Appearing White Matter in Multiple Sclerosis by Quantitative Susceptibility Mapping. AMERICAN JOURNAL OF ROENTGENOLOGY. 2017;209(4):889-94.
749.	Chen WW, Zhang Y, Mu KT, Pan C, Gauthier SA, Zhu WZ, et al. Quantifying the Susceptibility Variation of Normal-Appearing White Matter in Multiple Sclerosis by Quantitative Susceptibility Mapping. AMERICAN JOURNAL OF ROENTGENOLOGY. 2017;209(4):889-94.
750.	Chen WY, Qian Y, Chen TY, Gu XP. [Research Progress of CD4(+)T Cells-mediated Regulation of Neuroinflammation Involved in Neurodegenerative Diseases]. Zhongguo Yi Xue Ke Xue Yuan Xue Bao. 2021;43(4):628-33.
751.	Chen X, Li Y-m, Luo T-y, Ouyang Y, Lv F-j, Zen C, et al. Quantitative analysis of iron deposition in the deep nucleus of relapsing-remitting multiple sclerosis and relapsing neuromyelitis optica with ESWAN. Chinese Journal of Medical Imaging Technology. 2012.
752.	Chen X, Li YM, Luo TY, Ouyang Y, Lv FJ, Zeng C, et al. Quantitative analysis of iron deposition in the deep nucleus of relapsing-remitting multiple sclerosis and relapsing neuromyelitis optica with ESWAN. Chinese Journal of Medical Imaging Technology. 2012.
753.	Chen X, Zeng C, Luo T, Ouyang Y, Lv F, Rumzan R, et al. Iron deposition of the deep grey matter in patients with multiple sclerosis and neuromyelitis optica: a control quantitative study by 3D-enhanced susceptibility-weighted angiography (ESWAN). European journal of radiology. 2012;81(4):e633-e9.
754.	Chen X, Zeng C, Luo T, Ouyang Y, Lv F, Rumzan R, et al. Iron deposition of the deep grey matter in patients with multiple sclerosis and neuromyelitis optica: A control quantitative study by 3D-enhanced susceptibility-weighted angiography (ESWAN). European Journal of Radiology. 2012.
755.	Chen X, Zeng C, Luo T, Ouyang Y, Lv F, Rumzan R, et al. Iron deposition of the deep grey matter in patients with multiple sclerosis and neuromyelitis optica: a control quantitative study by 3D-enhanced susceptibility-weighted angiography (ESWAN). Eur J Radiol. 2012;81(4):e633-9.
756.	Chen X, Zeng C, Luo TY, Ouyang Y, Lv FJ, Rumzan R, et al. Iron deposition of the deep grey matter in patients with multiple sclerosis and neuromyelitis optica: A control quantitative study by 3D-enhanced susceptibility-weighted angiography (ESWAN). EUROPEAN JOURNAL OF RADIOLOGY. 2012;81(4):E633-E9.
757.	Chen X, Zeng C, Luo TY, Ouyang Y, Lv FJ, Rumzan R, et al. Iron deposition of the deep grey matter in patients with multiple sclerosis and neuromyelitis optica: A control quantitative study by 3D-enhanced susceptibility-weighted angiography (ESWAN). EUROPEAN JOURNAL OF RADIOLOGY. 2012;81(4):E633-E9.
758.	Chen X, Zeng C, Luo TY, Ouyang Y, Lv FJ, Rumzan R, et al. Iron deposition of the deep grey matter in patients with multiple sclerosis and neuromyelitis optica: A control quantitative study by 3D-enhanced susceptibility-weighted angiography (ESWAN). EUROPEAN JOURNAL OF RADIOLOGY. 2012;81(4):E633-E9.
759.	Chen X, Zhang Y, Wang H, Liu L, Li W, Xie P. The regulatory effects of lactic acid on neuropsychiatric disorders. Discov Ment Health. 2022;2(1):8.
760.	Chen Y, Wang H, Wang WZ, Wang D, Skaggs K, Zhang HT. Phosphodiesterase 7(PDE7): A unique drug target for central nervous system diseases. Neuropharmacology. 2021;196:108694.
761.	Chenard CA, Rubenstein LM, Snetselaar LG, Wahls TL. Nutrient Composition Comparison between a Modified Paleolithic Diet for Multiple Sclerosis and the Recommended Healthy US-Style Eating Pattern. NUTRIENTS. 2019;11(3).
762.	Chenard CA, Rubenstein LM, Snetselaar LG, Wahls TL. Nutrient Composition Comparison between the Low Saturated Fat Swank Diet for Multiple Sclerosis and Healthy US-Style Eating Pattern. NUTRIENTS. 2019;11(3).
763.	Chenard CA, Rubenstein LM, Snetselaar LG, Wahls TL. Nutrient composition comparison between the low saturated fat swank diet for multiple sclerosis and healthy U.S.-style eating pattern. Nutrients. 2019.
764.	Chenard CA, Rubenstein LM, Snetselaar LG, Wahls TL. Nutrient composition comparison between a modified paleolithic diet for multiple sclerosis and the recommended healthy U.S.-style eating pattern. Nutrients. 2019.
765.	Chenard CA, Rubenstein LM, Snetselaar LG, Wahls TL. Nutrient Composition Comparison between the Low Saturated Fat Swank Diet for Multiple Sclerosis and Healthy U.S.-Style Eating Pattern. Nutrients. 2019;11(3).
766.	Chenard CA, Rubenstein LM, Snetselaar LG, Wahls TL. Nutrient Composition Comparison between a Modified Paleolithic Diet for Multiple Sclerosis and the Recommended Healthy U.S.-Style Eating Pattern. Nutrients. 2019;11(3).
767.	Chenard CA, Rubenstein LM, Snetselaar LG, Wahls TL. Nutrient Composition Comparison between a Modified Paleolithic Diet for Multiple Sclerosis and the Recommended Healthy US-Style Eating Pattern. NUTRIENTS. 2019;11(3).
768.	Chenard CA, Rubenstein LM, Snetselaar LG, Wahls TL. Nutrient Composition Comparison between the Low Saturated Fat Swank Diet for Multiple Sclerosis and Healthy US-Style Eating Pattern. NUTRIENTS. 2019;11(3).
769.	Chêne C, Rongvaux-Gaïda D, Thomas M, Rieger F, Nicco C, Batteux F. Optimal combination of arsenic trioxide and copper ions to prevent autoimmunity in a murine HOCl-induced model of systemic sclerosis. Front Immunol. 2023;14:1149869.
770.	Cheng F, Liu H, Luo X, Jiang H, Shen J, Chen K, et al. Progress in the Design of Matrix Metalloproteinase Inhibitors. Progress in Chemistry. 2001.
771.	Cheng F, Liu H, Luo XM, Jiang HL, Shen JK, Chen KX, et al. Progress in the design of matrix metalloproteinase inhibitors. PROGRESS IN CHEMISTRY. 2001;13(4):283-93.
772.	Cheng F, Liu H, Luo XM, Jiang HL, Shen JK, Chen KX, et al. Progress in the design of matrix metalloproteinase inhibitors. PROGRESS IN CHEMISTRY. 2001;13(4):283-93.
773.	Cheng HL, Stikov N, Ghugre NR, Wright GA. Practical medical applications of quantitative MR relaxometry. J Magn Reson Imaging. 2012;36(4):805-24.
774.	Cheng HLM, Stikov N, Ghugre NR, Wright GA. Practical medical applications of quantitative MR relaxometry. JOURNAL OF MAGNETIC RESONANCE IMAGING. 2012;36(4):805-24.
775.	Chernigovskaia NV. [The clinical characteristics and differential diagnosis of hyperkinetic forms of multiple sclerosis]. Zh Nevropatol Psikhiatr Im S S Korsakova. 1970;70(1):23-7.
776.	Chhablani PP, Ambiya V, Nair AG, Bondalapati S, Chhablani J. Retinal Findings on OCT in Systemic Conditions. Seminars in Ophthalmology. 2018.
777.	Chiang GC, Hu J, Morris E, Wang Y, Gauthier SA. Quantitative Susceptibility Mapping of the Thalamus: Relationships with Thalamic Volume, Total Gray Matter Volume, and T2 Lesion Burden. AMERICAN JOURNAL OF NEURORADIOLOGY. 2018;39(3):467-72.
778.	Chiang GC, Hu J, Morris E, Wang Y, Gauthier SA. Quantitative susceptibility mapping of the thalamus: Relationships with thalamic volume, total gray matter volume, and t2 lesion burden. American Journal of Neuroradiology. 2018.
779.	Chiang GC, Hu J, Morris E, Wang Y, Gauthier SA. Quantitative Susceptibility Mapping of the Thalamus: Relationships with Thalamic Volume, Total Gray Matter Volume, and T2 Lesion Burden. AJNR Am J Neuroradiol. 2018;39(3):467-72.
780.	Chiang GC, Hu J, Morris E, Wang Y, Gauthier SA. Quantitative Susceptibility Mapping of the Thalamus: Relationships with Thalamic Volume, Total Gray Matter Volume, and T2 Lesion Burden. AMERICAN JOURNAL OF NEURORADIOLOGY. 2018;39(3):467-72.
781.	Chiang GC, Pinto S, Comunale JP, Gauthier SA. Gadolinium-Enhancing Lesions Lead to Decreases in White Matter Tract Fractional Anisotropy in Multiple Sclerosis. JOURNAL OF NEUROIMAGING. 2016;26(3):289-95.
782.	Chiang GC, Pinto S, Comunale JP, Gauthier SA. Gadolinium-Enhancing Lesions Lead to Decreases in White Matter Tract Fractional Anisotropy in Multiple Sclerosis. JOURNAL OF NEUROIMAGING. 2016;26(3):289-95.
783.	Chiang GC, Pinto S, Comunale JP, Gauthier SA. Gadolinium-Enhancing Lesions Lead to Decreases in White Matter Tract Fractional Anisotropy in Multiple Sclerosis. JOURNAL OF NEUROIMAGING. 2016;26(3):289-95.
784.	Chiang GC, Pinto S, Comunale JP, Gauthier SA. Gadolinium‐Enhancing Lesions Lead to Decreases in White Matter Tract Fractional Anisotropy in Multiple Sclerosis. Journal of Neuroimaging. 2016;26(3):289-95.
785.	Chiba S, Matsumoto H, Motoi Y, Miyano N, Kashiwagi M. High serum adenosine deaminase activity and its correlation with lymphocyte subsets in myasthenia gravis. J Neurol Sci. 1990;100(1-2):174-7.
786.	Chin P, von Rosenstiel P, Haering D, Francis G, Kappos L. Fingolimod leads to early clinical and MRI benefits in relapsing-remitting multiple sclerosis. JOURNAL OF NEUROLOGY. 2012;259:S70-S1.
787.	Chiou B, Lucassen E, Sather M, Kallianpur A, Connor J. Semaphorin4A and H-ferritin utilize Tim-1 on human oligodendrocytes: A novel neuro-immune axis. GLIA. 2018;66(7):1317-30.
788.	Chiou B, Lucassen E, Sather M, Kallianpur A, Connor J. Semaphorin4A and H-ferritin utilize Tim-1 on human oligodendrocytes: A novel neuro-immune axis. GLIA. 2018.
789.	Chiou B, Lucassen E, Sather M, Kallianpur A, Connor J. Semaphorin4A and H-ferritin utilize Tim-1 on human oligodendrocytes: A novel neuro-immune axis. Glia. 2018;66(7):1317-30.
790.	Chiou B, Lucassen E, Sather M, Kallianpur A, Connor J. Semaphorin4A and H-ferritin utilize Tim-1 on human oligodendrocytes: A novel neuro-immune axis. GLIA. 2018;66(7):1317-30.
791.	Chitrala KN, Guan HB, Singh NP, Busbee B, Gandy A, Mehrpouya-Bahrami P, et al. CD44 deletion leading to attenuation of experimental autoimmune encephalomyelitis results from alterations in gut microbiome in mice. EUROPEAN JOURNAL OF IMMUNOLOGY. 2017;47(7):1188-99.
792.	Chitrala KN, Guan HB, Singh NP, Busbee B, Gandy A, Mehrpouya-Bahrami P, et al. CD44 deletion leading to attenuation of experimental autoimmune encephalomyelitis results from alterations in gut microbiome in mice. EUROPEAN JOURNAL OF IMMUNOLOGY. 2017;47(7):1188-99.
793.	Choi BY, Jang BG, Kim JH, Seo J-N, Wu G, Sohn M, et al. Copper/zinc chelation by clioquinol reduces spinal cord white matter damage and behavioral deficits in a murine MOG-induced multiple sclerosis model. Neurobiology of Disease. 2013;54:382-91.
794.	Choi BY, Jang BG, Kim JH, Seo JN, Wu G, Sohn M, et al. Copper/zinc chelation by clioquinol reduces spinal cord white matter damage and behavioral deficits in a murine MOG-induced multiple sclerosis model. Neurobiology of Disease. 2013.
795.	Choi BY, Jang BG, Kim JH, Seo JN, Wu G, Sohn M, et al. Copper/zinc chelation by clioquinol reduces spinal cord white matter damage and behavioral deficits in a murine MOG-induced multiple sclerosis model. NEUROBIOLOGY OF DISEASE. 2013;54:382-91.
796.	Choi BY, Jang BG, Kim JH, Seo JN, Wu G, Sohn M, et al. Copper/zinc chelation by clioquinol reduces spinal cord white matter damage and behavioral deficits in a murine MOG-induced multiple sclerosis model. Neurobiol Dis. 2013;54:382-91.
797.	Choi BY, Jeong JH, Eom JW, Koh JY, Kim YH, Suh SW. A Novel Zinc Chelator, 1H10, Ameliorates Experimental Autoimmune Encephalomyelitis by Modulating Zinc Toxicity and AMPK Activation. INTERNATIONAL JOURNAL OF MOLECULAR SCIENCES. 2020;21(9).
798.	Choi BY, Jeong JH, Eom JW, Koh JY, Kim YH, Suh SW. A novel zinc chelator, 1H10, ameliorates experimental autoimmune encephalomyelitis by modulating zinc toxicity and AMPK activation. International Journal of Molecular Sciences. 2020.
799.	Choi BY, Jeong JH, Eom JW, Koh JY, Kim YH, Suh SW. A Novel Zinc Chelator, 1H10, Ameliorates Experimental Autoimmune Encephalomyelitis by Modulating Zinc Toxicity and AMPK Activation. Int J Mol Sci. 2020;21(9).
800.	Choi BY, Jeong JH, Eom JW, Koh JY, Kim YH, Suh SW. A Novel Zinc Chelator, 1H10, Ameliorates Experimental Autoimmune Encephalomyelitis by Modulating Zinc Toxicity and AMPK Activation. INTERNATIONAL JOURNAL OF MOLECULAR SCIENCES. 2020;21(9).
801.	Choi BY, Jung JW, Suh SW. The Emerging Role of Zinc in the Pathogenesis of Multiple Sclerosis. INTERNATIONAL JOURNAL OF MOLECULAR SCIENCES. 2017;18(10).
802.	Choi BY, Jung JW, Suh SW. The emerging role of zinc in the pathogenesis of multiple sclerosis. International Journal of Molecular Sciences. 2017.
803.	Choi BY, Jung JW, Suh SW. The Emerging Role of Zinc in the Pathogenesis of Multiple Sclerosis. INTERNATIONAL JOURNAL OF MOLECULAR SCIENCES. 2017;18(10).
804.	Choi BY, Jung JW, Suh SW. The Emerging Role of Zinc in the Pathogenesis of Multiple Sclerosis. Int J Mol Sci. 2017;18(10).
805.	Choi BY, Jung JW, Suh SW. The emerging role of zinc in the pathogenesis of multiple sclerosis. International journal of molecular sciences. 2017;18(10):2070.
806.	Choi BY, Kim IY, Kim JH, Kho AR, Lee SH, Lee BE, et al. Zinc transporter 3 (ZnT3) gene deletion reduces spinal cord white matter damage and motor deficits in a murine MOG-induced multiple sclerosis model. Neurobiology of disease. 2016;94:205-12.
807.	Choi BY, Kim IY, Kim JH, Kho AR, Lee SH, Lee BE, et al. Zinc transporter 3 (ZnT3) gene deletion reduces spinal cord white matter damage and motor deficits in a murine MOG-induced multiple sclerosis model. NEUROBIOLOGY OF DISEASE. 2016;94:205-12.
808.	Choi BY, Kim IY, Kim JH, Kho AR, Lee SH, Lee BE, et al. Zinc transporter 3 (ZnT3) gene deletion reduces spinal cord white matter damage and motor deficits in a murine MOG-induced multiple sclerosis model. Neurobiology of Disease. 2016.
809.	Choi BY, Kim IY, Kim JH, Kho AR, Lee SH, Lee BE, et al. Zinc transporter 3 (ZnT3) gene deletion reduces spinal cord white matter damage and motor deficits in a murine MOG-induced multiple sclerosis model. NEUROBIOLOGY OF DISEASE. 2016;94:205-12.
810.	Choi BY, Kim IY, Kim JH, Kho AR, Lee SH, Lee BE, et al. Zinc transporter 3 (ZnT3) gene deletion reduces spinal cord white matter damage and motor deficits in a murine MOG-induced multiple sclerosis model. Neurobiol Dis. 2016;94:205-12.
811.	Choi BY, Kim IY, Kim JH, Kho AR, Lee SH, Lee BE, et al. Zinc transporter 3 (ZnT3) gene deletion reduces spinal cord white matter damage and motor deficits in a murine MOG-induced multiple sclerosis model. NEUROBIOLOGY OF DISEASE. 2016;94:205-12.
812.	Chomyk A, Kucinski R, Kim J, Christie E, Cyncynatus K, Gossman Z, et al. Transcript Profiles of Microglia/Macrophage Cells at the Borders of Chronic Active and Subpial Gray Matter Lesions in Multiple Sclerosis. Ann Neurol. 2024;95(5):907-16.
813.	Chong L, Tian R, Shi R, Ouyang Z, Xia Y. Coupling the Paternò-Büchi (PB) Reaction With Mass Spectrometry to Study Unsaturated Fatty Acids in Mouse Model of Multiple Sclerosis. Front Chem. 2019;7:807.
814.	Chonglo L, Tian R, Shi RY, Ouyang Z, Xia Y. Coupling the Paterno-Buchi (PB) Reaction With Mass Spectrometry to Study Unsaturated Fatty Acids in Mouse Model of Multiple Sclerosis. FRONTIERS IN CHEMISTRY. 2019;7.
815.	Chonglo L, Tian R, Shi RY, Ouyang Z, Xia Y. Coupling the Paterno-Buchi (PB) Reaction With Mass Spectrometry to Study Unsaturated Fatty Acids in Mouse Model of Multiple Sclerosis. FRONTIERS IN CHEMISTRY. 2019;7.
816.	Chora AA, Fontoura P, Cunha A, Pais TF, Cardoso S, Ho PP, et al. Heme oxygenase-1 and carbon monoxide suppress autoimmune neuroinflammation. JOURNAL OF CLINICAL INVESTIGATION. 2007;117(2):438-47.
817.	Chora ÂA, Fontoura P, Cunha A, Pais TF, Cardoso S, Ho PP, et al. Heme oxygenase-1 and carbon monoxide suppress autoimmune neuroinflammation. Journal of Clinical Investigation. 2007.
818.	Chora AA, Fontoura P, Cunha A, Pais TF, Cardoso S, Ho PP, et al. Heme oxygenase-1 and carbon monoxide suppress autoimmune neuroinflammation. J Clin Invest. 2007;117(2):438-47.
819.	Chora AA, Fontoura P, Cunha A, Pais TF, Cardoso S, Ho PP, et al. Heme oxygenase-1 and carbon monoxide suppress autoimmune neuroinflammation. JOURNAL OF CLINICAL INVESTIGATION. 2007;117(2):438-47.
820.	Chou CM, Chou SH, Chen YC, Yang CJ. Using machine learning methods to detect physical conditions with postural balance. JOURNAL OF AMBIENT INTELLIGENCE AND HUMANIZED COMPUTING.
821.	Chou CM, Chou SH, Chen YC, Yang CJ. Using machine learning methods to detect physical conditions with postural balance. JOURNAL OF AMBIENT INTELLIGENCE AND HUMANIZED COMPUTING.
822.	Christenson RH, Russell ME, Gubar KT, Silverman LM, Ebers GC. Oligoclonal banding in cerebrospinal fluid assessed by electrophoresis on agarose after centrifugal sample concentration through a microconcentrator membrane. Clin Chem. 1985;31(10):1734-6.
823.	Chugh P. INI204Y1 Roger Riendeau April 7 th, 2014 The Liberation Treatment for Multiple Sclerosis: False Methods Lead to False Hope.
824.	Chun Z, Yongmei L, Yu O, Fajin L, Xuan C, Zhongping W. Analysis of iron deposition in the brain lesions of patients with multiple sclerosis by three dimensional enhanced T {sub 2}-star weighted angiography. Chinese Journal of Radiology. 2011;45.
825.	Cieslak M, Komoszynski M. The role of ecto-purines in inflammation leading to demyelination - new means for therapies against multiple sclerosis. NEUROLOGIA I NEUROCHIRURGIA POLSKA. 2011;45(5):489-99.
826.	Ciofu I, Ceausu I, Chirca NM, Persu C. Solifenacin Treatment After Intradetrusor Injections With Botulinum Toxin in Patients With Neurogenic Detrusor Overactivity. Am J Ther. 2022;29(5):e507-e11.
827.	Cizmarevic NS, Curko-Cofek B, Barac-Latas V, Peterlin B, Ristic S. Lack of association between C282Y and H63D polymorphisms in the hemochromatosis gene and risk of multiple sclerosis: A meta-analysis. BIOMEDICAL REPORTS. 2022;16(2).
828.	Cizmarevic NS, Curko-Cofek B, Barac-Latas V, Peterlin B, Ristic S. Lack of association between C282Y and H63D polymorphisms in the hemochromatosis gene and risk of multiple sclerosis: A meta-analysis. BIOMEDICAL REPORTS. 2022;16(2).
829.	Claes N, Dhaeze T, Fraussen J, Broux B, Van Wijmeersch B, Stinissen P, et al. Compositional Changes of B and T Cell Subtypes during Fingolimod Treatment in Multiple Sclerosis Patients: A 12-Month Follow-Up Study. PLOS ONE. 2014;9(10).
830.	Claes N, Dhaeze T, Fraussen J, Broux B, Van Wijmeersch B, Stinissen P, et al. Compositional changes of B and T cell subtypes during fingolimod treatment in multiple sclerosis patients: a 12-month follow-up study. PLoS One. 2014;9(10):e111115.
831.	Claes N, Dhaeze T, Fraussen J, Broux B, Van Wijmeersch B, Stinissen P, et al. Compositional Changes of B and T Cell Subtypes during Fingolimod Treatment in Multiple Sclerosis Patients: A 12-Month Follow-Up Study. PLOS ONE. 2014;9(10).
832.	Clarke M, Ferreira LMP, Pareto D, Arrambide G, Alberich M, Crescenzo F, et al. The diagnostic value of susceptibility-weighted imaging in multiple sclerosis: the central vein sign and iron rings are specific markers of multiple sclerosis lesions. MULTIPLE SCLEROSIS JOURNAL. 2019;25:357-.
833.	Clarke M, Ferreira LMP, Pareto D, Arrambide G, Alberich M, Crescenzo F, et al. The central vein sign and iron rings: insights from a large cohort of patients with multiple sclerosis and mimicking disorders. MULTIPLE SCLEROSIS JOURNAL. 2019;25:38-9.
834.	Clarke M, Pessini Ferreira LM, Pareto D, Arrambide G, Alberich M, Crescenzo F, et al., editors. The central vein sign and iron rings: Insights from a large cohort of patients with multiple sclerosis and mimicking disorders2019: SAGE PUBLICATIONS LTD 1 OLIVERS YARD, 55 CITY ROAD, LONDON EC1Y 1SP, ENGLAND.
835.	Clarke M, Pessini Ferreira LM, Pareto D, Arrambide G, Alberich M, Crescenzo F, et al., editors. The diagnostic value of susceptibility-weighted imaging in multiple sclerosis: the central vein sign and iron rings are specific markers of multiple sclerosis lesions2019: SAGE PUBLICATIONS LTD 1 OLIVERS YARD, 55 CITY ROAD, LONDON EC1Y 1SP, ENGLAND.
836.	Clarke MA, Pareto D, Pessini-Ferreira L, Arrambide G, Alberich M, Crescenzo F, et al. Value of 3T susceptibility-weighted imaging in the diagnosis of multiple sclerosis. American Journal of Neuroradiology. 2020.
837.	Clarke MA, Pareto D, Pessini-Ferreira L, Arrambide G, Alberich M, Crescenzo F, et al. Value of 3T Susceptibility-Weighted Imaging in the Diagnosis of Multiple Sclerosis. AJNR Am J Neuroradiol. 2020;41(6):1001-8.
838.	Claro da Silva T, Hiller C, Gai Z, Kullak-Ublick GA. Vitamin D3 transactivates the zinc and manganese transporter SLC30A10 via the Vitamin D receptor. Journal of Steroid Biochemistry and Molecular Biology. 2016.
839.	Claro da Silva T, Hiller C, Gai Z, Kullak-Ublick GA. Vitamin D3 transactivates the zinc and manganese transporter SLC30A10 via the Vitamin D receptor. J Steroid Biochem Mol Biol. 2016;163:77-87.
840.	Clasen RA. Principles of nuclear magnetic resonance as applied to brain imaging. Adv Neurol. 1990;52:511-24.
841.	Clausen J. Mercury and multiple sclerosis. Acta Neurol Scand. 1993;87(6):461-4.
842.	Clausen J, Jensen GE, Nielsen SA. Selenium in chronic neurologic diseases. Multiple sclerosis and Batten's disease. Biol Trace Elem Res. 1988;15:179-203.
843.	Cleland NRW, Al-Juboori SI, Dobrinskikh E, Bruce KD. Altered substrate metabolism in neurodegenerative disease: new insights from metabolic imaging. J Neuroinflammation. 2021;18(1):248.
844.	Clemente D, Ortega MC, Melero-Jerez C, de Castro F. The effect of glia-glia interactions on oligodendrocyte precursor cell biology during development and in demyelinating diseases. FRONTIERS IN CELLULAR NEUROSCIENCE. 2013;7.
845.	Clemente D, Ortega MC, Melero-Jerez C, de Castro F. The effect of glia-glia interactions on oligodendrocyte precursor cell biology during development and in demyelinating diseases. Frontiers in Cellular Neuroscience. 2013.
846.	Clemente D, Ortega MC, Melero-Jerez C, de Castro F. The effect of glia-glia interactions on oligodendrocyte precursor cell biology during development and in demyelinating diseases. Front Cell Neurosci. 2013;7:268.
847.	Clough M, Millist L, Lizak N, Beh S, Frohman TC, Frohman EM, et al. Ocular motor measures of cognitive dysfunction in multiple sclerosis I: inhibitory control. J Neurol. 2015;262(5):1130-7.
848.	Cobzas D, Sun H, Walsh AJ, Lebel RM, Blevins G, Wilman AH. Subcortical gray matter segmentation and voxel-based analysis using transverse relaxation and quantitative susceptibility mapping with application to multiple sclerosis. Journal of Magnetic Resonance Imaging. 2015.
849.	Cobzas D, Sun H, Walsh AJ, Lebel RM, Blevins G, Wilman AH. Subcortical gray matter segmentation and voxel-based analysis using transverse relaxation and quantitative susceptibility mapping with application to multiple sclerosis. J Magn Reson Imaging. 2015;42(6):1601-10.
850.	Cobzas D, Sun HF, Walsh AJ, Lebel RM, Blevins G, Wilman AH. Subcortical Gray Matter Segmentation and Voxel-Based Analysis Using Transverse Relaxation and Quantitative Susceptibility Mapping With Application to Multiple Sclerosis. JOURNAL OF MAGNETIC RESONANCE IMAGING. 2015;42(6):1601-10.
851.	Cobzas D, Sun HF, Walsh AJ, Lebel RM, Blevins G, Wilman AH. Subcortical Gray Matter Segmentation and Voxel-Based Analysis Using Transverse Relaxation and Quantitative Susceptibility Mapping With Application to Multiple Sclerosis. JOURNAL OF MAGNETIC RESONANCE IMAGING. 2015;42(6):1601-10.
852.	Cocuzza CE, Piazza F, Musumeci R, Oggioni D, Andreoni S, Gardinetti M, et al. Quantitative detection of epstein-barr virus DNA in cerebrospinal fluid and blood samples of patients with relapsing-remitting multiple sclerosis. PLoS One. 2014;9(4):e94497.
853.	Coda AR, Anzilotti S, Boscia F, Greco A, Panico M, Gargiulo S, et al. In vivo imaging of CNS microglial activation/macrophage infiltration with combined [F-18]DPA-714-PET and SPIO-MRI in a mouse model of relapsing remitting experimental autoimmune encephalomyelitis. EUROPEAN JOURNAL OF NUCLEAR MEDICINE AND MOLECULAR IMAGING. 2021;48(1):40-52.
854.	Coda AR, Anzilotti S, Boscia F, Greco A, Panico M, Gargiulo S, et al. In vivo imaging of CNS microglial activation/macrophage infiltration with combined [18F]DPA-714-PET and SPIO-MRI in a mouse model of relapsing remitting experimental autoimmune encephalomyelitis. European Journal of Nuclear Medicine and Molecular Imaging. 2021.
855.	Coda AR, Anzilotti S, Boscia F, Greco A, Panico M, Gargiulo S, et al. In vivo imaging of CNS microglial activation/macrophage infiltration with combined [(18)F]DPA-714-PET and SPIO-MRI in a mouse model of relapsing remitting experimental autoimmune encephalomyelitis. Eur J Nucl Med Mol Imaging. 2021;48(1):40-52.
856.	Coda AR, Anzilotti S, Boscia F, Greco A, Panico M, Gargiulo S, et al. In vivo imaging of CNS microglial activation/macrophage infiltration with combined [F-18]DPA-714-PET and SPIO-MRI in a mouse model of relapsing remitting experimental autoimmune encephalomyelitis. EUROPEAN JOURNAL OF NUCLEAR MEDICINE AND MOLECULAR IMAGING. 2021;48(1):40-52.
857.	Coe S, Tektonidis TG, Coverdale C, Penny S, Collett J, Chu BTY, et al. A cross sectional assessment of nutrient intake and the association of the inflammatory properties of nutrients and foods with symptom severity in a large cohort from the UK Multiple Sclerosis Registry. NUTRITION RESEARCH. 2021;85:31-9.
858.	Coe S, Tektonidis TG, Coverdale C, Penny S, Collett J, Chu BTY, et al. A cross sectional assessment of nutrient intake and the association of the inflammatory properties of nutrients and foods with symptom severity in a large cohort from the UK Multiple Sclerosis Registry. Nutrition Research. 2021.
859.	Coe S, Tektonidis TG, Coverdale C, Penny S, Collett J, Chu BTY, et al. A cross sectional assessment of nutrient intake and the association of the inflammatory properties of nutrients and foods with symptom severity in a large cohort from the UK Multiple Sclerosis Registry. Nutr Res. 2021;85:31-9.
860.	Coe S, Tektonidis TG, Coverdale C, Penny S, Collett J, Chu BTY, et al. A cross sectional assessment of nutrient intake and the association of the inflammatory properties of nutrients and foods with symptom severity in a large cohort from the UK Multiple Sclerosis Registry. NUTRITION RESEARCH. 2021;85:31-9.
861.	Cohen M, Bresch S, Thommel Rocchi O, Morain E, Benoit J, Levraut M, et al. Should we still only rely on EDSS to evaluate disability in multiple sclerosis patients? A study of inter and intra rater reliability. Mult Scler Relat Disord. 2021;54:103144.
862.	Cohen ME, Muja N, Fainstein N, Bulte JW, Ben-Hur T. Conserved fate and function of ferumoxides-labeled neural precursor cells in vitro and in vivo. J Neurosci Res. 2010;88(5):936-44.
863.	Cohen ME, Muja N, Fainstein N, Bulte JWM, Ben-Hur T. Conserved Fate and Function of Ferumoxides-Labeled Neural Precursor Cells In Vitro and In Vivo. JOURNAL OF NEUROSCIENCE RESEARCH. 2010;88(5):936-44.
864.	Cohen ME, Muja N, Fainstein N, Bulte JWM, Ben-Hur T. Conserved fate and function of ferumoxides-labeled neural precursor cells in vitro and in vivo. Journal of Neuroscience Research. 2010.
865.	Cohen ME, Muja N, Fainstein N, Bulte JWM, Ben-Hur T. Conserved Fate and Function of Ferumoxides-Labeled Neural Precursor Cells In Vitro and In Vivo. JOURNAL OF NEUROSCIENCE RESEARCH. 2010;88(5):936-44.
866.	Cohen R, Robinson D, Jr., Paramore C, Fraeman K, Renahan K, Bala M. Autoimmune disease concomitance among inflammatory bowel disease patients in the United States, 2001-2002. Inflamm Bowel Dis. 2008;14(6):738-43.
867.	Cojocaru IM, Socoliuc G, Sapira V, Cojocaru M. Primary Sjögren's syndrome or multiple sclerosis? Our experience concerning the dilemma of clinically isolated syndrome. Rom J Intern Med. 2011;49(4):301-18.
868.	Colaceci S, Zambri F, Marchetti F, Trivelli G, Rossi E, Petruzzo A, et al. 'A sleeping volcano that could erupt sooner or later'. Lived experiences of women with multiple sclerosis during childbearing age and motherhood: A phenomenological qualitative study. Mult Scler Relat Disord. 2021;51:102938.
869.	Colaço CB, Scadding GK, Lockhart S. Anti-cardiolipin antibodies in neurological disorders: cross-reaction with anti-single stranded DNA activity. Clin Exp Immunol. 1987;68(2):313-9.
870.	Colombel JF, Peyrin-Biroulet L. Natalizumab: a promising treatment for Crohn's disease. Expert Rev Clin Immunol. 2006;2(5):677-89.
871.	Colombo E, Triolo D, Bassani C, Bedogni F, Di Dario M, Dina G, et al. Dysregulated copper transport in multiple sclerosis may cause demyelination via astrocytes. Proc Natl Acad Sci U S A. 2021;118(27).
872.	Colonna I, Buchmann A, Damulina A, Pinter D, Helmlinger B, Pirpamer L, et al., editors. Comparison of deep grey matter iron deposition assessed by 3t mri r2 (star) relaxometry in multiple sclerosis, alzheimer's disease and in normal controls2020: SAGE PUBLICATIONS LTD 1 OLIVERS YARD, 55 CITY ROAD, LONDON EC1Y 1SP, ENGLAND.
873.	Colonna I, Buchmann A, Damulina A, Pinter D, Helmlinger B, Pirpamer L, et al. Comparison of deep grey matter iron deposition assessed by 3t mri r2(star) relaxometry in multiple sclerosis, alzheimer's disease and in normal controls. MULTIPLE SCLEROSIS JOURNAL. 2020;26(3_SUPPL):659-.
874.	Comabella M, Clarke MA, Schaedelin S, Tintore M, Pareto D, Fissolo N, et al. CSF chitinase 3-like 1 is associated with iron rims in patients with a first demyelinating event. MULTIPLE SCLEROSIS JOURNAL. 2022;28(1):71-81.
875.	Comabella M, Clarke MA, Schaedelin S, Tintoré M, Pareto D, Fissolo N, et al. CSF chitinase 3-like 1 is associated with iron rims in patients with a first demyelinating event. Multiple Sclerosis Journal. 2022.
876.	Comabella M, Clarke MA, Schaedelin S, Tintoré M, Pareto D, Fissolo N, et al. CSF chitinase 3-like 1 is associated with iron rims in patients with a first demyelinating event. Mult Scler. 2022;28(1):71-81.
877.	Comabella M, Clarke MA, Schaedelin S, Tintore M, Pareto D, Fissolo N, et al. CSF chitinase 3-like 1 is associated with iron rims in patients with a first demyelinating event. MULTIPLE SCLEROSIS JOURNAL. 2022;28(1):71-81.
878.	Comabella M, Craig DW, Morcillo-Suarez C, Rio J, Navarro A, Fernandez M, et al. Genome-wide Scan of 500 000 Single-Nucleotide Polymorphisms Among Responders and Nonresponders to Interferon Beta Therapy in Multiple Sclerosis. ARCHIVES OF NEUROLOGY. 2009;66(8):972-8.
879.	Comabella M, Craig DW, Morcillo-Suárez C, Río J, Navarro A, Fernández M, et al. Genome-wide scan of 500 000 single-nucleotide polymorphisms among responders and nonresponders to interferon beta therapy in multiple sclerosis. Archives of Neurology. 2009.
880.	Comabella M, Craig DW, Morcillo-Suárez C, Río J, Navarro A, Fernández M, et al. Genome-wide scan of 500,000 single-nucleotide polymorphisms among responders and nonresponders to interferon beta therapy in multiple sclerosis. Arch Neurol. 2009;66(8):972-8.
881.	Comabella M, Craig DW, Morcillo-Suarez C, Rio J, Navarro A, Fernandez M, et al. Genome-wide Scan of 500 000 Single-Nucleotide Polymorphisms Among Responders and Nonresponders to Interferon Beta Therapy in Multiple Sclerosis. ARCHIVES OF NEUROLOGY. 2009;66(8):972-8.
882.	Comes G, Fernandez-Gayol O, Molinero A, Giralt M, Capdevila M, Atrian S, et al. Mouse metallothionein-1 and metallothionein-2 are not biologically interchangeable in an animal model of multiple sclerosis, EAE. METALLOMICS. 2019;11(2):327-37.
883.	Comes G, Fernandez-Gayol O, Molinero A, Giralt M, Capdevila M, Atrian S, et al. Mouse metallothionein-1 and metallothionein-2 are not biologically interchangeable in an animal model of multiple sclerosis, EAE. Metallomics. 2019.
884.	Comes G, Fernandez-Gayol O, Molinero A, Giralt M, Capdevila M, Atrian S, et al. Mouse metallothionein-1 and metallothionein-2 are not biologically interchangeable in an animal model of multiple sclerosis, EAE. Metallomics. 2019;11(2):327-37.
885.	Comes G, Fernandez-Gayol O, Molinero A, Giralt M, Capdevila M, Atrian S, et al. Mouse metallothionein-1 and metallothionein-2 are not biologically interchangeable in an animal model of multiple sclerosis, EAE. METALLOMICS. 2019;11(2):327-37.
886.	Cone W, Russel C, Harwood RU. Lead as a possible cause of multiple sclerosis. Archives of Neurology & Psychiatry. 1934;31(2):236-69.
887.	Cone W, Russel C, Harwood RU. Lead and Multiple Sclerosis. The Journal of Nervous and Mental Disease. 1934;80(3):344-5.
888.	Connell L, Daws R, Hampshire A, Nicholas R, Raffel J. Validating a participant-led computerised cognitive battery in people with multiple sclerosis. MULTIPLE SCLEROSIS JOURNAL. 2016;22:140-1.
889.	Constantinescu C. Relationship between iron accumulation and white matter injury in multiple sclerosis: a case-control study. Current Medical Literature. 2015;7(1):12.
890.	Conti A, Treaba CA, Mehndiratta A, Barletta VT, Mainero C, Toschi N. An Interpretable Machine Learning Model to Predict Cortical Atrophy in Multiple Sclerosis. Brain Sci. 2023;13(2).
891.	Conway DS, Briggs FB, Mowry EM, Fitzgerald KC, Hersh CM. Racial disparities in hypertension management among multiple sclerosis patients. Mult Scler Relat Disord. 2022;64:103972.
892.	Cooper G, Chien C, Zimmermann H, Bellmann-Strobl J, Ruprecht K, Kuchling J, et al. Longitudinal analysis of T1w/T2w ratio in patients with multiple sclerosis from first clinical presentation. Mult Scler. 2021;27(14):2180-90.
893.	Corbin JG, Kelly D, Rath EM, Baerwald KD, Suzuki K, Popko B. Targeted CNS expression of interferon-gamma in transgenic mice leads to hypomyelination, reactive gliosis, and abnormal cerebellar development. MOLECULAR AND CELLULAR NEUROSCIENCE. 1996;7(5):354-70.
894.	Corbin JG, Kelly D, Rath EM, Baerwald KD, Suzuki K, Popko B. Targeted CNS expression of interferon-gamma in transgenic mice leads to hypomyelination, reactive gliosis, and abnormal cerebellar development. MOLECULAR AND CELLULAR NEUROSCIENCE. 1996;7(5):354-70.
895.	Cordioli C, Callari G, Fantozzi R, Caruso F, Martucci G, Mascara S, et al. Pegylated interferon beta-1a (Plegridy) Italian real-world experience: a Delphi analysis of injection-site reaction and flu-like symptom management. Neurol Sci. 2021;42(4):1515-21.
896.	Cornelison C, Fadel S. Clickable Biomaterials for Modulating Neuroinflammation. Int J Mol Sci. 2022;23(15).
897.	Corot C, Petry KG, Trivedi R, Saleh A, Jonkmanns C, Le Bas JF, et al. Macrophage imaging in central nervous system and in carotid atherosclerotic plaque using ultrasmall superparamagnetic iron oxide in magnetic resonance imaging. INVESTIGATIVE RADIOLOGY. 2004;39(10):619-25.
898.	Corot C, Petry KG, Trivedi R, Saleh A, Jonkmanns C, Le Bas JF, et al. Macrophage imaging in central nervous system and in carotid atherosclerotic plaque using ultrasmall superparamagnetic iron oxide in magnetic resonance imaging. Investigative Radiology. 2004.
899.	Corot C, Petry KG, Trivedi R, Saleh A, Jonkmanns C, Le Bas JF, et al. Macrophage imaging in central nervous system and in carotid atherosclerotic plaque using ultrasmall superparamagnetic iron oxide in magnetic resonance imaging. Invest Radiol. 2004;39(10):619-25.
900.	Corrales GP, Lopez CV, Payero MA. EFFECT OF PHARMACOLOGIC TREATMENT OF THE NUTRITIONAL STATUS OF NEUROLOGIC PATIENTS. NUTRICION HOSPITALARIA. 2014;29:47-56.
901.	Corrales GP, López CV, Payero MA. Effect of pharmacologic treatment of the nutritional status of neurologic patients. Nutricion Hospitalaria. 2014.
902.	Corrales GP, Lopez CV, Payero MA. EFFECT OF PHARMACOLOGIC TREATMENT OF THE NUTRITIONAL STATUS OF NEUROLOGIC PATIENTS. NUTRICION HOSPITALARIA. 2014;29:47-56.
903.	Correale J, Chiquete E, Milojevic S, Frider N, Bajusz I. Assessing the potential impact of non-proprietary drug copies on quality of medicine and treatment in patients with relapsing multiple sclerosis: the experience with fingolimod. DRUG DESIGN DEVELOPMENT AND THERAPY. 2014;8:859-67.
904.	Correale J, Chiquete E, Milojevic S, Frider N, Bajusz I. Assessing the potential impact of non-proprietary drug copies on quality of medicine and treatment in patients with relapsing multiple sclerosis: The experience with fingolimod. Drug Design, Development and Therapy. 2014.
905.	Correale J, Chiquete E, Milojevic S, Frider N, Bajusz I. Assessing the potential impact of non-proprietary drug copies on quality of medicine and treatment in patients with relapsing multiple sclerosis: the experience with fingolimod. Drug Des Devel Ther. 2014;8:859-67.
906.	Correale J, Chiquete E, Milojevic S, Frider N, Bajusz I. Assessing the potential impact of non-proprietary drug copies on quality of medicine and treatment in patients with relapsing multiple sclerosis: the experience with fingolimod. DRUG DESIGN DEVELOPMENT AND THERAPY. 2014;8:859-67.
907.	Cortese I, Capone S, Luchetti S, Cortese R, Nicosia A. Cross-reactive phage-displayed mimotopes lead to the discovery of mimicry between HSV-1 and a brain-specific protein. JOURNAL OF NEUROIMMUNOLOGY. 2001;113(1):119-28.
908.	Cortese I, Capone S, Luchetti S, Cortese R, Nicosia A. Cross-reactive phage-displayed mimotopes lead to the discovery of mimicry between HSV-1 and a brain-specific protein. JOURNAL OF NEUROIMMUNOLOGY. 2001;113(1):119-28.
909.	Cortese M, Chitnis T, Ascherio A, Munger KL. Total intake of different minerals and the risk of multiple sclerosis. NEUROLOGY. 2019;92(18):E2127-E35.
910.	Cortese M, Chitnis T, Ascherio A, Munger KL. Total intake of different minerals and the risk of multiple sclerosis. Neurology. 2019.
911.	Cortese M, Chitnis T, Ascherio A, Munger KL. Total intake of different minerals and the risk of multiple sclerosis. Neurology. 2019;92(18):e2127-e35.
912.	Cortese M, Chitnis T, Ascherio A, Munger KL. Total intake of different minerals and the risk of multiple sclerosis. NEUROLOGY. 2019;92(18):E2127-E35.
913.	Cortese R, Prosperini L, Stasolla A, Haggiag S, Villani V, Simone IL, et al. Clinical course of central nervous system demyelinating neurological adverse events associated with anti-TNF therapy. J Neurol. 2021;268(8):2895-9.
914.	Cory-Slechta DA, Sobolewski M, Oberdorster G. Air Pollution-Related Brain Metal Dyshomeostasis as a Potential Risk Factor for Neurodevelopmental Disorders and Neurodegenerative Diseases. ATMOSPHERE. 2020;11(10).
915.	Cory‐slechta DA, Sobolewski M, Oberdörster G. Air pollution‐related brain metal dyshomeostasis as a potential risk factor for neurodevelopmental disorders and neurodegenerative diseases. Atmosphere. 2020.
916.	Costa I, Barbosa DJ, Benfeito S, Silva V, Chavarria D, Borges F, et al. Molecular mechanisms of ferroptosis and their involvement in brain diseases. Pharmacol Ther. 2023;244:108373.
917.	Coyle PK. What Can We Learn from Sex Differences in MS? J Pers Med. 2021;11(10).
918.	Craelius W, Jacobs RM, Jones AOL. Mineral Composition of Brains of Normal and Multiple Sclerosis Victims. Proceedings of the Society for Experimental Biology and Medicine. 1980.
919.	Craelius W, Migdal MW, Luessenhop CP, Sugar A, Mihalakis I. Iron deposits surrounding multiple sclerosis plaques. Archives of Pathology and Laboratory Medicine. 1982.
920.	Craelius W, Migdal MW, Luessenhop CP, Sugar A, Mihalakis I. IRON DEPOSITS SURROUNDING MULTIPLE-SCLEROSIS PLAQUES. ARCHIVES OF PATHOLOGY & LABORATORY MEDICINE. 1982;106(8):397-9.
921.	Craelius W, Migdal MW, Luessenhop CP, Sugar A, Mihalakis I. Iron deposits surrounding multiple sclerosis plaques. Arch Pathol Lab Med. 1982;106(8):397-9.
922.	Craelius W, Migdal MW, Luessenhop CP, Sugar A, Mihalakis I. Iron deposits surrounding multiple sclerosis plaques. Archives of pathology & laboratory medicine. 1982;106(8):397-9.
923.	Craig K, Avila P, Brown DA. Design, Synthesis, and Anti-Neuroinflammatory Activity of Amide- Containing Dithiolethiones. Med Chem. 2021;17(10):1166-74.
924.	Crawford L, Loprinzi PD. Effects of Exercise on Memory Interference in Neuropsychiatric Disorders. Adv Exp Med Biol. 2020;1228:425-38.
925.	Creange A, Lefaucheur JP, Balleyguier MO, Galacteros F. Iron depletion induced by bloodletting and followed by rhEPO administration as a therapeutic strategy in progressive multiple sclerosis: A pilot, open-label study with neurophysiological measurements. NEUROPHYSIOLOGIE CLINIQUE-CLINICAL NEUROPHYSIOLOGY. 2013;43(5-6):303-12.
926.	Créange A, Lefaucheur JP, Balleyguier MO, Galactéros F. Iron depletion induced by bloodletting and followed by rhEPO administration as a therapeutic strategy in progressive multiple sclerosis: A pilot, open-label study with neurophysiological measurements. Neurophysiologie Clinique. 2013.
927.	Creange A, Lefaucheur JP, Balleyguier MO, Galacteros F. Iron depletion induced by bloodletting and followed by rhEPO administration as a therapeutic strategy in progressive multiple sclerosis: A pilot, open-label study with neurophysiological measurements. NEUROPHYSIOLOGIE CLINIQUE-CLINICAL NEUROPHYSIOLOGY. 2013;43(5-6):303-12.
928.	Créange A, Lefaucheur JP, Balleyguier MO, Galactéros F. Iron depletion induced by bloodletting and followed by rhEPO administration as a therapeutic strategy in progressive multiple sclerosis: a pilot, open-label study with neurophysiological measurements. Neurophysiol Clin. 2013;43(5-6):303-12.
929.	Creange A, Lefaucheur JP, Balleyguier MO, Galacteros F. Iron depletion induced by bloodletting and followed by rhEPO administration as a therapeutic strategy in progressive multiple sclerosis: A pilot, open-label study with neurophysiological measurements. NEUROPHYSIOLOGIE CLINIQUE-CLINICAL NEUROPHYSIOLOGY. 2013;43(5-6):303-12.
930.	Créange A, Lefaucheur JP, Balleyguier MO, Galactéros F. Iron depletion induced by bloodletting and followed by rhEPO administration as a therapeutic strategy in progressive multiple sclerosis: a pilot, open-label study with neurophysiological measurements. Neurophysiologie Clinique/Clinical Neurophysiology. 2013;43(5-6):303-12.
931.	Cree BA, Cohen JA, Reder AT, Tomic D, Silva D, Piani Meier D, et al. Disability improvement as a clinically relevant outcome in clinical trials of relapsing forms of multiple sclerosis. Mult Scler. 2021;27(14):2219-31.
932.	Crescenzo F, Clarke M, Tamanti A, Pareto D, Pizzini F, Alberich M, et al., editors. Susceptibility weighted imaging in relapsing-remitting multiple sclerosis: an exploratory study comparing the detection of iron rims using two different 3T MRI pulse sequences2019: SAGE PUBLICATIONS LTD 1 OLIVERS YARD, 55 CITY ROAD, LONDON EC1Y 1SP, ENGLAND.
933.	Crescenzo F, Clarke M, Tamanti A, Pareto D, Pizzini F, Alberich M, et al. Susceptibility weighted imaging in relapsing- remitting multiple sclerosis: an exploratory study comparing the detection of iron rims using two different 3T MRI pulse sequences. MULTIPLE SCLEROSIS JOURNAL. 2019;25:677-8.
934.	Crielaard BJ, Lammers T, Morgan ME, Chaabane L, Carboni S, Greco B, et al. Macrophages and liposomes in inflammatory disease: friends or foes? Int J Pharm. 2011;416(2):499-506.
935.	Cronin MJ, Wharton S, Al-Radaideh A, Constantinescu C, Evangelou N, Bowtell R, et al. A comparison of phase imaging and quantitative susceptibility mapping in the imaging of multiple sclerosis lesions at ultrahigh field. Magma. 2016;29(3):543-57.
936.	Cross AK, Woodroofe MN. Chemokine modulation of matrix metalloproteinase and TIMP production in adult rat brain microglia and a human microglial cell line in vitro. GLIA. 1999;28(3):183-9.
937.	Cross AK, Woodroofe MN. Chemokine modulation of matrix metalloproteinase and TIMP production in adult rat brain microglia and a human microglial cell line in vitro. GLIA. 1999.
938.	Cross AK, Woodroofe MN. Chemokine modulation of matrix metalloproteinase and TIMP production in adult rat brain microglia and a human microglial cell line in vitro. Glia. 1999;28(3):183-9.
939.	Cross AK, Woodroofe MN. Chemokine modulation of matrix metalloproteinase and TIMP production in adult rat brain microglia and a human microglial cell line in vitro. GLIA. 1999;28(3):183-9.
940.	Cuadrado A, Rojo AI. Heme oxygenase-1 as a therapeutic target in neurodegenerative diseases and brain infections. Curr Pharm Des. 2008;14(5):429-42.
941.	Cullimore DR. Observations on the bacterial populations recovered from water wells subjected to disinfection. Canadian Water Resources Journal. 1989.
942.	Cunnane SC, Ho SY, Dore-Duffy P, Ells KR, Horrobin DF. Essential fatty acid and lipid profiles in plasma and erythrocytes in patients with multiple sclerosis. American Journal of Clinical Nutrition. 1989.
943.	Cunnane SC, Ho SY, Dore-Duffy P, Ells KR, Horrobin DF. Essential fatty acid and lipid profiles in plasma and erythrocytes in patients with multiple sclerosis. Am J Clin Nutr. 1989;50(4):801-6.
944.	Cunnane SC, Manku MS, Horrobin DF. The pineal and regulation of fibrosis: Pinealectomy as a model of primary biliary cirrhosis: Roles of melatonin and prostaglandins in fibrosis and regulation of T lymphocytes. Medical Hypotheses. 1979.
945.	Cunnane SC, Manku MS, Horrobin DF. The pineal and regulation of fibrosis: pinealectomy as a model of primary biliary cirrhosis: roles of melatonin and prostaglandins in fibrosis and regulation of T lymphocytes. Med Hypotheses. 1979;5(4):403-14.
946.	Ćurko-Cofek B, Grubić Kezele T, Barac-Latas V. Hepcidin and metallothioneins as molecular base for sex-dependent differences in clinical course of experimental autoimmune encephalomyelitis in chronic iron overload. Medical Hypotheses. 2017.
947.	Ćurko-Cofek B, Grubić Kezele T, Barac-Latas V. Hepcidin and metallothioneins as molecular base for sex-dependent differences in clinical course of experimental autoimmune encephalomyelitis in chronic iron overload. Med Hypotheses. 2017;107:51-4.
948.	Curko-Cofek B, Kezele TG, Barac-Latas V. Hepcidin and metallothioneins as molecular base for sex-dependent differences in clinical course of experimental autoimmune encephalomyelitis in chronic iron overload. MEDICAL HYPOTHESES. 2017;107:51-4.
949.	Curko-Cofek B, Kezele TG, Barac-Latas V. Hepcidin and metallothioneins as molecular base for sex-dependent differences in clinical course of experimental autoimmune encephalomyelitis in chronic iron overload. MEDICAL HYPOTHESES. 2017;107:51-4.
950.	Curko-Cofek B, Kezele TG, Marinic J, Tota M, Cizmarevic NS, Milin C, et al. Chronic iron overload induces gender-dependent changes in iron homeostasis, lipid peroxidation and clinical course of experimental autoimmune encephalomyelitis. NEUROTOXICOLOGY. 2016;57:1-12.
951.	Ćurko-Cofek B, Kezele TG, Marinić J, Tota M, Čizmarević NS, Milin Č, et al. Chronic iron overload induces gender-dependent changes in iron homeostasis, lipid peroxidation and clinical course of experimental autoimmune encephalomyelitis. NeuroToxicology. 2016.
952.	Ćurko-Cofek B, Kezele TG, Marinić J, Tota M, Čizmarević NS, Milin Č, et al. Chronic iron overload induces gender-dependent changes in iron homeostasis, lipid peroxidation and clinical course of experimental autoimmune encephalomyelitis. Neurotoxicology. 2016;57:1-12.
953.	Curko-Cofek B, Kezele TG, Marinic J, Tota M, Cizmarevic NS, Milin C, et al. Chronic iron overload induces gender-dependent changes in iron homeostasis, lipid peroxidation and clinical course of experimental autoimmune encephalomyelitis. NEUROTOXICOLOGY. 2016;57:1-12.
954.	Cusick MF, Libbey JE, Fujinami RS. Picornavirus infection leading to immunosuppression. FUTURE VIROLOGY. 2014;9(5):475-82.
955.	Cusick MF, Libbey JE, Trede NS, Fujinami RS. Targeting Insulin-Like Growth Factor 1 Leads to Amelioration of Inflammatory Demyelinating Disease. PLOS ONE. 2014;9(4).
956.	Cusick MF, Libbey JE, Trede NS, Fujinami RS. Targeting Insulin-Like Growth Factor 1 Leads to Amelioration of Inflammatory Demyelinating Disease. PLOS ONE. 2014;9(4).
957.	Członkowska A, Półtorak M, Cendrowski W, Korlak J. Sensitization of cerebrospinal fluid and peripheral blood lymphocytes to myelin basic protein in multiple sclerosis. Acta Neurol Scand. 1982;66(1):121-9.
958.	D'Elios MM, Aldinucci A, Amoriello R, Benagiano M, Bonechi E, Maggi P, et al. Myelin-specific T cells carry and release magnetite PGLA-PEG COOH nanoparticles in the mouse central nervous system. RSC ADVANCES. 2018;8(2):904-13.
959.	D'Elios MM, Aldinucci A, Amoriello R, Benagiano M, Bonechi E, Maggi P, et al. Myelin-specific T cells carry and release magnetite PGLA-PEG COOH nanoparticles in the mouse central nervous system. RSC Advances. 2018.
960.	D'Elios MM, Aldinucci A, Amoriello R, Benagiano M, Bonechi E, Maggi P, et al. Myelin-specific T cells carry and release magnetite PGLA-PEG COOH nanoparticles in the mouse central nervous system. RSC Adv. 2018;8(2):904-13.
961.	D'Elios MM, Aldinucci A, Amoriello R, Benagiano M, Bonechi E, Maggi P, et al. Myelin-specific T cells carry and release magnetite PGLA-PEG COOH nanoparticles in the mouse central nervous system. RSC ADVANCES. 2018;8(2):904-13.
962.	D'Haens G, Sandborn WJ, Colombel JF, Rutgeerts P, Brown K, Barkay H, et al. A phase II study of laquinimod in Crohn's disease. Gut. 2015;64(8):1227-35.
963.	D'Souza J, Biswas A, Gada P, Mangroliya J, Natarajan M. Barriers leading to increased disability in neurologically challenged populations during COVID-19 pandemic: a scoping review. DISABILITY AND REHABILITATION.
964.	D'Souza J, Biswas A, Gada P, Mangroliya J, Natarajan M. Barriers leading to increased disability in neurologically challenged populations during COVID-19 pandemic: a scoping review. DISABILITY AND REHABILITATION.
965.	da Silva TC, Hiller C, Gai ZB, Kullak-Ublick GA. Vitamin D-3 transactivates the zinc and manganese transporter SLC30A10 via the Vitamin D receptor. JOURNAL OF STEROID BIOCHEMISTRY AND MOLECULAR BIOLOGY. 2016;163:77-87.
966.	da Silva TC, Hiller C, Gai ZB, Kullak-Ublick GA. Vitamin D-3 transactivates the zinc and manganese transporter SLC30A10 via the Vitamin D receptor. JOURNAL OF STEROID BIOCHEMISTRY AND MOLECULAR BIOLOGY. 2016;163:77-87.
967.	Daglas M, Adlard PA. The Involvement of Iron in Traumatic Brain Injury and Neurodegenerative Disease. FRONTIERS IN NEUROSCIENCE. 2018;12.
968.	Daglas M, Adlard PA. The Involvement of Iron in Traumatic Brain Injury and Neurodegenerative Disease. Frontiers in Neuroscience. 2018.
969.	Daglas M, Adlard PA. The Involvement of Iron in Traumatic Brain Injury and Neurodegenerative Disease. Front Neurosci. 2018;12:981.
970.	Dahl K, Turner T, Vasdev N. Radiosynthesis of a Bruton's tyrosine kinase inhibitor, [(11) C]Tolebrutinib, via palladium-NiXantphos-mediated carbonylation. J Labelled Comp Radiopharm. 2020;63(11):482-7.
971.	Dahl S. [Occurrence of multiple sclerosis (MS; sclerosis disseminata) in various age groups]. Z Alternsforsch. 1971;24(4):333-42.
972.	Dakanalis A, Tryfonos C, Pavlidou E, Vadikolias K, Papadopoulou SK, Alexatou O, et al. Associations between Mediterranean Diet Adherence, Quality of Life, and Mental Health in Patients with Multiple Sclerosis: A Cross-Sectional Study. J Pers Med. 2024;14(2).
973.	Dal-Bianco A, Grabner G, Kronnerwetter C, Weber M, Berger T, Leutmezer F, et al., editors. Iron rim lesions in multiple sclerosis at 7 Tesla magnetic resonance imaging: A 7 year prospective longitudinal study2019: SAGE PUBLICATIONS LTD 1 OLIVERS YARD, 55 CITY ROAD, LONDON EC1Y 1SP, ENGLAND.
974.	Dal-Bianco A, Grabner G, Kronnerwetter C, Weber M, Berger T, Leutmezer F, et al. Iron rim lesions in multiple sclerosis at 7 Tesla magnetic resonance imaging: a 7 year prospective longitudinal study. MULTIPLE SCLEROSIS JOURNAL. 2019;25:50-1.
975.	Dal-Bianco A, Grabner G, Kronnerwetter C, Weber M, Höftberger R, Berger T, et al. Slow expansion of multiple sclerosis iron rim lesions: pathology and 7 T magnetic resonance imaging. Acta neuropathologica. 2017;133(1):25-42.
976.	Dal-Bianco A, Grabner G, Kronnerwetter C, Weber M, Hoftberger R, Berger T, et al. Slow expansion of multiple sclerosis iron rim lesions: pathology and 7 T magnetic resonance imaging. ACTA NEUROPATHOLOGICA. 2017;133(1):25-42.
977.	Dal-Bianco A, Grabner G, Kronnerwetter C, Weber M, Höftberger R, Berger T, et al. Slow expansion of multiple sclerosis iron rim lesions: pathology and 7 T magnetic resonance imaging. Acta Neuropathologica. 2017.
978.	Dal-Bianco A, Grabner G, Kronnerwetter C, Weber M, Hoftberger R, Berger T, et al. Slow expansion of multiple sclerosis iron rim lesions: pathology and 7 T magnetic resonance imaging. ACTA NEUROPATHOLOGICA. 2017;133(1):25-42.
979.	Dal-Bianco A, Grabner G, Kronnerwetter C, Weber M, Höftberger R, Berger T, et al. Slow expansion of multiple sclerosis iron rim lesions: pathology and 7 T magnetic resonance imaging. Acta Neuropathol. 2017;133(1):25-42.
980.	Dal-Bianco A, Grabner G, Kronnerwetter C, Weber M, Hoftberger R, Berger T, et al. Slow expansion of multiple sclerosis iron rim lesions: pathology and 7 T magnetic resonance imaging. ACTA NEUROPATHOLOGICA. 2017;133(1):25-42.
981.	Dal-Bianco A, Grabner G, Kronnerwetter C, Weber M, Kornek B, Kasprian G, et al. Long-term evolution of multiple sclerosis iron rim lesions in 7 T MRI. Brain. 2021;144(3):833-47.
982.	Dal-Bianco A, Grabner G, Kronnerwetter C, Weber M, Kornek B, Kasprian G, et al. Long-term evolution of multiple sclerosis iron rim lesions in 7 T MRI. BRAIN. 2021;144:833-47.
983.	Dal-Bianco A, Grabner G, Kronnerwetter C, Weber M, Kornek B, Kasprian G, et al. Long-term evolution of multiple sclerosis iron rim lesions in 7 T MRI. Brain. 2021.
984.	Dal-Bianco A, Grabner G, Kronnerwetter C, Weber M, Kornek B, Kasprian G, et al. Long-term evolution of multiple sclerosis iron rim lesions in 7 T MRI. BRAIN. 2021;144:833-47.
985.	Dal-Bianco A, Grabner G, Kronnerwetter C, Weber M, Kornek B, Kasprian G, et al. Long-term evolution of multiple sclerosis iron rim lesions in 7 T MRI. Brain. 2021;144(3):833-47.
986.	Dal-Bianco A, Grabner G, Kronnerwetter C, Weber M, Kornek B, Kasprian G, et al. Long-term evolution of multiple sclerosis iron rim lesions in 7 T MRI. BRAIN. 2021;144:833-47.
987.	Dal-Bianco A, Kolbrink S, Pusswald G, Grabner G, Kronnerwetter C, Reiter G, et al. Do 7T observed iron rim lesions in patients with multiple sclerosis serve as a marker for neuropsychological deficits? MULTIPLE SCLEROSIS JOURNAL. 2019;25:732-.
988.	Dal-Bianco A, Schranzer R, Grabner G, Lanzinger M, Kolbrink S, Pusswald G, et al. Iron Rims in Patients With Multiple Sclerosis as Neurodegenerative Marker? A 7-Tesla Magnetic Resonance Study. Frontiers in neurology. 2021;12.
989.	Dal-Bianco A, Schranzer R, Grabner G, Lanzinger M, Kolbrink S, Pusswald G, et al. Iron Rims in Patients With Multiple Sclerosis as Neurodegenerative Marker? A 7-Tesla Magnetic Resonance Study. FRONTIERS IN NEUROLOGY. 2021;12.
990.	Dal-Bianco A, Schranzer R, Grabner G, Lanzinger M, Kolbrink S, Pusswald G, et al. Iron Rims in Patients With Multiple Sclerosis as Neurodegenerative Marker? A 7-Tesla Magnetic Resonance Study. Frontiers in Neurology. 2021.
991.	Dal-Bianco A, Schranzer R, Grabner G, Lanzinger M, Kolbrink S, Pusswald G, et al. Iron Rims in Patients With Multiple Sclerosis as Neurodegenerative Marker? A 7-Tesla Magnetic Resonance Study. FRONTIERS IN NEUROLOGY. 2021;12.
992.	Dal-Bianco A, Schranzer R, Grabner G, Lanzinger M, Kolbrink S, Pusswald G, et al. Iron Rims in Patients With Multiple Sclerosis as Neurodegenerative Marker? A 7-Tesla Magnetic Resonance Study. Front Neurol. 2021;12:632749.
993.	Dal-Bianco A, Schranzer R, Grabner G, Lanzinger M, Kolbrink S, Pusswald G, et al. Iron Rims in Patients With Multiple Sclerosis as Neurodegenerative Marker? A 7-Tesla Magnetic Resonance Study. Front Neurol. 2021;12:632749.
994.	Dal-Bianco A, Schranzer R, Grabner G, Lanzinger M, Kolbrink S, Pusswald G, et al. Iron Rims in Patients With Multiple Sclerosis as Neurodegenerative Marker? A 7-Tesla Magnetic Resonance Study. FRONTIERS IN NEUROLOGY. 2021;12.
995.	Dalla Costa G, Leocani L, Comi G. Ofatumumab subcutaneous injection for the treatment of relapsing forms of multiple sclerosis. Expert Rev Clin Immunol. 2022;18(2):105-14.
996.	Dalton CM, Bodini B, Samson RS, Battaglini M, Fisniku LK, Thompson AJ, et al. Brain lesion location and clinical status 20 years after a diagnosis of clinically isolated syndrome suggestive of multiple sclerosis. Mult Scler. 2012;18(3):322-8.
997.	Damiano S, La Rosa G, Sozio C, Cavaliere G, Trinchese G, Raia M, et al. 5-Hydroxytryptamine Modulates Maturation and Mitochondria Function of Human Oligodendrocyte Progenitor M03-13 Cells. Int J Mol Sci. 2021;22(5).
998.	Damiano S, Sasso A, De Felice B, Terrazzano G, Bresciamorra V, Carotenuto A, et al. The IFN-β 1b effect on Cu Zn superoxide dismutase (SOD1) in peripheral mononuclear blood cells of relapsing-remitting multiple sclerosis patients and in neuroblastoma SK-N-BE cells. Brain Res Bull. 2015;118:1-6.
999.	Damuzzo V, Agnoletto L, Rampazzo R, Cammalleri F, Cancanelli L, Chiumente M, et al. The QOSMOS Study: Pharmacist-Led Multicentered Observational Study on Quality of Life in Multiple Sclerosis. NEUROLOGY INTERNATIONAL. 2021;13(4):682-94.
1000.	Damuzzo V, Agnoletto L, Rampazzo R, Cammalleri F, Cancanelli L, Chiumente M, et al. The QOSMOS Study: Pharmacist-Led Multicentered Observational Study on Quality of Life in Multiple Sclerosis. NEUROLOGY INTERNATIONAL. 2021;13(4):682-94.
1001.	Damuzzo V, Agnoletto L, Rampazzo R, Cammalleri F, Cancanelli L, Chiumente M, et al. The QOSMOS Study: Pharmacist-Led Multicentered Observational Study on Quality of Life in Multiple Sclerosis. NEUROLOGY INTERNATIONAL. 2021;13(4):682-94.
1002.	Danielczyk W. D penicillamine in the treatment of multiple sclerosis. Therapiewoche. 1973.
1003.	Danikowski KM, Jayaraman S, Prabhakar BS. Regulatory T cells in multiple sclerosis and myasthenia gravis. J Neuroinflammation. 2017;14(1):117.
1004.	Darazam IA, Rabiei MM, Moradi O, Gharehbagh FJ, Roozbeh M, Nourinia R, et al. A Case of Fingolimod-associated Cryptococcal Meningitis. Curr HIV Res. 2022;20(4):337-42.
1005.	Darbinian N, Selzer ME. Oligodendrocyte pathology in fetal alcohol spectrum disorders. Neural Regen Res. 2022;17(3):497-502.
1006.	Das J, Chauhan VD, Mills D, Johal NJ, Tan M, Matthews R, et al. Therapeutic plasma exchange in neurological disorders: Experience from a tertiary neuroscience centre. Transfus Apher Sci. 2019;58(6):102654.
1007.	Dastoorpoor M, Zamanian M, Moradzadeh R, Nabavi SM, Kousari R. Prevalence of sexual dysfunction in men with multiple sclerosis: a systematic review and meta-analysis. Syst Rev. 2021;10(1):10.
1008.	Daugherty KK, Butler JS, Mattingly M, Ryan M. Factors leading patients to discontinue multiple sclerosis therapies. JOURNAL OF THE AMERICAN PHARMACISTS ASSOCIATION. 2005;45(3):371-5.
1009.	Daugherty KK, Butler JS, Mattingly M, Ryan M. Factors leading patients to discontinue multiple sclerosis therapies. JOURNAL OF THE AMERICAN PHARMACISTS ASSOCIATION. 2005;45(3):371-5.
1010.	Daugherty KK, Butler JS, Mattingly M, Ryan M. Factors leading patients to discontinue multiple sclerosis therapies. JOURNAL OF THE AMERICAN PHARMACISTS ASSOCIATION. 2005;45(3):371-5.
1011.	David Ruban S, Christina Hilt C, Petersen T. Quality of life in multiple sclerosis: The differential impact of motor and cognitive fatigue. Mult Scler J Exp Transl Clin. 2021;7(1):2055217321996040.
1012.	David S, Jhelum P, Ryan F, Jeong SY, Kroner A. Dysregulation of Iron Homeostasis in the Central Nervous System and the Role of Ferroptosis in Neurodegenerative Disorders. ANTIOXIDANTS & REDOX SIGNALING.
1013.	David S, Jhelum P, Ryan F, Jeong SY, Kroner A. Dysregulation of Iron Homeostasis in the Central Nervous System and the Role of Ferroptosis in Neurodegenerative Disorders. ANTIOXIDANTS & REDOX SIGNALING.
1014.	David S, Jhelum P, Ryan F, Jeong SY, Kroner A. Dysregulation of Iron Homeostasis in the Central Nervous System and the Role of Ferroptosis in Neurodegenerative Disorders. Antioxid Redox Signal. 2022;37(1-3):150-70.
1015.	Davies BE. TRACE-ELEMENTS IN THE HUMAN ENVIRONMENT - PROBLEMS AND RISKS. ENVIRONMENTAL GEOCHEMISTRY AND HEALTH. 1994;16(3-4):97-106.
1016.	Davies BE. Trace elements in the human environment: Problems and risks. Environmental Geochemistry and Health. 1994.
1017.	Davies BE. Trace elements in the human environment: Problems and risks. Environ Geochem Health. 1994;16(3-4):97-106.
1018.	Davies BE. TRACE-ELEMENTS IN THE HUMAN ENVIRONMENT - PROBLEMS AND RISKS. ENVIRONMENTAL GEOCHEMISTRY AND HEALTH. 1994;16(3-4):97-106.
1019.	Davis JM, Davis KR, Newhouse J, Pfister RC. Expanded high iodine dose in computed cranial tomography: a preliminary report. Radiology. 1979;131(2):373-80.
1020.	Davis SL, Korkmas MA, Crandall CG, Frohman EM. Impaired sweating in multiple sclerosis leads to increased reliance on skin blood flow for heat dissipation. FASEB JOURNAL. 2010;24.
1021.	de Bustos F, Navarro JA, de Andres C, Molina JA, Jimenez-Jimenez FJ, Orti-Pareja M, et al. Cerebrospinal fluid nitrate levels in patients with multiple sclerosis. EUROPEAN NEUROLOGY. 1999;41(1):44-7.
1022.	De Bustos F, Navarro JA, De Andrés C, Molina JA, Jiménez-Jiménez FJ, Ortí-Pareja M, et al. Cerebrospinal fluid nitrate levels in patients with multiple sclerosis. European Neurology. 1999.
1023.	de Bustos F, Navarro JA, de Andrés C, Molina JA, Jiménez-Jiménez FJ, Ortí-Pareja M, et al. Cerebrospinal fluid nitrate levels in patients with multiple sclerosis. Eur Neurol. 1999;41(1):44-7.
1024.	de Bustos F, Navarro JA, de Andres C, Molina JA, Jimenez-Jimenez FJ, Orti-Pareja M, et al. Cerebrospinal fluid nitrate levels in patients with multiple sclerosis. EUROPEAN NEUROLOGY. 1999;41(1):44-7.
1025.	de Graaf WL. High Field MRI in Multiple Sclerosis: Novel multi-contrast protocols for detection of MS lesions and iron. 2012.
1026.	De Jager PL, Sawcer S, Waliszewska A, Farwell L, Wild G, Cohen A, et al. Evaluating the role of the 620W allele of protein tyrosine phosphatase PTPN22 in Crohn's disease and multiple sclerosis. Eur J Hum Genet. 2006;14(3):317-21.
1027.	De Keyser J, Wilczak N, Walter JH, Zurbriggen A. Disappearance of beta2-adrenergic receptors on astrocytes in canine distemper encephalitis: possible implications for the pathogenesis of multiple sclerosis. Neuroreport. 2001;12(2):191-4.
1028.	De la Torre GG, Mato I, Doval S, Espinosa R, Moya M, Cantero R, et al. Neurocognitive and emotional status after one-year of mindfulness-based intervention in patients with relapsing-remitting multiple sclerosis. Appl Neuropsychol Adult. 2022;29(2):183-92.
1029.	De Lury AD, Bisulca JA, Lee JS, Altaf MD, Coyle PK, Duong TQ. Magnetic resonance imaging detection of deep gray matter iron deposition in multiple sclerosis: A systematic review. J Neurol Sci. 2023;453:120816.
1030.	De Masi R, Orlando S, Bagordo F, Grassi T. IFP35 Is a Relevant Factor in Innate Immunity, Multiple Sclerosis, and Other Chronic Inflammatory Diseases: A Review. Biology (Basel). 2021;10(12).
1031.	De Mercanti S, Rolla S, Cucci A, Bardina V, Cocco E, Vladic A, et al. Alemtuzumab long-term immunologic effect Treg suppressor function increases up to 24 months. NEUROLOGY-NEUROIMMUNOLOGY & NEUROINFLAMMATION. 2016;3(1).
1032.	De Morsier G. Multiple sclerosis in two brothers both building painters; considerations on the possible role of lead in the etiology. Schweizerische medizinische Wochenschrift. 1952.
1033.	De Morsier G. [Multiple sclerosis in two brothers both building painters; considerations on the possible role of lead in the etiology]. Schweiz Med Wochenschr. 1952;82(16):443-5.
1034.	de Oliveira CA, Zissen M, Mongon J, McCammon JA. Molecular dynamics simulations of metalloproteinases types 2 and 3 reveal differences in the dynamic behavior of the S1' binding pocket. Curr Pharm Des. 2007;13(34):3471-5.
1035.	de Oliveira CAF, Zissen M, Mongon J, McCammon JA. Molecular dynamics simulations of metalloproteinases types 2 and 3 reveal differences in the dynamic behavior of the S1 ' binding pocket. CURRENT PHARMACEUTICAL DESIGN. 2007;13(34):3471-5.
1036.	de Oliveira CAF, Zissen M, Mongon J, McCammon JA. Molecular dynamics simulations of metalloproteinases types 2 and 3 reveal differences in the dynamic behavior of the S1′ binding pocket. Current Pharmaceutical Design. 2007.
1037.	de Oliveira M, Gianeti TMR, da Rocha FCG, Lisboa PN, Piacenti-Silva M. A preliminary study of the concentration of metallic elements in the blood of patients with multiple sclerosis as measured by ICP-MS. SCIENTIFIC REPORTS. 2020;10(1).
1038.	de Oliveira M, Gianeti TMR, da Rocha FCG, Lisboa PN, Piacenti-Silva M. A preliminary study of the concentration of metallic elements in the blood of patients with multiple sclerosis as measured by ICP-MS. SCIENTIFIC REPORTS. 2020;10(1).
1039.	de Oliveira M, Gianeti TMR, da Rocha FCG, Lisboa-Filho PN, Piacenti-Silva M. A preliminary study of the concentration of metallic elements in the blood of patients with multiple sclerosis as measured by ICP-MS. Scientific Reports. 2020.
1040.	de Oliveira M, Gianeti TMR, da Rocha FCG, Lisboa-Filho PN, Piacenti-Silva M. A preliminary study of the concentration of metallic elements in the blood of patients with multiple sclerosis as measured by ICP-MS. Sci Rep. 2020;10(1):13112.
1041.	de Oliveira M, Santinelli FB, Lisboa-Filho PN, Barbieri FA. The Blood Concentration of Metallic Nanoparticles Is Related to Cognitive Performance in People with Multiple Sclerosis: An Exploratory Analysis. Biomedicines. 2023;11(7).
1042.	De Riccardis L, Buccolieri A, Muci M, Pitotti E, De Robertis F, Trianni G, et al. Copper and ceruloplasmin dyshomeostasis in serum and cerebrospinal fluid of multiple sclerosis subjects. Biochim Biophys Acta Mol Basis Dis. 2018;1864(5 Pt A):1828-38.
1043.	de Toledo J, Fraga-Silva TFC, Borim PA, de Oliveira LRC, Oliveira EDS, Périco LL, et al. Organic Selenium Reaches the Central Nervous System and Downmodulates Local Inflammation: A Complementary Therapy for Multiple Sclerosis? Front Immunol. 2020;11:571844.
1044.	Deane K, Sarfraz A, Sarfraz Z, Valentine D, Idowu AR, Sanchez V. Unilateral Optic Neuritis Associated with SARS-CoV-2 Infection: A Rare Complication. Am J Case Rep. 2021;22:e931665.
1045.	DeBono N, Kelly-Reif K, Richardson D, Keil A, Robinson W, Troester M, et al. Mortality among autoworkers manufacturing electronics in Huntsville, Alabama. Am J Ind Med. 2019;62(4):282-95.
1046.	Decherney AH. The use of birth control pills in women with medical disorders. Clin Obstet Gynecol. 1981;24(3):965-75.
1047.	Decker BM, Thibault D, Davis KA, Willis AW. Population-Based Study of Nonelective Postpartum Readmissions in Women With Stroke, Migraine, Multiple Sclerosis, and Myasthenia Gravis. Neurology. 2022;98(15):e1545-e54.
1048.	Declemy A, Haddad R, Chesnel C, Charlanes A, Le Breton F, Sheikh Ismael S, et al. Prevalence of comorbidities in multiple sclerosis patients with neurogenic bladder. Prog Urol. 2021;31(12):732-8.
1049.	Dehbashi S, Hamouda D, Shanina E. Co-occurrence of multiple sclerosis and myasthenia gravis: A case report and review of immunological theories. Mult Scler Relat Disord. 2019;34:135-6.
1050.	Dehghanifiroozabadi M, Noferesti P, Amirabadizadeh A, Nakhaee S, Aaseth J, Noorbakhsh F, et al. Blood lead levels and multiple sclerosis: A case-control study. MULTIPLE SCLEROSIS AND RELATED DISORDERS. 2019;27:151-5.
1051.	Dehghanifiroozabadi M, Noferesti P, Amirabadizadeh A, Nakhaee S, Aaseth J, Noorbakhsh F, et al. Blood lead levels and multiple sclerosis: A case-control study. Multiple Sclerosis and Related Disorders. 2019.
1052.	Dehghanifiroozabadi M, Noferesti P, Amirabadizadeh A, Nakhaee S, Aaseth J, Noorbakhsh F, et al. Blood lead levels and multiple sclerosis: A case-control study. MULTIPLE SCLEROSIS AND RELATED DISORDERS. 2019;27:151-5.
1053.	Dehghanifiroozabadi M, Noferesti P, Amirabadizadeh A, Nakhaee S, Aaseth J, Noorbakhsh F, et al. Blood lead levels and multiple sclerosis: A case-control study. Mult Scler Relat Disord. 2019;27:151-5.
1054.	Dehghanifiroozabadi M, Noferesti P, Amirabadizadeh A, Nakhaee S, Aaseth J, Noorbakhsh F, et al. Blood lead levels and multiple sclerosis: A case-control study. MULTIPLE SCLEROSIS AND RELATED DISORDERS. 2019;27:151-5.
1055.	Dehghanifiroozabadi M, Noferesti P, Amirabadizadeh A, Nakhaee S, Aaseth J, Noorbakhsh F, et al. Blood lead levels and multiple sclerosis: A case-control study. Multiple sclerosis and related disorders. 2019;27:151-5.
1056.	Della Corte M, Santangelo G, Bisecco A, Sacco R, Siciliano M, d'Ambrosio A, et al. A simple measure of cognitive reserve is relevant for cognitive performance in MS patients. Neurol Sci. 2018;39(7):1267-73.
1057.	Deloire MS, Touil T, Brochet B, Dousset V, Caillé JM, Petry KG. Macrophage brain infiltration in experimental autoimmune encephalomyelitis is not completely compromised by suppressed T-cell invasion: in vivo magnetic resonance imaging illustration in effective anti-VLA-4 antibody treatment. Mult Scler. 2004;10(5):540-8.
1058.	Deloire MSA, Touil T, Brochet B, Dousset V, Caille JM, Petry KG. Macrophage brain infiltration in experimental autoimmune encephalomyelitis is not completely compromised by suppressed T-cell invasion: in vivo magnetic resonance imaging illustration in effective anti-VLA-4 antibody treatment. MULTIPLE SCLEROSIS. 2004;10(5):540-8.
1059.	Deloire MSA, Touil T, Brochet B, Dousset V, Caillé JM, Petry KG. Macrophage brain infiltration in experimental autoimmune encephalomyelitis is not completely compromised by suppressed T-cell invasion: In vivo magnetic resonance imaging illustration in effective anti-VLA-4 antibody treatment. Multiple Sclerosis. 2004.
1060.	Deloire MSA, Touil T, Brochet B, Dousset V, Caille JM, Petry KG. Macrophage brain infiltration in experimental autoimmune encephalomyelitis is not completely compromised by suppressed T-cell invasion: in vivo magnetic resonance imaging illustration in effective anti-VLA-4 antibody treatment. MULTIPLE SCLEROSIS. 2004;10(5):540-8.
1061.	Deluca HF, Cantorna MT. Vitamin D: its role and uses in immunology. Faseb j. 2001;15(14):2579-85.
1062.	Demoulins T, Gachelin G, Bequet D, Dormont D. A biased V alpha 24(+) T-cell repertoire leads to circulating NKT-cell defects in a multiple sclerosis patient at the onset of his disease. IMMUNOLOGY LETTERS. 2003;90(2-3):223-8.
1063.	Demoulins T, Gachelin G, Bequet D, Dormont D. A biased V alpha 24(+) T-cell repertoire leads to circulating NKT-cell defects in a multiple sclerosis patient at the onset of his disease. IMMUNOLOGY LETTERS. 2003;90(2-3):223-8.
1064.	Demoulins T, Gachelin G, Bequet D, Dormont D. A biased V alpha 24(+) T-cell repertoire leads to circulating NKT-cell defects in a multiple sclerosis patient at the onset of his disease. IMMUNOLOGY LETTERS. 2003;90(2-3):223-8.
1065.	Deniz U, Ozkirimli E, Ulgen KO. A systematic methodology for large scale compound screening: A case study on the discovery of novel S1PL inhibitors. JOURNAL OF MOLECULAR GRAPHICS & MODELLING. 2016;63:110-24.
1066.	Deniz U, Ozkirimli E, Ulgen KO. A systematic methodology for large scale compound screening: A case study on the discovery of novel S1PL inhibitors. Journal of Molecular Graphics and Modelling. 2016.
1067.	Deniz U, Ozkirimli E, Ulgen KO. A systematic methodology for large scale compound screening: A case study on the discovery of novel S1PL inhibitors. J Mol Graph Model. 2016;63:110-24.
1068.	Deniz U, Ozkirimli E, Ulgen KO. A systematic methodology for large scale compound screening: A case study on the discovery of novel S1PL inhibitors. JOURNAL OF MOLECULAR GRAPHICS & MODELLING. 2016;63:110-24.
1069.	Deraos G, Rodi M, Kalbacher H, Chatzantoni K, Karagiannis F, Synodinos L, et al. Properties of myelin altered peptide ligand cyclo (87-99)(Ala91, Ala96) MBP87-99 render it a promising drug lead for immunotherapy of multiple sclerosis. European journal of medicinal chemistry. 2015;101:13-23.
1070.	Deraos G, Rodi M, Kalbacher H, Chatzantoni K, Karagiannis F, Synodinos L, et al. Properties of myelin altered peptide ligand cyclo(87-99)(Ala91,Ala96) MBP87-99 render it a promising drug lead for immunotherapy of multiple sclerosis. EUROPEAN JOURNAL OF MEDICINAL CHEMISTRY. 2015;101:13-23.
1071.	Deraos G, Rodi M, Kalbacher H, Chatzantoni K, Karagiannis F, Synodinos L, et al. Properties of myelin altered peptide ligand cyclo(87-99)(Ala91,Ala96) MBP87-99 render it a promising drug lead for immunotherapy of multiple sclerosis. EUROPEAN JOURNAL OF MEDICINAL CHEMISTRY. 2015;101:13-23.
1072.	Deraos G, Rodi M, Kalbacher H, Chatzantoni K, Karagiannis F, Synodinos L, et al. Properties of myelin altered peptide ligand cyclo(87-99)(Ala91,Ala96) MBP87-99 render it a promising drug lead for immunotherapy of multiple sclerosis. EUROPEAN JOURNAL OF MEDICINAL CHEMISTRY. 2015;101:13-23.
1073.	Derdelinckx J, Cras P, Berneman ZN, Cools N. Antigen-Specific Treatment Modalities in MS: The Past, the Present, and the Future. Front Immunol. 2021;12:624685.
1074.	Derkus B, Acar Bozkurt P, Tulu M, Emregul KC, Yucesan C, Emregul E. Simultaneous quantification of Myelin Basic Protein and Tau proteins in cerebrospinal fluid and serum of Multiple Sclerosis patients using nanoimmunosensor. Biosensors and Bioelectronics. 2017.
1075.	Derkus B, Acar Bozkurt P, Tulu M, Emregul KC, Yucesan C, Emregul E. Simultaneous quantification of Myelin Basic Protein and Tau proteins in cerebrospinal fluid and serum of Multiple Sclerosis patients using nanoimmunosensor. Biosens Bioelectron. 2017;89(Pt 2):781-8.
1076.	deShazer DO. The mercury-multiple sclerosis connection. J Colo Dent Assoc. 1985;63(4):4.
1077.	Despotov K, Klivényi P, Nagy I, Pálvölgyi A, Vécsei L, Rajda C. Rare co-occurrence of multiple sclerosis and Wilson's disease - case report. BMC Neurol. 2022;22(1):178.
1078.	Desquenneclark L, Esch TR, Otvos L, Heberkatz E. T-CELL RECEPTOR PEPTIDE IMMUNIZATION LEADS TO ENHANCED AND CHRONIC EXPERIMENTAL ALLERGIC ENCEPHALOMYELITIS. PROCEEDINGS OF THE NATIONAL ACADEMY OF SCIENCES OF THE UNITED STATES OF AMERICA. 1991;88(16):7219-23.
1079.	Desquenneclark L, Esch TR, Otvos L, Heberkatz E. T-CELL RECEPTOR PEPTIDE IMMUNIZATION LEADS TO ENHANCED AND CHRONIC EXPERIMENTAL ALLERGIC ENCEPHALOMYELITIS. PROCEEDINGS OF THE NATIONAL ACADEMY OF SCIENCES OF THE UNITED STATES OF AMERICA. 1991;88(16):7219-23.
1080.	Dezern AE, Styler MJ, Drachman DB, Hummers LK, Jones RJ, Brodsky RA. Repeated treatment with high dose cyclophosphamide for severe autoimmune diseases. Am J Blood Res. 2013;3(1):84-90.
1081.	Dhanapalaratnam R, Markoulli M, Krishnan AV. Disorders of vision in multiple sclerosis. Clin Exp Optom. 2022;105(1):3-12.
1082.	Di Castro MA, Volterra A. Astrocyte control of the entorhinal cortex-dentate gyrus circuit: Relevance to cognitive processing and impairment in pathology. Glia. 2022;70(8):1536-53.
1083.	Di Cello F, Siddharthan V, Paul-Satyaseela M, Kim KS. Divergent effects of zinc depletion in brain vs non-brain endothelial cells. BIOCHEMICAL AND BIOPHYSICAL RESEARCH COMMUNICATIONS. 2005;335(2):373-6.
1084.	Di Cello F, Siddharthan V, Paul-Satyaseela M, Kim KS. Divergent effects of zinc depletion in brain vs non-brain endothelial cells. Biochem Biophys Res Commun. 2005;335(2):373-6.
1085.	Di Cello F, Siddharthan V, Paul-Satyaseela M, Kim KS. Divergent effects of zinc depletion in brain vs non-brain endothelial cells. BIOCHEMICAL AND BIOPHYSICAL RESEARCH COMMUNICATIONS. 2005;335(2):373-6.
1086.	Di Cello F, Siddharthan V, Paul-Satyaseela M, Kwang SK. Divergent effects of zinc depletion in brain vs non-brain endothelial cells. Biochemical and Biophysical Research Communications. 2005.
1087.	Di Ieva A, Lam T, Alcaide-Leon P, Bharatha A, Montanera W, Cusimano MD. Magnetic resonance susceptibility weighted imaging in neurosurgery: current applications and future perspectives. J Neurosurg. 2015;123(6):1463-75.
1088.	Di Lorenzo D, Biasiotto G, Zanella I. Source of iron overload in multiple sclerosis Diego Di Lorenzo, Giorgio Biasiotto, Isabella Zanella. Cellular and Molecular Life Sciences. 2014.
1089.	Di Lorenzo D, Biasiotto G, Zanella I. Source of iron overload in multiple sclerosis. CELLULAR AND MOLECULAR LIFE SCIENCES. 2014;71(16):3187-9.
1090.	Di Lorenzo D, Biasiotto G, Zanella I. Source of iron overload in multiple sclerosis. Cell Mol Life Sci. 2014;71(16):3187-9.
1091.	Di Lorenzo D, Biasiotto G, Zanella I. Source of iron overload in multiple sclerosis. Cellular and Molecular Life Sciences. 2014;71(16):3187-9.
1092.	Di Pietrantonj C, Rivetti A, Marchione P, Debalini MG, Demicheli V. Vaccines for measles, mumps, rubella, and varicella in children. Cochrane Database Syst Rev. 2021;11(11):Cd004407.
1093.	Diaconu CI, Conway D, Fox RJ, Rae-Grant A. Chronic cerebrospinal venous insufficiency as a cause of multiple sclerosis: Controversy and reality. Current Treatment Options in Cardiovascular Medicine. 2012.
1094.	Diaconu CI, Conway D, Fox RJ, Rae-Grant A. Chronic cerebrospinal venous insufficiency as a cause of multiple sclerosis: controversy and reality. Curr Treat Options Cardiovasc Med. 2012;14(2):203-14.
1095.	Dias de Sousa MA, Desidério CS, da Silva Catarino J, Trevisan RO, Alves da Silva DA, Rocha VFR, et al. Role of Cytokines, Chemokines and IFN-γ(+) IL-17(+) Double-Positive CD4(+) T Cells in Patients with Multiple Sclerosis. Biomedicines. 2022;10(9).
1096.	Dias-Carvalho A, Ferreira M, Reis-Mendes A, Ferreira R, Bastos ML, Fernandes E, et al. Chemobrain: mitoxantrone-induced oxidative stress, apoptotic and autophagic neuronal death in adult CD-1 mice. Arch Toxicol. 2022;96(6):1767-82.
1097.	Dias-Carvalho A, Margarida-Araújo A, Reis-Mendes A, Sequeira CO, Pereira SA, Guedes de Pinho P, et al. A Clinically Relevant Dosage of Mitoxantrone Disrupts the Glutathione and Lipid Metabolic Pathways of the CD-1 Mice Brain: A Metabolomics Study. Int J Mol Sci. 2023;24(17).
1098.	Dickerson KJ. Prognostic uncertainty in multiple sclerosis: A concept analysis. J Clin Nurs. 2023;32(3-4):633-42.
1099.	Didonna A, Canto Puig E, Ma Q, Matsunaga A, Ho B, Caillier SJ, et al. Ataxin-1 regulates B cell function and the severity of autoimmune experimental encephalomyelitis. Proc Natl Acad Sci U S A. 2020;117(38):23742-50.
1100.	Diem L, Ovchinnikov A, Friedli C, Hammer H, Kamber N, Chan A, et al. Efficacy and safety of ocrelizumab in patients with relapsing multiple sclerosis: Real-world experience of two Swiss multiple sclerosis centers. Mult Scler Relat Disord. 2024;86:105570.
1101.	Dillenseger A, Weidemann ML, Trentzsch K, Inojosa H, Haase R, Schriefer D, et al. Digital Biomarkers in Multiple Sclerosis. Brain Sci. 2021;11(11).
1102.	Dimov AV, Gillen KM, Nguyen TD, Kang J, Sharma R, Pitt D, et al. Magnetic Susceptibility Source Separation Solely from Gradient Echo Data: Histological Validation. Tomography. 2022;8(3):1544-51.
1103.	Dimov AV, Nguyen TD, Gillen KM, Marcille M, Spincemaille P, Pitt D, et al. Susceptibility source separation from gradient echo data using magnitude decay modeling. J Neuroimaging. 2022;32(5):852-9.
1104.	Ding D, Valdivia AO, Bhattacharya SK. Nuclear prelamin a recognition factor and iron dysregulation in multiple sclerosis. METABOLIC BRAIN DISEASE. 2020;35(2):275-82.
1105.	Ding D, Valdivia AO, Bhattacharya SK. Nuclear prelamin a recognition factor and iron dysregulation in multiple sclerosis. Metabolic Brain Disease. 2020.
1106.	Ding D, Valdivia AO, Bhattacharya SK. Nuclear prelamin a recognition factor and iron dysregulation in multiple sclerosis. METABOLIC BRAIN DISEASE. 2020;35(2):275-82.
1107.	Ding D, Valdivia AO, Bhattacharya SK. Nuclear prelamin a recognition factor and iron dysregulation in multiple sclerosis. Metab Brain Dis. 2020;35(2):275-82.
1108.	Ding D, Valdivia AO, Bhattacharya SK. Nuclear prelamin a recognition factor and iron dysregulation in multiple sclerosis. METABOLIC BRAIN DISEASE. 2020;35(2):275-82.
1109.	Ding D, Valdivia AO, Bhattacharya SK. Nuclear prelamin a recognition factor and iron dysregulation in multiple sclerosis. Metabolic brain disease. 2020;35(2):275-82.
1110.	Ding XL, Yan YP, Li X, Li K, Ciric B, Yang JX, et al. Silencing IFN-gamma Binding/Signaling in Astrocytes versus Microglia Leads to Opposite Effects on Central Nervous System Autoimmunity. JOURNAL OF IMMUNOLOGY. 2015;194(9):4251-64.
1111.	Ding XL, Yan YP, Li X, Li K, Ciric B, Yang JX, et al. Silencing IFN-gamma Binding/Signaling in Astrocytes versus Microglia Leads to Opposite Effects on Central Nervous System Autoimmunity. JOURNAL OF IMMUNOLOGY. 2015;194(9):4251-64.
1112.	Dinges J, Harris CM, Wallace GA, Argiriadi MA, Queeney KL, Perron DC, et al. Hit-to-lead evaluation of a novel class of sphingosine 1-phosphate lyase inhibitors. BIOORGANIC & MEDICINAL CHEMISTRY LETTERS. 2016;26(9):2297-302.
1113.	Dinges J, Harris CM, Wallace GA, Argiriadi MA, Queeney KL, Perron DC, et al. Hit-to-lead evaluation of a novel class of sphingosine 1-phosphate lyase inhibitors. BIOORGANIC & MEDICINAL CHEMISTRY LETTERS. 2016;26(9):2297-302.
1114.	Dionisio-Santos DA, Karaahmet B, Belcher EK, Owlett LD, Trojanczyk LA, Olschowka JA, et al. Evaluating Effects of Glatiramer Acetate Treatment on Amyloid Deposition and Tau Phosphorylation in the 3xTg Mouse Model of Alzheimer's Disease. Front Neurosci. 2021;15:758677.
1115.	Dmitriev AN, Osintseva ED, Gress VV, Kuvshinova IS. VEP Amplitude Variability for the 3x3, 5x5 and 8x8 Reversible Checkerboard LED Pattern Size in Multiple Sclerosis Diagnostic: Study on the Health Subjects.  XIV RUSSIAN-GERMANY CONFERENCE ON BIOMEDICAL ENGINEERING (RGC-2019)2019.
1116.	Do HT, Baars W, Borns K, Windhagen A, Schwinzer R. The 77C -> G mutation in the human CD45 (PTPRC) gene leads to increased intensity of TCR signaling in T cell lines from healthy individuals and patients with multiple sclerosis. JOURNAL OF IMMUNOLOGY. 2006;176(2):931-8.
1117.	Dobryakova E, Hulst HE, Spirou A, Chiaravalloti ND, Genova HM, Wylie GR, et al. Fronto-striatal network activation leads to less fatigue in multiple sclerosis. MULTIPLE SCLEROSIS JOURNAL. 2018;24(9):1174-82.
1118.	Dobryakova E, Hulst HE, Spirou A, Chiaravalloti ND, Genova HM, Wylie GR, et al. Fronto-striatal network activation leads to less fatigue in multiple sclerosis. MULTIPLE SCLEROSIS JOURNAL. 2018;24(9):1174-82.
1119.	Dobryakova E, Hulst HE, Spirou A, Chiaravalloti ND, Genova HM, Wylie GR, et al. Fronto-striatal network activation leads to less fatigue in multiple sclerosis. MULTIPLE SCLEROSIS JOURNAL. 2018;24(9):1174-82.
1120.	Doğan HO, Yildiz Ö K. Serum NADPH oxidase concentrations and the associations with iron metabolism in relapsing remitting multiple sclerosis. J Trace Elem Med Biol. 2019;55:39-43.
1121.	Dogan HO, Yildiz OK. Serum NADPH oxidase concentrations and the associations with iron metabolism in relapsing remitting multiple sclerosis. JOURNAL OF TRACE ELEMENTS IN MEDICINE AND BIOLOGY. 2019;55:39-43.
1122.	Doğan HO, Yildiz ÖK. Serum NADPH oxidase concentrations and the associations with iron metabolism in relapsing remitting multiple sclerosis. Journal of Trace Elements in Medicine and Biology. 2019.
1123.	Dogan HO, Yildiz OK. Serum NADPH oxidase concentrations and the associations with iron metabolism in relapsing remitting multiple sclerosis. JOURNAL OF TRACE ELEMENTS IN MEDICINE AND BIOLOGY. 2019;55:39-43.
1124.	Dogan HO, Yildiz OK. Serum NADPH oxidase concentrations and the associations with iron metabolism in relapsing remitting multiple sclerosis. JOURNAL OF TRACE ELEMENTS IN MEDICINE AND BIOLOGY. 2019;55:39-43.
1125.	Doğan HO, Yildiz ÖK. Serum NADPH oxidase concentrations and the associations with iron metabolism in relapsing remitting multiple sclerosis. Journal of Trace Elements in Medicine and Biology. 2019;55:39-43.
1126.	Doi K, Ishikura S, Shirasawa S. The Roles of ZFAT in Thymocyte Differentiation and Homeostasis of Peripheral Naive T-Cells. ANTICANCER RESEARCH. 2014;34(8):4489-95.
1127.	Doi K, Ishikura S, Shirasawa S. The roles of ZFAT in thymocyte differentiation and homeostasis of peripheral naive T-cells. Anticancer Research. 2014.
1128.	Doi K, Ishikura S, Shirasawa S. The roles of ZFAT in thymocyte differentiation and homeostasis of peripheral naive T-cells. Anticancer Res. 2014;34(8):4489-95.
1129.	Doi K, Ishikura S, Shirasawa S. The Roles of ZFAT in Thymocyte Differentiation and Homeostasis of Peripheral Naive T-Cells. ANTICANCER RESEARCH. 2014;34(8):4489-95.
1130.	Domonkos J, Huszak I, Konyves-Kolonics L, Tass J. [The problem of blood antihyaluronidase activation in multiple sclerosis]. Monatsschr Psychiatr Neurol. 1956;132(4):233-40.
1131.	Donatien P, Anand U, Yiangou Y, Sinisi M, Fox M, MacQuillan A, et al. Granulocyte-macrophage colony-stimulating factor receptor expression in clinical pain disorder tissues and role in neuronal sensitization. Pain Rep. 2018;3(5):e676.
1132.	Dong X, Xu G, Wang J, Yin N, Meng N. Clinical and MRI predictors of cognitive decline in patients with relapsing-remitting multiple sclerosis: A 2-year longitudinal study. Mult Scler Relat Disord. 2022;65:103838.
1133.	Dönmez B, Ozakbas S, Oktem MA, Gedizlioglu M, Coker I, Genc A, et al. HLA genotypes in Turkish patients with myasthenia gravis: comparison with multiple sclerosis patients on the basis of clinical subtypes and demographic features. Hum Immunol. 2004;65(7):752-7.
1134.	Donzé C, Massot C, Defer G, Vermersch P, Lecoz P, Derepeer O, et al. NUTRISEP: Assessment of the nutritional status of patients with multiple sclerosis and link to fatigue. Rev Neurol (Paris). 2023;179(4):282-8.
1135.	Dore-Duffy P, Catalanotto F, Donaldson JO, Ostrom KM, Testa MA. Zinc in multiple sclerosis. Ann Neurol. 1983;14(4):450-4.
1136.	Dore-Duffy P, Catalanotto F, Ostrom M, Donaldson JO. Zinc in multiple sclerosis (Abstr.). Ann Neurol. 1981;10:97.
1137.	Dore-Duffy P, Zurier RB. Lymphocyte adherence in multiple sclerosis. Role of monocytes and increased sensitivity of MS lymphocytes to prostaglandin E. Clinical Immunology and Immunopathology. 1981.
1138.	Dore‐Duffy P, Catalanotto F, Donaldson JO, Ostrom KM, Testa MA. Zinc in muliple sclerosis. Annals of Neurology. 1983.
1139.	Doreduffy P, Catalanotto F, Donaldson JO, Ostrom KM, Testa MA. ZINC IN MULTIPLE-SCLEROSIS. ANNALS OF NEUROLOGY. 1983;14(4):450-4.
1140.	Doreduffy P, Catalanotto F, Ostrom M, Donaldson JO. ZINC IN MULTIPLE-SCLEROSIS. TRANSACTIONS OF THE AMERICAN NEUROLOGICAL ASSOCIATION. 1981;106:282-3.
1141.	Doreduffy P, Catalanotto F, Ostrom M, Donaldson JO. ZINC IN MULTIPLE-SCLEROSIS. ANNALS OF NEUROLOGY. 1981;10(1):97-.
1142.	Dorsey-Campbell RJ, Quinn T, Felongco T, Delacruz D, Walters P, Scalfari A, et al. Pharmacy-led monitoring of disease modifying treatments in multiple sclerosis improves the quality of monitoring and improves patient satisfaction. MULTIPLE SCLEROSIS JOURNAL. 2016;22:792-.
1143.	Dotan N, Altstock RT, Schwarz M, Dukler A. Anti-glycan antibodies as biomarkers for diagnosis and prognosis. Lupus. 2006;15(7):442-50.
1144.	Dousset V, Brochet B, Caille JM, Petry KJ, editors. Enhancement of multiple sclerosis lesions with ultrasmall particle iron oxide: phase II study2001.
1145.	Dousset V, Brochet B, Deloire MS. a, Lagoarde L, Barroso B, Caille JM, Petry KG. MR imaging of relapsing multiple sclerosis patients using ultra-small-particle iron oxide and compared with gadolinium.[Internet]. AJNR American journal of neuroradiology. 2006;27(5):1000-5.
1146.	Dousset V, Brochet B, Deloire MS, Lagoarde L, Barroso B, Caille JM, et al. MR imaging of relapsing multiple sclerosis patients using ultra-small-particle iron oxide and compared with gadolinium. AJNR Am J Neuroradiol. 2006;27(5):1000-5.
1147.	Dousset V, Brochet B, Deloire MSA, Lagoarde L, Barroso B, Caille JM, et al. MR Imaging of relapsing multiple sclerosis patients using ultra-small-particle iron oxide and compared with gadolinium. AMERICAN JOURNAL OF NEURORADIOLOGY. 2006;27(5):1000-5.
1148.	Dousset V, Brochet B, Deloire MSA, Lagoarde L, Barroso B, Caille JM, et al. MR imaging of relapsing multiple sclerosis patients using ultra-small-particle iron oxide and compared with gadolinium. American Journal of Neuroradiology. 2006.
1149.	Dousset V, Brochet B, Deloire MSA, Lagoarde L, Barroso B, Caille JM, et al. MR Imaging of relapsing multiple sclerosis patients using ultra-small-particle iron oxide and compared with gadolinium. AMERICAN JOURNAL OF NEURORADIOLOGY. 2006;27(5):1000-5.
1150.	Dousset V, Brochet B, Deloire MSA, Lagoarde L, Barroso B, Caille JM, et al. MR Imaging of relapsing multiple sclerosis patients using ultra-small-particle iron oxide and compared with gadolinium. AMERICAN JOURNAL OF NEURORADIOLOGY. 2006;27(5):1000-5.
1151.	Dousset V, Brochet B, Deloire MSA, Lagoarde L, Barroso B, Caille JM, et al. MR imaging of relapsing multiple sclerosis patients using ultra-small-particle iron oxide and compared with gadolinium. American journal of neuroradiology. 2006;27(5):1000-5.
1152.	Dow CT. M. paratuberculosis and Parkinson's disease--is this a trigger. Med Hypotheses. 2014;83(6):709-12.
1153.	Dow CT, Kidess L. BCG Vaccine-The Road Not Taken. Microorganisms. 2022;10(10).
1154.	Downey DC. Fatigue syndromes: new thoughts and reinterpretation of previous data. Med Hypotheses. 1992;39(2):185-90.
1155.	Drayer B, Burger P, Hurwitz B. Reduced signal intensity on MR images of thalamus and putamen in multiple sclerosis: Increased iron content? American Journal of Neuroradiology. 1987.
1156.	Drayer B, Burger P, Hurwitz B, Dawson D, Cain J. Reduced signal intensity on MR images of thalamus and putamen in multiple sclerosis: Increased iron content? American Journal of Roentgenology. 1987.
1157.	Drayer B, Burger P, Hurwitz B, Dawson D, Cain J. REDUCED SIGNAL INTENSITY ON MR IMAGES OF THALAMUS AND PUTAMEN IN MULTIPLE-SCLEROSIS - INCREASED IRON CONTENT. AMERICAN JOURNAL OF NEURORADIOLOGY. 1987;8(3):413-9.
1158.	Drayer B, Burger P, Hurwitz B, Dawson D, Cain J. REDUCED SIGNAL INTENSITY ON MR IMAGES OF THALAMUS AND PUTAMEN IN MULTIPLE-SCLEROSIS - INCREASED IRON CONTENT. AMERICAN JOURNAL OF ROENTGENOLOGY. 1987;149(2):357-63.
1159.	Drayer B, Burger P, Hurwitz B, Dawson D, Cain J. Reduced signal intensity on MR images of thalamus and putamen in multiple sclerosis: increased iron content? AJR Am J Roentgenol. 1987;149(2):357-63.
1160.	Drayer B, Burger P, Hurwitz B, Dawson D, Cain J. Reduced signal intensity on MR images of thalamus and putamen in multiple sclerosis: increased iron content? American Journal of Neuroradiology. 1987;8(3):413-9.
1161.	Drayer BP, Burger P, Hurwitz B, Dawson D, Cain J, Leong J, et al. Magnetic resonance imaging in multiple sclerosis: Decreased signal in thalamus and putamen. Annals of Neurology. 1987.
1162.	Drayer BP, Burger P, Hurwitz B, Dawson D, Cain J, Leong J, et al. Magnetic resonance imaging in multiple sclerosis: decreased signal in thalamus and putamen. Ann Neurol. 1987;22(4):546-50.
1163.	Drerup M, Roth A, Kane A, Sullivan AB. Therapeutic Approaches to Insomnia and Fatigue in Patients with Multiple Sclerosis. Nat Sci Sleep. 2021;13:201-7.
1164.	Dressman D, Elyaman W. T Cells: A Growing Universe of Roles in Neurodegenerative Diseases. Neuroscientist. 2022;28(4):335-48.
1165.	Dropcho EJ, Richman DP, Antel JP, Arnason BG. Defective mitogenic responses in myasthenia gravis and multiple sclerosis. Ann Neurol. 1982;11(5):456-62.
1166.	Du S, Sah SK, Zeng C, Wang J, Liu Y, Xiong H, et al. Iron deposition in the gray matter in patients with relapse-remitting multiple sclerosis: A longitudinal study using three-dimensional (3D)-enhanced T2∗-weighted angiography (ESWAN). European Journal of Radiology. 2015.
1167.	Du S, Sah SK, Zeng C, Wang J, Liu Y, Xiong H, et al. Iron deposition in the gray matter in patients with relapse-remitting multiple sclerosis: A longitudinal study using three-dimensional (3D)-enhanced T2*-weighted angiography (ESWAN). Eur J Radiol. 2015;84(7):1325-32.
1168.	Du S, Sah SK, Zeng C, Wang J, Liu Y, Xiong H, et al. Iron deposition in the gray matter in patients with relapse-remitting multiple sclerosis: a longitudinal study using three-dimensional (3D)-enhanced T2*-weighted angiography (ESWAN). European Journal of Radiology. 2015;84(7):1325-32.
1169.	Du S, Zeng C, Zhang Z. Longitudinal Study of Iron Deposition and Volume in the Precentral Gyrus in Patients with Relapse-Remitting Multiple Sclerosis. Journal of Biosciences and Medicines. 2020;8(05):84.
1170.	Du SL, Li YM, Zeng C, Hou HX, Huang FH, Meng B, et al. MRI in longitudinal observation on iron deposition in precentral gray matter in patients with relapse-remitting multiple sclerosis. Chinese Journal of Medical Imaging Technology. 2013.
1171.	Du SL, Sah SK, Zeng C, Wang JJ, Liu Y, Xiong H, et al. Iron deposition in the gray matter in patients with relapse-remitting multiple sclerosis: A longitudinal study using three-dimensional (3D)-enhanced T2*-weighted angiography (ESWAN). EUROPEAN JOURNAL OF RADIOLOGY. 2015;84(7):1325-32.
1172.	Du SL, Sah SK, Zeng C, Wang JJ, Liu Y, Xiong H, et al. Iron deposition in the gray matter in patients with relapse-remitting multiple sclerosis: A longitudinal study using three-dimensional (3D)-enhanced T2*-weighted angiography (ESWAN). EUROPEAN JOURNAL OF RADIOLOGY. 2015;84(7):1325-32.
1173.	Du SL, Sah SK, Zeng C, Wang JJ, Liu Y, Xiong H, et al. Iron deposition in the gray matter in patients with relapse-remitting multiple sclerosis: A longitudinal study using three-dimensional (3D)-enhanced T2*-weighted angiography (ESWAN). EUROPEAN JOURNAL OF RADIOLOGY. 2015;84(7):1325-32.
1174.	Duan W, Sun Y, Wu M, Zhang Z, Zhang T, Wang H, et al. Carbon-silicon switch led to the discovery of novel synthetic cannabinoids with therapeutic effects in a mouse model of multiple sclerosis. Eur J Med Chem. 2021;226:113878.
1175.	Duan WW, Sun Y, Wu M, Zhang ZY, Zhang TT, Wang H, et al. Carbon-silicon switch led to the discovery of novel synthetic cannabinoids with therapeutic effects in a mouse model of multiple sclerosis. EUROPEAN JOURNAL OF MEDICINAL CHEMISTRY. 2021;226.
1176.	Duarte-Silva E, Meuth SG, Peixoto CA. The role of iron metabolism in the pathogenesis and treatment of multiple sclerosis. Front Immunol. 2023;14:1137635.
1177.	Dubbioso R, Ruggiero L, Esposito M, Tarantino P, De Angelis M, Aruta F, et al. Different cortical excitability profiles in hereditary brain iron and copper accumulation. NEUROLOGICAL SCIENCES. 2020;41(3):679-85.
1178.	Dubbioso R, Ruggiero L, Esposito M, Tarantino P, De Angelis M, Aruta F, et al. Different cortical excitability profiles in hereditary brain iron and copper accumulation. Neurological Sciences. 2020.
1179.	Dubbioso R, Ruggiero L, Esposito M, Tarantino P, De Angelis M, Aruta F, et al. Different cortical excitability profiles in hereditary brain iron and copper accumulation. Neurol Sci. 2020;41(3):679-85.
1180.	Dubbioso R, Ruggiero L, Esposito M, Tarantino P, De Angelis M, Aruta F, et al. Different cortical excitability profiles in hereditary brain iron and copper accumulation. NEUROLOGICAL SCIENCES. 2020;41(3):679-85.
1181.	Dubik M, Marczynska J, Mørch MT, Webster G, Jensen KN, Wlodarczyk A, et al. Innate Signaling in the CNS Prevents Demyelination in a Focal EAE Model. Front Neurosci. 2021;15:682451.
1182.	Dudek I, Zagórski T, Liskiewicz J, Kedziora J, Chmielewski H. [Effect of gamma radiation on selected indicators of oxygen metabolism in erythrocytes of patients with multiple sclerosis]. Neurol Neurochir Pol. 1992;26(1):34-9.
1183.	Dufek M, Vanicek J. Early Initiation of a High-Dose Interferon Beta-1a Treatment (Rebif (R) 44) in an Active Form of Multiple Sclerosis Leading to Long-Term Disease Stabilization - a Case Report. CESKA A SLOVENSKA NEUROLOGIE A NEUROCHIRURGIE. 2013;76:9-11.
1184.	Dufek M, Vanicek J. Early Initiation of a High-Dose Interferon Beta-1a Treatment (Rebif (R) 44) in an Active Form of Multiple Sclerosis Leading to Long-Term Disease Stabilization - a Case Report. CESKA A SLOVENSKA NEUROLOGIE A NEUROCHIRURGIE. 2013;76:9-11.
1185.	Dufek M, Vanicek J. Early Initiation of a High-Dose Interferon Beta-1a Treatment (Rebif (R) 44) in an Active Form of Multiple Sclerosis Leading to Long-Term Disease Stabilization - a Case Report. CESKA A SLOVENSKA NEUROLOGIE A NEUROCHIRURGIE. 2013;76:9-11.
1186.	Dufour A, Salmaggi A, Eoli M, La Mantia L, Milanese C, Nespolo A. Phenotype analysis of unstimulated lymphocytes and anti-CD3-stimulated proliferating T-cells from cerebrospinal fluid and peripheral blood in patients with multiple sclerosis and other neurological diseases. Int J Neurosci. 1993;73(3-4):277-85.
1187.	Dufour A, Salmaggi A, Eoli M, Lamantia L, Milanese C, Nespolo A. PHENOTYPE ANALYSIS OF UNSTIMULATED LYMPHOCYTES AND ANTI-CD3-STIMULATED PROLIFERATING T-CELLS FROM CEREBROSPINAL-FLUID AND PERIPHERAL-BLOOD IN PATIENTS WITH MULTIPLE-SCLEROSIS AND OTHER NEUROLOGICAL DISEASES. INTERNATIONAL JOURNAL OF NEUROSCIENCE. 1993;73(3-4):277-85.
1188.	Dufour A, Salmaggi A, Eoli M, Lamantia L, Milanese C, Nespolo A. PHENOTYPE ANALYSIS OF UNSTIMULATED LYMPHOCYTES AND ANTI-CD3-STIMULATED PROLIFERATING T-CELLS FROM CEREBROSPINAL-FLUID AND PERIPHERAL-BLOOD IN PATIENTS WITH MULTIPLE-SCLEROSIS AND OTHER NEUROLOGICAL DISEASES. INTERNATIONAL JOURNAL OF NEUROSCIENCE. 1993;73(3-4):277-85.
1189.	Dufour A, Salmaggi A, La Mantia L, Eoli M, Nespolo A, Milanese C. High-dose methylprednisolone treatment-induced changes in immunological parameters in progressive MS patients. Int J Neurosci. 1994;75(1-2):119-28.
1190.	Duncan ID, Brower A, Kondo Y, Curlee JF, Schultz RD. Extensive remyelination of the CNS leads to functional recovery. PROCEEDINGS OF THE NATIONAL ACADEMY OF SCIENCES OF THE UNITED STATES OF AMERICA. 2009;106(16):6832-6.
1191.	Duncan ID, Brower A, Kondo Y, Curlee JF, Schultz RD. Extensive remyelination of the CNS leads to functional recovery. PROCEEDINGS OF THE NATIONAL ACADEMY OF SCIENCES OF THE UNITED STATES OF AMERICA. 2009;106(16):6832-6.
1192.	Dunham J, Bauer J, Campbell GR, Mahad DJ, van Driel N, van der Pol SMA, et al. Oxidative Injury and Iron Redistribution Are Pathological Hallmarks of Marmoset Experimental Autoimmune Encephalomyelitis. JOURNAL OF NEUROPATHOLOGY AND EXPERIMENTAL NEUROLOGY. 2017;76(6):467-78.
1193.	Dunham J, Bauer J, Campbell GR, Mahad DJ, van Driel N, van der Pol SMA, et al. Oxidative injury and iron redistribution are pathological hallmarks of marmoset experimental autoimmune encephalomyelitis. Journal of Neuropathology and Experimental Neurology. 2017.
1194.	Dunham J, Bauer J, Campbell GR, Mahad DJ, van Driel N, van der Pol SMA, et al. Oxidative Injury and Iron Redistribution Are Pathological Hallmarks of Marmoset Experimental Autoimmune Encephalomyelitis. J Neuropathol Exp Neurol. 2017;76(6):467-78.
1195.	Dunham J, Bauer J, Campbell GR, Mahad DJ, van Driel N, van der Pol SMA, et al. Oxidative Injury and Iron Redistribution Are Pathological Hallmarks of Marmoset Experimental Autoimmune Encephalomyelitis. JOURNAL OF NEUROPATHOLOGY AND EXPERIMENTAL NEUROLOGY. 2017;76(6):467-78.
1196.	Dunham J, Van De Vis R, Bauer J, Wubben J, Van Driel N, Laman JD, et al. Severe oxidative stress in an acute inflammatory demyelinating model in the rhesus monkey. PLoS ONE. 2017.
1197.	Dunham J, van de Vis R, Bauer J, Wubben J, van Driel N, Laman JD, et al. Severe oxidative stress in an acute inflammatory demyelinating model in the rhesus monkey. PLOS ONE. 2017;12(11).
1198.	Dunham J, van de Vis R, Bauer J, Wubben J, van Driel N, Laman JD, et al. Severe oxidative stress in an acute inflammatory demyelinating model in the rhesus monkey. PLoS One. 2017;12(11):e0188013.
1199.	Dunham J, van de Vis R, Bauer J, Wubben J, van Driel N, Laman JD, et al. Severe oxidative stress in an acute inflammatory demyelinating model in the rhesus monkey. PLOS ONE. 2017;12(11).
1200.	Durelli L, Conti L, Clerico M, Boselli D, Contessa G, Ripellino P, et al. T-Helper 17 Cells Expand in Multiple Sclerosis and Are Inhibited by Interferon-beta. ANNALS OF NEUROLOGY. 2009;65(5):499-509.
1201.	Durelli L, Conti L, Clerico M, Boselli D, Contessa G, Ripellino P, et al. T-helper 17 cells expand in multiple sclerosis and are inhibited by interferon-beta. Ann Neurol. 2009;65(5):499-509.
1202.	Durelli L, Conti L, Clerico M, Boselli D, Contessa G, Ripellino P, et al. T-Helper 17 Cells Expand in Multiple Sclerosis and Are Inhibited by Interferon-beta. ANNALS OF NEUROLOGY. 2009;65(5):499-509.
1203.	Durgun E, Ulusoy H, Narin İ. Sensitive, reliable and simultaneous determination of Fingolimod and Citalopram drug molecules used in multiple sclerosis treatment based on magnetic solid phase extraction and HPLC-PDA. J Chromatogr B Analyt Technol Biomed Life Sci. 2024;1237:124071.
1204.	Durlach J, Bac P, Durlach V, Durlach A, Bara M, Guiet-Bara A. Are age-related neurodegenerative diseases linked with various types of magnesium depletion? MAGNESIUM RESEARCH. 1997;10(4):339-53.
1205.	Durlach J, Bac P, Durlach V, Durlach A, Bara M, Guiet-Bara A. Are age-related neurodegenerative diseases linked with various types of magnesium depletion? MAGNESIUM RESEARCH. 1997;10(4):339-53.
1206.	Durlach J, Pagès N, Bac P, Bara M, Guiet-Bara A. Importance of magnesium depletion with hypofunction of the biological clock in the pathophysiology of headhaches with photophobia, sudden infant death and some clinical forms of multiple sclerosis. Magnesium Research. 2004.
1207.	Durlach J, Pages N, Bac P, Bara M, Guiet-Bara A. Importance of magnesium depletion with hypofunction of the biological clock in the pathophysiology of headhaches with photophobia, sudden infant death and some clinical forms of multiple sclerosis. MAGNESIUM RESEARCH. 2004;17(4):314-26.
1208.	Durlach J, Pagès N, Bac P, Bara M, Guiet-Bara A. Importance of magnesium depletion with hypofunction of the biological clock in the pathophysiology of headhaches with photophobia, sudden infant death and some clinical forms of multiple sclerosis. Magnes Res. 2004;17(4):314-26.
1209.	Durlach J, Pagès N, Bac P, Bara M, Guiet-Bara A. Importance of magnesium depletion with hypofunction of the biological clock in the pathophysiology of headhaches with photophobia, sudden infant death and some clinical forms of multiple sclerosis. Magnesium research. 2004;17(4):314-26.
1210.	Durmanova V, Parnicka Z, Javor J, Cserkoova A, Copikova-Cudrakova D, Lisa I, et al. Association of MMP9 polymorphism rs3918242 with clinical findings in Slovak multiple sclerosis patients. ACTIVITAS NERVOSA SUPERIOR REDIVIVA. 2019;61(1):12-8.
1211.	Durmanova V, Parnicka Z, Javor J, Cserkoova A, Copikova-Cudrakova D, Lisa I, et al. Association of MMP9 polymorphism rs3918242 with clinical findings in Slovak multiple sclerosis patients. Activitas Nervosa Superior Rediviva. 2019.
1212.	Durmanova V, Parnicka Z, Javor J, Cserkoova A, Copikova-Cudrakova D, Lisa I, et al. Association of MMP9 polymorphism rs3918242 with clinical findings in Slovak multiple sclerosis patients. ACTIVITAS NERVOSA SUPERIOR REDIVIVA. 2019;61(1):12-8.
1213.	Durrenberger PF, Fernando FS, Kashefi SN, Bonnert TP, Seilhean D, Nait-Oumesmar B, et al. Common mechanisms in neurodegeneration and neuroinflammation: a BrainNet Europe gene expression microarray study. JOURNAL OF NEURAL TRANSMISSION. 2015;122(7):1055-68.
1214.	Durrenberger PF, Fernando FS, Kashefi SN, Bonnert TP, Seilhean D, Nait-Oumesmar B, et al. Common mechanisms in neurodegeneration and neuroinflammation: a BrainNet Europe gene expression microarray study. Journal of Neural Transmission. 2015.
1215.	Durrenberger PF, Fernando FS, Kashefi SN, Bonnert TP, Seilhean D, Nait-Oumesmar B, et al. Common mechanisms in neurodegeneration and neuroinflammation: a BrainNet Europe gene expression microarray study. J Neural Transm (Vienna). 2015;122(7):1055-68.
1216.	Durrenberger PF, Fernando FS, Kashefi SN, Bonnert TP, Seilhean D, Nait-Oumesmar B, et al. Common mechanisms in neurodegeneration and neuroinflammation: a BrainNet Europe gene expression microarray study. JOURNAL OF NEURAL TRANSMISSION. 2015;122(7):1055-68.
1217.	Dusek P, Hofer T, Alexander J, Roos PM, Aaseth JO. Cerebral Iron Deposition in Neurodegeneration. Biomolecules. 2022;12(5).
1218.	Dusek P, Schneider SA, Aaseth J. Iron chelation in the treatment of neurodegenerative diseases. JOURNAL OF TRACE ELEMENTS IN MEDICINE AND BIOLOGY. 2016;38:81-92.
1219.	Dusek P, Schneider SA, Aaseth J. Iron chelation in the treatment of neurodegenerative diseases. Journal of Trace Elements in Medicine and Biology. 2016.
1220.	Dusek P, Schneider SA, Aaseth J. Iron chelation in the treatment of neurodegenerative diseases. J Trace Elem Med Biol. 2016;38:81-92.
1221.	Dusek P, Schneider SA, Aaseth J. Iron chelation in the treatment of neurodegenerative diseases. JOURNAL OF TRACE ELEMENTS IN MEDICINE AND BIOLOGY. 2016;38:81-92.
1222.	Dworsky-Fried Z, Faig CA, Vogel HA, Kerr BJ, Taylor AMW. Central amygdala inflammation drives pain hypersensitivity and attenuates morphine analgesia in experimental autoimmune encephalomyelitis. Pain. 2022;163(1):e49-e61.
1223.	Dwyer M, Heininen-Brown M, Hagemeier J, Poloni G, Bergsland N, Magnano C, et al. Iron Deposition in Multiple Sclerosis Lesions Measured by Susceptibility-Weighted Imaging Filtered Phase. A Case Control Study (P03. 042). AAN Enterprises; 2012.
1224.	Dwyer M, Heininen-Brown M, Hagemeier J, Poloni G, Bergsland N, Magnano C, et al. Iron Deposition in Multiple Sclerosis Lesions Measured by Susceptibility-Weighted Imaging Filtered Phase. A Case Control Study. NEUROLOGY. 2012;78.
1225.	Dwyer MG, Dolezal O, Hussein S, Horakova D, Havrdova E, Cox JL, et al., editors. Development of central atrophy may lead to underestimation of lesion accrual in patients with multiple sclerosis2007: DR DIETRICH STEINKOPFF VERLAG PO BOX 10 04 62, D-64204 DARMSTADT, GERMANY.
1226.	Dwyer MG, Dolezal O, Hussein S, Horakova D, Havrdova E, Cox JL, et al. Development of central atrophy may lead to underestimation of lesion accrual in patients with multiple sclerosis. JOURNAL OF NEUROLOGY. 2007;254:189-.
1227.	Dwyer MG, Zamboni P, Haacke M, Menegatti E, Weinstock-Guttman B, Schirda C, et al., editors. Chronic Cerebrospinal Venous Insufficiency and Iron Deposition on Susceptibility-Weighted Imaging in Patients with Multiple Sclerosis2010: LIPPINCOTT WILLIAMS & WILKINS 530 WALNUT ST, PHILADELPHIA, PA 19106-3621 USA.
1228.	Dwyer MG, Zamboni P, Haacke M, Menegatti E, Weinstock-Guttman B, Schirda C, et al. Chronic Cerebrospinal Venous Insufficiency and Iron Deposition on Susceptibility-Weighted Imaging in Patients with Multiple Sclerosis. NEUROLOGY. 2010;74(9):A239-A.
1229.	Dwyer MG, Zivadinov R, Markovic-Plese S, Bergsland N, Heininen-Brown M, Carl E, et al. Associations between changes in ferritin levels and susceptibility-weighted imaging filtered phase in patients with relapsing-remitting multiple sclerosis over 24 weeks of therapy with subcutaneous interferon beta-1a three times weekly. J Neuroimmunol. 2015;281:44-50.
1230.	Dymecka J, Gerymski R, Tataruch R, Bidzan M. Sense of Coherence and Health-Related Quality of Life in Patients with Multiple Sclerosis: The Role of Physical and Neurological Disability. J Clin Med. 2022;11(6).
1231.	Dymecki J, Bertrand E, Tomankiewicz Z, Szuniewicz H. Hallervorden-Spatz disease in an adult patient. FOLIA NEUROPATHOLOGICA. 1999;37(4):235-8.
1232.	Dymecki J, Bertrand E, Tomankiewicz Z, Szuniewicz H. Hallervorden-Spatz disease in an adult patient. Folia Neuropathologica. 1999.
1233.	Dymecki J, Bertrand E, Tomankiewicz Z, Szuniewicz H. Hallervorden-Spatz disease in an adult patient. Folia Neuropathol. 1999;37(4):235-8.
1234.	Dymecki J, Bertrand E, Tomankiewicz Z, Szuniewicz H. Hallervorden-Spatz disease in an adult patient. FOLIA NEUROPATHOLOGICA. 1999;37(4):235-8.
1235.	Dzieżyc K, Litwin T, Członkowska A. Multiple sclerosis in two patients with coexisting Wilson's disease. Mult Scler Relat Disord. 2014;3(3):387-90.
1236.	Ebadi M, Iversen PL, Hao R, Cerutis DR, Rojas P, Happe HK, et al. EXPRESSION AND REGULATION OF BRAIN METALLOTHIONEIN. NEUROCHEMISTRY INTERNATIONAL. 1995;27(1):1-22.
1237.	Ebadi M, Iversen PL, Hao R, Cerutis DR, Rojas P, Happe HK, et al. Expression and regulation of brain metallothionein. Neurochemistry International. 1995.
1238.	Ebadi M, Iversen PL, Hao R, Cerutis DR, Rojas P, Happe HK, et al. Expression and regulation of brain metallothionein. Neurochem Int. 1995;27(1):1-22.
1239.	Ebrahimiyan H, Aslani S, Rezaei N, Jamshidi A, Mahmoudi M. Survivin and autoimmunity; the ins and outs. Immunol Lett. 2018;193:14-24.
1240.	Eckermann M, Meer FVD, Cloetens P, Ruhwedel T, Mobius W, Stadelmann C, et al. Three-dimensional virtual histology of the cerebral cortex based on phase-contrast X-ray tomography. Biomedical Optics Express. 2021.
1241.	Eckermann M, Van der Meer F, Cloetens P, Ruhwedel T, Mobius W, Stadelmann C, et al. Three-dimensional virtual histology of the cerebral cortex based on phase-contrast X-ray tomography. BIOMEDICAL OPTICS EXPRESS. 2021;12(12):7582-98.
1242.	Eckermann M, van der Meer F, Cloetens P, Ruhwedel T, Möbius W, Stadelmann C, et al. Three-dimensional virtual histology of the cerebral cortex based on phase-contrast X-ray tomography. Biomed Opt Express. 2021;12(12):7582-98.
1243.	Eckermann M, Van der Meer F, Cloetens P, Ruhwedel T, Mobius W, Stadelmann C, et al. Three-dimensional virtual histology of the cerebral cortex based on phase-contrast X-ray tomography. BIOMEDICAL OPTICS EXPRESS. 2021;12(12):7582-98.
1244.	Eggers EL, Michel BA, Wu H, Wang SZ, Bevan CJ, Abounasr A, et al. Clonal relationships of CSF B cells in treatment-naive multiple sclerosis patients. JCI Insight. 2017;2(22).
1245.	Eggers EL, Michel BA, Wu H, Wang SZ, Bevan CJ, Abounasr A, et al. Clonal relationships of CSF B cells in treatment-naive multiple sclerosis patients. JCI INSIGHT. 2017;2(22).
1246.	Eggers EL, Michel BA, Wu H, Wang SZ, Bevan CJ, Abounasr A, et al. Clonal relationships of CSF B cells in treatment-naive multiple sclerosis patients. JCI INSIGHT. 2017;2(22).
1247.	Eghbali BB, Saadat S, Hasanzadeh K, Pourramzani A, Khatami SS, Saberi A, et al. Relationship between self-compassion and psychological well-being with the mediating role of resilience in people with multiple sclerosis. Postep Psychiatr Neurol. 2022;31(2):43-51.
1248.	Eisele P, Wittayer M, Weber CE, Platten M, Schirmer L, Gass A. Impact of disease-modifying therapies on evolving tissue damage in iron rim multiple sclerosis lesions. Mult Scler. 2022;28(14):2294-8.
1249.	Ekundayo TC, Olasehinde TA, Falade AO, Adewoyin MA, Iwu CD, Igere BE, et al. Systematic review and meta-analysis of Mycobacterium avium subsp. paratuberculosis as environmental trigger of multiple sclerosis. Mult Scler Relat Disord. 2022;59:103671.
1250.	El-Ghanem M, Abdulrazeq H, Brasiliense L, Abbad H, Aguilar-Salinas P, Al-Mufti F, et al. Outcomes of Mechanical Thrombectomy in Patients With Neurological Disorders: A National Inpatient Sample Database Analysis. Cureus. 2024;16(2):e54063.
1251.	El'chaninov AP, Artiushkin AV, Skoromets AA. [Diagnostic value of radionuclide visualization of the reticuloendothelial system of macrophages in multiple sclerosis]. Zh Nevropatol Psikhiatr Im S S Korsakova. 1986;86(2):200-3.
1252.	Elbagory AM, Hussein AA, Meyer M. The In Vitro Immunomodulatory Effects Of Gold Nanoparticles Synthesized From Hypoxis hemerocallidea Aqueous Extract And Hypoxoside On Macrophage And Natural Killer Cells. Int J Nanomedicine. 2019;14:9007-18.
1253.	Elberry RA, Alazab R, Abdel Hady A, Abdellatif S, Elsayed A. Risk Analysis of Specific Environmental Heavy Metals as a Trigger of CD4 & CD8 Among Cases of Multiple Sclerosis: Case-control study. Al-Azhar International Medical Journal. 2020;1(5):164-70.
1254.	Elbert G. Trace elements in the management of multiple sclerosis. Hippokrates. 1956.
1255.	Elbert G. [Trace elements in the management of multiple sclerosis]. Hippokrates. 1956;27(15):488-91.
1256.	Elbert G. Trace elements in the management of multiple sclerosis. Hippokrates. 1956;27(15):488-91.
1257.	Eley BM, Cox SW. The release, absorption and possible health effects of mercury from dental amalgam: a review of recent findings. Br Dent J. 1993;175(10):355-62.
1258.	ElFar OA, Billa N, Lim HR, Chew KW, Cheah WY, Munawaroh HSH, et al. Advances in delivery methods of Arthrospira platensis (spirulina) for enhanced therapeutic outcomes. Bioengineered. 2022;13(6):14681-718.
1259.	Elgallab J, Charvet L, Krupp L. A Case of Cognitive and Behavioral Decline leading to Onset of Pediatric Onset Multiple Sclerosis. NEUROLOGY. 2018;90.
1260.	Elham K, Mehran H, Masood E. THE RELATIONSHIP OF CONCENTRATIONS OF LEAD AND ZINC AND MULTIPLE SCLEROSIS IN ISFAHAN PROVINCE, IRAN. JOURNAL OF ISFAHAN MEDICAL SCHOOL (IUMS). 2014;32(275):160-9.
1261.	Elitt CM, Fahrni CJ, Rosenberg PA. Zinc homeostasis and zinc signaling in white matter development and injury. NEUROSCIENCE LETTERS. 2019;707.
1262.	Elitt CM, Fahrni CJ, Rosenberg PA. Zinc homeostasis and zinc signaling in white matter development and injury. Neuroscience Letters. 2019.
1263.	Elitt CM, Fahrni CJ, Rosenberg PA. Zinc homeostasis and zinc signaling in white matter development and injury. Neurosci Lett. 2019;707:134247.
1264.	Elitt CM, Fahrni CJ, Rosenberg PA. Zinc homeostasis and zinc signaling in white matter development and injury. NEUROSCIENCE LETTERS. 2019;707.
1265.	Elkady A. Development and Application of Quantitative Gradient-Echo MRI Techniques for Assessment of Iron and Myelin in the Multiple Sclerosis Brain. 2018.
1266.	Elkady AM, Cobzas D, Sun H, Blevins G, Wilman AH. Progressive iron accumulation across multiple sclerosis phenotypes revealed by sparse classification of deep gray matter. Journal of Magnetic Resonance Imaging. 2017.
1267.	Elkady AM, Cobzas D, Sun H, Blevins G, Wilman AH. Progressive iron accumulation across multiple sclerosis phenotypes revealed by sparse classification of deep gray matter. J Magn Reson Imaging. 2017;46(5):1464-73.
1268.	Elkady AM, Cobzas D, Sun H, Blevins G, Wilman AH. Progressive iron accumulation across multiple sclerosis phenotypes revealed by sparse classification of deep gray matter. Journal of Magnetic Resonance Imaging. 2017;46(5):1464-73.
1269.	Elkady AM, Cobzas D, Sun H, Blevins G, Wilman AH. Discriminative analysis of regional evolution of iron and myelin/calcium in deep gray matter of multiple sclerosis and healthy subjects. Journal of Magnetic Resonance Imaging. 2018.
1270.	Elkady AM, Cobzas D, Sun H, Blevins G, Wilman AH. Discriminative analysis of regional evolution of iron and myelin/calcium in deep gray matter of multiple sclerosis and healthy subjects. J Magn Reson Imaging. 2018.
1271.	Elkady AM, Cobzas D, Sun H, Blevins G, Wilman AH. Discriminative analysis of regional evolution of iron and myelin/calcium in deep gray matter of multiple sclerosis and healthy subjects. Journal of Magnetic Resonance Imaging. 2018;48(3):652-68.
1272.	Elkady AM, Cobzas D, Sun H, Seres P, Blevins G, Wilman AH. Five year iron changes in relapsing-remitting multiple sclerosis deep gray matter compared to healthy controls. Multiple Sclerosis and Related Disorders. 2019.
1273.	Elkady AM, Cobzas D, Sun H, Seres P, Blevins G, Wilman AH. Five year iron changes in relapsing-remitting multiple sclerosis deep gray matter compared to healthy controls. Mult Scler Relat Disord. 2019;33:107-15.
1274.	Elkady AM, Cobzas D, Sun H, Seres P, Blevins G, Wilman AH. Five year iron changes in relapsing-remitting multiple sclerosis deep gray matter compared to healthy controls. Multiple sclerosis and related disorders. 2019;33:107-15.
1275.	Elkady AM, Cobzas D, Sun HF, Blevins G, Wilman AH. Progressive Iron Accumulation Across Multiple Sclerosis Phenotypes Revealed by Sparse Classification of Deep Gray Matter. JOURNAL OF MAGNETIC RESONANCE IMAGING. 2017;46(5):1464-73.
1276.	Elkady AM, Cobzas D, Sun HF, Blevins G, Wilman AH. Progressive Iron Accumulation Across Multiple Sclerosis Phenotypes Revealed by Sparse Classification of Deep Gray Matter. JOURNAL OF MAGNETIC RESONANCE IMAGING. 2017;46(5):1464-73.
1277.	Elkady AM, Cobzas D, Sun HF, Blevins G, Wilman AH. Progressive Iron Accumulation Across Multiple Sclerosis Phenotypes Revealed by Sparse Classification of Deep Gray Matter. JOURNAL OF MAGNETIC RESONANCE IMAGING. 2017;46(5):1464-73.
1278.	Elkady AM, Cobzas D, Sun HF, Blevins G, Wilman AH. Discriminative Analysis of Regional Evolution of Iron and Myelin/Calcium in Deep Gray Matter of Multiple Sclerosis and Healthy Subjects. JOURNAL OF MAGNETIC RESONANCE IMAGING. 2018;48(3):652-68.
1279.	Elkady AM, Cobzas D, Sun HF, Blevins G, Wilman AH. Discriminative Analysis of Regional Evolution of Iron and Myelin/Calcium in Deep Gray Matter of Multiple Sclerosis and Healthy Subjects. JOURNAL OF MAGNETIC RESONANCE IMAGING. 2018;48(3):652-68.
1280.	Elkady AM, Cobzas D, Sun HF, Blevins G, Wilman AH. Discriminative Analysis of Regional Evolution of Iron and Myelin/Calcium in Deep Gray Matter of Multiple Sclerosis and Healthy Subjects. JOURNAL OF MAGNETIC RESONANCE IMAGING. 2018;48(3):652-68.
1281.	Elkady AM, Cobzas D, Sun HF, Seres P, Blevins G, Wilman AH. Five year iron changes in relapsing-remitting multiple sclerosis deep gray matter compared to healthy controls. MULTIPLE SCLEROSIS AND RELATED DISORDERS. 2019;33:107-15.
1282.	Elkama A, Orhan G, Alp O, Tosun G, Öztekin N, Karahalil B. DNA repair and detoxifying gene polymorphisms in patients with multiple sclerosis and dental amalgam fillings. Gazi Medical Journal. 2020.
1283.	Elkama A, Orhan G, Alp O, Tosun G, Oztekin N, Karahalil B. DNA Repair and Detoxifying Gene Polymorphisms in Patients with Multiple Sclerosis and Dental Amalgam Fillings. GAZI MEDICAL JOURNAL. 2021;32(1):33-9.
1284.	Elkama A, Orhan G, Alp O, Tosun G, Oztekin N, Karahalil B. DNA Repair and Detoxifying Gene Polymorphisms in Patients with Multiple Sclerosis and Dental Amalgam Fillings. GAZI MEDICAL JOURNAL. 2021;32(1):33-9.
1285.	Elkjaer ML, Hartebrodt A, Oubounyt M, Weber A, Vitved L, Reynolds R, et al. Single-Cell Multi-Omics Map of Cell Type-Specific Mechanistic Drivers of Multiple Sclerosis Lesions. Neurol Neuroimmunol Neuroinflamm. 2024;11(3):e200213.
1286.	Ellidag H, Kurtulus F, Yaman A, Eren E, Yılmaz N, Aydin O, et al. Serum iron metabolism markers including hepcidin in multiple sclerosis patients. Neurochemical Journal. 2014.
1287.	Ellidag HY, Kurtulus F, Yaman A, Eren E, Yilmaz N, Aydin O, et al. Serum iron metabolism markers including hepcidin in multiple sclerosis patients. NEUROCHEMICAL JOURNAL. 2014;8(3):226-30.
1288.	Ellidag HY, Kurtulus F, Yaman A, Eren E, Yilmaz N, Aydin O, et al. Serum iron metabolism markers including hepcidin in multiple sclerosis patients. NEUROCHEMICAL JOURNAL. 2014;8(3):226-30.
1289.	Ellidag HY, Kurtulus F, Yaman A, Eren E, Yilmaz N, Aydin O, et al. Serum iron metabolism markers including hepcidin in multiple sclerosis patients. NEUROCHEMICAL JOURNAL. 2014;8(3):226-30.
1290.	Elovaara I, Apostolski S, van Doorn P, Gilhus NE, Hietaharju A, Honkaniemi J, et al. EFNS guidelines for the use of intravenous immunoglobulin in treatment of neurological diseases: EFNS task force on the use of intravenous immunoglobulin in treatment of neurological diseases. Eur J Neurol. 2008;15(9):893-908.
1291.	Elwenspoek MMC, Jackson J, O'Donnell R, Sinobas A, Dawson S, Everitt H, et al. The accuracy of diagnostic indicators for coeliac disease: A systematic review and meta-analysis. PLoS One. 2021;16(10):e0258501.
1292.	Emard JF, Thouez JP, Gauvreau D. Neurodegenerative diseases and risk factors: a literature review. Soc Sci Med. 1995;40(6):847-58.
1293.	Emmerich J, Bachert P, Ladd ME, Straub S. On the separation of susceptibility sources in quantitative susceptibility mapping: Theory and phantom validation with an in vivo application to multiple sclerosis lesions of different age. JOURNAL OF MAGNETIC RESONANCE. 2021;330.
1294.	Emmerich J, Bachert P, Ladd ME, Straub S. On the separation of susceptibility sources in quantitative susceptibility mapping: Theory and phantom validation with an in vivo application to multiple sclerosis lesions of different age. Journal of Magnetic Resonance. 2021.
1295.	Emmerich J, Bachert P, Ladd ME, Straub S. On the separation of susceptibility sources in quantitative susceptibility mapping: Theory and phantom validation with an in vivo application to multiple sclerosis lesions of different age. J Magn Reson. 2021;330:107033.
1296.	Emmerich J, Bachert P, Ladd ME, Straub S. On the separation of susceptibility sources in quantitative susceptibility mapping: Theory and phantom validation with an in vivo application to multiple sclerosis lesions of different age. JOURNAL OF MAGNETIC RESONANCE. 2021;330.
1297.	Engberink RD, van der Pol SM, Walczak P, van der Toorn A, Viergever MA, Dijkstra CD, et al. Magnetic resonance imaging of monocytes labeled with ultrasmall superparamagnetic particles of iron oxide using magnetoelectroporation in an animal model of multiple sclerosis. Mol Imaging. 2010;9(5):268-77.
1298.	Engberink RDO, Blezer ELA, Dijkstra CD, van der Pol SMA, van der Toorn A, de Vries HE. Dynamics and fate of USPIO in the central nervous system in experimental autoimmune encephalomyelitis. NMR IN BIOMEDICINE. 2010;23(9):1087-96.
1299.	Engberink RDO, Blezer ELA, Dijkstra CD, van der Pol SMA, van der Toorn A, de Vries HE. Dynamics and fate of USPIO in the central nervous system in experimental autoimmune encephalomyelitis. NMR IN BIOMEDICINE. 2010;23(9):1087-96.
1300.	Engberink RDO, van der Pol SMA, Walczak P, van der Toorn A, Viergever MA, Dijkstra CD, et al. Magnetic Resonance Imaging of Monocytes Labeled with Ultrasmall Superparamagnetic Particles of Iron Oxide Using Magnetoelectroporation in an Animal Model of Multiple Sclerosis. MOLECULAR IMAGING. 2010;9(5):268-77.
1301.	Engberink RDO, van der Pol SMA, Walczak P, van der Toorn A, Viergever MA, Dijkstra CD, et al. Magnetic Resonance Imaging of Monocytes Labeled with Ultrasmall Superparamagnetic Particles of Iron Oxide Using Magnetoelectroporation in an Animal Model of Multiple Sclerosis. MOLECULAR IMAGING. 2010;9(5):268-77.
1302.	Engberink RDO, van der Pol SMA, Walczak P, van der Toorn A, Viergever MA, Dijkstra CD, et al. Magnetic Resonance Imaging of Monocytes Labeled with Ultrasmall Superparamagnetic Particles of Iron Oxide Using Magnetoelectroporation in an Animal Model of Multiple Sclerosis. MOLECULAR IMAGING. 2010;9(5):268-77.
1303.	Engberink RDO, Van Der Pol SMA, Walczak P, Van Der Toorn A, Viergever MA, Dijkstra CD, et al. Magnetic resonance imaging of monocytes labeled with ultrasmall superparamagnetic particles of iron oxide using magnetoelectroporation in an animal model of multiple sclerosis. Molecular imaging. 2010;9(5):7290-2010.
1304.	Engell T, Hvidberg A, Uhrenholdt A. Multiple sclerosis: periphlebitis retinalis et cerebro-spinalis. A correlation between periphlebitis retinalis and abnormal technetium brain scintigraphy. Acta Neurol Scand. 1984;69(5):293-7.
1305.	Entezari M, Ehrampoush MH, Rahimdel A, Shahi MA, Keyghobady N, Jalili M, et al. Is there a relationship between homes' radon gas of MS and non-MS individuals, and the patients' paraclinical magnetic resonance imaging and visually evoked potentials in Yazd-Iran? Environ Sci Pollut Res Int. 2021;28(7):8907-14.
1306.	Erbay MF, Kamisli O, Karatoprak NB. Can T2 blackout effect be a marker of iron accumulation in brains of multiple sclerosis patients? BRITISH JOURNAL OF RADIOLOGY. 2020;93(1113).
1307.	Erbay MF, Kamişli Ö, Karatoprak NB. Can T2 blackout effect be a marker of iron accumulation in brains of multiple sclerosis patients? British Journal of Radiology. 2020.
1308.	Erbay MF, Kamisli O, Karatoprak NB. Can T2 blackout effect be a marker of iron accumulation in brains of multiple sclerosis patients? BRITISH JOURNAL OF RADIOLOGY. 2020;93(1113).
1309.	Erbay MF, Kamisli O, Karatoprak NB. Can T2 blackout effect be a marker of iron accumulation in brains of multiple sclerosis patients? BRITISH JOURNAL OF RADIOLOGY. 2020;93(1113).
1310.	Erbay MF, Kamışlı Ö, Karatoprak NB. Can T2 blackout effect be a marker of iron accumulation in brains of multiple sclerosis patients? Br J Radiol. 2020;93(1113):20200552.
1311.	Erbay MF, Kamışlı Ö, Karatoprak NB. Can T2 blackout effect be a marker of iron accumulation in brains of multiple sclerosis patients? The British Journal of Radiology. 2020;93(1113):20200552.
1312.	Ernerudh J, Fredriksson S, Olsson T, Forsberg P. Leukocyte types in cerebrospinal fluid and peripheral blood enumerated immunoenzymatically in aseptic meningitis and the Guillain-Barré syndrome. Acta Neurol Scand. 1989;79(1):68-74.
1313.	Ernst TM, Schlamann M, Timmann D. MRI aspects: Conventional, SWI, DTI.  Essentials of Cerebellum and Cerebellar Disorders: A Primer for Graduate Students2016.
1314.	Eschborn M, Pawlitzki M, Wirth T, Nelke C, Pfeuffer S, Schulte-Mecklenbeck A, et al. Evaluation of Age-Dependent Immune Signatures in Patients With Multiple Sclerosis. Neurol Neuroimmunol Neuroinflamm. 2021;8(6).
1315.	Escribano BM, Muñoz-Jurado A, Luque E, Conde C, Feijóo M, LaTorre M, et al. Lactose and Casein Cause Changes on Biomarkers of Oxidative Damage and Dysbiosis in an Experimental Model of Multiple Sclerosis. CNS Neurol Disord Drug Targets. 2022;21(8):680-92.
1316.	Escudié JB, Rance B, Malamut G, Khater S, Burgun A, Cellier C, et al. A novel data-driven workflow combining literature and electronic health records to estimate comorbidities burden for a specific disease: a case study on autoimmune comorbidities in patients with celiac disease. BMC Med Inform Decis Mak. 2017;17(1):140.
1317.	Eskreis-Winkler S, Deh K, Gupta A, Liu T, Wisnieff C, Jin M, et al. Multiple sclerosis lesion geometry in quantitative susceptibility mapping (QSM) and phase imaging. J Magn Reson Imaging. 2015;42(1):224-9.
1318.	Eskreis-Winkler S, Zhang Y, Zhang J, Liu Z, Dimov A, Gupta A, et al. The clinical utility of QSM: disease diagnosis, medical management, and surgical planning. NMR in Biomedicine. 2017.
1319.	Eskreis-Winkler S, Zhang Y, Zhang J, Liu Z, Dimov A, Gupta A, et al. The clinical utility of QSM: disease diagnosis, medical management, and surgical planning. NMR Biomed. 2017;30(4).
1320.	Eskreis-Winkler S, Zhang Y, Zhang JW, Liu Z, Dimov A, Gupta A, et al. The clinical utility of QSM: disease diagnosis, medical management, and surgical planning. NMR IN BIOMEDICINE. 2017;30(4).
1321.	Eskreis-Winkler S, Zhang Y, Zhang JW, Liu Z, Dimov A, Gupta A, et al. The clinical utility of QSM: disease diagnosis, medical management, and surgical planning. NMR IN BIOMEDICINE. 2017;30(4).
1322.	Esmaeilnejad S, Semnanian S, Javan M. Metformin Protects Myelin from Degeneration in A Mouse Model of Iysophosphatidylcholine-Induced Demyelination in The Optic Chiasm. Cell J. 2021;23(1):119-28.
1323.	Esposito P, Gheorghe D, Kandere K, Pang X, Connolly R, Jacobson S, et al. Acute stress increases permeability of the blood-brain-barrier through activation of brain mast cells. Brain Res. 2001;888(1):117-27.
1324.	Etemadifar M, Abtahi SH, Dehghani A, Abtahi MA, Akbari M, Tabrizi N, et al. Myasthenia Gravis during the Course of Neuromyelitis Optica. Case Rep Neurol. 2011;3(3):268-73.
1325.	Etemadifar M, Kiani R, Mehrabi B, Fereidan-Esfahani M, editors. The association of multiple sclerosis prevalence and the soil heavy metal in Isfahan, Iran: one step closer to understanding etiology2014: SAGE PUBLICATIONS LTD 1 OLIVERS YARD, 55 CITY ROAD, LONDON EC1Y 1SP, ENGLAND.
1326.	Etemadifar M, Kiani R, Mehrabi B, Fereidan-Esfahani M. The association of multiple sclerosis prevalence and the soil heavy metal in Isfahan, Iran: one step closer to understanding etiology. MULTIPLE SCLEROSIS JOURNAL. 2014;20:216-.
1327.	Etemadifar M, Mehrabi B, Kiani-Peykani R, Abtahi SH, Nekouie-Isfahani K, Ramagopalan SV, et al. Soil heavy metals are associated with the distribution of multiple sclerosis in Isfahan, Iran. ACTA NEUROLOGICA SCANDINAVICA. 2016;134(4):292-9.
1328.	Etemadifar M, Mehrabi B, Kiani-Peykani R, Abtahi SH, Nekouie-Isfahani K, Ramagopalan SV, et al. Soil heavy metals are associated with the distribution of multiple sclerosis in Isfahan, Iran. Acta Neurologica Scandinavica. 2016.
1329.	Etemadifar M, Mehrabi B, Kiani-Peykani R, Abtahi SH, Nekouie-Isfahani K, Ramagopalan SV, et al. Soil heavy metals are associated with the distribution of multiple sclerosis in Isfahan, Iran. ACTA NEUROLOGICA SCANDINAVICA. 2016;134(4):292-9.
1330.	Etemadifar M, Mehrabi B, Kiani-Peykani R, Abtahi SH, Nekouie-Isfahani K, Ramagopalan SV, et al. Soil heavy metals are associated with the distribution of multiple sclerosis in Isfahan, Iran. Acta Neurol Scand. 2016;134(4):292-9.
1331.	Etemadifar M, Mehrabi B, Kiani-Peykani R, Abtahi SH, Nekouie-Isfahani K, Ramagopalan SV, et al. Soil heavy metals are associated with the distribution of multiple sclerosis in Isfahan, Iran. ACTA NEUROLOGICA SCANDINAVICA. 2016;134(4):292-9.
1332.	Etemadifar M, Mehrabi B, Kiani‐Peykani R, Abtahi SH, Nekouie‐Isfahani K, Ramagopalan SV, et al. Soil heavy metals are associated with the distribution of multiple sclerosis in Isfahan, Iran. Acta Neurologica Scandinavica. 2016;134(4):292-9.
1333.	Evagorou O, Arvaniti A, Angelopoulou C, Mavraki E, Mikellides G, Samakouri M. "Sleeping Beauty Syndrome" and Psychosis as Precursory Symptoms of Multiple Sclerosis: A Rare Case and Literature Review. J Nerv Ment Dis. 2021;209(12):933-5.
1334.	Exley C, Mamutse G, Korchazhkina O, Pye E, Srekopytov S, Polwart A, et al. Elevated urinary excretion of aluminium and iron in multiple sclerosis. JOURNAL OF NEUROLOGY. 2006;253:80-.
1335.	Exley C, Mamutse G, Korchazhkina O, Pye E, Strekopytov S, Polwart A, et al. Elevated urinary excretion of aluminium and iron in multiple sclerosis. Multiple Sclerosis. 2006.
1336.	Exley C, Mamutse G, Korchazhkina O, Pye E, Strekopytov S, Polwart A, et al. Elevated urinary excretion of aluminium and iron in multiple sclerosis. Mult Scler. 2006;12(5):533-40.
1337.	Exley C, Mamutse G, Korchazhkina O, Pye E, Strekopytov S, Polwart A, et al. Elevated urinary excretion of aluminium and iron in multiple sclerosis. Multiple Sclerosis Journal. 2006;12(5):533-40.
1338.	Exley C, Mamutse G, Korchozhkina O, Pye E, Strekopytov S, Polwart A, et al. Elevated urinary excretion of aluminium and iron in multiple sclerosis. MULTIPLE SCLEROSIS JOURNAL. 2006;12(5):533-40.
1339.	Exley C, Mamutse G, Korchozhkina O, Pye E, Strekopytov S, Polwart A, et al. Elevated urinary excretion of aluminium and iron in multiple sclerosis. MULTIPLE SCLEROSIS JOURNAL. 2006;12(5):533-40.
1340.	Exley C, Mamutse G, Korchozhkina O, Pye E, Strekopytov S, Polwart A, et al. Elevated urinary excretion of aluminium and iron in multiple sclerosis. MULTIPLE SCLEROSIS JOURNAL. 2006;12(5):533-40.
1341.	Eyeson J, House I, Yang YH, Warnakulasuriya KA. Relationship between mercury levels in blood and urine and complaints of chronic mercury toxicity from amalgam restorations. Br Dent J. 2010;208(4):E7; discussion 162-3.
1342.	Fagone P, Patti F, Mangano K, Mammana S, Coco M, Touil-Boukoffa C, et al. Heme oxygenase-1 expression in peripheral blood mononuclear cells correlates with disease activity in multiple sclerosis. JOURNAL OF NEUROIMMUNOLOGY. 2013;261(1-2):82-6.
1343.	Fagone P, Patti F, Mangano K, Mammana S, Coco M, Touil-Boukoffa C, et al. Heme oxygenase-1 expression in peripheral blood mononuclear cells correlates with disease activity in multiple sclerosis. Journal of Neuroimmunology. 2013.
1344.	Fagone P, Patti F, Mangano K, Mammana S, Coco M, Touil-Boukoffa C, et al. Heme oxygenase-1 expression in peripheral blood mononuclear cells correlates with disease activity in multiple sclerosis. J Neuroimmunol. 2013;261(1-2):82-6.
1345.	Fagone P, Patti F, Mangano K, Mammana S, Coco M, Touil-Boukoffa C, et al. Heme oxygenase-1 expression in peripheral blood mononuclear cells correlates with disease activity in multiple sclerosis. JOURNAL OF NEUROIMMUNOLOGY. 2013;261(1-2):82-6.
1346.	Faissner S, Bongert M, Gold R. Progression in multiple sclerosis - arrest due to new therapeutics. Arzneimitteltherapie. 2021.
1347.	Faissner S, Gold R. New therapeutic approaches in progressive multiple sclerosis (Reprinted). FORTSCHRITTE DER NEUROLOGIE PSYCHIATRIE. 2019;87(11):653-71.
1348.	Faissner S, Gold R. Progressive multiple sclerosis: latest therapeutic developments and future directions. THERAPEUTIC ADVANCES IN NEUROLOGICAL DISORDERS. 2019;12.
1349.	Faissner S, Gold R. Progressive multiple sclerosis: latest therapeutic developments and future directions. Therapeutic Advances in Neurological Disorders. 2019.
1350.	Faissner S, Gold R. [New therapeutic approaches in progressive multiple sclerosis]. Fortschr Neurol Psychiatr. 2019;87(11):653-71.
1351.	Faissner S, Gold R. Progressive multiple sclerosis: latest therapeutic developments and future directions. Ther Adv Neurol Disord. 2019;12:1756286419878323.
1352.	Faissner S, Gold R. New therapeutic approaches in progressive multiple sclerosis (Reprinted). FORTSCHRITTE DER NEUROLOGIE PSYCHIATRIE. 2019;87(11):653-71.
1353.	Faissner S, Mahjoub Y, Mishra M, Haupeltshofer S, Hahn JN, Gold R, et al. Unexpected additive effects of minocycline and hydroxychloroquine in models of multiple sclerosis: Prospective combination treatment for progressive disease? MULTIPLE SCLEROSIS JOURNAL. 2018;24(12):1543-56.
1354.	Faissner S, Mahjoub Y, Mishra M, Haupeltshofer S, Hahn JN, Gold R, et al. Unexpected additive effects of minocycline and hydroxychloroquine in models of multiple sclerosis: Prospective combination treatment for progressive disease? Multiple Sclerosis Journal. 2018.
1355.	Faissner S, Mahjoub Y, Mishra M, Haupeltshofer S, Hahn JN, Gold R, et al. Unexpected additive effects of minocycline and hydroxychloroquine in models of multiple sclerosis: Prospective combination treatment for progressive disease? Mult Scler. 2018;24(12):1543-56.
1356.	Faissner S, Mahjoub Y, Mishra M, Haupeltshofer S, Hahn JN, Gold R, et al. Unexpected additive effects of minocycline and hydroxychloroquine in models of multiple sclerosis: Prospective combination treatment for progressive disease? MULTIPLE SCLEROSIS JOURNAL. 2018;24(12):1543-56.
1357.	Faissner S, Mishra M, Kaushik DK, Wang J, Fan Y, Silva C, et al. Systematic screening of generic drugs for progressive multiple sclerosis identifies clomipramine as a promising therapeutic. Nature Communications. 2017.
1358.	Faissner S, Mishra M, Kaushik DK, Wang J, Fan Y, Silva C, et al. Systematic screening of generic drugs for progressive multiple sclerosis identifies clomipramine as a promising therapeutic. Nat Commun. 2017;8(1):1990.
1359.	Faissner S, Mishra M, Kaushik DK, Wang JX, Fan Y, Silva C, et al. Systematic screening of generic drugs for progressive multiple sclerosis identifies clomipramine as a promising therapeutic. NATURE COMMUNICATIONS. 2017;8.
1360.	Faissner S, Mishra M, Kaushik DK, Wang JX, Fan Y, Silva C, et al. Systematic screening of generic drugs for progressive multiple sclerosis identifies clomipramine as a promising therapeutic. NATURE COMMUNICATIONS. 2017;8.
1361.	Faivre A, Robinet E, Guye M, Rousseau C, Maarouf A, Le Troter A, et al. Depletion of brain functional connectivity enhancement leads to disability progression in multiple sclerosis: A longitudinal resting-state fMRI study. MULTIPLE SCLEROSIS JOURNAL. 2016;22(13):1695-708.
1362.	Faivre A, Robinet E, Guye M, Rousseau C, Maarouf A, Le Troter A, et al. Depletion of brain functional connectivity enhancement leads to disability progression in multiple sclerosis: A longitudinal resting-state fMRI study. MULTIPLE SCLEROSIS JOURNAL. 2016;22(13):1695-708.
1363.	Faivre A, Robinet E, Guye M, Rousseau C, Maarouf A, Le Troter A, et al. Depletion of brain functional connectivity enhancement leads to disability progression in multiple sclerosis: A longitudinal resting-state fMRI study. MULTIPLE SCLEROSIS JOURNAL. 2016;22(13):1695-708.
1364.	Faivre A, Robinet E, Rousseau C, Maarouf A, Le Troter A, Zaaraoui W, et al. Disruption of brain functional reorganization leads to disability progression in multiple sclerosis. MULTIPLE SCLEROSIS JOURNAL. 2014;20:305-6.
1365.	Fakharzadeh S, Sahraian MA, Hafizi M, Kalanaky S, Masoumi Z, Mahdavi M, et al. The therapeutic effects of MSc1 nanocomplex, synthesized by nanochelating technology, on experimental autoimmune encephalomyelitic C57/BL6 mice. INTERNATIONAL JOURNAL OF NANOMEDICINE. 2014;9:3841-53.
1366.	Fakharzadeh S, Sahraian MA, Hafizi M, Kalanaky S, Masoumi Z, Mahdavi M, et al. The therapeutic effects of MSc1 nanocomplex, synthesized by nanochelating technology, on experimental autoimmune encephalomyelitic C57/Bl6 mice. International Journal of Nanomedicine. 2014.
1367.	Fakharzadeh S, Sahraian MA, Hafizi M, Kalanaky S, Masoumi Z, Mahdavi M, et al. The therapeutic effects of MSc1 nanocomplex, synthesized by nanochelating technology, on experimental autoimmune encephalomyelitic C57/BL6 mice. Int J Nanomedicine. 2014;9:3841-53.
1368.	Fakharzadeh S, Sahraian MA, Hafizi M, Kalanaky S, Masoumi Z, Mahdavi M, et al. The therapeutic effects of MSc1 nanocomplex, synthesized by nanochelating technology, on experimental autoimmune encephalomyelitic C57/BL6 mice. INTERNATIONAL JOURNAL OF NANOMEDICINE. 2014;9:3841-53.
1369.	Fan X, Lin C, Han J, Jiang X, Zhu J, Jin T. Follicular Helper CD4+ T Cells in Human Neuroautoimmune Diseases and Their Animal Models. Mediators Inflamm. 2015;2015:638968.
1370.	Fan X, Zhang H, Cheng Y, Jiang X, Zhu J, Jin T. Double Roles of Macrophages in Human Neuroimmune Diseases and Their Animal Models. Mediators Inflamm. 2016;2016:8489251.
1371.	Fanara S, Aprile M, Iacono S, Schirò G, Bianchi A, Brighina F, et al. The Role of Nutritional Lifestyle and Physical Activity in Multiple Sclerosis Pathogenesis and Management: A Narrative Review. Nutrients. 2021;13(11).
1372.	Fang F, Sveinsson O, Thormar G, Granqvist M, Askling J, Lundberg IE, et al. The autoimmune spectrum of myasthenia gravis: a Swedish population-based study. J Intern Med. 2015;277(5):594-604.
1373.	Farhangi S, Karimi E, Khajeh K, Hosseinkhani S, Javan M. Peptide mediated targeted delivery of gold nanoparticles into the demyelination site ameliorates myelin impairment and gliosis. Nanomedicine. 2023;47:102609.
1374.	Farlow MR, Edwards MK, Kolar OJ, Stevens JC, Yu PL. Magnetic resonance imaging in multiple sclerosis: analysis of correlations to peripheral blood and spinal fluid abnormalities. Neurology. 1987;37(9):1527-30.
1375.	Farran EK, Waggas DS, Alkhunani TA, Almuwallad SA, Aljohani RA. Assessment of Multiple Sclerosis Awareness and Knowledge among the Community of Jeddah, Saudi Arabia. J Neurosci Rural Pract. 2021;12(4):733-8.
1376.	Farrokhi M, Dabirzadeh M, Dastravan N, Etemadifar M, Ghadimi K, Saadatpour Z, et al. Mannose-binding Lectin Mediated Complement Pathway in Autoimmune Neurological Disorders. Iran J Allergy Asthma Immunol. 2016;15(3):251-6.
1377.	Fathi F, Sadjadi MS, Cherati MG. Systematic review: Superparamagnetic Iron Oxide nanoparticles as contrast agents in diagnosis of multiple sclerosis. INTERNATIONAL JOURNAL OF NANO DIMENSION. 2016;7(4):270-7.
1378.	Fathi F, Sadjadi MS, Cherati MG. Systematic review: Superparamagnetic Iron Oxide nanoparticles as contrast agents in diagnosis of multiple sclerosis. INTERNATIONAL JOURNAL OF NANO DIMENSION. 2016;7(4):270-7.
1379.	Fathi F, Seyed Sadjadi MA, Ghafari Cherati M. Systematic review: superparamagnetic iron oxide nanoparticles as contrast agents in diagnosis of multiple sclerosis. International Journal of Nano Dimension. 2016;7(4):270-7.
1380.	Fathi M, Pustokhina I, Kuznetsov SV, Khayrullin M, Hojjat-Farsangi M, Karpisheh V, et al. T-cell immunoglobulin and ITIM domain, as a potential immune checkpoint target for immunotherapy of colorectal cancer. IUBMB Life. 2021;73(5):726-38.
1381.	Fathman CG, Yip L, Gómez-Martín D, Yu M, Seroogy CM, Hurt CR, et al. How GRAIL controls Treg function to maintain self-tolerance. Front Immunol. 2022;13:1046631.
1382.	Fazeli AS, Nasrabadi D, Pouya A, Mirshavaladi S, Sanati MH, Baharvand H, et al. Proteome analysis of post-transplantation recovery mechanisms of an EAE model of multiple sclerosis treated with embryonic stem cell-derived neural precursors. JOURNAL OF PROTEOMICS. 2013;94:437-50.
1383.	Fazeli AS, Nasrabadi D, Pouya A, Mirshavaladi S, Sanati MH, Baharvand H, et al. Proteome analysis of post-transplantation recovery mechanisms of an EAE model of multiple sclerosis treated with embryonic stem cell-derived neural precursors. Journal of Proteomics. 2013.
1384.	Fazeli AS, Nasrabadi D, Pouya A, Mirshavaladi S, Sanati MH, Baharvand H, et al. Proteome analysis of post-transplantation recovery mechanisms of an EAE model of multiple sclerosis treated with embryonic stem cell-derived neural precursors. J Proteomics. 2013;94:437-50.
1385.	Fazeli AS, Nasrabadi D, Pouya A, Mirshavaladi S, Sanati MH, Baharvand H, et al. Proteome analysis of post-transplantation recovery mechanisms of an EAE model of multiple sclerosis treated with embryonic stem cell-derived neural precursors. JOURNAL OF PROTEOMICS. 2013;94:437-50.
1386.	Feagan BG, Schreiber S, Afzali A, Rieder F, Hyams J, Kollengode K, et al. Ozanimod as a novel oral small molecule therapy for the treatment of Crohn's disease: The YELLOWSTONE clinical trial program. Contemp Clin Trials. 2022;122:106958.
1387.	Feijoo de Freixo M, Jiménez García M, Martínez Muerza F, Lunar Domínguez A. [Painful tonic seizures in multiple sclerosis. Clinical and electromyographic aspects (author's transl)]. Med Clin (Barc). 1981;76(10):454-6.
1388.	Feijoo De Freixo M, Jimenez Garcia M, Martinez Muerza F, Lunar Dominiguez A. Painful tonic seizures in multiple sclerosis. Clinical and electromyographic aspects. Medicina Clinica. 1981.
1389.	Feldman DS, Smith DK, editors. COPPER-ZINC-SELENIUM INTERACTIONS IN MULTIPLE-SCLEROSIS (MS)1985: AMER SOC CLINICAL NUTRITION 9650 ROCKVILLE PIKE, SUBSCRIPTIONS, RM L-2310 ….
1390.	Feldman DS, Smith DK. COPPER-ZINC-SELENIUM INTERACTIONS IN MULTIPLE-SCLEROSIS (MS). AMERICAN JOURNAL OF CLINICAL NUTRITION. 1985;41(4):866-.
1391.	Feldman DS, Smith DK. COPPER-ZINC-SELENIUM INTERACTIONS IN MULTIPLE-SCLEROSIS (MS). CLINICAL RESEARCH. 1985;33(2):A703-A.
1392.	Feng L, Chao J, Ye P, Luong Q, Sun G, Liu W, et al. Developing Hypoimmunogenic Human iPSC-Derived Oligodendrocyte Progenitor Cells as an Off-The-Shelf Cell Therapy for Myelin Disorders. Adv Sci (Weinh). 2023;10(23):e2206910.
1393.	Feremutsch K. Multiple sclerosis and schizophrenia as a syndrome with trace element illnesses. KARGER ALLSCHWILERSTRASSE 10, CH-4009 BASEL, SWITZERLAND; 1952.
1394.	Ferguson TB, Clifford DB, Montgomery EB, Bruns KA, McGregor PJ, Trotter JL. Thymectomy in multiple sclerosis. Two preliminary trials. J Thorac Cardiovasc Surg. 1983;85(1):88-93.
1395.	Ferini-Strambi L, Carli G, Casoni F, Galbiati A. Restless Legs Syndrome and Parkinson Disease: A Causal Relationship Between the Two Disorders? FRONTIERS IN NEUROLOGY. 2018;9.
1396.	Ferini-Strambi L, Carli G, Casoni F, Galbiati A. Restless legs syndrome and Parkinson disease: A causal relationship between the two disorders? Frontiers in Neurology. 2018.
1397.	Ferini-Strambi L, Carli G, Casoni F, Galbiati A. Restless Legs Syndrome and Parkinson Disease: A Causal Relationship Between the Two Disorders? Front Neurol. 2018;9:551.
1398.	Fernandes MGF, Mohammadnia A, Pernin F, Schmitz-Gielsdorf LE, Hodgins C, Cui QL, et al. Mechanisms of metabolic stress induced cell death of human oligodendrocytes: relevance for progressive multiple sclerosis. Acta Neuropathol Commun. 2023;11(1):108.
1399.	Fernandes SJ, Ericsson M, Khademi M, Jagodic M, Olsson T, Gomez-Cabrero D, et al. Deep characterization of paired chromatin and transcriptomes in four immune cell types from multiple sclerosis patients. Epigenomics. 2021;13(20):1607-18.
1400.	Fernandez O, Aladro Y, Arroyo R, Brieva L, Calles-Hernandez MC, Carrascal P, et al. 12th Post-ECTRIMS Meeting: review of the novelties from the 2019 ECTRIMS Congress (I). REVISTA DE NEUROLOGIA. 2020;70(10):379-89.
1401.	Fernandez O, Costa-Frossard L, Martínez-Ginés ML, Montero P, Prieto-González JM, Ramió-Torrentà L. Integrated Management of Multiple Sclerosis Spasticity and Associated Symptoms Using the Spasticity-Plus Syndrome Concept: Results of a Structured Specialists' Discussion Using the Workmat(®) Methodology. Front Neurol. 2021;12:722801.
1402.	Fernando MM, Stevens CR, Walsh EC, De Jager PL, Goyette P, Plenge RM, et al. Defining the role of the MHC in autoimmunity: a review and pooled analysis. PLoS Genet. 2008;4(4):e1000024.
1403.	Ferraldeschi M, Romano S, Buscarinu MC, Fornasiero A, Mechelli R, Cerasoli B, et al. Chemical Elements and Oxidative Status in Neuroinflammation.  Biometals in Neurodegenerative Diseases: Mechanisms and Therapeutics2017.
1404.	Ferraro D, Simone AM, Adani G, Vitetta F, Mauri C, Strumia S, et al. Definitive childlessness in women with multiple sclerosis: a multicenter study. Neurol Sci. 2017;38(8):1453-9.
1405.	Ferreira AC, Da Mesquita S, Sousa JC, Correia-Neves M, Sousa N, Palha JA, et al. From the periphery to the brain: Lipocalin-2, a friend or foe? PROGRESS IN NEUROBIOLOGY. 2015;131:120-36.
1406.	Ferreira AC, Dá Mesquita S, Sousa JC, Correia-Neves M, Sousa N, Palha JA, et al. From the periphery to the brain: Lipocalin-2, a friend or foe? Progress in Neurobiology. 2015.
1407.	Ferreira AC, S DM, Sousa JC, Correia-Neves M, Sousa N, Palha JA, et al. From the periphery to the brain: Lipocalin-2, a friend or foe? Prog Neurobiol. 2015;131:120-36.
1408.	Ferreira AC, Sousa N, Sousa JC, Marques F. Age-related changes in mice behavior and the contribution of lipocalin-2. Front Aging Neurosci. 2023;15:1179302.
1409.	Ferreira KPZ, Oliveira SR, Kallaur AP, Kaimen-Maciel DR, Lozovoy MAB, de Almeida ERD, et al. Disease progression and oxidative stress are associated with higher serum ferritin levels in patients with multiple sclerosis. J Neurol Sci. 2017;373:236-41.
1410.	Ferrero ME. Neuron Protection by EDTA May Explain the Successful Outcomes of Toxic Metal Chelation Therapy in Neurodegenerative Diseases. Biomedicines. 2022;10(10).
1411.	Ferrero ME. Neuron Protection by EDTA May Explain the Successful Outcomes of Toxic Metal Chelation Therapy in Neurodegenerative Diseases. Biomedicines. 2022;10(10).
1412.	Ferro JM, Oliveira Santos M. Neurology of inflammatory bowel disease. J Neurol Sci. 2021;424:117426.
1413.	Ferro JM, Oliveira SN, Correia L. Neurologic manifestations of inflammatory bowel diseases. Handb Clin Neurol. 2014;120:595-605.
1414.	Ferrò MT, Franciotta D, Riccardi T, D'Adda E, Mainardi E, Montanelli A. A case of multiple sclerosis with atypical onset associated with autoimmune hepatitis and silent coeliac disease. Neurol Sci. 2008;29(1):29-31.
1415.	Fewster ME, Ellison GW, Myers LW, Kirashige EY. Oligodendroglial toxicity by serum and lymphocytes from patients with multiple sclerosis. Neurology. 1975;25(8):735-9.
1416.	Filip P, Dufek M, Mangia S, Michaeli S, Bares M, Schwarz D, et al. Alterations in Sensorimotor and Mesiotemporal Cortices and Diffuse White Matter Changes in Primary Progressive Multiple Sclerosis Detected by Adiabatic Relaxometry. FRONTIERS IN NEUROSCIENCE. 2021;15.
1417.	Filip P, Svatkova A, Carpenter AF, Eberly LE, Nestrasil I, Nissi MJ, et al. Rotating frame MRI relaxations as markers of diffuse white matter abnormalities in multiple sclerosis. NEUROIMAGE-CLINICAL. 2020;26.
1418.	Filip P, Svatkova A, Carpenter AF, Eberly LE, Nestrasil I, Nissi MJ, et al. Rotating frame MRI relaxations as markers of diffuse white matter abnormalities in multiple sclerosis. NeuroImage: Clinical. 2020.
1419.	Filip P, Svatkova A, Carpenter AF, Eberly LE, Nestrasil I, Nissi MJ, et al. Rotating frame MRI relaxations as markers of diffuse white matter abnormalities in multiple sclerosis. Neuroimage Clin. 2020;26:102234.
1420.	Filip P, Svatkova A, Carpenter AF, Eberly LE, Nestrasil I, Nissi MJ, et al. Rotating frame MRI relaxations as markers of diffuse white matter abnormalities in multiple sclerosis. NEUROIMAGE-CLINICAL. 2020;26.
1421.	Filippi M, Agosta F. Closing the clinical-imaging gap in multiple sclerosis? Imaging iron deposition in deep gray matter. Journal of Neuroimaging. 2009.
1422.	Filippi M, Agosta F. Closing the Clinical-Imaging Gap in Multiple Sclerosis ? Imaging Iron Deposition in Deep Gray Matter. JOURNAL OF NEUROIMAGING. 2009;19(1):1-2.
1423.	Filippi M, Agosta F. Closing the clinical-imaging gap in multiple sclerosis? Imaging iron deposition in deep gray matter. J Neuroimaging. 2009;19(1):1-2.
1424.	Filippi M, Agosta F. Closing the clinical-imaging gap in multiple sclerosis? Imaging iron deposition in deep gray matter. Journal of Neuroimaging: Official Journal of the American Society of Neuroimaging. 2009;19(1):1-2.
1425.	Filippi M, Brück W, Chard D, Fazekas F, Geurts JJG, Enzinger C, et al. Association between pathological and MRI findings in multiple sclerosis. Lancet Neurol. 2019;18(2):198-210.
1426.	Filippi M, Bruck W, Chard D, Fazekas F, Geurts JJG, Enzinger C, et al. Association between pathological and MRI findings in multiple sclerosis. LANCET NEUROLOGY. 2019;18(2):198-210.
1427.	Filippi M, Brück W, Chard D, Fazekas F, Geurts JJG, Enzinger C, et al. Association between pathological and MRI findings in multiple sclerosis. The Lancet Neurology. 2019.
1428.	Filippi M, Evangelou N, Kangarlu A, Inglese M, Mainero C, Horsfield MA, et al. Ultra-high-field MR imaging in multiple sclerosis. JOURNAL OF NEUROLOGY NEUROSURGERY AND PSYCHIATRY. 2014;85(1):60-6.
1429.	Filippi M, Evangelou N, Kangarlu A, Inglese M, Mainero C, Horsfield MA, et al. Ultra-high-field MR imaging in multiple sclerosis. Journal of Neurology, Neurosurgery and Psychiatry. 2014.
1430.	Filippi M, Evangelou N, Kangarlu A, Inglese M, Mainero C, Horsfield MA, et al. Ultra-high-field MR imaging in multiple sclerosis. J Neurol Neurosurg Psychiatry. 2014;85(1):60-6.
1431.	Filippi M, Preziosa P, Rocca MA. Multiple sclerosis. In: Masdeu JC, Gonzalez RG, editors. NEUROIMAGING, PT I. 1352016. p. 399-423.
1432.	Filippi M, Preziosa P, Rocca MA. Multiple sclerosis. Handbook of Clinical Neurology2016.
1433.	Filippi M, Preziosa P, Rocca MA. Multiple sclerosis. Handb Clin Neurol. 2016;135:399-423.
1434.	Filippi M, Rocca MA. MR Imaging of Multiple Sclerosis. RADIOLOGY. 2011;259(3):659-81.
1435.	Filippi M, Rocca MA. MR imaging of multiple sclerosis. Radiology. 2011.
1436.	Filippi M, Rocca MA. MR imaging of multiple sclerosis. Radiology. 2011;259(3):659-81.
1437.	Filippi M, Rocca MA, Calabrese M, Sormani MP, Rinaldi F, Perini P, et al. Intracortical lesions: relevance for new MRI diagnostic criteria for multiple sclerosis. Neurology. 2010;75(22):1988-94.
1438.	Filippi M, Rocca MA, De Stefano N, Enzinger C, Fisher E, Horsfield MA, et al. Magnetic Resonance Techniques in Multiple Sclerosis. ARCHIVES OF NEUROLOGY. 2011;68(12):1514-20.
1439.	Filippi M, Rocca MA, De Stefano N, Enzinger C, Fisher E, Horsfield MA, et al. Magnetic resonance techniques in multiple sclerosis: The present and the future. Archives of Neurology. 2011.
1440.	Filippi M, Rocca MA, De Stefano N, Enzinger C, Fisher E, Horsfield MA, et al. Magnetic resonance techniques in multiple sclerosis: the present and the future. Arch Neurol. 2011;68(12):1514-20.
1441.	Filippini G, Minozzi S, Borrelli F, Cinquini M, Dwan K. Cannabis and cannabinoids for symptomatic treatment for people with multiple sclerosis. Cochrane Database Syst Rev. 2022;5(5):Cd013444.
1442.	Finsterer J, Leutmezer F. Celiac disease with cerebral and peripheral nerve involvement mimicking multiple sclerosis. J Med Life. 2014;7(3):440-4.
1443.	Fisch G. [ON THE ACTIVITY OF CERULOPLASMIN IN NEUROLOGIC AND PSYCHIATRIC DISORDERS WITH SPECIAL REFERENCE TO MULTIPLE SCLEROSIS]. Confin Neurol. 1964;24:257-80.
1444.	Fiscone C, Rundo L, Lugaresi A, Manners DN, Allinson K, Baldin E, et al. Assessing robustness of quantitative susceptibility-based MRI radiomic features in patients with multiple sclerosis. Sci Rep. 2023;13(1):16239.
1445.	Fisher AA, Le Couteur DG. Lead poisoning from complementary and alternative medicine in multiple sclerosis. JOURNAL OF NEUROLOGY NEUROSURGERY AND PSYCHIATRY. 2000;69(5):687-9.
1446.	Fisher AA, Le Couteur DG. Lead poisoning from complementary and alternative medicine in multiple sclerosis. Journal of Neurology Neurosurgery and Psychiatry. 2000.
1447.	Fisher AA, Le Couteur DG. Lead poisoning from complementary and alternative medicine in multiple sclerosis. JOURNAL OF NEUROLOGY NEUROSURGERY AND PSYCHIATRY. 2000;69(5):687-9.
1448.	Fisher AA, Le Couteur DG. Lead poisoning from complementary and alternative medicine in multiple sclerosis. JOURNAL OF NEUROLOGY NEUROSURGERY AND PSYCHIATRY. 2000;69(5):687-9.
1449.	Fisher AA, Le Couteur DG. Lead poisoning from complementary and alternative medicine in multiple sclerosis. Journal of Neurology, Neurosurgery & Psychiatry. 2000;69(5):687-9.
1450.	Fjaer S, Bo L, Myhr KM, Torkildsen O, Wergeland S. Magnetization transfer ratio does not correlate to myelin content in the brain in the MOG-EAE mouse model. NEUROCHEMISTRY INTERNATIONAL. 2015;83-84:28-40.
1451.	Fjær S, Bø L, Myhr KM, Torkildsen O, Wergeland S. Magnetization transfer ratio does not correlate to myelin content in the brain in the MOG-EAE mouse model. Neurochemistry International. 2015.
1452.	Fjær S, Bø L, Myhr KM, Torkildsen Ø, Wergeland S. Magnetization transfer ratio does not correlate to myelin content in the brain in the MOG-EAE mouse model. Neurochem Int. 2015;83-84:28-40.
1453.	Fjaer S, Bo L, Myhr KM, Torkildsen O, Wergeland S. Magnetization transfer ratio does not correlate to myelin content in the brain in the MOG-EAE mouse model. NEUROCHEMISTRY INTERNATIONAL. 2015;83-84:28-40.
1454.	Flammer J, Pache M, Resink T. Vasospasm, its role in the pathogenesis of diseases with particular reference to the eye. PROGRESS IN RETINAL AND EYE RESEARCH. 2001;20(3):319-49.
1455.	Flammer J, Pache M, Resink T. Vasospasm, its role in the pathogenesis of diseases with particular reference to the eye. Progress in Retinal and Eye Research. 2001.
1456.	Flammer J, Pache M, Resink T. Vasospasm, its role in the pathogenesis of diseases with particular reference to the eye. Prog Retin Eye Res. 2001;20(3):319-49.
1457.	Fletcher J, Bishop EL, Harrison SR, Swift A, Cooper SC, Dimeloe SK, et al. Autoimmune disease and interconnections with vitamin D. Endocr Connect. 2022;11(3).
1458.	Floris S, Blezer EL, Schreibelt G, Döpp E, van der Pol SM, Schadee-Eestermans IL, et al. Blood-brain barrier permeability and monocyte infiltration in experimental allergic encephalomyelitis: a quantitative MRI study. Brain. 2004;127(Pt 3):616-27.
1459.	Floris S, Blezer ELA, Schreibelt G, Dopp E, van der Pol SMA, Schadee-Eestermans IL, et al. Blood-brain barrier permeability and monocyte infiltration in experimental allergic encephalomyelitis - A quantitative MRI study. BRAIN. 2004;127:616-27.
1460.	Floris S, Blezer ELA, Schreibelt G, Döpp E, Van Der Pol SMA, Schadee-Eestermans IL, et al. Blood-brain barrier permeability and monocyte infiltration in experimental allergic encephalomyelitis: A quantitative MRI study. Brain. 2004.
1461.	Floris S, Blezer ELA, Schreibelt G, Dopp E, van der Pol SMA, Schadee-Eestermans IL, et al. Blood-brain barrier permeability and monocyte infiltration in experimental allergic encephalomyelitis - A quantitative MRI study. BRAIN. 2004;127:616-27.
1462.	Foley JM, Donovan AM, Moloney WC. Correlation of the zinc sulfate precipitation test with the colloidal gold test in neurosyphilis, multiple sclerosis, and cerebral vascular disease. Journal of neuropathology and experimental neurology. 1951.
1463.	Foley JM, Donovan AM, Moloney WC. Correlation of the zinc sulfate precipitation test with the colloidal gold test in neurosyphilis, multiple sclerosis, and cerebral vascular disease. J Neuropathol Exp Neurol. 1951;10(1):89-91.
1464.	Foote KD, Seignourel P, Fernandez HH, Jacobson C, Rodriguez RL, Okun MS. The use of two DBS leads for the treatment of post-traumatic and Multiple Sclerosis tremor. MOVEMENT DISORDERS. 2005;20:S149-S.
1465.	Forbes JD, Chen CY, Knox NC, Marrie RA, El-Gabalawy H, de Kievit T, et al. A comparative study of the gut microbiota in immune-mediated inflammatory diseases-does a common dysbiosis exist? Microbiome. 2018;6(1):221.
1466.	Førde JL, Herfindal L, Myhr KM, Torkildsen Ø, Mollnes TE, Skrede S. Ocrelizumab and ofatumumab, but not rituximab, trigger complement induction in vitro. Int Immunopharmacol. 2023;124(Pt B):111021.
1467.	Forge JK, Pedchenko TV, LeVine SM. Iron deposits in the central nervous system of SJL mice with experimental allergic encephalomyelitis. LIFE SCIENCES. 1998;63(25):2271-84.
1468.	Forge JK, Pedchenko TV, LeVine SM. Iron deposits in the central nervous system of SJL mice with experimental allergic encephalomyelitis. Life Sciences. 1998.
1469.	Forge JK, Pedchenko TV, LeVine SM. Iron deposits in the central nervous system of SJL mice with experimental allergic encephalomyelitis. Life Sci. 1998;63(25):2271-84.
1470.	Forge JK, Pedchenko TV, LeVine SM. Iron deposits in the central nervous system of SJL mice with experimental allergic encephalomyelitis. LIFE SCIENCES. 1998;63(25):2271-84.
1471.	Foroughi AA, Zare N, Saeedi-Moghadam M, Zeinali-Rafsanjani B, Nazeri M. Correlation between contrast enhanced plaques and plaque diffusion restriction and their signal intensities in FLAIR images in patients who admitted with acute symptoms of multiple sclerosis. J Med Imaging Radiat Sci. 2021;52(1):121-6.
1472.	Forte G, Visconti A, Santucci S, Ghazaryan A, Figà-Talamanca L, Cannoni S, et al. Quantification of chemical elements in blood of patients affected by multiple sclerosis. Annali dell'Istituto Superiore di Sanita. 2005.
1473.	Forte G, Visconti A, Santucci S, Ghazaryan A, Figà-Talamanca L, Cannoni S, et al. Quantification of chemical elements in blood of patients affected by multiple sclerosis. Ann Ist Super Sanita. 2005;41(2):213-6.
1474.	Foster HD. Disease family trees: The possible roles of iodine in goitre, cretinism, multiple sclerosis, amyotrophic lateral sclerosis, Alzheimer's and Parkinson's diseases and cancers of the thyroid, nervous system and skin. Medical Hypotheses. 1987.
1475.	Foster HD. Disease family trees: the possible roles of iodine in goitre, cretinism, multiple sclerosis, amyotrophic lateral sclerosis, Alzheimer's and Parkinson's diseases and cancers of the thyroid, nervous system and skin. Med Hypotheses. 1987;24(3):249-63.
1476.	Foster HD. Reducing the incidence of multiple sclerosis. Environments. 1988.
1477.	Foster HD. The iodine-selenium connection: its possible roles in intelligence, cretinism, sudden infant death syndrome, breast cancer and multiple sclerosis. Med Hypotheses. 1993;40(1):61-5.
1478.	Foster T, Weinstock-Guttman B, Hagemeier J, Carl E, Hojnacki D, Dwyer M, et al. Iron Content in Benign and Non-Benign Patients with Relapsing-Remitting Multiple Sclerosis (P06. 108). AAN Enterprises; 2013.
1479.	Foster T, Weinstock-Guttman B, Hagemeier J, Carl E, Hojnacki D, Dwyer M, et al. Iron Content in Benign and Non-Benign Patients with Relapsing-Remitting Multiple Sclerosis. NEUROLOGY. 2013;80.
1480.	Foster VS, Rash LD, King GF, Rank MM. Acid-Sensing Ion Channels: Expression and Function in Resident and Infiltrating Immune Cells in the Central Nervous System. Front Cell Neurosci. 2021;15:738043.
1481.	Fotio Y, Sasso O, Ciccocioppo R, Piomelli D. Antinociceptive Profile of ARN19702, (2-Ethylsulfonylphenyl)-[(2S)-4-(6-fluoro-1,3-benzothiazol-2-yl)-2-methylpiperazin-1-yl]methanone, a Novel Orally Active N-Acylethanolamine Acid Amidase Inhibitor, in Animal Models. J Pharmacol Exp Ther. 2021;378(2):70-6.
1482.	Franco PG, Pasquini LA, Perez MJ, Rosato-Siri MV, Silvestroff L, Pasquini JM. Paving the way for adequate myelination: The contribution of galectin-3, transferrin and iron. FEBS LETTERS. 2015;589(22):3388-95.
1483.	Franco PG, Pasquini LA, Pérez MJ, Rosato-Siri MV, Silvestroff L, Pasquini JM. Paving the way for adequate myelination: The contribution of galectin-3, transferrin and iron. FEBS Letters. 2015.
1484.	Franco PG, Pasquini LA, Pérez MJ, Rosato-Siri MV, Silvestroff L, Pasquini JM. Paving the way for adequate myelination: The contribution of galectin-3, transferrin and iron. FEBS Lett. 2015;589(22):3388-95.
1485.	Franklin RJ, Blaschuk KL, Bearchell MC, Prestoz LL, Setzu A, Brindle KM, et al. Magnetic resonance imaging of transplanted oligodendrocyte precursors in the rat brain. Neuroreport. 1999;10(18):3961-5.
1486.	Franklin RJM, Blaschuk KL, Bearchell MC, Prestoz LLC, Setzu A, Brindle KM, et al. Magnetic resonance imaging of transplanted oligodendrocyte precursors in the rat brain. NEUROREPORT. 1999;10(18):3961-5.
1487.	Franklin RJM, Blaschuk KL, Bearchell MC, Prestoz LLC, Setzu A, Brindle KM, et al. Magnetic resonance imaging of transplanted oligodendrocyte precursors in the rat brain. NeuroReport. 1999.
1488.	Franklin RJM, Blaschuk KL, Bearchell MC, Prestoz LLC, Setzu A, Brindle KM, et al. Magnetic resonance imaging of transplanted oligodendrocyte precursors in the rat brain. NEUROREPORT. 1999;10(18):3961-5.
1489.	Fransson J, Gómez-Conde AI, Romero-Imbroda J, Fernández O, Leyva L, de Fonseca FR, et al. Activation of Macrophages by Lysophosphatidic Acid through the Lysophosphatidic Acid Receptor 1 as a Novel Mechanism in Multiple Sclerosis Pathogenesis. Mol Neurobiol. 2021;58(2):470-82.
1490.	Fraschilla I, Amatullah H, Rahman RU, Jeffrey KL. Immune chromatin reader SP140 regulates microbiota and risk for inflammatory bowel disease. Cell Host Microbe. 2022;30(10):1370-81.e5.
1491.	Freedman MS, Coyle PK, Comi G, S LS, Damian D, Hyvert Y, et al. Early MRI outcomes in participants with a first clinical demyelinating event at risk of multiple sclerosis in the ORACLE-MS study. Mult Scler J Exp Transl Clin. 2021;7(1):2055217321990852.
1492.	Freeman L, Longbrake EE, Coyle PK, Hendin B, Vollmer T. High-Efficacy Therapies for Treatment-Naïve Individuals with Relapsing-Remitting Multiple Sclerosis. CNS Drugs. 2022;36(12):1285-99.
1493.	Freitas S, Batista S, Afonso AC, Simões MR, de Sousa L, Cunha L, et al. The Montreal Cognitive Assessment (MoCA) as a screening test for cognitive dysfunction in multiple sclerosis. Appl Neuropsychol Adult. 2018;25(1):57-70.
1494.	Frequin S, Lamers KJB, Borm GF, Barkhof F, Jongen PJH, Hommes OR. T-CELL SUBSETS IN THE CEREBROSPINAL-FLUID AND PERIPHERAL-BLOOD OF MULTIPLE-SCLEROSIS PATIENTS TREATED WITH HIGH-DOSE INTRAVENOUS METHYLPREDNISOLONE. ACTA NEUROLOGICA SCANDINAVICA. 1993;88(2):80-6.
1495.	Frequin S, Lamers KJB, Borm GF, Barkhof F, Jongen PJH, Hommes OR. T-CELL SUBSETS IN THE CEREBROSPINAL-FLUID AND PERIPHERAL-BLOOD OF MULTIPLE-SCLEROSIS PATIENTS TREATED WITH HIGH-DOSE INTRAVENOUS METHYLPREDNISOLONE. ACTA NEUROLOGICA SCANDINAVICA. 1993;88(2):80-6.
1496.	Frequin ST, Lamers KJ, Borm GF, Barkhof F, Jongen PJ, Hommes OR. T-cell subsets in the cerebrospinal fluid and peripheral blood of multiple sclerosis patients treated with high-dose intravenous methylprednisolone. Acta Neurol Scand. 1993;88(2):80-6.
1497.	Friend S, Richman S, Bloomgren G, Cristiano LM, Wenten M. Evaluation of pregnancy outcomes from the Tysabri® (natalizumab) pregnancy exposure registry: a global, observational, follow-up study. BMC Neurol. 2016;16(1):150.
1498.	Frischer JM, Haider L, Simeonidou C, Steinberger G, Hametner S, Grigoriadis N, et al., editors. Multiple sclerosis deep grey matter: the relation between demyelination, neurodegeneration, inflammation and iron2014: SAGE PUBLICATIONS LTD 1 OLIVERS YARD, 55 CITY ROAD, LONDON EC1Y 1SP, ENGLAND.
1499.	Frischer JM, Haider L, Simeonidou C, Steinberger G, Hametner S, Grigoriadis N, et al. Multiple sclerosis deep grey matter: the relation between demyelination, neurodegeneration, inflammation and iron. MULTIPLE SCLEROSIS JOURNAL. 2014;20:16-.
1500.	Frisullo G, Nociti V, Iorio R, Patanella AK, Marti A, Cammarota G, et al. Increased expression of T-bet in circulating B cells from a patient with multiple sclerosis and celiac disease. Hum Immunol. 2008;69(12):837-9.
1501.	Frohman EM, Goodin DS, Calabresi PA, Corboy JR, Coyle PK, Filippi M, et al. The utility of MRI in suspected MS: report of the Therapeutics and Technology Assessment Subcommittee of the American Academy of Neurology. Neurology. 2003;61(5):602-11.
1502.	Frost G, Finlayson H, Saeidiborojeni S, Lagnau P, Reebye R. Perioperative Botulinum Toxin Injections to Enhance Surgical Outcomes in Patients With Spasticity: Preoperative, Intraoperative, and Postoperative Case Reports. Arch Rehabil Res Clin Transl. 2021;3(1):100101.
1503.	Fu X, Liu H, Huang G, Dai SS. The emerging role of neutrophils in autoimmune-associated disorders: effector, predictor, and therapeutic targets. MedComm (2020). 2021;2(3):402-13.
1504.	Fuhua P, Xuhui D, Zhiyang Z, Ying J, Yu Y, Feng T, et al. Antioxidant status of bilirubin and uric acid in patients with myasthenia gravis. Neuroimmunomodulation. 2012;19(1):43-9.
1505.	Fujioka T, Ishida T, Kurihara T, Kinoshita M. [A case of myasthenia gravis associated with multiple sclerosis and positive anticardiolipin antibodies]. Rinsho Shinkeigaku. 1993;33(5):572-4.
1506.	Fujiwara E, Kmech JA, Cobzas D, Sun H, Seres P, Blevins G, et al. Cognitive Implications of Deep Gray Matter Iron in Multiple Sclerosis. AMERICAN JOURNAL OF NEURORADIOLOGY. 2017;38(5):942-8.
1507.	Fujiwara E, Kmech JA, Cobzas D, Sun H, Seres P, Blevins G, et al. Cognitive implications of deep gray matter iron in multiple sclerosis. American Journal of Neuroradiology. 2017.
1508.	Fujiwara E, Kmech JA, Cobzas D, Sun H, Seres P, Blevins G, et al. Cognitive Implications of Deep Gray Matter Iron in Multiple Sclerosis. AMERICAN JOURNAL OF NEURORADIOLOGY. 2017;38(5):942-8.
1509.	Fujiwara E, Kmech JA, Cobzas D, Sun H, Seres P, Blevins G, et al. Cognitive Implications of Deep Gray Matter Iron in Multiple Sclerosis. AJNR Am J Neuroradiol. 2017;38(5):942-8.
1510.	Fujiwara E, Kmech JA, Cobzas D, Sun H, Seres P, Blevins G, et al. Cognitive Implications of Deep Gray Matter Iron in Multiple Sclerosis. AMERICAN JOURNAL OF NEURORADIOLOGY. 2017;38(5):942-8.
1511.	Fujiwara E, Kmech JA, Cobzas D, Sun H, Seres P, Blevins G, et al. Cognitive implications of deep gray matter iron in multiple sclerosis. American Journal of Neuroradiology. 2017;38(5):942-8.
1512.	Fulgente T, Thomas A, Lobefalo L, Mastropasqua L, Gallenga PE, Gambi D, et al. Are VEP abnormalities in optic neuritis (ON) dependent on plaque size? A reappraisal of the physiopathology of ON based on improved MRI and multiple-lead recordings. ITALIAN JOURNAL OF NEUROLOGICAL SCIENCES. 1996;17(1):43-54.
1513.	Fulgente T, Thomas A, Lobefalo L, Mastropasqua L, Gallenga PE, Gambi D, et al. Are VEP abnormalities in optic neuritis (ON) dependent on plaque size? A reappraisal of the physiopathology of ON based on improved MRI and multiple-lead recordings. ITALIAN JOURNAL OF NEUROLOGICAL SCIENCES. 1996;17(1):43-54.
1514.	Fulgenzi A, Zanella SG, Mariani MM, Vietti D, Ferrero ME. A case of multiple sclerosis improvement following removal of heavy metal intoxication Lessons learnt from Matteo's case. BIOMETALS. 2012;25(3):569-76.
1515.	Fulgenzi A, Zanella SG, Mariani MM, Vietti D, Ferrero ME. A case of multiple sclerosis improvement following removal of heavy metal intoxication. BioMetals. 2012.
1516.	Fulgenzi A, Zanella SG, Mariani MM, Vietti D, Ferrero ME. A case of multiple sclerosis improvement following removal of heavy metal intoxication Lessons learnt from Matteo's case. BIOMETALS. 2012;25(3):569-76.
1517.	Fulgenzi A, Zanella SG, Mariani MM, Vietti D, Ferrero ME. A case of multiple sclerosis improvement following removal of heavy metal intoxication: lessons learnt from Matteo's case. Biometals. 2012;25(3):569-76.
1518.	Fulgenzi A, Zanella SG, Mariani MM, Vietti D, Ferrero ME. A case of multiple sclerosis improvement following removal of heavy metal intoxication Lessons learnt from Matteo's case. BIOMETALS. 2012;25(3):569-76.
1519.	Fulgenzi A, Zanella SG, Mariani MM, Vietti D, Ferrero ME. A case of multiple sclerosis improvement following removal of heavy metal intoxication. Biometals. 2012;25(3):569-76.
1520.	Fung EY, Smyth DJ, Howson JM, Cooper JD, Walker NM, Stevens H, et al. Analysis of 17 autoimmune disease-associated variants in type 1 diabetes identifies 6q23/TNFAIP3 as a susceptibility locus. Genes Immun. 2009;10(2):188-91.
1521.	Fung YK, Meade AG, Rack EP, Blotcky AJ. Brain mercury in neurodegenerative disorders. JOURNAL OF TOXICOLOGY-CLINICAL TOXICOLOGY. 1997;35(1):49-54.
1522.	Fung YK, Meade AG, Rack EP, Blotcky AJ. Brain mercury in neurodegenerative disorders. Journal of Toxicology - Clinical Toxicology. 1997.
1523.	Fung YK, Meade AG, Rack EP, Blotcky AJ. Brain mercury in neurodegenerative disorders. J Toxicol Clin Toxicol. 1997;35(1):49-54.
1524.	Fung YK, Meade AG, Rack EP, Blotcky AJ. Brain mercury in neurodegenerative disorders. JOURNAL OF TOXICOLOGY-CLINICAL TOXICOLOGY. 1997;35(1):49-54.
1525.	Furlan R, Martino G, Galbiati F, Poliani PL, Smiroldo S, Bergami A, et al. Caspase-1 regulates the inflammatory process leading to autoimmune demyelination. JOURNAL OF IMMUNOLOGY. 1999;163(5):2403-9.
1526.	Furlan R, Martino G, Galbiati F, Poliani PL, Smiroldo S, Bergami A, et al. Caspase-1 regulates the inflammatory process leading to autoimmune demyelination. JOURNAL OF IMMUNOLOGY. 1999;163(5):2403-9.
1527.	Fuxench ZCC, Mitra N, Hoffstad OJ, Phillips EJ, Margolis DJ. Association between atopic dermatitis, autoimmune illnesses, Epstein-Barr virus, and cytomegalovirus. Arch Dermatol Res. 2023;315(9):2689-92.
1528.	Gacem N, Nait-Oumesmar B. Oligodendrocyte Development and Regenerative Therapeutics in Multiple Sclerosis. Life (Basel). 2021;11(4).
1529.	Gadani SP, Reyes-Mantilla M, Jank L, Harris S, Douglas M, Smith MD, et al. Discordant humoral and T cell immune responses to SARS-CoV-2 vaccination in people with multiple sclerosis on anti-CD20 therapy. EBioMedicine. 2021;73:103636.
1530.	Gaĭkova ON, Bisaga GN, Onishchenko LS, Chikurov AA, Pakhomov IM, Pozdniakov AV, et al. [New morphological data on multiple sclerosis]. Arkh Patol. 2006;68(2):28-34.
1531.	Galarza-Muñoz G, Kennedy-Boone D, Schott G, Bradrick SS, Garcia-Blanco MA. Antisense modulation of IL7R splicing to control sIL7R expression in human CD4(+) T cells. Rna. 2022;28(8):1058-73.
1532.	Galbusera R, Bahn E, Weigel M, Cagol A, Lu PJ, Schaedelin SA, et al. Characteristics, Prevalence, and Clinical Relevance of Juxtacortical Paramagnetic Rims in Patients With Multiple Sclerosis. Neurology. 2024;102(3):e207966.
1533.	Galichet C, Clayton RW, Lovell-Badge R. Novel Tools and Investigative Approaches for the Study of Oligodendrocyte Precursor Cells (NG2-Glia) in CNS Development and Disease. Front Cell Neurosci. 2021;15:673132.
1534.	Gallyas F. Silver staining of micro- and oligodendroglia by means of physical development. Acta Neuropathol. 1970;16(1):35-8.
1535.	Gallyas F. An argyrophil III method for the demonstration of fibrous neuroglia. Acta Morphol Acad Sci Hung. 1981;29(2-3):185-93.
1536.	Ganieva I, Parpieva Y, Khalimova K, Yakubova M. Clinical feature of sensorimotor manifestations as a leading predictor of radiological isolated syndrome in multiple sclerosis. JOURNAL OF THE NEUROLOGICAL SCIENCES. 2019;405.
1537.	Garbo R, Lorenzut S, Del Negro I, Merlino G, Gigli GL, Cargnelutti D, et al. Lower lymphocyte counts and older age are associated with reduced multiple sclerosis disease activity during dimethyl fumarate treatment. Mult Scler Relat Disord. 2021;49:102781.
1538.	García-Beltrán O, Urrutia PJ, Núñez MT. On the Chemical and Biological Characteristics of Multifunctional Compounds for the Treatment of Parkinson's Disease. Antioxidants (Basel). 2023;12(2).
1539.	García-Rodrigo L, Ramos-López C, Sánchez-Tirado E, Agüí L, González-Cortés A, Yáñez-Sedeño P, et al. Label-free electrochemical immunosensing of glial fibrillary acidic protein (GFAP) at synthesized rGO/MoS(2)/AgNPs nanocomposite. Application to the determination in human cerebrospinal fluid. Talanta. 2024;270:125597.
1540.	Garcion E, Sindji L, Nataf S, Brachet P, Darcy F, Montero-Menei CN. Treatment of experimental autoimmune encephalomyelitis in rat by 1,25-dihydroxyvitamin D-3 leads to early effects within the central nervous system. ACTA NEUROPATHOLOGICA. 2003;105(5):438-48.
1541.	Garcion E, Sindji L, Nataf S, Brachet P, Darcy F, Montero-Menei CN. Treatment of experimental autoimmune encephalomyelitis in rat by 1,25-dihydroxyvitamin D-3 leads to early effects within the central nervous system. ACTA NEUROPATHOLOGICA. 2003;105(5):438-48.
1542.	Garehdaghi F, Sarbaz Y. Analyzing global features of magnetic resonance images in widespread neurodegenerative diseases: new hope to understand brain mechanism and robust neurodegenerative disease diagnosis. Med Biol Eng Comput. 2023;61(3):773-84.
1543.	Garg A, Singhal N, Kumar M. Discerning novel drug targets for treating Mycobacterium avium ss. paratuberculosis-associated autoimmune disorders: an in silico approach. Brief Bioinform. 2021;22(3).
1544.	Garvey PH, Rockwell FV. Lead Content of the Spinal Fluid with Special Reference to Multiple Sclerosis. Proceedings of the Society for Experimental Biology and Medicine. 1936;35(1):201-3.
1545.	Gasparini S, Russo M, Dattola V, Ferlazzo E, Aguglia U. Cryptogenic cerebral venous thrombosis in a multiple-sclerosis-patient treated with Alemtuzumab. Mult Scler Relat Disord. 2020;44:102246.
1546.	Gasperoni F. The Innate Chronotoxicity hypothesis: an ubiquitous physiological flaw both unnoticed and inevitable. Could circadian rhythm be an evolutionary mismatch? Clin Ter. 2022;173(1):67-78.
1547.	Gattringer T, Khalil M, Langkammer C, Jehna M, Pichler A, Pinter D, et al. No evidence for increased brain iron deposition in patients with ischemic white matter disease. NEUROBIOLOGY OF AGING. 2016;45:61-3.
1548.	Gattringer T, Khalil M, Langkammer C, Jehna M, Pichler A, Pinter D, et al. No evidence for increased brain iron deposition in patients with ischemic white matter disease. Neurobiology of Aging. 2016.
1549.	Gattringer T, Khalil M, Langkammer C, Jehna M, Pichler A, Pinter D, et al. No evidence for increased brain iron deposition in patients with ischemic white matter disease. Neurobiol Aging. 2016;45:61-3.
1550.	Gattringer T, Khalil M, Langkammer C, Jehna M, Pichler A, Pinter D, et al. No evidence for increased brain iron deposition in patients with ischemic white matter disease. NEUROBIOLOGY OF AGING. 2016;45:61-3.
1551.	Gauberti M, Fournier AP, Docagne F, Vivien D, de Lizarrondo SM. Molecular Magnetic Resonance Imaging of Endothelial Activation in the Central Nervous System. THERANOSTICS. 2018;8(5):1195-212.
1552.	Gauberti M, Fournier AP, Docagne F, Vivien D, de Lizarrondo SM. Molecular magnetic resonance imaging of endothelial activation in the central nervous system. Theranostics. 2018.
1553.	Gauberti M, Fournier AP, Docagne F, Vivien D, Martinez de Lizarrondo S. Molecular Magnetic Resonance Imaging of Endothelial Activation in the Central Nervous System. Theranostics. 2018;8(5):1195-212.
1554.	Gauberti M, Martinez de Lizarrondo S. Molecular MRI of Neuroinflammation: Time to Overcome the Translational Roadblock. Neuroscience. 2021;474:30-6.
1555.	Gauberti M, Montagne A, Quenault A, Vivien D. Molecular magnetic resonance imaging of brain-immune interactions. Front Cell Neurosci. 2014;8:389.
1556.	Gavriilaki M, Kimiskidis VK, Gavriilaki E. Precision Medicine in Neurology: The Inspirational Paradigm of Complement Therapeutics. Pharmaceuticals (Basel). 2020;13(11).
1557.	Ge Y, Jensen JH, Inglese M. Iron accumulation in the deep gray matter of patients with multiple sclerosis measured by magnetic field correlation. Proceedings of the Radiological Society of North America; Chicago. 2005;563.
1558.	Ge Y, Jensen JH, Lu H, Helpern JA, Miles L, Inglese M, et al. Quantitative assessment of iron accumulation in the deep gray matter of multiple sclerosis by magnetic field correlation imaging. AMERICAN JOURNAL OF NEURORADIOLOGY. 2007;28(9):1639-44.
1559.	Ge Y, Jensen JH, Lu H, Helpern JA, Miles L, Inglese M, et al. Quantitative assessment of iron accumulation in the deep gray matter of multiple sclerosis by magnetic field correlation imaging. American Journal of Neuroradiology. 2007.
1560.	Ge Y, Jensen JH, Lu H, Helpern JA, Miles L, Inglese M, et al. Quantitative assessment of iron accumulation in the deep gray matter of multiple sclerosis by magnetic field correlation imaging. AMERICAN JOURNAL OF NEURORADIOLOGY. 2007;28(9):1639-44.
1561.	Ge Y, Jensen JH, Lu H, Helpern JA, Miles L, Inglese M, et al. Quantitative assessment of iron accumulation in the deep gray matter of multiple sclerosis by magnetic field correlation imaging. AJNR Am J Neuroradiol. 2007;28(9):1639-44.
1562.	Ge Y, Jensen JH, Lu H, Helpern JA, Miles L, Inglese M, et al. Quantitative assessment of iron accumulation in the deep gray matter of multiple sclerosis by magnetic field correlation imaging. AMERICAN JOURNAL OF NEURORADIOLOGY. 2007;28(9):1639-44.
1563.	Ge Y, Jensen JH, Lu H, Helpern JA, Miles L, Inglese M, et al. Quantitative assessment of iron accumulation in the deep gray matter of multiple sclerosis by magnetic field correlation imaging. American Journal of Neuroradiology. 2007;28(9):1639-44.
1564.	Ge Y, Sheng H, Chawla S, Kister I, Herbert J, Grossman RI, editors. Cellular and microstructural changes due to iron deposition in multiple sclerosis lesions2014: SAGE PUBLICATIONS LTD 1 OLIVERS YARD, 55 CITY ROAD, LONDON EC1Y 1SP, ENGLAND.
1565.	Ge Y, Sheng H, Chawla S, Kister I, Herbert J, Grossman RI. Cellular and microstructural changes due to iron deposition in multiple sclerosis lesions. MULTIPLE SCLEROSIS JOURNAL. 2014;20:297-.
1566.	Gellein K, Skogholt JH, Aaseth J, Thoresen GB, Lierhagen S, Steinnes E, et al. Trace elements in cerebrospinal fluid and blood from patients with a rare progressive central and peripheral demyelinating disease. JOURNAL OF THE NEUROLOGICAL SCIENCES. 2008;266(1-2):70-8.
1567.	Gellein K, Skogholt JH, Aaseth J, Thoresen GB, Lierhagen S, Steinnes E, et al. Trace elements in cerebrospinal fluid and blood from patients with a rare progressive central and peripheral demyelinating disease. Journal of the Neurological Sciences. 2008.
1568.	Gellein K, Skogholt JH, Aaseth J, Thoresen GB, Lierhagen S, Steinnes E, et al. Trace elements in cerebrospinal fluid and blood from patients with a rare progressive central and peripheral demyelinating disease. J Neurol Sci. 2008;266(1-2):70-8.
1569.	Gellein K, Skogholt JH, Aaseth J, Thoresen GB, Lierhagen S, Steinnes E, et al. Trace elements in cerebrospinal fluid and blood from patients with a rare progressive central and peripheral demyelinating disease. JOURNAL OF THE NEUROLOGICAL SCIENCES. 2008;266(1-2):70-8.
1570.	Geloso MC, D'Ambrosi N. Microglial Pruning: Relevance for Synaptic Dysfunction in Multiple Sclerosis and Related Experimental Models. Cells. 2021;10(3).
1571.	Gemmati D, Zeri G, Orioli E, De Gaetano FE, Salvi F, Bartolomei I, et al. Polymorphisms in the genes coding for iron binding and transporting proteins are associated with disability, severity, and early progression in multiple sclerosis. BMC MEDICAL GENETICS. 2012;13.
1572.	Gemmati D, Zeri G, Orioli E, De Gaetano FE, Salvi F, Bartolomei I, et al. Polymorphisms in the genes coding for iron binding and transporting proteins are associated with disability, severity, and early progression in multiple sclerosis. BMC Medical Genetics. 2012.
1573.	Gemmati D, Zeri G, Orioli E, De Gaetano FE, Salvi F, Bartolomei I, et al. Polymorphisms in the genes coding for iron binding and transporting proteins are associated with disability, severity, and early progression in multiple sclerosis. BMC MEDICAL GENETICS. 2012;13.
1574.	Gemmati D, Zeri G, Orioli E, De Gaetano FE, Salvi F, Bartolomei I, et al. Polymorphisms in the genes coding for iron binding and transporting proteins are associated with disability, severity, and early progression in multiple sclerosis. BMC Med Genet. 2012;13:70.
1575.	Gemmati D, Zeri G, Orioli E, De Gaetano FE, Salvi F, Bartolomei I, et al. Polymorphisms in the genes coding for iron binding and transporting proteins are associated with disability, severity, and early progression in multiple sclerosis. BMC MEDICAL GENETICS. 2012;13.
1576.	Gemmati D, Zeri G, Orioli E, De Gaetano FE, Salvi F, Bartolomei I, et al. Iron Gene Polymorphisms are Associated to Disability, Severity and Early Progression in Multiple Sclerosis. BMC Med Gen. 2012.
1577.	Gemmati D, Zeri G, Orioli E, De Gaetano FE, Salvi F, Bartolomei I, et al. Polymorphisms in the genes coding for iron binding and transporting proteins are associated with disability, severity, and early progression in multiple sclerosis. BMC medical genetics. 2012;13(1):1-13.
1578.	Gerbeaux J, Labrune B, Tournier G. [Pediatrics in 1977]. Rev Prat. 1978;28(23):1703-25.
1579.	Gerrard B, Singh V, Babenko O, Gauthier I, Wee Yong V, Kovalchuk I, et al. Chronic mild stress exacerbates severity of experimental autoimmune encephalomyelitis in association with altered non-coding RNA and metabolic biomarkers. Neuroscience. 2017.
1580.	Gerrard B, Singh V, Babenko O, Gauthier I, Yong VW, Kovalchuk I, et al. CHRONIC MILD STRESS EXACERBATES SEVERITY OF EXPERIMENTAL AUTOIMMUNE ENCEPHALOMYELITIS IN ASSOCIATION WITH ALTERED NON-CODING RNA AND METABOLIC BIOMARKERS. NEUROSCIENCE. 2017;359:299-307.
1581.	Gerrard B, Singh V, Babenko O, Gauthier I, Yong VW, Kovalchuk I, et al. CHRONIC MILD STRESS EXACERBATES SEVERITY OF EXPERIMENTAL AUTOIMMUNE ENCEPHALOMYELITIS IN ASSOCIATION WITH ALTERED NON-CODING RNA AND METABOLIC BIOMARKERS. NEUROSCIENCE. 2017;359:299-307.
1582.	Gervasoni E, Beghi E, Corrini C, Parelli R, Bianchi E, Mestanza Mattos FG, et al. Validity of 2 Fall Prevention Strategy Scales for People With Stroke, Parkinson's Disease, and Multiple Sclerosis. J Geriatr Phys Ther. 2023;46(1):36-45.
1583.	Gevorkyan AA, Kotov SV, Lizhdvoy VY. [Robotic mechanotherapy in patients with multiple sclerosis with impaired walking function]. Zh Nevrol Psikhiatr Im S S Korsakova. 2020;120(7):29-34.
1584.	Ghajarzadeh M, Moghadasi AN, Navardi S, Mohammadi A, Hamtaee S, Sahraian MA, et al. Attitude and Knowledge of Patients with Multiple Sclerosis (MS) About COVID-19 as Provided by Physicians. ARCHIVES OF NEUROSCIENCE. 2021;8(1).
1585.	Ghajarzadeh M, Moghadasi AN, Navardi S, Mohammadi A, Hamtaee S, Sahraian MA, et al. Attitude and Knowledge of Patients with Multiple Sclerosis (MS) About COVID-19 as Provided by Physicians. ARCHIVES OF NEUROSCIENCE. 2021;8(1).
1586.	Gharagozli K, Shojaei M, Harandi AA, Akbari N, Ilkhani M. Myasthenia gravis development and crisis subsequent to multiple sclerosis. Case Rep Med. 2011;2011:291731.
1587.	Gharagozloo M, Mace JW, Calabresi PA. Animal models to investigate the effects of inflammation on remyelination in multiple sclerosis. Front Mol Neurosci. 2022;15:995477.
1588.	Ghassaban K, Liu S, Jiang C, Haacke EM. Quantifying iron content in magnetic resonance imaging. NeuroImage. 2019.
1589.	Ghassaban K, Liu S, Jiang C, Haacke EM. Quantifying iron content in magnetic resonance imaging. Neuroimage. 2019;187:77-92.
1590.	Ghassaban K, Liu SF, Jiang CH, Haacke EM. Quantifying iron content in magnetic resonance imaging. NEUROIMAGE. 2019;187:77-92.
1591.	Ghassaban K, Liu SF, Jiang CH, Haacke EM. Quantifying iron content in magnetic resonance imaging. NEUROIMAGE. 2019;187:77-92.
1592.	Ghazavi A, Kianbakht S, Ghasami K, Mosayebi G. High Copper and Low Zinc Serum Levels in Iranian Patients with Multiple Sclerosis: A Case Control Study. CLINICAL LABORATORY. 2012;58(1-2):161-4.
1593.	Ghazavi A, Kianbakht S, Ghasami K, Mosayebi G. High copper and low zinc serum levels in iranian patients with multiple sclerosis: A case control study. Clinical Laboratory. 2012.
1594.	Ghazavi A, Kianbakht S, Ghasami K, Mosayebi G. High Copper and Low Zinc Serum Levels in Iranian Patients with Multiple Sclerosis: A Case Control Study. CLINICAL LABORATORY. 2012;58(1-2):161-4.
1595.	Ghazavi A, Kianbakht S, Ghasami K, Mosayebi G. High copper and low zinc serum levels in Iranian patients with multiple sclerosis: a case control study. Clin Lab. 2012;58(1-2):161-4.
1596.	Ghazavi A, Kianbakht S, Ghasami K, Mosayebi G. High Copper and Low Zinc Serum Levels in Iranian Patients with Multiple Sclerosis: A Case Control Study. CLINICAL LABORATORY. 2012;58(1-2):161-4.
1597.	Ghazavi A, Kianbakht S, Ghasami K, Mosayebi G. High copper and low zinc serum levels in Iranian patients with multiple sclerosis: a case control study. Clin Lab. 2012;58(1-2):161-4.
1598.	Ghezzi A, Comi G, Federico A. Chronic cerebro-spinal venous insufficiency (CCSVI) and multiple sclerosis. NEUROLOGICAL SCIENCES. 2011;32(1):17-21.
1599.	Ghezzi A, Comi G, Federico A. Chronic cerebro-spinal venous insufficiency (CCSVI) and multiple sclerosis. Neurological Sciences. 2011.
1600.	Ghezzi A, Comi G, Federico A. Chronic cerebro-spinal venous insufficiency (CCSVI) and multiple sclerosis. Neurol Sci. 2011;32(1):17-21.
1601.	Ghobadinezhad F, Ebrahimi N, Mozaffari F, Moradi N, Beiranvand S, Pournazari M, et al. The emerging role of regulatory cell-based therapy in autoimmune disease. Front Immunol. 2022;13:1075813.
1602.	Gholizadeh S, Haghaei H, Karami H, Soltani S, Zakariazadeh M, Shokri J. Mode of binding, kinetic and thermodynamic properties of a lipid-like drug (Fingolimod) interacting with Human Serum Albumin. Bioimpacts. 2023;13(2):109-21.
1603.	Ghoreishi A, Mohseni M, Amraei R, Alizadeh A, Mazloomzadeh S. Investigation the amount of copper, lead, zinc and cadmium levels in serum of Iranian multiple sclerosis patients. Journal of Chemical and Pharmaceutical Sciences. 2015;8:40-5.
1604.	Ghoreishi A, Mohseni M, Amraei R, Alizadeh AM, Mazloomzadeh S. Investigation the amount of copper, lead, zinc and cadmium levels in serum of Iranian multiple sclerosis patients. Journal of Chemical and Pharmaceutical Sciences. 2015.
1605.	Giacoppo S, Galuppo M, Calabro RS, D'Aleo G, Marra A, Sessa E, et al. Heavy Metals and Neurodegenerative Diseases: An Observational Study. BIOLOGICAL TRACE ELEMENT RESEARCH. 2014;161(2):151-60.
1606.	Giacoppo S, Galuppo M, Calabrò RS, D'Aleo G, Marra A, Sessa E, et al. Heavy metals and neurodegenerative diseases: an observational study. Biol Trace Elem Res. 2014;161(2):151-60.
1607.	Giacoppo S, Galuppo M, Calabro RS, D'Aleo G, Marra A, Sessa E, et al. Heavy Metals and Neurodegenerative Diseases: An Observational Study. BIOLOGICAL TRACE ELEMENT RESEARCH. 2014;161(2):151-60.
1608.	Giacoppo S, Galuppo M, Calabrò RS, D’Aleo G, Marra A, Sessa E, et al. Heavy Metals and Neurodegenerative Diseases: An Observational Study. Biological Trace Element Research. 2014.
1609.	Gibson EG, Pender M, Angerbauer M, Cook C, Jones B, Spivak AM, et al. Prolonged SARS-CoV-2 Illness in a Patient Receiving Ocrelizumab for Multiple Sclerosis. Open Forum Infect Dis. 2021;8(7):ofab176.
1610.	Gierer S, Kannamueller E, Maier V, Gierer S, editors. Fatigue and cognitive impairment in multiple sclerosis patients lead to impaired performance in computer-assisted test for driving fitness2013: SAGE PUBLICATIONS LTD 1 OLIVERS YARD, 55 CITY ROAD, LONDON EC1Y 1SP, ENGLAND.
1611.	Gierer S, Kannamuller E, Maier V, Gierer S. Fatigue and cognitive impairment in multiple sclerosis patients lead to impaired performance in computer-assisted test for driving fitness. MULTIPLE SCLEROSIS JOURNAL. 2013;19(11):200-.
1612.	Gigli GL, Serafini A, Lorenzut S, Valente M. Restless Legs Syndrome in Internal Medicine.  Encyclopedia of Sleep2013.
1613.	Gil-González I, Pérez-San-Gregorio M, Conrad R, Martín-Rodríguez A. Beyond the Boundaries of Disease-Significant Post-traumatic Growth in Multiple Sclerosis Patients and Caregivers. Front Psychol. 2022;13:903508.
1614.	Gilland O. [American neurology for Swedish use]. Lakartidningen. 1968;65(15):1539-46.
1615.	Gillen KM, Mubarak M, Nguyen TD, Pitt D. Significance and in vivo detection of iron-laden microglia in white matter multiple sclerosis lesions. Front Immunol. 2018; 9: 255.
1616.	Gillen KM, Mubarak M, Nguyen TD, Pitt D. Significance and In Vivo Detection of Iron-Laden Microglia in White Matter Multiple Sclerosis Lesions. FRONTIERS IN IMMUNOLOGY. 2018;9.
1617.	Gillen KM, Mubarak M, Nguyen TD, Pitt D. Significance and in vivo detection of iron-laden microglia in white matter multiple sclerosis lesions. Frontiers in Immunology. 2018.
1618.	Gillen KM, Mubarak M, Nguyen TD, Pitt D. Significance and In Vivo Detection of Iron-Laden Microglia in White Matter Multiple Sclerosis Lesions. FRONTIERS IN IMMUNOLOGY. 2018;9.
1619.	Gillen KM, Mubarak M, Nguyen TD, Pitt D. Significance and In Vivo Detection of Iron-Laden Microglia in White Matter Multiple Sclerosis Lesions. Front Immunol. 2018;9:255.
1620.	Gillen KM, Mubarak M, Nguyen TD, Pitt D. Significance and In Vivo Detection of Iron-Laden Microglia in White Matter Multiple Sclerosis Lesions. FRONTIERS IN IMMUNOLOGY. 2018;9.
1621.	Gillen KM, Mubarak M, Nguyen TD, Pitt D. Significance and in vivo detection of iron-laden microglia in white matter multiple sclerosis lesions. Frontiers in immunology. 2018;9:255.
1622.	Gillen KM, Mubarak M, Park C, Ponath G, Zhang S, Dimov A, et al. QSM is an imaging biomarker for chronic glial activation in multiple sclerosis lesions. Annals of Clinical and Translational Neurology. 2021.
1623.	Gillen KM, Mubarak M, Park C, Ponath G, Zhang S, Dimov A, et al. QSM is an imaging biomarker for chronic glial activation in multiple sclerosis lesions. Ann Clin Transl Neurol. 2021;8(4):877-86.
1624.	Gilliam DT, Menon V, Bretz NP, Pruszak J. The CD24 surface antigen in neural development and disease. Neurobiol Dis. 2017;99:133-44.
1625.	Gilmore RL, Kasarskis EJ, Carr WA, Norvell E. Comparative impact of paraclinical studies in establishing the diagnosis of multiple sclerosis. Electroencephalogr Clin Neurophysiol. 1989;73(5):433-42.
1626.	Gilon C, Klazas M, Lahiani A, Schumacher-Klinger A, Merzbach S, Naoum JN, et al. Synthesis and Pharmacological Characterization of Visabron, a Backbone Cyclic Peptide Dual Antagonist of α4β1 (VLA-4)/α9β1 Integrin for Therapy of Multiple Sclerosis. JACS Au. 2021;1(12):2361-76.
1627.	Giménez-Orenga K, Pierquin J, Brunel J, Charvet B, Martín-Martínez E, Perron H, et al. HERV-W ENV antigenemia and correlation of increased anti-SARS-CoV-2 immunoglobulin levels with post-COVID-19 symptoms. Front Immunol. 2022;13:1020064.
1628.	Giordano A, Lugaresi A, Confalonieri P, Granella F, Radice D, Trojano M, et al. Implementation of the 'Sapere Migliora' information aid for newly diagnosed people with multiple sclerosis in routine clinical practice: a late-phase controlled trial. Mult Scler. 2014;20(9):1234-43.
1629.	Giordano A, Santoro S, Sorosina M, Clarelli F, Ferre L, Cannizzaro M, et al. A gene-set analysis suggests the possible involvement of iron homeostasis in neurodegeneration in progressive multiple sclerosis. JOURNAL OF THE NEUROLOGICAL SCIENCES. 2021;429.
1630.	Giordano A, Santoro S, Sorosina M, Clarelli F, Ferrè L, Cannizzaro M, et al. A gene-set analysis suggests the possible involvement of iron homeostasis in neurodegeneration in progressive multiple sclerosis. Journal of the Neurological Sciences. 2021;429.
1631.	Giordano A, Santoro S, Sorosina M, Mascia E, Clarelli F, Cannizzaro M, et al. A gene-set analysis suggests the possible involvement of iron homeostasis in neurodegeneration in progressive multiple sclerosis. MULTIPLE SCLEROSIS JOURNAL. 2021;27(2_SUPPL):50-.
1632.	Giordo R, Wehbe Z, Posadino AM, Erre GL, Eid AH, Mangoni AA, et al. Disease-Associated Regulation of Non-Coding RNAs by Resveratrol: Molecular Insights and Therapeutic Applications. Front Cell Dev Biol. 2022;10:894305.
1633.	Giorgio A, De Stefano N. Advanced Structural and Functional Brain MRI in Multiple Sclerosis. SEMINARS IN NEUROLOGY. 2016;36(2):163-76.
1634.	Giovannetti AM, Barabasch A, Giordano A, Quintas R, Alfieri S, Schiffman I, et al. Consensus on an user-led resource for people transitioning to secondary progressive multiple sclerosis - results of an international multi-stakeholder nominal group technique study. MULTIPLE SCLEROSIS JOURNAL. 2019;25(7):1037-.
1635.	Giovannetti AM, Barabasch A, Giordano A, Quintas R, Barello S, Graffigna G, et al. Construction of a User-Led Resource for People Transitioning to Secondary Progressive Multiple Sclerosis: Results of an International Nominal Group Study. FRONTIERS IN NEUROLOGY. 2020;11.
1636.	Giovannetti AM, Barabasch A, Giordano A, Quintas R, Barello S, Graffigna G, et al. Construction of a User-Led Resource for People Transitioning to Secondary Progressive Multiple Sclerosis: Results of an International Nominal Group Study. FRONTIERS IN NEUROLOGY. 2020;11.
1637.	Giovannetti AM, Barabasch A, Giordano A, Quintas R, Barello S, Graffigna G, et al. Construction of a User-Led Resource for People Transitioning to Secondary Progressive Multiple Sclerosis: Results of an International Nominal Group Study. FRONTIERS IN NEUROLOGY. 2020;11.
1638.	Giussani P, Prinetti A, Tringali C. The role of Sphingolipids in myelination and myelin stability and their involvement in childhood and adult demyelinating disorders. J Neurochem. 2021;156(4):403-14.
1639.	Giustiniano M, Tortorella P, Agamennone M, Di Pizio A, Rossello A, Nuti E, et al. Amino Acid derivatives as new zinc binding groups for the design of selective matrix metalloproteinase inhibitors. J Amino Acids. 2013;2013:178381.
1640.	Gize RW, Mishkin FS. Brain scans in multiple sclerosis. Radiology. 1970;97(2):297-9.
1641.	Gkagkanasiou M, Ploussi A, Gazouli M, Efstathopoulos EP. USPIO-Enhanced MRI Neuroimaging. JOURNAL OF NEUROIMAGING. 2016;26(2):161-8.
1642.	Gkagkanasiou M, Ploussi A, Gazouli M, Efstathopoulos EP. USPIO-Enhanced MRI Neuroimaging: A Review. Journal of Neuroimaging. 2016.
1643.	Gkagkanasiou M, Ploussi A, Gazouli M, Efstathopoulos EP. USPIO-Enhanced MRI Neuroimaging: A Review. J Neuroimaging. 2016;26(2):161-8.
1644.	Glenn JD, Smith MD, Xue P, Chan-Li Y, Collins S, Calabresi PA, et al. CNS-targeted autoimmunity leads to increased influenza mortality in mice. JOURNAL OF EXPERIMENTAL MEDICINE. 2017;214(2):297-307.
1645.	Glenn JD, Smith MD, Xue P, Chan-Li Y, Collins S, Calabresi PA, et al. CNS-targeted autoimmunity leads to increased influenza mortality in mice. JOURNAL OF EXPERIMENTAL MEDICINE. 2017;214(2):297-307.
1646.	Glinsky GV. An SNP-guided microRNA map of fifteen common human disorders identifies a consensus disease phenocode aiming at principal components of the nuclear import pathway. Cell Cycle. 2008;7(16):2570-83.
1647.	Goderie T, van Wier MF, Lissenberg-Witte BI, Merkus P, Smits C, Leemans CR, et al. Factors Associated With the Development of Tinnitus and With the Degree of Annoyance Caused by Newly Developed Tinnitus. Ear Hear. 2022;43(6):1807-15.
1648.	Goh YX, Jalil J, Lam KW, Husain K, Premakumar CM. Genistein: A Review on its Anti-Inflammatory Properties. Front Pharmacol. 2022;13:820969.
1649.	Gokuladhas S, Schierding W, Cameron-Smith D, Wake M, Scotter EL, O'Sullivan J. Shared Regulatory Pathways Reveal Novel Genetic Correlations Between Grip Strength and Neuromuscular Disorders. Front Genet. 2020;11:393.
1650.	Gola L, Bierhansl L, Hummel N, Korn L, Pawlowski M, Cerina M, et al. MMF induces antioxidative and anaplerotic pathways and is neuroprotective in hyperexcitability in vitro. Free Radic Biol Med. 2023;194:337-46.
1651.	Goldberg P, Fleming MC, Picard EH. Multiple sclerosis: Decreased relapse rate through dietary supplementation with calcium, magnesium and vitamin D. Medical Hypotheses. 1986.
1652.	Goldberg P, Fleming MC, Picard EH. MULTIPLE-SCLEROSIS - DECREASED RELAPSE RATE THROUGH DIETARY SUPPLEMENTATION WITH CALCIUM, MAGNESIUM AND VITAMIN-D. MEDICAL HYPOTHESES. 1986;21(2):193-200.
1653.	Goldberg P, Fleming MC, Picard EH. Multiple sclerosis: decreased relapse rate through dietary supplementation with calcium, magnesium and vitamin D. Med Hypotheses. 1986;21(2):193-200.
1654.	Goldberg P, Fleming MC, Picard EH. Multiple sclerosis: decreased relapse rate through dietary supplementation with calcium, magnesium and vitamin D. Medical hypotheses. 1986;21(2):193-200.
1655.	Goldschmidt C, McGinley MP. Advances in the Treatment of Multiple Sclerosis. Neurol Clin. 2021;39(1):21-33.
1656.	Golmohammadi M, Kheirouri S, Ebrahimzadeh Attari V, Moludi J, Sulistyowati R, Nachvak SM, et al. Is there any association between dietary inflammatory index and quality of life? A systematic review. Front Nutr. 2022;9:1067468.
1657.	Golpour F, Abbasi-Alaei M, Babaei F, Mirzababaei M, Parvardeh S, Mohammadi G, et al. Short chain fatty acids, a possible treatment option for autoimmune diseases. Biomed Pharmacother. 2023;163:114763.
1658.	Gomes A, Adoni T. Differential diagnosis of demyelinating diseases: what's new? Arq Neuropsiquiatr. 2022;80(5 Suppl 1):137-42.
1659.	Gomes A, Feo LB, Silva GD, Disserol CCD, Paolilo RB, Lara AN, et al. Reducing infection risk in multiple sclerosis and neuromyelitis optica spectrum disorders: a Brazilian reference center's approach. Arq Neuropsiquiatr. 2022;80(10):1057-66.
1660.	Gomes AC, Jonsson G, Mjornheim S, Olsson T, Hillert J, Grandien A. Upregulation of the apoptosis regulators cFLIP, CD95 and CD95 ligand in peripheral blood mononuclear cells in relapsing-remitting multiple sclerosis. JOURNAL OF NEUROIMMUNOLOGY. 2003;135(1-2):126-34.
1661.	Gomes AC, Jönsson G, Mjörnheim S, Olsson T, Hillert J, Grandien A. Upregulation of the apoptosis regulators cFLIP, CD95 and CD95 ligand in peripheral blood mononuclear cells in relapsing-remitting multiple sclerosis. J Neuroimmunol. 2003;135(1-2):126-34.
1662.	Gomes AC, Jonsson G, Mjornheim S, Olsson T, Hillert J, Grandien A. Upregulation of the apoptosis regulators cFLIP, CD95 and CD95 ligand in peripheral blood mononuclear cells in relapsing-remitting multiple sclerosis. JOURNAL OF NEUROIMMUNOLOGY. 2003;135(1-2):126-34.
1663.	Gontika M, Skarlis C, Markoglou N, Evangelopoulos ME, Velonakis G, Chrousos GP, et al. Fingolimod as a first- or second-line treatment in a mini-series of young Hellenic patients with adolescent-onset multiple sclerosis: focus on immunological data. Neurol Sci. 2022;43(4):2641-9.
1664.	González LM, Ospina LN, Sperling LE, Chaparro O, Cucarián JD. Therapeutic Effects of Physical Exercise and the Mesenchymal Stem Cell Secretome by Modulating Neuroinflammatory Response in Multiple Sclerosis. Curr Stem Cell Res Ther. 2022;17(7):621-32.
1665.	Gori F, Mulinacci B, Massai L, Avolio C, Caragnano M, Peroni E, et al. IgG and IgM antibodies to the refolded MOG(1-125) extracellular domain in humans. J Neuroimmunol. 2011;233(1-2):216-20.
1666.	Górska A, Markiewicz-Gospodarek A, Markiewicz R, Chilimoniuk Z, Borowski B, Trubalski M, et al. Distribution of Iron, Copper, Zinc and Cadmium in Glia, Their Influence on Glial Cells and Relationship with Neurodegenerative Diseases. Brain Sci. 2023;13(6).
1667.	Gotkine M, Fellig Y, Abramsky O. Occurrence of CNS demyelinating disease in patients with myasthenia gravis. Neurology. 2006;67(5):881-3.
1668.	Gould CE, Warren HV. Trace elements in human biology and a preliminary report on a possible relationship to multiple sclerosis. Science of the Total Environment. 1980.
1669.	Gould CE, Warren HV. TRACE-ELEMENTS IN HUMAN-BIOLOGY AND A PRELIMINARY-REPORT ON A POSSIBLE RELATIONSHIP TO MULTIPLE-SCLEROSIS. SCIENCE OF THE TOTAL ENVIRONMENT. 1980;15(3):261-8.
1670.	Gould CE, Warren HV. Trace elements in human biology and a preliminary report on a possible relationship to multiple sclerosis. Science of the Total Environment. 1980;15(3):261-8.
1671.	Graber JJ, Allie SR, Mullen KM, Jones MV, Wang D, Krishnan C, et al. Elevated T-cell production of IL-17 in transverse myelitis and multiple sclerosis leads to induction of astrocyte IL-6. NEUROLOGY. 2006;66(5):311-.
1672.	Grabner G, Dal-Bianco A, Schernthaner M, Vass K, Lassmann H, Trattnig S. Analysis of Multiple Sclerosis Lesions Using a Fusion of 3.0 T FLAIR and 7.0 T SWI Phase: FLAIR SWI. JOURNAL OF MAGNETIC RESONANCE IMAGING. 2011;33(3):543-9.
1673.	Grabner G, Dal-Bianco A, Schernthaner M, Vass K, Lassmann H, Trattnig S. Analysis of multiple sclerosis lesions using a fusion of 3.0 T FLAIR and 7.0 T SWI phase: FLAIR SWI. Journal of Magnetic Resonance Imaging. 2011.
1674.	Grabner G, Dal-Bianco A, Schernthaner M, Vass K, Lassmann H, Trattnig S. Analysis of multiple sclerosis lesions using a fusion of 3.0 T FLAIR and 7.0 T SWI phase: FLAIR SWI. J Magn Reson Imaging. 2011;33(3):543-9.
1675.	Grabner G, Dal-Bianco A, Schernthaner M, Vass K, Lassmann H, Trattnig S. Analysis of Multiple Sclerosis Lesions Using a Fusion of 3.0 T FLAIR and 7.0 T SWI Phase: FLAIR SWI. JOURNAL OF MAGNETIC RESONANCE IMAGING. 2011;33(3):543-9.
1676.	Grabrucker AM, Rowan M, Garner CC. Brain-Delivery of Zinc-Ions as Potential Treatment for Neurological Diseases: Mini Review. Drug Deliv Lett. 2011;1(1):13-23.
1677.	Gracien RM, Reitz SC, Hof SM, Fleischer V, Zimmermann H, Droby A, et al. Assessment of cortical damage in early multiple sclerosis with quantitative T-2 relaxometry. NMR IN BIOMEDICINE. 2016;29(4):444-50.
1678.	Gracien RM, Reitz SC, Hof SM, Fleischer V, Zimmermann H, Droby A, et al. Assessment of cortical damage in early multiple sclerosis with quantitative T2 relaxometry. NMR in Biomedicine. 2016.
1679.	Gracien RM, Reitz SC, Hof SM, Fleischer V, Zimmermann H, Droby A, et al. Assessment of cortical damage in early multiple sclerosis with quantitative T2 relaxometry. NMR Biomed. 2016;29(4):444-50.
1680.	Gracien RM, Reitz SC, Hof SM, Fleischer V, Zimmermann H, Droby A, et al. Assessment of cortical damage in early multiple sclerosis with quantitative T-2 relaxometry. NMR IN BIOMEDICINE. 2016;29(4):444-50.
1681.	Grahmann H. [The so-called paralysis curve of the goldsol reaction in nonsyphiliic neuropathies]. Nervenarzt. 1969;40(2):92-5.
1682.	Grant SM, Wiesinger JA, Beard JL, Cantorna MT. Iron-deficient mice fail to develop autoimmune encephalomyelitis. JOURNAL OF NUTRITION. 2003;133(8):2635-8.
1683.	Grant SM, Wiesinger JA, Beard JL, Cantorna MT. Iron-deficient mice fail to develop autoimmune encephalomyelitis. Journal of Nutrition. 2003.
1684.	Grant SM, Wiesinger JA, Beard JL, Cantorna MT. Iron-deficient mice fail to develop autoimmune encephalomyelitis. J Nutr. 2003;133(8):2635-8.
1685.	Grant SM, Wiesinger JA, Beard JL, Cantorna MT. Iron-deficient mice fail to develop autoimmune encephalomyelitis. JOURNAL OF NUTRITION. 2003;133(8):2635-8.
1686.	Gratton SM, Herro AM, Feuer WJ, Lam BL. Cigarette Smoking and Activities of Daily Living in Ocular Myasthenia Gravis. J Neuroophthalmol. 2016;36(1):37-40.
1687.	Grauffel C, Weng WH, Dudev T, Lim C. Trinuclear Calcium Site in the C2 Domain of PKCα/γ Is Prone to Lithium Attack. ACS Omega. 2021;6(31):20657-66.
1688.	Gray KJ, Gibbs JE. Adaptive immunity, chronic inflammation and the clock. Semin Immunopathol. 2022;44(2):209-24.
1689.	Greenham LW, Peacock DB. GEOGRAPHICAL DISTRIBUTION OF MULTIPLE SCLEROSIS. The Lancet. 1975.
1690.	Greenham LW, Peacock DB. Letter: Geographical distribution of multiple sclerosis. Lancet. 1975;1(7898):106.
1691.	Grimaldi-Bensouda L, Papeix C, Hamon Y, Benichou J, Abenhaim L. Association between vaccination and the risk of central demyelination: results from a case-referent study. J Neurol. 2023;270(10):4678-86.
1692.	Grimaldi-Bensouda L, Rossignol M, Koné-Paut I, Krivitzky A, Lebrun-Frenay C, Clet J, et al. Risk of autoimmune diseases and human papilloma virus (HPV) vaccines: Six years of case-referent surveillance. J Autoimmun. 2017;79:84-90.
1693.	Grimaud J, Millar J, Thorpe JW, Moseley IF, McDonald WI, Miller DH. SIGNAL INTENSITY ON MRI OF BASAL GANGLIA IN MULTIPLE-SCLEROSIS. JOURNAL OF NEUROLOGY NEUROSURGERY AND PSYCHIATRY. 1995;59(3):306-8.
1694.	Grimaud J, Millar J, Thorpe JW, Moseley IF, McDonald WI, Miller DH. Signal intensity on MRI of basal ganglia in multiple sclerosis. Journal of Neurology, Neurosurgery and Psychiatry. 1995.
1695.	Grimaud J, Millar J, Thorpe JW, Moseley IF, McDonald WI, Miller DH. Signal intensity on MRI of basal ganglia in multiple sclerosis. J Neurol Neurosurg Psychiatry. 1995;59(3):306-8.
1696.	Gruchot J, Charvet B, Silva M, Dietrich M, Albrecht P, Perron H, et al. Endogenous expression of HERV-W envelope protein leads to impaired glial functions in a mouse model of multiple sclerosis. GLIA. 2021;69:E179-E.
1697.	Grzesiuk AK. Epidemiological profile in multiple sclerosis patients, Uberaba, MG, Brazil. Arq Neuropsiquiatr. 2011;69(5):852.
1698.	Guan D, Su Y, Li Y, Wu C, Meng Y, Peng X, et al. Tetramethylpyrazine inhibits CoCl2 -induced neurotoxicity through enhancement of Nrf2/GCLc/GSH and suppression of HIF1α/NOX2/ROS pathways. J Neurochem. 2015;134(3):551-65.
1699.	Guardiani C, Marsili S, Procacci P, Livi R. Fragment 101-108 of Myelin Oligodendrocyte Glycoprotein: A Possible Lead Compound for Multiple Sclerosis. JOURNAL OF THE AMERICAN CHEMICAL SOCIETY. 2009;131(47):17176-84.
1700.	Guardiani C, Marsili S, Procacci P, Livi R. Fragment 101-108 of Myelin Oligodendrocyte Glycoprotein: A Possible Lead Compound for Multiple Sclerosis. JOURNAL OF THE AMERICAN CHEMICAL SOCIETY. 2009;131(47):17176-84.
1701.	Guardiani C, Marsili S, Procacci P, Livi R. Fragment 101-108 of Myelin Oligodendrocyte Glycoprotein: A Possible Lead Compound for Multiple Sclerosis. JOURNAL OF THE AMERICAN CHEMICAL SOCIETY. 2009;131(47):17176-84.
1702.	Guardiani C, Marsili S, Procacci P, Livi R. Fragment 101− 108 of Myelin Oligodendrocyte Glycoprotein: A Possible Lead Compound for Multiple Sclerosis. Journal of the American Chemical Society. 2009;131(47):17176-84.
1703.	Guardiani C, Procacci P. The conformational landscape of tartrate-based inhibitors of the TACE enzyme as revealed by Hamiltonian Replica Exchange simulation. PHYSICAL CHEMISTRY CHEMICAL PHYSICS. 2013;15(23):9186-96.
1704.	Guardiani C, Procacci P. The conformational landscape of tartrate-based inhibitors of the TACE enzyme as revealed by Hamiltonian Replica Exchange simulation. Physical Chemistry Chemical Physics. 2013.
1705.	Guardiani C, Procacci P. The conformational landscape of tartrate-based inhibitors of the TACE enzyme as revealed by Hamiltonian Replica Exchange simulation. Phys Chem Chem Phys. 2013;15(23):9186-96.
1706.	Guardiani C, Procacci P. The conformational landscape of tartrate-based inhibitors of the TACE enzyme as revealed by Hamiltonian Replica Exchange simulation. PHYSICAL CHEMISTRY CHEMICAL PHYSICS. 2013;15(23):9186-96.
1707.	Guarnaccia JB, Khan A, Ayettey R, Treu JA, Comerford B, Njike VY. Patterns of Medical Cannabis Use among Patients Diagnosed with Multiple Sclerosis. Mult Scler Relat Disord. 2021;50:102830.
1708.	Gubič Š, Hendrickx LA, Toplak Ž, Sterle M, Peigneur S, Tomašič T, et al. Discovery of K(V) 1.3 ion channel inhibitors: Medicinal chemistry approaches and challenges. Med Res Rev. 2021;41(4):2423-73.
1709.	Gudden J, Arias Vasquez A, Bloemendaal M. The Effects of Intermittent Fasting on Brain and Cognitive Function. Nutrients. 2021;13(9).
1710.	Gudesblatt M, Roman C, Singer BA, Schmidt H, Thomas J, Shankar SL, et al. Health-Related Quality of Life with Diroximel Fumarate in Patients with Relapsing Forms of Multiple Sclerosis: Findings from Qualitative Research Using Patient Interviews. Adv Ther. 2022;39(7):3199-213.
1711.	Guerini FR, Cagliani R, Forni D, Agliardi C, Caputo D, Cassinotti A, et al. A functional variant in ERAP1 predisposes to multiple sclerosis. PLoS One. 2012;7(1):e29931.
1712.	Guerra G, Ravaglia V, Pozzi R, Cenni P, Scrittori N. Characterizing iron deposition in healthy and multiple sclerosis patients using susceptibility weighted imaging in MR. Physica Medica: European Journal of Medical Physics. 2021;92:S218-S9.
1713.	Guerrero S, Sánchez-Tirado E, Agüí L, González-Cortés A, Yáñez-Sedeño P, Pingarrón JM. Development of an Electrochemical CCL5 Chemokine Immunoplatform for Rapid Diagnosis of Multiple Sclerosis. Biosensors (Basel). 2022;12(8).
1714.	Guery D, Marignier R, Durand-Dubief F, Lavie C, Pique J, Guerrier O, et al. Clinical failure of natalizumab in multiple sclerosis: Specific causes and strategy. Rev Neurol (Paris). 2021;177(10):1241-9.
1715.	Guglielmetti C, Praet J, Rangarajan JR, Vreys R, De Vocht N, Maes F, et al. Multimodal imaging of subventricular zone neural stem/progenitor cells in the cuprizone mouse model reveals increased neurogenic potential for the olfactory bulb pathway, but no contribution to remyelination of the corpus callosum. NEUROIMAGE. 2014;86:99-110.
1716.	Guglielmetti C, Praet J, Rangarajan JR, Vreys R, De Vocht N, Maes F, et al. Multimodal imaging of subventricular zone neural stem/progenitor cells in the cuprizone mouse model reveals increased neurogenic potential for the olfactory bulb pathway, but no contribution to remyelination of the corpus callosum. NeuroImage. 2014.
1717.	Guglielmetti C, Praet J, Rangarajan JR, Vreys R, De Vocht N, Maes F, et al. Multimodal imaging of subventricular zone neural stem/progenitor cells in the cuprizone mouse model reveals increased neurogenic potential for the olfactory bulb pathway, but no contribution to remyelination of the corpus callosum. Neuroimage. 2014;86:99-110.
1718.	Guglielmetti C, Praet J, Rangarajan JR, Vreys R, De Vocht N, Maes F, et al. Multimodal imaging of subventricular zone neural stem/progenitor cells in the cuprizone mouse model reveals increased neurogenic potential for the olfactory bulb pathway, but no contribution to remyelination of the corpus callosum. NEUROIMAGE. 2014;86:99-110.
1719.	Guinebretiere O, Nedelec T, Gantzer L, Lekens B, Durrleman S, Louapre C. Association Between Diseases and Symptoms Diagnosed in Primary Care and the Subsequent Specific Risk of Multiple Sclerosis. Neurology. 2023;101(24):e2497-e508.
1720.	Günal SY, Saçmacı H, Saçmacı Ş, Mirza M, İpekten F. Effect of heavy metals and sialic acid in multiple sclerosis. International Research Journal of Public and Environmental Health. 2018.
1721.	Guo CC, Chiesa PA, de Moor C, Fazeli MS, Schofield T, Hofer K, et al. Digital Devices for Assessing Motor Functions in Mobility-Impaired and Healthy Populations: Systematic Literature Review. J Med Internet Res. 2022;24(11):e37683.
1722.	Guo Z, Long L, Qiu W, Lu T, Zhang L, Shu Y, et al. The Distributional Characteristics of Multiple Sclerosis Lesions on Quantitative Susceptibility Mapping and Their Correlation With Clinical Severity. Frontiers in Neurology. 2021.
1723.	Guo Z, Long L, Qiu W, Lu T, Zhang L, Shu Y, et al. The Distributional Characteristics of Multiple Sclerosis Lesions on Quantitative Susceptibility Mapping and Their Correlation With Clinical Severity. Front Neurol. 2021;12:647519.
1724.	Guo ZX, Long L, Qiu W, Lu TT, Zhang LA, Shu YQ, et al. The Distributional Characteristics of Multiple Sclerosis Lesions on Quantitative Susceptibility Mapping and Their Correlation With Clinical Severity. FRONTIERS IN NEUROLOGY. 2021;12.
1725.	Guo ZX, Long L, Qiu W, Lu TT, Zhang LA, Shu YQ, et al. The Distributional Characteristics of Multiple Sclerosis Lesions on Quantitative Susceptibility Mapping and Their Correlation With Clinical Severity. FRONTIERS IN NEUROLOGY. 2021;12.
1726.	Gupta A, Lacoste B, Pistel PJ, Ingram DK, Hamel E, Alaoui-Jamali MA, et al. Neurotherapeutic effects of novel HO-1 inhibitors in vitro and in a transgenic mouse model of Alzheimer's disease. JOURNAL OF NEUROCHEMISTRY. 2014;131(6):778-90.
1727.	Gupta A, Lacoste B, Pistel PJ, Ingram DK, Hamel E, Alaoui-Jamali MA, et al. Neurotherapeutic effects of novel HO-1 inhibitors in vitro and in a transgenic mouse model of Alzheimer's disease. Journal of Neurochemistry. 2014.
1728.	Gupta A, Lacoste B, Pistel PJ, Ingram DK, Hamel E, Alaoui-Jamali MA, et al. Neurotherapeutic effects of novel HO-1 inhibitors in vitro and in a transgenic mouse model of Alzheimer's disease. JOURNAL OF NEUROCHEMISTRY. 2014;131(6):778-90.
1729.	Gupta A, Lacoste B, Pistell PJ, Ingram DK, Hamel E, Alaoui-Jamali MA, et al. Neurotherapeutic effects of novel HO-1 inhibitors in vitro and in a transgenic mouse model of Alzheimer's disease. J Neurochem. 2014;131(6):778-90.
1730.	Gurr MI. The role of lipids in the regulation of the immune system. Prog Lipid Res. 1983;22(4):257-87.
1731.	Gusev A, Bhatia G, Zaitlen N, Vilhjalmsson BJ, Diogo D, Stahl EA, et al. Quantifying missing heritability at known GWAS loci. PLoS Genet. 2013;9(12):e1003993.
1732.	Gusev EI, Martynov MY, Boyko AN, Voznyuk IA, Latsh NY, Sivertseva SA, et al. The Novel Coronavirus Infection (COVID-19) and Nervous System Involvement: Mechanisms of Neurological Disorders, Clinical Manifestations, and the Organization of Neurological Care. Neurosci Behav Physiol. 2021;51(2):147-54.
1733.	Haacke EM. Imaging Iron in MS using Susceptibility Weighted Imaging (SWI):“Is the basic etiology of multiple sclerosis vascular in origin?”.
1734.	Haacke EM, Ayaz M, Khan A, Manova ES, Krishnamurthy B, Gollapalli L, et al. Establishing a baseline phase behavior in magnetic resonance imaging to determine normal vs. abnormal iron content in the brain. JOURNAL OF MAGNETIC RESONANCE IMAGING. 2007;26(2):256-64.
1735.	Haacke EM, Ayaz M, Khan A, Manova ES, Krishnamurthy B, Gollapalli L, et al. Establishing a baseline phase behavior in magnetic resonance imaging to determine normal vs. abnormal iron content in the brain. Journal of Magnetic Resonance Imaging. 2007.
1736.	Haacke EM, Ayaz M, Khan A, Manova ES, Krishnamurthy B, Gollapalli L, et al. Establishing a baseline phase behavior in magnetic resonance imaging to determine normal vs. abnormal iron content in the brain. J Magn Reson Imaging. 2007;26(2):256-64.
1737.	Haacke EM, Ayaz M, Khan A, Manova ES, Krishnamurthy B, Gollapalli L, et al. Establishing a baseline phase behavior in magnetic resonance imaging to determine normal vs. abnormal iron content in the brain. JOURNAL OF MAGNETIC RESONANCE IMAGING. 2007;26(2):256-64.
1738.	Haacke EM, Beggs CB, Habib C. The Role of Venous Abnormalities in Neurological Disease. REVIEWS ON RECENT CLINICAL TRIALS. 2012;7(2):100-16.
1739.	Haacke EM, Beggs CB, Habib C. The role of venous abnormalities in neurological disease. Reviews on Recent Clinical Trials. 2012.
1740.	Haacke EM, Beggs CB, Habib C. The role of venous abnormalities in neurological disease. Rev Recent Clin Trials. 2012;7(2):100-16.
1741.	Haacke EM, Beggs CB, Habib C. The Role of Venous Abnormalities in Neurological Disease. REVIEWS ON RECENT CLINICAL TRIALS. 2012;7(2):100-16.
1742.	Haacke EM, Cheng NY, House MJ, Liu Q, Neelavalli J, Ogg RJ, et al. Imaging iron stores in the brain using magnetic resonance imaging. Magn Reson Imaging. 2005;23(1):1-25.
1743.	Haacke EM, Cheng NYC, House MJ, Liu Q, Neelavalli J, Ogg RJ, et al. Imaging iron stores in the brain using magnetic resonance imaging. Magnetic Resonance Imaging. 2005.
1744.	Haacke EM, Chengb NYC, House MJ, Liu Q, Neelavalli J, Ogg RJ, et al. Imaging iron stores in the brain using magnetic resonance imaging. MAGNETIC RESONANCE IMAGING. 2005;23(1):1-25.
1745.	Haacke EM, Garbern J, Miao Y, Habib C, Liu M. Iron stores and cerebral veins in MS studied by susceptibility weighted imaging. INTERNATIONAL ANGIOLOGY. 2010;29(2):149-57.
1746.	Haacke EM, Garbern J, Miao Y, Habib C, Liu M. Iron stores and cerebral veins in MS studied by susceptibility weighted imaging. International Angiology. 2010.
1747.	Haacke EM, Garbern J, Miao Y, Habib C, Liu M. Iron stores and cerebral veins in MS studied by susceptibility weighted imaging. Int Angiol. 2010;29(2):149-57.
1748.	Haacke EM, Garbern J, Miao Y, Habib C, Liu M. Iron stores and cerebral veins in MS studied by susceptibility weighted imaging. INTERNATIONAL ANGIOLOGY. 2010;29(2):149-57.
1749.	Haacke EM, Ge Y, Sethi SK, Buch S, Zamboni P. An Overview of Venous Abnormalities Related to the Development of Lesions in Multiple Sclerosis. Front Neurol. 2021;12:561458.
1750.	Haacke EM, Khan O, Makki M, Hreha S, Selvan M, Latif Z, et al., editors. Visualization and quantification of iron in multiple sclerosis lesions using susceptibility-weighted imaging2008: SAGE PUBLICATIONS LTD 1 OLIVERS YARD, 55 CITY ROAD, LONDON EC1Y 1SP, ENGLAND.
1751.	Haacke EM, Khan O, Makki M, Hreha S, Selvan M, Latif Z, et al. Visualization and quantification of iron in multiple sclerosis lesions using susceptibility-weighted imaging. MULTIPLE SCLEROSIS. 2008;14:S106-S.
1752.	Haacke EM, Li M, Kish K. Susceptibility-weighted imaging.  Clinical MR Neuroimaging: Physiological and Functional Techniques, Second Edition2011.
1753.	Haacke EM, Makki M, Ge Y, Maheshwari M, Garbern J, Khan O, et al., editors. Correlating iron with T2 signal intensity in multiple sclerosis lesions using susceptibility weighted imaging2008.
1754.	Haacke EM, Makki M, Ge Y, Maheshwari M, Sehgal V, Hu J, et al. Characterizing iron deposition in multiple sclerosis lesions using susceptibility weighted imaging. Journal of Magnetic Resonance Imaging: An Official Journal of the International Society for Magnetic Resonance in Medicine. 2009;29(3):537-44.
1755.	Haacke EM, Makki M, Ge Y, Maheshwari M, Sehgal V, Hu J, et al. Characterizing iron deposition in multiple sclerosis lesions using susceptibility weighted imaging. Journal of Magnetic Resonance Imaging. 2009.
1756.	Haacke EM, Makki M, Ge Y, Maheshwari M, Sehgal V, Hu J, et al. Characterizing iron deposition in multiple sclerosis lesions using susceptibility weighted imaging. J Magn Reson Imaging. 2009;29(3):537-44.
1757.	Haacke EM, Makki M, Ge YL, Maheshwari M, Sehgal V, Hu JN, et al. Characterizing Iron Deposition in Multiple Sclerosis Lesions Using Susceptibility Weighted Imaging. JOURNAL OF MAGNETIC RESONANCE IMAGING. 2009;29(3):537-44.
1758.	Haacke EM, Makki M, Ge YL, Maheshwari M, Sehgal V, Hu JN, et al. Characterizing Iron Deposition in Multiple Sclerosis Lesions Using Susceptibility Weighted Imaging. JOURNAL OF MAGNETIC RESONANCE IMAGING. 2009;29(3):537-44.
1759.	Haacke EM, Makki M, Ge YL, Maheshwari M, Sehgal V, Hu JN, et al. Characterizing Iron Deposition in Multiple Sclerosis Lesions Using Susceptibility Weighted Imaging. JOURNAL OF MAGNETIC RESONANCE IMAGING. 2009;29(3):537-44.
1760.	Haas J, Bekeredjian-Ding I, Milkova M, Balint B, Schwarz A, Korporal M, et al. B cells undergo unique compartmentalized redistribution in multiple sclerosis. J Autoimmun. 2011;37(4):289-99.
1761.	Haas J, Bekeredjian-Ding I, Milkova M, Balinta B, Schwarz A, Korporal M, et al. B cells undergo unique compartmentalized redistribution in multiple sclerosis. JOURNAL OF AUTOIMMUNITY. 2011;37(4):289-99.
1762.	Haas J, Bekeredjian-Ding I, Milkova M, Balinta B, Schwarz A, Korporal M, et al. B cells undergo unique compartmentalized redistribution in multiple sclerosis. JOURNAL OF AUTOIMMUNITY. 2011;37(4):289-99.
1763.	Habek M, Adamec I, Pavliša G, Brinar VV. Diagnostic approach of patients with longitudinally extensive transverse myelitis. Acta Neurol Belg. 2012;112(1):39-43.
1764.	Habib CA. Imaging Iron Content In Patients With Multiple Sclerosis Using Magnetic Resonance Imaging. 2013.
1765.	Habib CA, Liu M, Bawany N, Garbern J, Krumbein I, Mentzel HJ, et al. Assessing abnormal iron content in the deep gray matter of patients with multiple sclerosis versus healthy controls. American Journal of Neuroradiology. 2012.
1766.	Habib CA, Liu M, Bawany N, Garbern J, Krumbein I, Mentzel HJ, et al. Assessing Abnormal Iron Content in the Deep Gray Matter of Patients with Multiple Sclerosis versus Healthy Controls. AMERICAN JOURNAL OF NEURORADIOLOGY. 2012;33(2):252-8.
1767.	Habib CA, Liu M, Bawany N, Garbern J, Krumbein I, Mentzel HJ, et al. Assessing abnormal iron content in the deep gray matter of patients with multiple sclerosis versus healthy controls. AJNR Am J Neuroradiol. 2012;33(2):252-8.
1768.	Habib CA, Liu M, Bawany N, Garbern J, Krumbein I, Mentzel HJ, et al. Assessing abnormal iron content in the deep gray matter of patients with multiple sclerosis versus healthy controls. American Journal of neuroradiology. 2012;33(2):252-8.
1769.	Habib CA, Zheng W, Mark Haacke E, Webb S, Nichol H, editors. Visualizing iron deposition in multiple sclerosis cadaver brains2010: American Institute of Physics.
1770.	Habib CA, Zheng W, Mark Haacke E, Webb S, Nichol H, editors. Visualizing iron deposition in multiple sclerosis cadaver brains2010.
1771.	Habib CA, Zheng WL, Haacke EM, Webb S, Nichol H. Visualizing Iron Deposition in Multiple Sclerosis Cadaver Brains.  6TH INTERNATIONAL CONFERENCE ON MEDICAL APPLICATIONS OF SYNCHROTRON RADIATION2010. p. 78-+.
1772.	Habib CA, Zheng WL, Haacke EM, Webb S, Nichol H. Visualizing Iron Deposition in Multiple Sclerosis Cadaver Brains.  6TH INTERNATIONAL CONFERENCE ON MEDICAL APPLICATIONS OF SYNCHROTRON RADIATION2010. p. 78-+.
1773.	Habib CA, Zheng WL, Haacke EM, Webb S, Nichol H. Visualizing Iron Deposition in Multiple Sclerosis Cadaver Brains.  6TH INTERNATIONAL CONFERENCE ON MEDICAL APPLICATIONS OF SYNCHROTRON RADIATION2010. p. 78-+.
1774.	Hachim MY, Elemam NM, Maghazachi AA. The beneficial and debilitating effects of environmental and microbial toxins, drugs, organic solvents and heavy metals on the onset and progression of multiple sclerosis. Toxins. 2019.
1775.	Hachim MY, Elemam NM, Maghazachi AA. The Beneficial and Debilitating Effects of Environmental and Microbial Toxins, Drugs, Organic Solvents and Heavy Metals on the Onset and Progression of Multiple Sclerosis. TOXINS. 2019;11(3).
1776.	Hachim MY, Elemam NM, Maghazachi AA. The Beneficial and Debilitating Effects of Environmental and Microbial Toxins, Drugs, Organic Solvents and Heavy Metals on the Onset and Progression of Multiple Sclerosis. Toxins (Basel). 2019;11(3).
1777.	Hachim MY, Elemam NM, Maghazachi AA. The beneficial and debilitating effects of environmental and microbial toxins, drugs, organic solvents and heavy metals on the onset and progression of multiple sclerosis. Toxins. 2019;11(3):147.
1778.	Haegele KF, Stueckle CA, Malin JP, Sindern E. Increase of CD8+T-effector memory cells in peripheral blood of patients with relapsing-remitting multiple sclerosis compared to healthy controls. JOURNAL OF NEUROIMMUNOLOGY. 2007;183(1-2):168-74.
1779.	Haegele KF, Stueckle CA, Malin JP, Sindern E. Increase of CD8+ T-effector memory cells in peripheral blood of patients with relapsing-remitting multiple sclerosis compared to healthy controls. J Neuroimmunol. 2007;183(1-2):168-74.
1780.	Haegele KF, Stueckle CA, Malin JP, Sindern E. Increase of CD8+T-effector memory cells in peripheral blood of patients with relapsing-remitting multiple sclerosis compared to healthy controls. JOURNAL OF NEUROIMMUNOLOGY. 2007;183(1-2):168-74.
1781.	Haegert DG. Clinical multiple sclerosis occurs at one end of a spectrum of CNS pathology: A modified threshold liability model leads to new ways of thinking about the cause of clinical multiple sclerosis. MEDICAL HYPOTHESES. 2005;65(2):232-7.
1782.	Haegert DG. Clinical multiple sclerosis occurs at one end of a spectrum of CNS pathology: A modified threshold liability model leads to new ways of thinking about the cause of clinical multiple sclerosis. MEDICAL HYPOTHESES. 2005;65(2):232-7.
1783.	Haegert DG. Clinical multiple sclerosis occurs at one end of a spectrum of CNS pathology: A modified threshold liability model leads to new ways of thinking about the cause of clinical multiple sclerosis. MEDICAL HYPOTHESES. 2005;65(2):232-7.
1784.	Hagedoorn M, Buunk BP, Kuijer RG, Ybema JF, De Jong GM, Wobbes T, et al. Caretaking of a partner with multiple sclerosis or cancer. Does it lead to burnout? GEDRAG EN GEZONDHEID. 1999;27:233-43.
1785.	Hagemeier J. The irony of iron: MRI and brain iron in multiple sclerosis. 2015.
1786.	Hagemeier J, Dwyer M, Bergsland N, Weinstock-Guttman B, Zivadinov R, Schweser F. Loss of Brain Iron is Linked to Disability in Multiple Sclerosis - The Difference Between Concentration and Mass of Iron. NEUROLOGY. 2019;92(15).
1787.	Hagemeier J, Dwyer M, Bergsland N, Weinstock-Guttman B, Zivadinov R, Schweser F. Loss of Brain Iron is Linked to Disability in Multiple Sclerosis-The Difference Between Concentration and Mass of Iron (P5. 2-011). AAN Enterprises; 2019.
1788.	Hagemeier J, Ferdinand S, Dwyer M, Polak P, Bergsland N, Weinstock-Guttman B, et al. Quantitative Susceptibility Mapping Study of Deep Gray Matter Iron at 3T in Large Cohort of Multiple Sclerosis Patients. NEUROLOGY. 2016;86.
1789.	Hagemeier J, Ferdinand S, Dwyer M, Polak P, Bergsland N, Weinstock-Guttman B, et al. Quantitative susceptibility mapping study of deep gray matter iron at 3T in large cohort of multiple sclerosis patients (P4. 162). AAN Enterprises; 2016.
1790.	Hagemeier J, Heininen-Brown M, Poloni GU, Bergsland N, Magnano CR, Durfee J, et al. Iron deposition in multiple sclerosis lesions measured by susceptibility-weighted imaging filtered phase: A case control study. Journal of Magnetic Resonance Imaging. 2012.
1791.	Hagemeier J, Heininen-Brown M, Poloni GU, Bergsland N, Magnano CR, Durfee J, et al. Iron deposition in multiple sclerosis lesions measured by susceptibility-weighted imaging filtered phase: A case control study. JOURNAL OF MAGNETIC RESONANCE IMAGING. 2012;36(1):73-83.
1792.	Hagemeier J, Heininen-Brown M, Poloni GU, Bergsland N, Magnano CR, Durfee J, et al. Iron deposition in multiple sclerosis lesions measured by susceptibility-weighted imaging filtered phase: a case control study. J Magn Reson Imaging. 2012;36(1):73-83.
1793.	Hagemeier J, Heininen‐Brown M, Poloni GU, Bergsland N, Magnano CR, Durfee J, et al. Iron deposition in multiple sclerosis lesions measured by susceptibility‐weighted imaging filtered phase: A case control study. Journal of Magnetic Resonance Imaging. 2012;36(1):73-83.
1794.	Hagemeier J, Ramanathan M, Schweser F, Dwyer MG, Lin F, Bergsland N, et al., editors. Quantitative susceptibility mapping and iron-related single nucleotide polymorphisms: an association with multiple sclerosis2016: SAGE PUBLICATIONS LTD 1 OLIVERS YARD, 55 CITY ROAD, LONDON EC1Y 1SP, ENGLAND.
1795.	Hagemeier J, Ramanathan M, Schweser F, Dwyer MG, Lin F, Bergsland N, et al. Quantitative susceptibility mapping and iron-related single nucleotide polymorphisms: an association with multiple sclerosis. MULTIPLE SCLEROSIS JOURNAL. 2016;22:207-.
1796.	Hagemeier J, Ramanathan M, Schweser F, Dwyer MG, Lin F, Bergsland N, et al. Iron-related gene variants and brain iron in multiple sclerosis and healthy individuals. NeuroImage: Clinical. 2018.
1797.	Hagemeier J, Ramanathan M, Schweser F, Dwyer MG, Lin F, Bergsland N, et al. Iron-related gene variants and brain iron in multiple sclerosis and healthy individuals. Neuroimage Clin. 2018;17:530-40.
1798.	Hagemeier J, Ramanathan M, Schweser F, Dwyer MG, Lin F, Bergsland N, et al. Iron-related gene variants and brain iron in multiple sclerosis and healthy individuals. NeuroImage: Clinical. 2018;17:530-40.
1799.	Hagemeier J, Ramanathan M, Schweser F, Dwyer MG, Lin FC, Bergsland N, et al. Iron-related gene variants and brain iron in multiple sclerosis and healthy individuals. NEUROIMAGE-CLINICAL. 2018;17:530-40.
1800.	Hagemeier J, Ramanathan M, Schweser F, Dwyer MG, Lin FC, Bergsland N, et al. Iron-related gene variants and brain iron in multiple sclerosis and healthy individuals. NEUROIMAGE-CLINICAL. 2018;17:530-40.
1801.	Hagemeier J, Ramanathan M, Schweser F, Dwyer MG, Lin FC, Bergsland N, et al. Iron-related gene variants and brain iron in multiple sclerosis and healthy individuals. NEUROIMAGE-CLINICAL. 2018;17:530-40.
1802.	Hagemeier J, Schweser F, Dwyer M, Polak P, Bergsland N, Weinstock-Guttman B, et al. Evolution of Brain Iron Levels in Multiple Sclerosis: A 2-Year Longitudinal Quantitative Susceptibility Mapping Study at 3T. NEUROLOGY. 2016;86.
1803.	Hagemeier J, Schweser F, Dwyer M, Polak P, Bergsland N, Weinstock-Guttman B, et al. Evolution of Brain Iron Levels in Multiple Sclerosis: A 2-Year Longitudinal Quantitative Susceptibility Mapping Study at 3T (P4. 163). AAN Enterprises; 2016.
1804.	Hagemeier J, Weinstock-Guttman B, Bergsland N, Heininen-Brown M, Carl E, Kennedy C, et al. Iron deposition on SWI-filtered phase in the subcortical deep gray matter of patients with clinically isolated syndrome may precede structure-specific atrophy. AJNR Am J Neuroradiol. 2012;33(8):1596-601.
1805.	Hagemeier J, Weinstock-Guttman B, Heininen-Brown M, Poloni GU, Bergsland N, Schirda C, et al. Gray matter SWI-filtered phase and atrophy are linked to Disability in MS. Frontiers in Bioscience - Elite. 2013.
1806.	Hagemeier J, Weinstock-Guttman B, Heininen-Brown M, Poloni GU, Bergsland N, Schirda C, et al. Gray matter SWI-filtered phase and atrophy are linked to disability in MS. Front Biosci (Elite Ed). 2013;5(2):525-32.
1807.	Hagemeier J, Yeh EA, Brown MH, Bergsland N, Dwyer MG, Carl E, et al. Iron content of the pulvinar nucleus of the thalamus is increased in adolescent multiple sclerosis. MULTIPLE SCLEROSIS JOURNAL. 2013;19(5):567-76.
1808.	Hagemeier J, Yeh EA, Brown MH, Bergsland N, Dwyer MG, Carl E, et al. Iron content of the pulvinar nucleus of the thalamus is increased in adolescent multiple sclerosis. Multiple Sclerosis Journal. 2013.
1809.	Hagemeier J, Yeh EA, Brown MH, Bergsland N, Dwyer MG, Carl E, et al. Iron content of the pulvinar nucleus of the thalamus is increased in adolescent multiple sclerosis. MULTIPLE SCLEROSIS JOURNAL. 2013;19(5):567-76.
1810.	Hagemeier J, Yeh EA, Brown MH, Bergsland N, Dwyer MG, Carl E, et al. Iron content of the pulvinar nucleus of the thalamus is increased in adolescent multiple sclerosis. Mult Scler. 2013;19(5):567-76.
1811.	Hagemeier J, Yeh EA, Brown MH, Bergsland N, Dwyer MG, Carl E, et al. Iron content of the pulvinar nucleus of the thalamus is increased in adolescent multiple sclerosis. MULTIPLE SCLEROSIS JOURNAL. 2013;19(5):567-76.
1812.	Hagemeier J, Yeh EA, Brown MH, Bergsland N, Dwyer MG, Carl E, et al. Iron content of the pulvinar nucleus of the thalamus is increased in adolescent multiple sclerosis. Multiple Sclerosis Journal. 2013;19(5):567-76.
1813.	Hagemeier J, Yeh EA, Heininen-Brown M, Bergsland N, Dwyer M, Carl E, et al. Iron Deposition in the Subcortical Deep-Gray Matter of Pediatric Multiple Sclerosis Patients. NEUROLOGY. 2012;78.
1814.	Hagemeier J, Yeh EA, Heininen-Brown M, Bergsland N, Dwyer M, Carl E, et al. Iron Deposition in the Subcortical Deep-Gray Matter of Pediatric Multiple Sclerosis Patients (S50. 006). AAN Enterprises; 2012.
1815.	Hagemeier J, Zivadinov R, Dwyer MG, Polak P, Bergsland N, Weinstock-Guttman B, et al. Changes of deep gray matter magnetic susceptibility over 2 years in multiple sclerosis and healthy control brain. NEUROIMAGE-CLINICAL. 2018;18:1007-16.
1816.	Hagemeier J, Zivadinov R, Dwyer MG, Polak P, Bergsland N, Weinstock-Guttman B, et al. Changes of deep gray matter magnetic susceptibility over 2 years in multiple sclerosis and healthy control brain. NeuroImage: Clinical. 2018.
1817.	Hagemeier J, Zivadinov R, Dwyer MG, Polak P, Bergsland N, Weinstock-Guttman B, et al. Changes of deep gray matter magnetic susceptibility over 2 years in multiple sclerosis and healthy control brain. Neuroimage Clin. 2018;18:1007-16.
1818.	Hagemeier J, Zivadinov R, Dwyer MG, Polak P, Bergsland N, Weinstock-Guttman B, et al. Changes of deep gray matter magnetic susceptibility over 2 years in multiple sclerosis and healthy control brain. NEUROIMAGE-CLINICAL. 2018;18:1007-16.
1819.	Hagens M, van Berckel B, Barkhof F. Novel MRI and PET markers of neuroinflammation in multiple sclerosis. CURRENT OPINION IN NEUROLOGY. 2016;29(3):229-36.
1820.	Hagens M, Van Berckel B, Barkhof F. Novel MRI and PET markers of neuroinflammation in multiple sclerosis. Current Opinion in Neurology. 2016.
1821.	Hagens M, van Berckel B, Barkhof F. Novel MRI and PET markers of neuroinflammation in multiple sclerosis. Curr Opin Neurol. 2016;29(3):229-36.
1822.	Hagens MH, Killestein J, Yaqub MM, van Dongen GA, Lammertsma AA, Barkhof F, et al. Cerebral rituximab uptake in multiple sclerosis: A (89)Zr-immunoPET pilot study. Mult Scler. 2018;24(4):543-5.
1823.	Hagihara K, Kita A, Mizukura A, Yao M, Kitai Y, Kunoh T, et al. Fingolimod (FTY720) Stimulates Ca2+/Calcineurin Signaling in Fission Yeast. PLOS ONE. 2013;8(12).
1824.	Hagihara K, Kita A, Mizukura A, Yao M, Kitai Y, Kunoh T, et al. Fingolimod (FTY720) stimulates Ca2+/calcineurin signaling in fission yeast. PLoS ONE. 2013.
1825.	Hagihara K, Kita A, Mizukura A, Yao M, Kitai Y, Kunoh T, et al. Fingolimod (FTY720) stimulates Ca(2+)/calcineurin signaling in fission yeast. PLoS One. 2013;8(12):e81907.
1826.	Hagihara K, Kita A, Mizukura A, Yao M, Kitai Y, Kunoh T, et al. Fingolimod (FTY720) Stimulates Ca2+/Calcineurin Signaling in Fission Yeast. PLOS ONE. 2013;8(12).
1827.	Haider L. Inflammation, iron, energy failure, and oxidative stress in the pathogenesis of multiple sclerosis. Oxid Med Cell Longev. 2015; 2015: 725370.
1828.	Haider L. Pathogenesis of Multiple Sclerosis–Oxidative Stress, Inflammation, Iron and Energy Failure.
1829.	Haider L. Inflammation, Iron, Energy Failure, and Oxidative Stress in the Pathogenesis of Multiple Sclerosis. OXIDATIVE MEDICINE AND CELLULAR LONGEVITY. 2015;2015.
1830.	Haider L. Inflammation, Iron, Energy Failure, and Oxidative Stress in the Pathogenesis of Multiple Sclerosis. Oxidative Medicine and Cellular Longevity. 2015.
1831.	Haider L. Inflammation, Iron, Energy Failure, and Oxidative Stress in the Pathogenesis of Multiple Sclerosis. OXIDATIVE MEDICINE AND CELLULAR LONGEVITY. 2015;2015.
1832.	Haider L. Inflammation, Iron, Energy Failure, and Oxidative Stress in the Pathogenesis of Multiple Sclerosis. Oxid Med Cell Longev. 2015;2015:725370.
1833.	Haider L. Inflammation, iron, energy failure, and oxidative stress in the pathogenesis of multiple sclerosis. Oxidative medicine and cellular longevity. 2015;2015.
1834.	Haider L, Simeonidou C, Steinberger G, Hametner S, Grigoriadis N, Deretzi G, et al. Multiple sclerosis deep grey matter: the relation between demyelination, neurodegeneration, inflammation and iron. JOURNAL OF NEUROLOGY NEUROSURGERY AND PSYCHIATRY. 2014;85(12):1386-95.
1835.	Haider L, Simeonidou C, Steinberger G, Hametner S, Grigoriadis N, Deretzi G, et al. Multiple sclerosis deep grey matter: The relation between demyelination, neurodegeneration, inflammation and iron. Journal of Neurology, Neurosurgery and Psychiatry. 2014.
1836.	Haider L, Simeonidou C, Steinberger G, Hametner S, Grigoriadis N, Deretzi G, et al. Multiple sclerosis deep grey matter: the relation between demyelination, neurodegeneration, inflammation and iron. JOURNAL OF NEUROLOGY NEUROSURGERY AND PSYCHIATRY. 2014;85(12):1386-95.
1837.	Haider L, Simeonidou C, Steinberger G, Hametner S, Grigoriadis N, Deretzi G, et al. Multiple sclerosis deep grey matter: the relation between demyelination, neurodegeneration, inflammation and iron. J Neurol Neurosurg Psychiatry. 2014;85(12):1386-95.
1838.	Haider L, Simeonidou C, Steinberger G, Hametner S, Grigoriadis N, Deretzi G, et al. Multiple sclerosis deep grey matter: the relation between demyelination, neurodegeneration, inflammation and iron. JOURNAL OF NEUROLOGY NEUROSURGERY AND PSYCHIATRY. 2014;85(12):1386-95.
1839.	Haider L, Simeonidou C, Steinberger G, Hametner S, Grigoriadis N, Deretzi G, et al. Multiple sclerosis deep grey matter: the relation between demyelination, neurodegeneration, inflammation and iron. Journal of Neurology, Neurosurgery & Psychiatry. 2014;85(12):1386-95.
1840.	Hajiabadi MM, Jakobs M, Unterberg A, Ahmadi R. Wireless Subcutaneous Trigeminal Nerve Field Stimulation for Refractory Trigeminal Pain: A Single Center Experience. Neuromodulation. 2021;24(6):1115-20.
1841.	Hajipour MJ, Ghasemi F, Aghaverdi H, Raoufi M, Linne U, Atyabi F, et al. Sensing of Alzheimer's Disease and Multiple Sclerosis Using Nano-Bio Interfaces. J Alzheimers Dis. 2017;59(4):1187-202.
1842.	Hall ED. Novel inhibitors of iron‐dependent lipid peroxidation for neurodegenerative disorders. Annals of Neurology. 1992.
1843.	Hall ED. Novel inhibitors of iron-dependent lipid peroxidation for neurodegenerative disorders. Ann Neurol. 1992;32 Suppl:S137-42.
1844.	Haller S, Haacke EM, Thurnher MM, Barkhof F. Susceptibility-weighted Imaging: Technical Essentials and Clinical Neurologic Applications. RADIOLOGY. 2021;299(1):3-26.
1845.	Haller S, Haacke EM, Thurnher MM, Barkhof F. Susceptibility-weighted imaging: Technical essentials and clinical neurologic applications. Radiology. 2021.
1846.	Haller S, Haacke EM, Thurnher MM, Barkhof F. Susceptibility-weighted Imaging: Technical Essentials and Clinical Neurologic Applications. Radiology. 2021;299(1):3-26.
1847.	Haller S, Haacke EM, Thurnher MM, Barkhof F. Susceptibility-weighted Imaging: Technical Essentials and Clinical Neurologic Applications. RADIOLOGY. 2021;299(1):3-26.
1848.	Hallpike JF, Adams CW, Bayliss OB. Histochemistry of myelin. 8. Proteolytic activity around multiple sclerosis plaques. Histochem J. 1970;2(3):199-208.
1849.	Hamdy E, Galeel AA, Ramadan I, Gaber D, Mustafa H, Mekky J. Iron deposition in multiple sclerosis: overall load or distribution alteration? Eur Radiol Exp. 2022;6(1):49.
1850.	Hametner S, Boyd E, Pawate S, Ikonomidou V, Smith S, Lassmann H, et al. Susceptibility Contrast in High Field MRI of Multiple Sclerosis Brains as a Function of Tissue Iron, Myelin, Calcium and Axon Content (P6. 128). AAN Enterprises; 2015.
1851.	Hametner S, Dal Bianco A, Trattnig S, Lassmann H. Iron related changes in MS lesions and their validity to characterize MS lesion types and dynamics with Ultra-high field magnetic resonance imaging. BRAIN PATHOLOGY. 2018;28(5):743-9.
1852.	Hametner S, Dal Bianco A, Trattnig S, Lassmann H. Iron related changes in MS lesions and their validity to characterize MS lesion types and dynamics with Ultra-high field magnetic resonance imaging. Brain Pathology. 2018.
1853.	Hametner S, Dal Bianco A, Trattnig S, Lassmann H. Iron related changes in MS lesions and their validity to characterize MS lesion types and dynamics with Ultra-high field magnetic resonance imaging. Brain Pathol. 2018;28(5):743-9.
1854.	Hametner S, Dal Bianco A, Trattnig S, Lassmann H. Iron related changes in MS lesions and their validity to characterize MS lesion types and dynamics with Ultra-high field magnetic resonance imaging. BRAIN PATHOLOGY. 2018;28(5):743-9.
1855.	Hametner S, Dal-Bianco A, Grabner G, Trattnig S, Bagnato F, Lassmann H, editors. Iron rings around plaques of multiple sclerosis patients2015: SAGE PUBLICATIONS LTD 1 OLIVERS YARD, 55 CITY ROAD, LONDON EC1Y 1SP, ENGLAND.
1856.	Hametner S, Dal-Bianco A, Grabner G, Trattnig S, Bagnato F, Lassmann H. Iron rings around plaques of multiple sclerosis patients. MULTIPLE SCLEROSIS JOURNAL. 2015;21:68-.
1857.	Hametner S, Lassmann H, editors. Glial iron dysregulation in multiple sclerosis brains2017: SAGE PUBLICATIONS LTD 1 OLIVERS YARD, 55 CITY ROAD, LONDON EC1Y 1SP, ENGLAND.
1858.	Hametner S, Lassmann H. Glial iron dysregulation in multiple sclerosis brains. MULTIPLE SCLEROSIS JOURNAL. 2017;23:489-90.
1859.	Hametner S, Wesseling C, Schuh C, Lassmann H, editors. Ferritin and iron in multiple sclerosis lesions2010: WILEY-BLACKWELL PUBLISHING, INC COMMERCE PLACE, 350 MAIN ST, MALDEN 02148 ….
1860.	Hametner S, Wesseling C, Schuh C, Lassmann H. Ferritin and iron in multiple sclerosis lesions. BRAIN PATHOLOGY. 2010;20:86-.
1861.	Hametner S, Wimmer I, Haider L, Brück W, Lassmann H. Iron and oxidative damage in the multiple sclerosis brain. Journal of the Neurological Sciences. 2013;333:e386-e7.
1862.	Hametner S, Wimmer I, Haider L, Pfeifenbring S, Bruck W, Lassmann H. Iron and Neurodegeneration in the Multiple Sclerosis Brain. ANNALS OF NEUROLOGY. 2013;74(6):848-61.
1863.	Hametner S, Wimmer I, Haider L, Pfeifenbring S, Brück W, Lassmann H. Iron and neurodegeneration in the multiple sclerosis brain. Annals of Neurology. 2013.
1864.	Hametner S, Wimmer I, Haider L, Pfeifenbring S, Bruck W, Lassmann H. Iron and Neurodegeneration in the Multiple Sclerosis Brain. ANNALS OF NEUROLOGY. 2013;74(6):848-61.
1865.	Hametner S, Wimmer I, Haider L, Pfeifenbring S, Bruck W, Lassmann H. Iron and oxidative damage in the multiple sclerosis brain. MULTIPLE SCLEROSIS JOURNAL. 2013;19(11):9-.
1866.	Hametner S, Wimmer I, Haider L, Pfeifenbring S, Brück W, Lassmann H. Iron and neurodegeneration in the multiple sclerosis brain. Ann Neurol. 2013;74(6):848-61.
1867.	Hametner S, Wimmer I, Haider L, Pfeifenbring S, Bruck W, Lassmann H. Iron and Neurodegeneration in the Multiple Sclerosis Brain. ANNALS OF NEUROLOGY. 2013;74(6):848-61.
1868.	Hametner S, Wimmer I, Haider L, Pfeifenbring S, Brück W, Lassmann H. Iron and neurodegeneration in the multiple sclerosis brain. Annals of neurology. 2013;74(6):848-61.
1869.	Hamilton AM, Mallett C, Foster PJ. High-resolution MRI and nanoparticles: the future of brain imaging. FUTURE NEUROLOGY. 2014;9(2):211-25.
1870.	Hamilton AM, Mallett C, Foster PJ. High-resolution MRI and nanoparticles: The future of brain imaging. Future Neurology. 2014.
1871.	Hamilton NB, Kolodziejczyk K, Kougioumtzidou E, Attwell D. Proton-gated Ca(2+)-permeable TRP channels damage myelin in conditions mimicking ischaemia. Nature. 2016;529(7587):523-7.
1872.	Hammond KE, Metcalf M, Carvajal L, Okuda DT, Srinivasan R, Vigneron D, et al. Quantitative In Vivo Magnetic Resonance Imaging of Multiple Sclerosis at 7 Tesla with Sensitivity to Iron. ANNALS OF NEUROLOGY. 2008;64(6):707-13.
1873.	Hammond KE, Metcalf M, Carvajal L, Okuda DT, Srinivasan R, Vigneron D, et al. Quantitative in vivo magnetic resonance imaging of multiple sclerosis at 7 Tesla with sensitivity to iron. Annals of Neurology. 2008.
1874.	Hammond KE, Metcalf M, Carvajal L, Okuda DT, Srinivasan R, Vigneron D, et al. Quantitative In Vivo Magnetic Resonance Imaging of Multiple Sclerosis at 7 Tesla with Sensitivity to Iron. ANNALS OF NEUROLOGY. 2008;64(6):707-13.
1875.	Hammond KE, Metcalf M, Carvajal L, Okuda DT, Srinivasan R, Vigneron D, et al. Quantitative in vivo magnetic resonance imaging of multiple sclerosis at 7 Tesla with sensitivity to iron. Ann Neurol. 2008;64(6):707-13.
1876.	Hammond KE, Metcalf M, Carvajal L, Okuda DT, Srinivasan R, Vigneron D, et al. Quantitative In Vivo Magnetic Resonance Imaging of Multiple Sclerosis at 7 Tesla with Sensitivity to Iron. ANNALS OF NEUROLOGY. 2008;64(6):707-13.
1877.	Hammond KE, Metcalf M, Carvajal L, Okuda DT, Srinivasan R, Vigneron D, et al. Quantitative in vivo magnetic resonance imaging of multiple sclerosis at 7 Tesla with sensitivity to iron. Annals of Neurology: Official Journal of the American Neurological Association and the Child Neurology Society. 2008;64(6):707-13.
1878.	Hammond KE, Metcalf M, Okuda DT, Nelson SJ, Vigneron DB, Pelletier D, editors. In vivo high resolution MR imaging at 7T of multiple sclerosis with sensitivity to iron2008: LIPPINCOTT WILLIAMS & WILKINS TWO COMMERCE SQ, 2001 MARKET ST, PHILADELPHIA ….
1879.	Hammond KE, Metcalf M, Okuda DT, Nelson SJ, Vigneron DB, Pelletier D. In vivo high resolution MR imaging at 7T of multiple sclerosis with sensitivity to iron. NEUROLOGY. 2008;70(11):A8-A.
1880.	Harandi AA, Shahbeigi S, Pakdaman H, Fereshtehnejad SM, Nikravesh E, Jalilzadeh R. Association of serum 25(OH) vitamin D-3 concentration with severity of multiple sclerosis. IRANIAN JOURNAL OF NEUROLOGY. 2012;11(2):54-8.
1881.	Harandi AA, Shahbeigi S, Pakdaman H, Fereshtehnejad SM, Nikravesh E, Jalilzadeh R. Association of serum 25(OH) vitamin D3 concentration with severity of multiple sclerosis. Iran J Neurol. 2012;11(2):54-8.
1882.	Harandi AA, Shahbeigi S, Pakdaman H, Fereshtehnejad SM, Nikravesh E, Jalilzadeh R. Association of serum 25(OH) vitamin D-3 concentration with severity of multiple sclerosis. IRANIAN JOURNAL OF NEUROLOGY. 2012;11(2):54-8.
1883.	Harauz G, Libich DS. The classic basic protein of myelin--conserved structural motifs and the dynamic molecular barcode involved in membrane adhesion and protein-protein interactions. Curr Protein Pept Sci. 2009;10(3):196-215.
1884.	Harbige LS, Pinto E, Xiang M, Shareif MK. Circulating plasma cytokines, zinc, copper, vitamins A and E in multiple sclerosis patients and healthy controls. PROCEEDINGS OF THE NUTRITION SOCIETY. 2011;70(OCE2):E36-E.
1885.	Harbige LS, Pinto E, Xiang M, Shareif MK. Circulating plasma cytokines, zinc, copper, vitamins A and E in multiple sclerosis patients and healthy controls. Proceedings of the Nutrition Society. 2011;70(OCE2).
1886.	Harilal S, Jose J, Parambi DGT, Kumar R, Unnikrishnan MK, Uddin MS, et al. Revisiting the blood-brain barrier: A hard nut to crack in the transportation of drug molecules. Brain Research Bulletin. 2020.
1887.	Harilal S, Jose J, Parambi DGT, Kumar R, Unnikrishnan MK, Uddin MS, et al. Revisiting the blood-brain barrier: A hard nut to crack in the transportation of drug molecules. Brain Res Bull. 2020;160:121-40.
1888.	Harilal S, Jose J, Parambi DT, Kumar R, Unnikrishnan MK, Uddin MS, et al. Revisiting the blood-brain barrier: A hard nut to crack in the transportation of drug molecules. BRAIN RESEARCH BULLETIN. 2020;160:121-40.
1889.	Haris PI. Correction: Multiple Sclerosis in the Mount Etna Region: Possible Role of Volcanogenic Trace Elements. 2014.
1890.	Harrer A, Pilz G, Wipfler P, Oppermann K, Sellner J, Hitzl W, et al. High interindividual variability in the CD4/CD8 T cell ratio and natalizumab concentration levels in the cerebrospinal fluid of patients with multiple sclerosis. CLINICAL AND EXPERIMENTAL IMMUNOLOGY. 2015;180(3):383-92.
1891.	Harrer A, Pilz G, Wipfler P, Oppermann K, Sellner J, Hitzl W, et al. High interindividual variability in the CD4/CD8 T cell ratio and natalizumab concentration levels in the cerebrospinal fluid of patients with multiple sclerosis. Clin Exp Immunol. 2015;180(3):383-92.
1892.	Harrer A, Pilz G, Wipfler P, Oppermann K, Sellner J, Hitzl W, et al. High interindividual variability in the CD4/CD8 T cell ratio and natalizumab concentration levels in the cerebrospinal fluid of patients with multiple sclerosis. CLINICAL AND EXPERIMENTAL IMMUNOLOGY. 2015;180(3):383-92.
1893.	Harrer C, Otto F, Pilz G, Haschke-Becher E, Trinka E, Hitzl W, et al. The CXCL13/CXCR5-chemokine axis in neuroinflammation: evidence of CXCR5+CD4 T cell recruitment to CSF. FLUIDS AND BARRIERS OF THE CNS. 2021;18(1).
1894.	Harrer C, Otto F, Pilz G, Haschke-Becher E, Trinka E, Hitzl W, et al. The CXCL13/CXCR5-chemokine axis in neuroinflammation: evidence of CXCR5+CD4 T cell recruitment to CSF. Fluids Barriers CNS. 2021;18(1):40.
1895.	Harrer C, Otto F, Pilz G, Haschke-Becher E, Trinka E, Hitzl W, et al. The CXCL13/CXCR5-chemokine axis in neuroinflammation: evidence of CXCR5+CD4 T cell recruitment to CSF. FLUIDS AND BARRIERS OF THE CNS. 2021;18(1).
1896.	Harrington MG, Merril CR. Two-dimensional electrophoresis and "ultrasensitive" silver staining of cerebrospinal fluid proteins in neurological diseases. Clin Chem. 1984;30(12 Pt 1):1933-7.
1897.	Harris PA, Faucher N, George N, Eidam PM, King BW, White GV, et al. Discovery and Lead-Optimization of 4,5-Dihydropyrazoles as Mono-Kinase Selective, Orally Bioavailable and Efficacious Inhibitors of Receptor Interacting Protein 1 (RIP1) Kinase. JOURNAL OF MEDICINAL CHEMISTRY. 2019;62(10):5096-110.
1898.	Harris PA, Faucher N, George N, Eidam PM, King BW, White GV, et al. Discovery and Lead-Optimization of 4,5-Dihydropyrazoles as Mono-Kinase Selective, Orally Bioavailable and Efficacious Inhibitors of Receptor Interacting Protein 1 (RIP1) Kinase. JOURNAL OF MEDICINAL CHEMISTRY. 2019;62(10):5096-110.
1899.	Harrison DM, Li X, Liu H, Jones CK, Caffo B, Calabresi PA, et al., editors. High Field, Multiparametric Susceptibility MRI Measures of Myelin and Iron Loss in Multiple Sclerosis2014: WILEY-BLACKWELL 111 RIVER ST, HOBOKEN 07030-5774, NJ USA.
1900.	Harrison DM, Li X, Liu H, Jones CK, Caffo B, Calabresi PA, et al. Lesion Heterogeneity on High-Field Susceptibility MRI Is Associated with Multiple Sclerosis Severity. AMERICAN JOURNAL OF NEURORADIOLOGY. 2016;37(8):1447-53.
1901.	Harrison DM, Li X, Liu H, Jones CK, Caffo B, Calabresi PA, et al. Lesion heterogeneity on high-field susceptibility MRI Is associated with multiple sclerosis severity. American Journal of Neuroradiology. 2016.
1902.	Harrison DM, Li X, Liu H, Jones CK, Caffo B, Calabresi PA, et al. Lesion Heterogeneity on High-Field Susceptibility MRI Is Associated with Multiple Sclerosis Severity. AJNR Am J Neuroradiol. 2016;37(8):1447-53.
1903.	Harrison DM, Li X, Liu H, Jones CK, Caffo B, Calabresi PA, et al. Lesion Heterogeneity on High-Field Susceptibility MRI Is Associated with Multiple Sclerosis Severity. AMERICAN JOURNAL OF NEURORADIOLOGY. 2016;37(8):1447-53.
1904.	Harrison DM, Li X, Liu HJ, Jones CK, Caffo B, Calabresi PA, et al. High Field, Multiparametric Susceptibility MRI Measures of Myelin and Iron Loss in Multiple Sclerosis. ANNALS OF NEUROLOGY. 2014;76:S113-S.
1905.	Hasan KM, Narayana PA. Magnetic resonance imaging-based quantitative iron mapping at 7-Tesla remains to be elusive in multiple sclerosis. Annals of Neurology. 2009.
1906.	Hasan KM, Narayana PA. Magnetic Resonance Imaging-Based Quantitative Iron Mapping at 7-Tesla Remains to Be Elusive in Multiple Sclerosis. ANNALS OF NEUROLOGY. 2009;66(6):867-.
1907.	Hasan KM, Narayana PA. Magnetic resonance imaging-based quantitative iron mapping at 7-tesla remains to be elusive in multiple sclerosis. Ann Neurol. 2009;66(6):867; author reply 
1908.	Hasan KM, Narayana PA. Magnetic resonance imaging-based quantitative iron mapping at 7-tesla remains to be elusive in multiple sclerosis. Annals of neurology. 2009;66(6):867-author.
1909.	Häsänen E, Kinnunen E, Alhonen P. Relationships between the prevalence of multiple sclerosis and some physical and chemical properties of soil. Science of the Total Environment, The. 1986.
1910.	Häsänen E, Kinnunen E, Alhonen P. Relationships between the prevalence of multiple sclerosis and some physical and chemical properties of soil. Sci Total Environ. 1986;58(3):263-72.
1911.	Hasani Nourian Y, Beh-Pajooh A, Aliomrani M, Amini M, Sahraian MA, Hosseini R, et al. Changes in DNA methylation in APOE and ACKR3 genes in multiple sclerosis patients and the relationship with their heavy metal blood levels. NeuroToxicology. 2021.
1912.	Hasani Nourian Y, Beh-Pajooh A, Aliomrani M, Amini M, Sahraian MA, Hosseini R, et al. Changes in DNA methylation in APOE and ACKR3 genes in multiple sclerosis patients and the relationship with their heavy metal blood levels. Neurotoxicology. 2021;87:182-7.
[truncated: 747,924 more chars]
